# Immunomodulatory Mechanism of Baiyaojian Decoction on Periodontitis: Network Pharmacology, Single‐Cell RNA Sequencing and Molecular Docking

**DOI:** 10.1111/jcmm.71034

**Published:** 2026-01-28

**Authors:** Bing‐jun Chen, Ming‐ming Li, Zhao‐yu Zheng, Wen‐qin Jin, Zhao Jin, Yu‐ling Zuo

**Affiliations:** ^1^ Department of Stomatology Hospital of Chengdu University of Traditional Chinese Medicine Chengdu People's Republic of China; ^2^ Chengdu University of Traditional Chinese Medicine Chengdu People's Republic of China

**Keywords:** molecular docking, network pharmacology, periodontitis, single‐cell RNA sequencing, traditional Chinese medicine

## Abstract

Periodontitis is one of the most common oral inflammatory diseases. Baiyaojian decoction, known for its prominent immunomodulatory and anti‐inflammatory properties, shows significant potential in treating periodontitis, though its molecular mechanisms remain unknown. The active ingredients and therapeutic targets were determined by integrating multiple databases. The protein–protein interaction network was constructed by the STRING platform. Bulk RNA seq data of GSE16134 were included and GO enrichment, GSEA and CIBERSORT algorithm were employed to investigate the immune microenvironment in periodontitis. Single‐cell RNA seq data of GSE152042 and GSE171213 were integrated by harmony; the cell–cell communication network was analysed by CellChat, and the differentiation trajectory was constructed by monocle3. Molecular docking was carried out using AutoDockTools, AutoDock Vina and PyMOL. Experimental validation was performed via qRT‐PCR, CCK‐8 assay, flow cytometry and ELISA. Twenty‐seven active ingredients and 207 therapeutic targets were obtained. Thirty‐one core therapeutic targets were identified. The infiltration of plasma cells, neutrophils, macrophages and mast cells was significantly enhanced in periodontitis tissues. Twenty‐eight of 31 core therapeutic targets were involved in their infiltration, differentiation and pro‐inflammatory activities. Molecular docking suggested stable bindings between ingredients and therapeutic targets. Experimental validation confirmed the elevated infiltration of above immune cells and demonstrated the anti‐inflammatory properties and target modulation capabilities of key ingredients including Coumestrol, Diosmetin and Gallicin. Baiyaojian decoction may exert immunomodulatory and anti‐inflammatory effects to treat periodontitis through multi‐ingredient and multi‐target mechanisms.

## 1 Introduction

Periodontitis is a common inflammatory disease characterised by progressive destruction of periodontal tissues, manifesting as attachment loss, alveolar bone resorption and periodontal pockets formation [1]. It represents a significant public health concern due to its high prevalence and widespread distribution [2]. Furthermore, periodontitis is closely associated with various systemic diseases, including cardiovascular diseases [3], diabetes [4], respiratory diseases [5], chronic kidney disease [6] and cancers [7]. The occurrence and progression of periodontitis are the result of a complex interplay between bacterial biofilm and the host's immune response [8]. The metabolic products and toxins produced by bacteria can stimulate the infiltration and activation of various immune cells that release a myriad of inflammatory cytokines [9], matrix metalloproteinases (MMPs) [10] and reactive oxygen species (ROS) [11], which shapes the inflammatory environment and leads to periodontal tissues destruction. The dysregulated immune response induced by persistent bacterial stimulation is believed to play a crucial role in driving periodontitis progression and periodontal tissues destruction [12, 13]. Current treatment strategies primarily focus on mechanically removing bacterial biofilm, alongside adjunctive antibiotic therapy when necessary [14]. However, these conventional approaches overlook the pivotal role of the immune response in periodontitis and are unable to rectify the dysregulated immune response [15, 16]. And as a result, inflammation frequently persists after treatment [17], impeding the recovery and regeneration of periodontal tissues [18] and leading to recurrence [19]. Consequently, there is an urgent need to explore the regulatory mechanisms of immune response dysregulation in periodontitis [20]. Such insights may provide more comprehensive approaches with immunomodulatory effect to treat this multifaceted disease [21].

Traditional Chinese Medicine (TCM), comprising a variety of active ingredients, can exert therapeutic effects through multi‐target and multi‐pathway mechanisms [22], which offers innovative strategies for treating a range of diseases [23]. In recent years, the application of TCM in the management of periodontitis has garnered increasing attention due to several advantages, such as low toxicity, minimal adverse reactions, cost‐effectiveness and the potential for long‐term use [24, 25, 26, 27]. Among them, Baiyaojian decoction stands out for its prominent anti‐inflammatory properties. As a TCM formulation, Baiyaojian decoction consists of Baiyaojian, Pugongyin and Fangfeng. Baiyaojian, derived from *Galla Chinensis* fermentation [28], has been employed in a range of inflammatory conditions, including inflammation in oral diseases, ulcerative colitis and chronic gastritis [29, 30]. Pugongying, sourced from the dried whole herbs of *Taraxacum genus*, has been reported to exhibit anti‐inflammatory and antioxidant properties in various disease [31, 32]. Its anti‐inflammatory effects have proven beneficial not only in treating periodontitis [33, 34] but also in addressing conditions like atherosclerosis [35] and lung inflammation [36]. Fangfeng, derived from the dried root of *Saposhnikovia divaricata*, is recognised for its immunomodulatory role and applicable in the treatment of periodontitis [37] as well as rheumatoid arthritis [38, 39] and colorectal cancer [40]. The cumulative evidence suggests that Baiyaojian decoction holds promising immunomodulatory and anti‐inflammatory potential for managing periodontitis. However, the specific active ingredients in Baiyaojian decoction, along with the immune cells, target genes and signalling pathways they act upon remain poorly understood. Such knowledge gaps hinder our comprehensive understanding of how Baiyaojian decoction ameliorates immune dysregulation in periodontitis at cellular and molecular levels, which is crucial for unlocking its full therapeutic potential.

**FIGURE 1:**
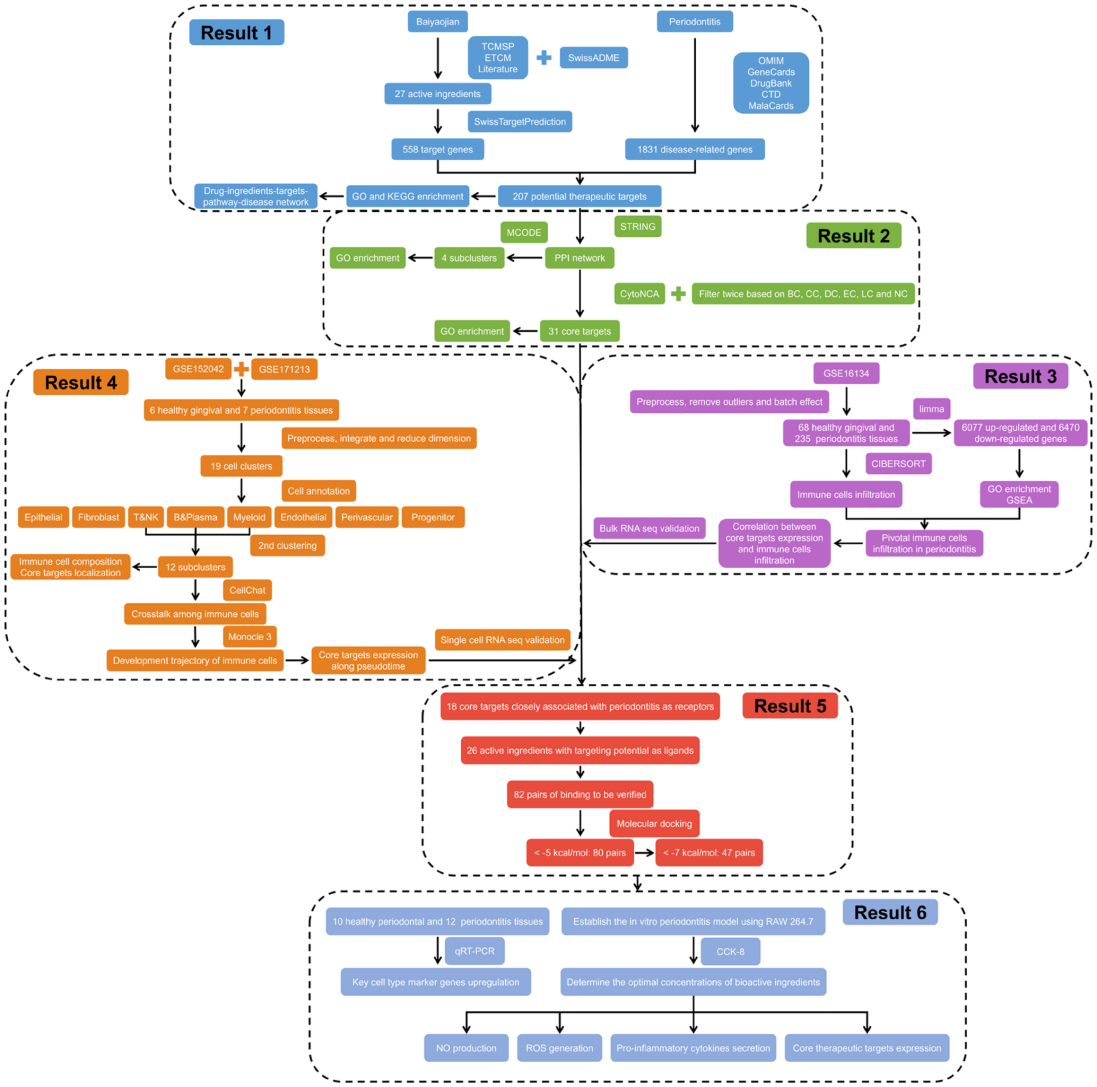
The schematic diagram of this study.

To bridge this gap, network pharmacology was employed to elucidate the active ingredients of Baiyaojian decoction and their potential therapeutic targets and signalling pathways for periodontitis, thus constructing a comprehensive drug‐ingredient‐target‐pathway‐diseases network. Then, single‐cell RNA sequencing was utilised to identify immune cell types closely associated with periodontitis progression. This approach revealed dynamic functional changes within these immune cells, demonstrating the roles of therapeutic targets in immune cell infiltration, differentiation and maturation in periodontitis. Furthermore, molecular docking was performed to investigate the binding sites and affinities of the active ingredients to their target proteins, providing valuable insights into the molecular interactions underlying the therapeutic efficacy of Baiyaojian decoction. Experimental validation confirmed the elevated infiltration of plasma cells, neutrophils, macrophages and mast cells in periodontitis tissues and demonstrated the anti‐inflammatory properties and target modulation capabilities of Coumestrol, Diosmetin and Gallicin. Our study aims to provide a comprehensive explanation of how Baiyaojian decoction exerts its immunomodulatory effect on periodontitis and lay a solid foundation for optimising its formulation. The working flow of this study is depicted in Figure 1.

## 2 Materials and Methods

### 2.1 Identify the Active Ingredients and Target Genes of Baiyaojian Decoction

The chemical ingredients of Baiyaojian decoction were identified through literature [28, 29] and searching in TCMSP database (https://old.tcmsp‐e.com/index.php) and ETCM database (http://www.tcmip.cn/ETCM2/front/#/) with the keywords ‘Baiyaojian’, ‘Pugongying’ and ‘Fangfeng’. Subsequently, the sdf files of these ingredients were downloaded from PubChem (https://pubchem.ncbi.nlm.nih.gov/) and uploaded to SwissADME (http://www.swissadme.ch/) to evaluate their drug‐likeness. Active ingredients were identified based on the criteria of having high gastrointestinal absorption and at least three ‘Yes’ of Lipinski, Ghose, Veber, Egan and Muegge [41]. Then, SwissTargetPrediction (http://www.swisstargetprediction.ch/) was used to predict the target genes of active ingredients, which were integrated as the target genes of Baiyaojian decoction.

### 2.2 Identify the Potential Therapeutic Targets of Baiyaojian Decoction in the Treatment of Periodontitis

Searches were conducted with the keyword ‘Periodontitis’ in OMIM (https://www.omim.org/), GeneCards (https://www.genecards.org/), DrugBank (https://go.drugbank.com/), CTD (https://ctdbase.org/) and MalaCards (https://www.malacards.org/) database. Periodontitis‐related pathogenic genes were identified based on the criterion of appearing in at least two databases. The intersection of the target genes of Baiyaojian decoction and periodontitis‐related pathogenic genes was selected as the potential therapeutic targets of Baiyaojian decoction in the treatment of periodontitis.

### 2.3 Enrichment Analysis

Enrichment analysis was performed using the clusterProfiler package [42] (version 4.4.4) with default parameters. The enrichment results were filtered based on the criterion of qvalue < 0.05, which were then visualised through ggplot2 package (version 3.5.1) or Sangorbox (http://sangerbox.com/home.html).

### 2.4 Construct Drug‐Ingredient‐Target‐Pathway‐Diseases Network

The associations between Baiyaojian decoction, ingredients, target genes, pathways and periodontitis were imported into Cytoscape software (version 3.10.2) to visualise the network.

### 2.5 Construct the PPI Network

All therapeutic targets were uploaded into the STRING database (https://cn.string‐db.org/) to establish the PPI network, which was filtered based on the minimum interaction score of 0.4. The original PPI network was then imported into Cytoscape software for further analysis. MCODE plugin (version 2.0.3) was used to cluster the PPI network with the default parameters. CytoNCA plugin (version 2.1.6) was used to calculate the topological properties of the PPI network, and core therapeutic targets were filtered based on their scores exceeding the median of Betweenness Centrality (BC), Closeness Centrality (CC), Degree Centrality (DC), Eigenvector Centrality (EC), Linkage Centrality (LC) and Neighbourhood Connectivity (NC) [43].

### 2.6 Analyse the Transcriptomic Microarray Data of Healthy Gingival Tissues and Periodontitis Tissues

First, the raw data of GSE16134 [44] dataset were downloaded from the GEO database (https://www.ncbi.nlm.nih.gov/geo/). The gene expression matrix was obtained after quality assessment, removing outlier samples, correcting batch effects and converting probes. Next, differential expression analysis was conducted using the limma package [45] (version 3.52.1) and differentially expressed genes were screened based on the criterion of adj.*p*.val < 0.05. Subsequently, Gene Set Enrichment Analysis (GSEA) was performed on the differential expression results using GSEA software (version 4.3.3). The CIBERSORT package [46] (version 0.1.0) was employed to analyse and compare the composition ratio of infiltrating immune cells in healthy gingival tissues and periodontitis tissues. Finally, Spearman correlation analysis was conducted to assess the relationship between the expression of core therapeutic targets and the infiltration of immune cells.

### 2.7 Analyse the Single‐Cell RNA Sequencing Data of Healthy Gingival Tissues and Periodontitis Tissues

First, raw data of GSE152042 [47] and GSE171213 [48] datasets were downloaded from the GEO database. The Seurat package [49] (version 4.2.0) and harmony package [50] (version 0.1.1) were then used for preprocessing, integration, dimension reduction and clustering. Differential expression analysis was conducted to obtain marker genes of each cell cluster by FindAllMarkers function, which were then compared with the CellMarker database (http://bio‐bigdata.hrbmu.edu.cn/CellMarker/) and relevant literature [47, 48] for cell type annotation. Subsequently, sequencing data of T cells, B cells and myeloid cells were extracted and cell subpopulations were distinguished using the same methods. The composition ratio of various immune cell types in healthy gingival tissues and periodontitis tissues was calculated and compared. The results were cross‐examined with those from the transcriptomic microarray data analysis above to identify cell types with higher composition ratio in periodontitis tissues as key cell types. The FeaturePlot function was employed to visualise the expression of core therapeutic targets within the immune cell subpopulations. The CellChat package [51] (version 2.1.2) was utilised to analyse interactions among immune cells. Finally, the monocle3 package [52] (version 1.3.1) was used to perform pseudotime analysis on key cell types, constructing differentiation trajectories along with periodontitis progression. The graph_test function was applied to extract pseudotime‐related genes.

### 2.8 Molecular Docking

UniProt database (https://www.uniprot.org/) was used to retrieve the docking receptor's protein ID and RCSB PDB database (https://www.rcsb.org/) was used to download the three‐dimensional structure of proteins. Docking ligand's two‐dimensional structure was converted into three‐dimensional structure using Chem3D software (version 21.0.0). The preprocessing of the receptor and ligand was performed using AutoDock software (version 1.5.7) to define the grid box, and AutoDock Vina software (version 1.1.2) was used to predict the binding sites, affinities and modes of the ligand–receptor complex. Finally, PyMOL software (version 3.0.4) was used to visualise the results. Docking affinity lower than −4.25 kcal/mol indicates potential binding, docking affinity lower than −5 kcal/mol indicates favourable binding and docking affinity lower than −7 kcal/mol indicates strong binding [53].

### 2.9 Patient Enrollment and Specimens

Clinical specimens were prospectively collected from the Department of Stomatology, Hospital of Chengdu University of Traditional Chinese Medicine during the period spanning January 2024 to December 2025. All tissue acquisitions were performed following written informed consent from patients.

### 2.10 qRT‐PCR

Total RNA was isolated utilising the Total RNA Extraction Kit (Sango Biotech), followed by cDNA synthesis with the First‐Strand cDNA Synthesis Kit (GeneCopoeia). qRT‐PCR was conducted using SYBR Green I master mix (TsingKe Biotechnology) with *GAPDH* (human) or *Gapdh* (murine) serving as endogenous reference genes. The primer sequences are in Table S1.

### 2.11 Cell Culture and Lipopolysaccharide (LPS) Stimulation

Murine macrophage cell line RAW 264.7 (Sunncel) was cultured in DMEM supplemented with 10% fetal bovine serum (Gibco). Cells were maintained at 37°C in a humidified 5% CO_2_ atmosphere. For inflammatory stimulation, cells were exposed to 1 μg/mL LPS (Solarbio) for 24 h.

### 2.12 Treatment With Coumestrol, Diosmetin and Gallicin

All of the compounds were purchased from MedChemExpress. Cells were exposed to these compounds for 12 h and cell viability was subsequently quantified using the CCK‐8 assay to identify the optimal concentrations for downstream experiments.

### 2.13 CCK‐8 Assay

Cells were seeded in 96‐well plates at 5000 cells/well and cultured for 24 h under standard conditions. Following the addition of 10 μL CCK‐8 solution per well, plates were incubated at 37°C for 2 h. Absorbance was measured at 450 nm using a microplate reader (Thermo Fisher).

### 2.14 NO Detection

NO levels in cell culture supernatants were quantified using the NO Detection Kit (Beyotime) according to the manufacturer's protocol, with absorbance measured at 540 nm via a microplate reader (Thermo Fisher).

### 2.15 ROS Detection

Intracellular ROS levels were quantified using the ROS Detection Kit (Beyotime) per manufacturer's instructions. Fluorescence intensity was measured by flow cytometry with excitation/emission wavelengths set at 488 nm/525 nm. Data analysis was performed using FlowJo software.

### 2.16 ELISA

The production of pro‐inflammatory cytokines IL‐1β, IL‐6 and TNF‐α was quantified using an ELISA kit (Proteintech) according to manufacturers' protocols. Absorbance measurements at 450 nm were acquired using a microplate reader (Thermo Fisher). Analyte concentrations were determined through regression of standard curves.

### 2.17 Statistical Analysis

All statistical analyses were executed in RStudio (R version 4.4.1). Continuous variables were reported as mean ± standard deviation. Categorical variable comparisons employed either Student's *t*‐test or Pearson's Chi‐squared test. Bivariate associations were assessed through linear regression modelling or Spearman's rank‐order correlation. Statistical significance was defined as *p* < 0.05.

## 3 Results

### 3.1 Identification of the Therapeutic Targets and Pathways of Baiyaojian Decoction in the Treatment of Periodontitis

**FIGURE 2:**
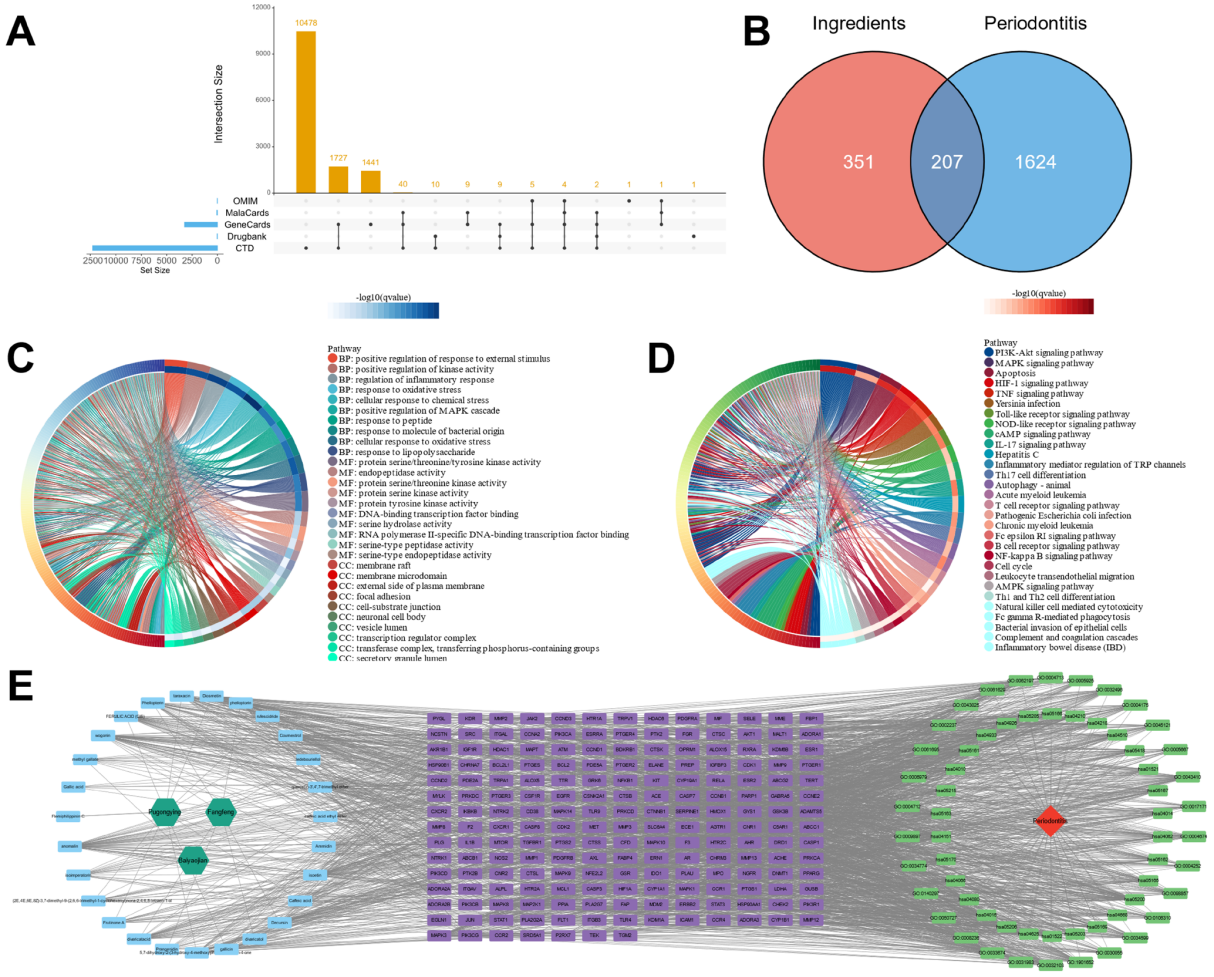
Construction of drug‐ingredient‐target‐pathway‐diseases network. (A) Upset diagram of periodontitis‐related pathogenic genes from OMIM, GeneCards, DrugBank, CTD and MalaCards databases; (B) Venn diagram of the intersection between active ingredients' target genes and periodontitis‐related pathogenic genes; (C) GO enrichment analysis results of the therapeutic targets (showing only the top 30 entries); (D) KEGG enrichment analysis results of the therapeutic targets (showing only the top 30 entries); (E) Drug‐ingredient‐target‐pathway‐diseases network (showing only the top 30 pathway‐related network).

The chemical ingredients of Baiyaojian decoction were identified through literature [28, 29] and TCMSP and ETCM databases, which were then evaluated by SwissADME based on their drug‐likeness and 27 active ingredients were obtained (Table 1). Then, SwissTargetPrediction was employed to predict the target genes of these active ingredients, yielding a total of 558 target genes (Table S2). Subsequently, periodontitis‐related pathogenic genes were obtained from OMIM, GeneCards, DrugBank, CTD and MalaCards databases and 1831 pathogenic genes were identified after applying a selection criterion of at least two appearances across all databases (Figure 2A, Table S3). The intersection of active ingredients' target genes and periodontitis‐related pathogenic genes yielded 207 potential therapeutic targets of Baiyaojian decoction in the treatment of periodontitis (Figure 2B, Table S4). GO enrichment analysis of these therapeutic targets revealed that Baiyaojian decoction may exert its effects by regulating the periodontal tissue's response to external stimuli, inflammatory response, oxidative stress process, protein kinase activity, DNA transcription and intercellular adhesion (Figure 2C). KEGG enrichment analysis indicated that the therapeutic targets were primarily enriched in signalling pathways related to inflammatory response, including PI3K‐AKT, MAPK and HIF‐1 signalling pathways, as well as pathways associated with the differentiation and maturation of immune cells, such as T cells, B cells and myeloid cells (Figure 2D). Finally, the drug‐ingredient‐target‐pathway‐diseases network was constructed (Figure 2E). In summary, Baiyaojian decoction may treat periodontitis through a multi‐target and multi‐pathway approach, particularly by regulating immune cells activities and inflammatory response.

**TABLE 1:** Active ingredients of Baiyaojian decoction.

Molecule name	Druglikeness					GI absorption
	Lipinski	Ghose	Veber	Egan	Muegge	
(2E,4E,6E,8Z)‐3,7‐dimethyl‐9‐(2,6,6‐trimethyl‐1‐cyclohexenyl)nona‐2,4,6,8‐tetraen‐1‐ol	Yes	Yes	Yes	Yes	No	High
5,7‐dihydroxy‐2‐(3‐hydroxy‐4‐methoxyphenyl)chroman‐4‐one	Yes	Yes	Yes	Yes	Yes	High
Ammidin	Yes	Yes	Yes	Yes	Yes	High
Anomalin	Yes	Yes	Yes	Yes	Yes	High
Caffeic acid ethyl ester	Yes	Yes	Yes	Yes	Yes	High
Caffeic acid	Yes	Yes	Yes	Yes	No	High
Coumestrol	Yes	Yes	Yes	Yes	Yes	High
Decursin	Yes	Yes	Yes	Yes	Yes	High
Diosmetin	Yes	Yes	Yes	Yes	Yes	High
Divaricatacid	Yes	Yes	Yes	Yes	Yes	High
Divaricatol	Yes	Yes	Yes	Yes	Yes	High
Ferulic acid (cis)	Yes	Yes	Yes	Yes	No	High
Flemiphilippinin C	Yes	Yes	Yes	Yes	No	High
Frutinone A	Yes	Yes	Yes	Yes	Yes	High
Gallic acid	Yes	No	Yes	Yes	No	High
Gallicin	Yes	Yes	Yes	Yes	Yes	High
Isoetin	Yes	Yes	Yes	Yes	Yes	High
Isoimperatorin	Yes	Yes	Yes	Yes	Yes	High
Ledebouriellol	Yes	Yes	Yes	Yes	Yes	High
Methyl gallate	Yes	Yes	Yes	Yes	No	High
Phellopterin	Yes	Yes	Yes	Yes	Yes	High
Phelloptorin	Yes	Yes	Yes	Yes	Yes	High
Prangenidin	Yes	Yes	Yes	Yes	Yes	High
Quercetin‐3′,4′,7‐trimethyl ether	Yes	Yes	Yes	Yes	Yes	High
Rufescidride	Yes	Yes	Yes	Yes	Yes	High
Taraxacin	Yes	Yes	Yes	Yes	Yes	High
Wogonin	Yes	Yes	Yes	Yes	Yes	High

### 3.2 The PPI Network of Therapeutic Targets

**FIGURE 3:**
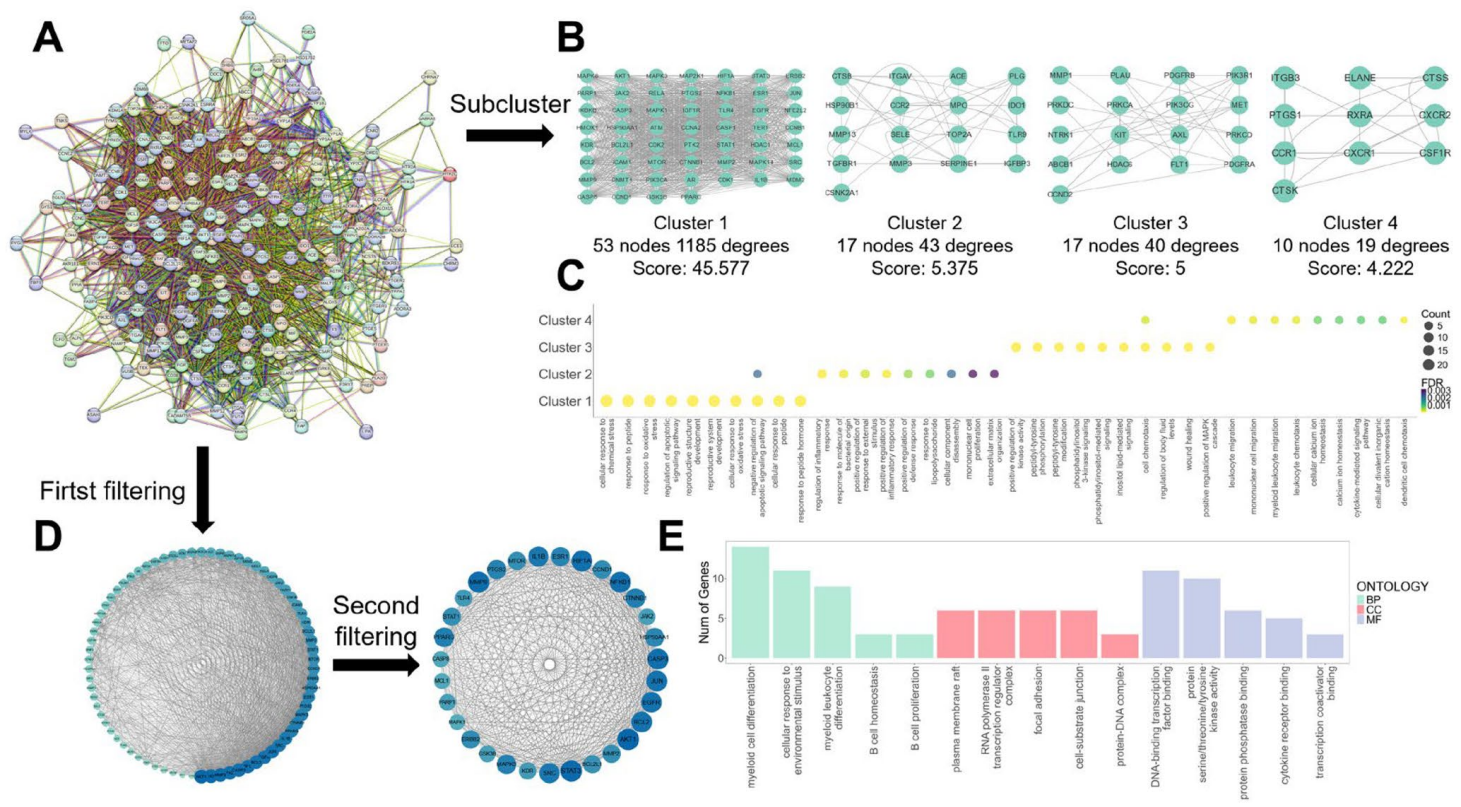
The PPI network and core therapeutic targets. (A) The PPI network diagram of therapeutic targets; (B) Clustering results of the PPI network; (C) GO enrichment analysis results of the four clusters; (D) Core therapeutic targets identified by double filtering; (E) GO enrichment analysis results of the core therapeutic targets.

To further understand the intrinsic relationships among the therapeutic targets, STRING platform was used to construct the PPI network, which consisted of 207 nodes and 3876 edges (Figure 3A). The PPI network was then imported into Cytoscape software for further analysis. MCODE plugin was utilised to perform clustering on the PPI network, resulting in four clusters of closely related therapeutic targets (Figure 3B). GO enrichment analysis was conducted for each cluster, revealing that Cluster 1 was primarily responsible for responding to external stimuli, Cluster 2 mainly promoted inflammatory response, Cluster 3 primarily regulated protein kinase activity and Cluster 4 was mainly involved in the chemotaxis of immune cells (Figure 3C). Furthermore, CytoNCA plugin was employed to compute the topological properties of the PPI network. All nodes were filtered twice based on the median values of BC, CC, DC, EC, LC and NC, ultimately yielding 31 core therapeutic targets (Figure 3D). These core therapeutic targets were primarily enriched in immune cells proliferation, infiltration and activation, protein kinase activity, DNA transcription, signal pathway activation and intercellular adhesion (Figure 3E), which was consistent with the GO enrichment results of all the therapeutic targets mentioned above, suggesting that these core therapeutic targets were representative of all therapeutic targets.

### 3.3 Core Therapeutic Targets Were Involved in Immune Cells Infiltration in Periodontitis

**FIGURE 4:**
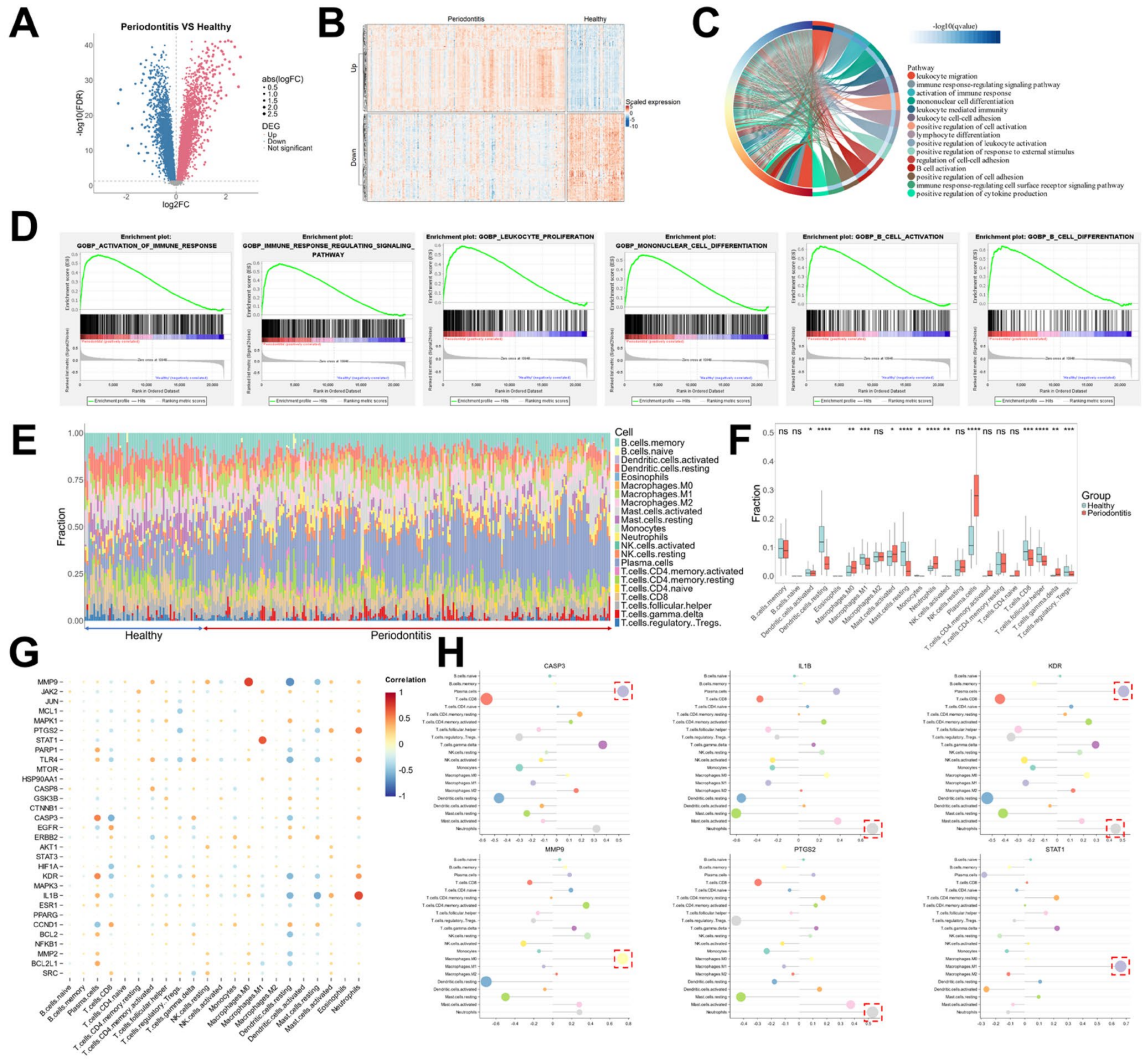
Correlation between core therapeutic targets expression and immune cells infiltration. (A) Volcano plot of differentially expressed genes between normal group and periodontitis group; (B) Heatmap of gene expression in normal group and periodontitis group; (C) GO enrichment analysis results of overexpressed genes in periodontitis group; (D) GSEA analysis results between normal group and periodontitis group (periodontitis versus normal); (E) The composition ratio of various immune cell types in normal group and periodontitis group; (F) Differences of the composition of immune cells between normal group and periodontitis group; (G) The correlation between core therapeutic targets expression and immune cells infiltration; (H) Correlation of the expression of CASP3, IL1B, KDR, MMP9, PTGS2 and STAT1 with immune cells infiltration (*p < 0.05, **p < 0.01, ***p < 0.001, ****p < 0.0001, ^ns^ *p* > 0.05).

The above results indicated that the therapeutic targets were closely related to the immune cells' activities and inflammatory response. To further investigate whether Baiyaojian decoction could relieve immune dysregulation in periodontitis by regulating immune cells' activities, the GSE16134 dataset was included. After preprocessing, removing outlier samples and correcting for batch effects (Figure S1A), the gene expression profiles of 68 normal gingival tissues and 235 periodontitis tissues were obtained. Gene differential expression analysis revealed 6077 upregulated genes (log2FC > 0, FDR < 0.05) and 6470 downregulated genes (log2FC < 0, FDR < 0.05) in the periodontitis group (Figure 4A,B). The upregulated genes were significantly enriched in immune response and immune regulatory signalling pathways (Figure 4C), which was consistent with the GO enrichment analysis results of the core therapeutic targets. GSEA analysis further yielded similar findings (Figure 4D). To understand the regulatory roles of core therapeutic targets in immune cell infiltration, we utilised the CIBERSORT algorithm to calculate the composition ratios of immune cells in the normal group and the periodontitis group (Figure 4E). The results showed a significant increase in the proportion of plasma cells, macrophages, mast cells and neutrophils in the periodontitis group (Figure 4F). Further analysis of the correlation between core therapeutic targets' expression and immune cell infiltration indicated that most core therapeutic targets were related to immune cell infiltration (Figure 4G). Notably, the expression of CASP3, IL1B, KDR, MMP9, PTGS2 and STAT1 was significantly correlated with the infiltration of plasma cells, neutrophils or macrophages (Figure 4H). These results suggest that core therapeutic targets are closely associated with immune cell infiltration in periodontitis, further implying that Baiyaojian decoction may treat periodontitis by modulating immune cell infiltration.

### 3.4 Single‐Cell RNA Sequencing Reveals the Critical Role of Core Therapeutic Targets in Immune Dysregulation

**FIGURE 5:**
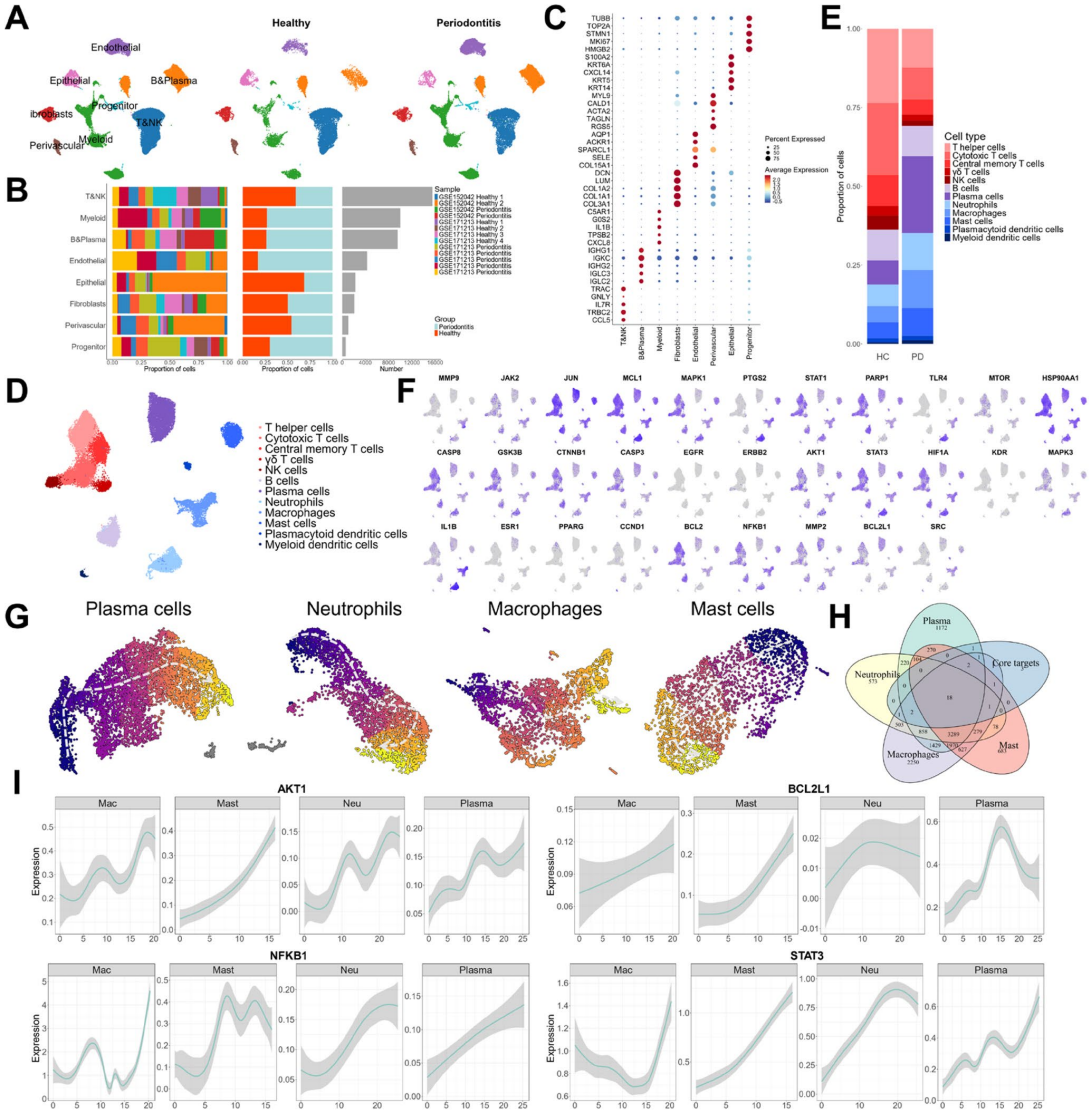
Single‐cell RNA sequencing explored the roles of core therapeutic targets in promoting periodontitis progression. (A) UMAP plots of cell type annotation; (B) Composition and quantitative statistics of cell origin and number; (C) Bubble plot of the expression of cell marker genes in various cell types; (D) UMAP plot of immune cells; (E) Composition of immune cells in normal tissues and periodontitis tissues; (F) UMAP plots of the expression of core therapeutic targets in immune cells; (G) UMAP plots of pseudotime trajectories of plasma cells, neutrophils, macrophages and mast cells; (H) Venn diagram showing the intersection of pseudotime‐related genes of plasma cells, neutrophils, macrophages and mast cells and core therapeutic targets; (I) Expression of a part of core therapeutic targets along the differentiation trajectories of plasma cells, neutrophils, macrophages and mast cells.

To further investigate the status of immune dysregulation in periodontitis tissues and the role of core therapeutic targets in immune cells activities, GSE152042 and GSE171213 datasets were included, which comprised single‐cell RNA sequencing data of 6 normal gingival tissues and 7 periodontitis tissues. After preprocessing, integrating, dimension reduction and clustering, 19 cell clusters were identified (Figure S2A,B). The marker genes of each cell cluster were compared with relevant databases and literature [47, 48] to annotate cell types, which ultimately classified all cell clusters into 8 cell types (Figure 5A–C), including T & NK cells (CCL5, TRBC2, IL7R), B & plasma cells (IGLC2, IGLC3, IGHG2), myeloid cells (CXCL8, TPSB2, IL1B), fibroblasts (COL3A1, COL1A1, COL1A2), endothelial cells (COL15A1, SELE, SPARCL1), perivascular cells (RGS5, TAGLN, ACTA2), epithelial cells (KRT14, KRT5, CXCL14) and progenitor cells (HMGB2, MKI67, STMN1). All immune cells were then classified into 12 subpopulations (Figure 5D). And the increase of plasma cells, neutrophils, macrophages and mast cells was observed in periodontitis tissues (Figure 5E), which aligned with the above results. Most of the core therapeutic targets were also prominently expressed in various immune cell subpopulations (Figure 5F), further demonstrating the important roles of core therapeutic targets in immune cells' activities. Cell–cell communication analysis among immune cells indicated that the number of interactions among immune cells in periodontitis tissues was higher than that in normal tissues (Figure S3A). Considering the enhanced infiltration of plasma cells, neutrophils, macrophages and mast cells in periodontitis tissues, these cell types were identified as key immune cells. Pseudotime analysis was conducted to construct their differentiation trajectories along with periodontitis progression and extract pseudotime‐related genes (Figure 5G, Figure S3B). These genes play a crucial role in the transition of key immune cells from a resting state to a pro‐inflammatory state, thus representing key genes contributing to immune dysregulation in periodontitis. By intersecting core therapeutic targets with pseudotime‐related genes, we found that out of the 31 core therapeutic targets, 28 belonged to the pseudotime‐related genes and 18 of them were associated with the differentiation of all four key cell types (Figure 5H,I, Figure S3C). These results indicate that core therapeutic targets are critical regulatory genes in key immune cells differentiation during periodontitis progression, promoting their transition from a resting state to a pro‐inflammatory state. This further suggests that Baiyaojian decoction may restore immune homeostasis in periodontitis tissues through its immunomodulatory properties.

### 3.5 Molecular Docking Verification

**FIGURE 6:**
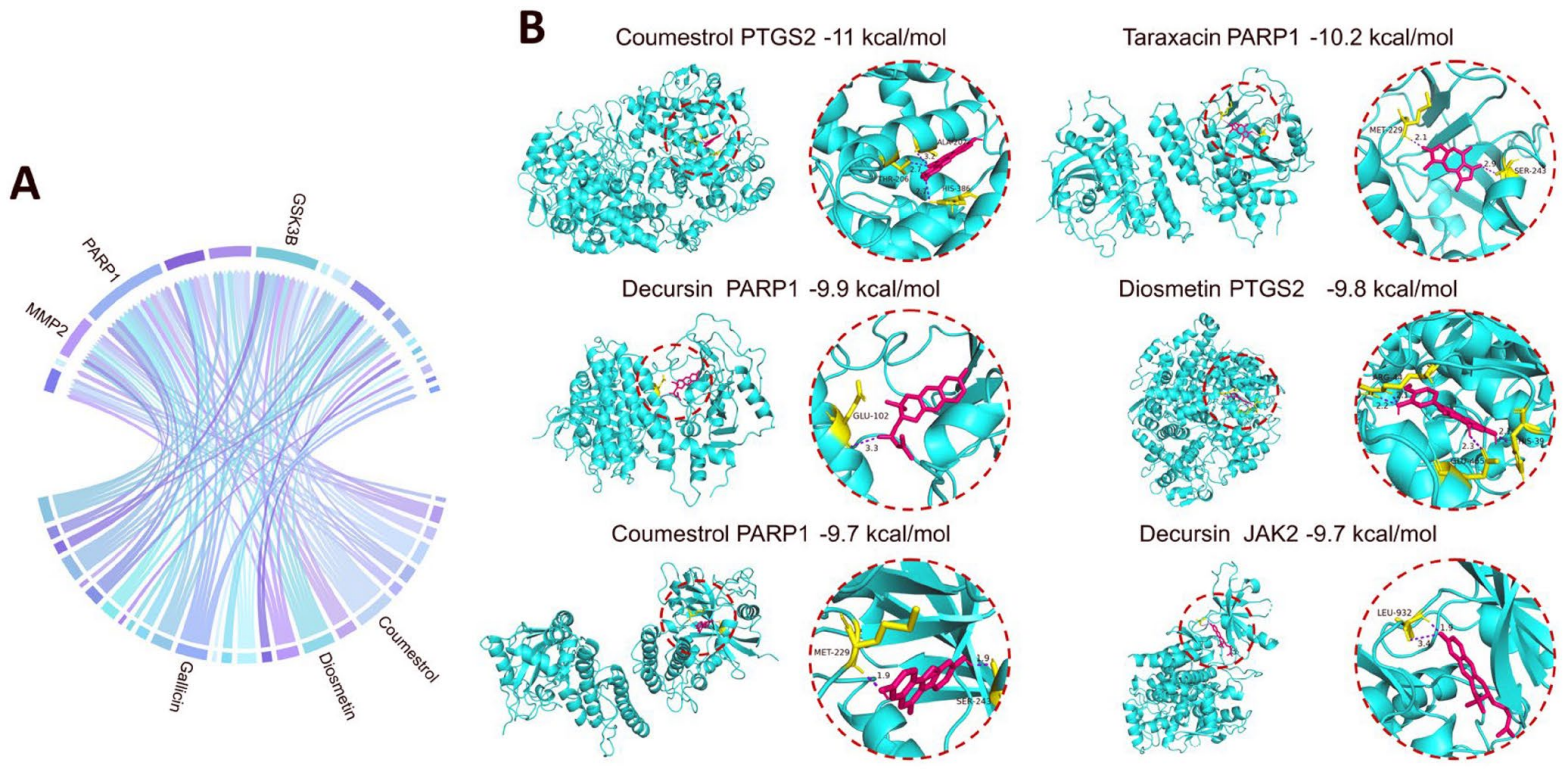
Molecular docking. (A) The chord diagram of 82 pairs of binding relationships. (B) Molecular docking of top 6 ingredient‐target pairs (left panel) and their binding sites and binding affinities (right panel).

To further investigate the immunomodulatory effect of Baiyaojian decoction on periodontitis, molecular docking was performed to investigate the binding capacity between active ingredients and therapeutic targets. Based on the above results, 18 core therapeutic targets closely related to the differentiation of key immune cells were identified as docking receptors and 26 active ingredients capable of targeting these proteins were identified as docking ligands, resulting in a total of 82 pairs of binding relationships (Figure 6A). Eighty pairs exhibited favourable docking affinity lower than −5 kcal/mol, among which 47 pairs exhibited strong docking affinity lower than −7 kcal/mol (Table 2). Among all the ingredients, Coumestrol, Diosmetin and Gallicin exhibited the highest number of stable bindings with therapeutic targets, making them the key active ingredients. Among all the therapeutic targets, PARP1, GSK3B and MMP2 could be targeted by the majority of active ingredients, making them the key therapeutic targets. The top 6 ingredient‐target pairs (Figure 6B) were Coumestrol‐PTGS2 (−11 kcal/mol), Taraxacin‐PARP1 (−10.2 kcal/mol), Decursin‐PARP1 (−9.9 kcal/mol), Diosmetin‐PTGS2 (−9.8 kcal/mol), Coumestrol‐PARP1 (−9.7 kcal/mol) and Decursin‐JAK2 (−9.7 kcal/mol). The molecular docking results further demonstrate the immunomodulatory role of Baiyaojian decoction in the treatment of periodontitis at the molecular level.

**TABLE 2:** Molecular docking results.

Target proteins	Active ingredients	Affinity (kcal/mol)
PTGS2	Coumestrol	−11
PARP1	Taraxacin	−10.2
PARP1	Decursin	−9.9
PTGS2	Diosmetin	−9.8
PARP1	Coumestrol	−9.7
JAK2	Decursin	−9.7
PARP1	5,7‐Dihydroxy‐2‐(3‐hydroxy‐4‐methoxyphenyl)chroman‐4‐one	−9.6
PARP1	Isoetin	−9.5
PARP1	Diosmetin	−9.4
GSK3B	Rufescidride	−9.4
PARP1	Divaricatacid	−9
PTGS2	Ammidin	−8.9
GSK3B	Coumestrol	−8.9
PARP1	Isoimperatorin	−8.9
PARP1	Quercetin‐3′,4′,7‐trimethyl ether	−8.9
PTGS2	Wogonin	−8.9
PARP1	Gallicin	−8.8
GSK3B	Decursin	−8.7
JAK2	Ammidin	−8.6
JAK2	Isoimperatorin	−8.6
PARP1	Phellopterin	−8.6
MAPK1	Anomalin	−8.5
GSK3B	Divaricatacid	−8.5
PARP1	Phelloptorin	−8.5
GSK3B	Quercetin‐3′,4′,7‐trimethyl ether	−8.5
GSK3B	Diosmetin	−8.4
GSK3B	Isoetin	−8.4
BCL2	5,7‐Dihydroxy‐2‐(3‐hydroxy‐4‐methoxyphenyl)chroman‐4‐one	−8.3
PTGS2	Anomalin	−8.3
HSP90AA1	Coumestrol	−8.2
JUN	Gallicin	−8.2
JAK2	Phellopterin	−8.2
JAK2	Phelloptorin	−8.2
HSP90AA1	Frutinone A	−8
GSK3B	Wogonin	−8
GSK3B	Anomalin	−7.9
HSP90AA1	Wogonin	−7.9
PTGS2	Gallicin	−7.8
GSK3B	Gallicin	−7.7
HSP90AA1	Ledebouriellol	−7.7
MAPK1	Divaricatacid	−7.5
HSP90AA1	Divaricatol	−7.5
JAK2	Anomalin	−7.4
HSP90AA1	Phellopterin	−7.4
JAK2	Gallicin	−7.2
HSP90AA1	Phelloptorin	−7.2
HIF1A	Divaricatol	−7.1
CASP3	Divaricatacid	−7
MCL1	Diosmetin	−6.8
PARP1	Caffeic acid ethyl ester	−6.7
PARP1	Ferulic acid (Cis)	−6.7
NFKB1	Frutinone A	−6.7
MCL1	Quercetin‐3′,4′,7‐trimethyl ether	−6.7
MCL1	Taraxacin	−6.7
AKT1	Coumestrol	−6.6
MMP2	Rufescidride	−6.6
MCL1	Wogonin	−6.6
MAPK1	(2e,4e,6e,8z)‐3,7‐Dimethyl‐9‐(2,6,6‐trimethyl‐1‐cyclohexenyl)nona‐2,4,6,8‐tetraen‐1‐Ol	−6.4
NFKB1	Coumestrol	−6.4
AKT1	Diosmetin	−6.4
AKT1	Quercetin‐3′,4′,7‐trimethyl ether	−6.3
PTGS2	Ferulic acid (Cis)	−6.2
IL1B	Gallicin	−6.2
AKT1	Isoetin	−6.2
MMP2	Prangenidin	−6.1
GSK3B	Caffeic acid ethyl ester	−6
MMP2	Coumestrol	−5.9
MMP2	Isoetin	−5.9
MAPK1	Caffeic acid ethyl ester	−5.8
BCL2L1	Gallic acid	−5.8
BCL2L1	Methyl gallate	−5.8
MMP2	Quercetin‐3′,4′,7‐trimethyl ether	−5.8
MMP2	5,7‐Dihydroxy‐2‐(3‐hydroxy‐4‐methoxyphenyl)chroman‐4‐one	−5.7
MAPK1	Caffeic acid	−5.7
MMP2	Wogonin	−5.7
STAT3	Caffeic acid	−5.6
CTNNB1	Ferulic acid (cis)	−5.5
MMP2	Diosmetin	−5.4
STAT3	Ferulic acid (cis)	−5.1
MMP2	Caffeic acid ethyl ester	−5
MMP2	Caffeic acid	−4.6
MMP2	Ferulic acid (cis)	−4.6

### 3.6 Experimental Validation of Immune Cell Infiltration and Therapeutic Efficacy of Bioactive Ingredients

**FIGURE 7:**
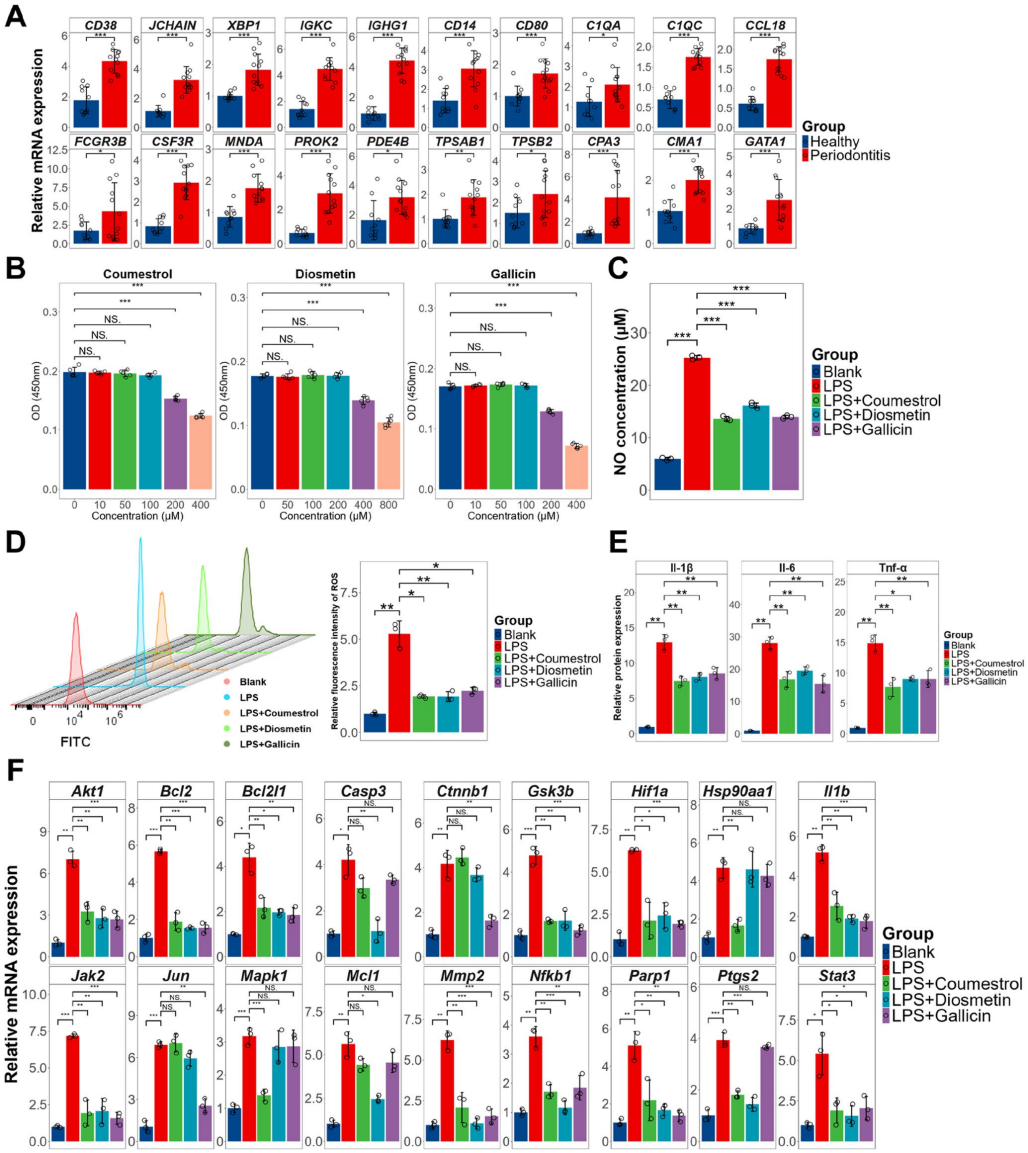
Experimental validation. (A) Differential expression of cell‐type–specific marker genes between healthy periodontal tissues and periodontitis tissues; (B) Identification of optimal non‐cytotoxic concentrations for active ingredients via CCK‐8 assays; (C) Comparative analysis of NO secretion among control, LPS‐stimulated group and LPS‐stimulated group co‐treated with active ingredients; (D) Differences in ROS production (left panel) with quantitative comparison (right panel) across control, LPS‐stimulated group and LPS‐stimulated group co‐treated with active ingredients; (E) Differential secretion of pro‐inflammatory cytokines among control, LPS‐stimulated group and LPS‐stimulated group co‐treated with active ingredients; (F) Expression of core therapeutic targets across control, LPS‐stimulated group and LPS‐stimulated group co‐treated with active ingredients (*p < 0.05, **p < 0.01, ***p < 0.001, ^NS^ *p* > 0.05).

To verify enhanced infiltration of plasma cells, macrophages, mast cells and neutrophils in periodontitis tissues, we collected 10 healthy periodontal specimens and 12 periodontitis specimens. Using cell‐type‐specific marker genes identified from sc‐RNA seq, qRT‐PCR revealed significantly elevated expression of all cell‐type marker genes in periodontitis tissues (Figure 7A), indicating substantially augmented infiltration of these immune cells. Subsequently, we established the in vitro periodontitis model by stimulating murine macrophage cell line RAW 264.7 with LPS to evaluate the anti‐inflammatory effects and target modulation capabilities of the top‐ranked bioactive ingredients, including Coumestrol, Diosmetin and Gallicin. CCK‐8 assay determined the optimal non‐cytotoxic concentrations of these ingredients (Figure 7B). All three compounds significantly suppressed the NO production and ROS generation in inflamed macrophages (Figure 7C,D). ELISA further confirmed their potent inhibition of pro‐inflammatory cytokine expression, including TNF‐α, IL‐6 and IL‐1β (Figure 7E). While LPS markedly upregulated the expression of 18 core therapeutic targets in macrophages, Coumestrol suppressed 14 of these targets, Diosmetin inhibited 14 targets and Gallicin downregulated 13 targets (Figure 7F). Collectively, these results demonstrate Coumestrol, Diosmetin and Gallicin possess significant anti‐inflammatory and therapeutic target‐modulating potential for periodontitis treatment.

## 4 Discussion

Current treatment strategies for periodontitis primarily focus on mechanical clearance of bacterial biofilm and antimicrobial therapies, while neglecting the critical role of immune response in periodontitis [14]. This oversight may result in insufficient control of inflammation, leading to prolonged chronic inflammation and a high incidence of recurrence after treatment [54]. Thus, exploring novel therapeutic strategies with immunomodulatory effects may help to effectively manage inflammation in periodontitis. Recently, TCM has emerged as a promising approach with immunomodulatory effects across various diseases [22, 55]. Baiyaojian decoction consists of Baiyaojian, Pugongying and Fangfeng, all of which have demonstrated immunomodulatory properties in various inflammation‐related disorders [29, 31, 40], making Baiyaojian decoction an innovative therapy for managing periodontitis. In this study, we employed network pharmacology to elucidate the active ingredients and therapeutic targets of Baiyaojian decoction for treating periodontitis. Utilising single‐cell RNA sequencing, we identified immune cells closely associated with periodontitis progression and demonstrated the crucial roles of therapeutic targets in the infiltration, differentiation and maturation of these immune cells. Additionally, molecular docking confirmed the binding between active ingredients and therapeutic targets, underscoring the immunomodulatory and anti‐inflammatory properties of Baiyaojian decoction in periodontitis. Experimental validation further confirmed the elevated infiltration of plasma cells, neutrophils, macrophages and mast cells in periodontitis tissues and demonstrated the anti‐inflammatory properties and target modulation capabilities of key ingredients including Coumestrol, Diosmetin and Gallicin.

Network pharmacology is limited to a general prediction of therapeutic targets and signalling pathways, unable to identify the specific cell types targeted by drugs [56]. However, periodontitis is a disease involving multiple cell types and complex cell–cell interactions [13]. Throughout periodontitis progression, different cell populations exhibit markedly distinct gene expression patterns and signalling pathway activities, resulting in diverse responses to medications [57]. Consequently, when exploring the therapeutic mechanisms for periodontitis, we should not overlook cellular heterogeneity. Instead, a more detailed analysis of the regulatory effects of active ingredients on different cell types is of great significance [1]. Single‐cell RNA sequencing allows for comprehensive gene expression profiling of all cells [58], which enables the identification of immune cells closely associated with periodontitis [59], providing new insights for understanding the complex biological mechanisms and identifying novel therapeutic targets [60]. Based on network pharmacology, single‐cell RNA sequencing can be utilised to further investigate the expression profiles of therapeutic targets across various immune cell subpopulations [61] and elucidate how they influence immune cells' activities to contribute to immune dysregulation in periodontitis [62]. This combined approach can also elucidate how Baiyaojian decoction may alleviate periodontitis by modulating specific cellular populations. In our study, we first identified the potential therapeutic targets based on network pharmacology. Subsequently, we performed single‐cell RNA sequencing analysis, identifying key immune cells with enhanced infiltration in periodontitis tissues and detecting the expression of therapeutic targets in these cells. We then conducted cell–cell interaction analysis to investigate the regulatory effects of these cells on other cells within the inflammatory environment. Finally, we established differentiation trajectories of these cells along periodontitis progression to identify genes regulating the transition from a resting state to a pro‐inflammatory state and further screened out 18 key therapeutic targets. By integrating the comprehensiveness of network pharmacology with the precision of single‐cell RNA sequencing, our study provides a multidimensional analysis of the therapeutic targets of Baiyaojian decoction, revealing its immunomodulatory properties in the treatment of periodontitis.

PARP1 plays a critical role in periodontitis progression by inducing periodontal tissues damage [63]. PARP1 is also significantly associated with the progression of periodontitis in patients with rheumatoid arthritis, increasing cardiovascular disease risk by enhancing inflammatory biomarker levels [64]. Mechanically, PARP1 is involved in cellular stress response and the inhibition of proliferation and ossification of periodontal ligament stem cells (PDLSCs) [65]. In addition, PARP1 has been revealed to interact with the long non‐coding RNA AC018926.2, enhancing ITGA2 expression, activating the ITGA2/FAK/AKT signalling pathway and ultimately inhibiting the osteogenic differentiation of PDLSCs [66]. GSK3B mediates IL‐17–induced phosphorylation of transcription factor C/EBPβ, reducing Del‐1 expression and contributing to periodontal bone loss [67]. Inhibition of GSK3B by lithium chloride enhances bone restoration by activating Wnt/β‐catenin signalling pathway [68]. By targeting the PI3K‐AKT‐GSK3B pathway, Curcumin exhibits anti‐inflammatory effects to enhance insulin sensitivity and mitigate the progression of diabetic periodontitis [69]. MMP2, a well‐known member of the matrix metalloproteinase family, has been extensively studied in periodontitis as an important effector in periodontal tissues destruction by degrading extracellular matrix and basement membrane components [13]. Targeting MMP2 through Tetracycline, Doxycycline [70], Curcumin [71] and other MMP inhibitors [72] can offer therapeutic benefits in regenerative strategies for periodontitis tissues. In our study, we first identified the therapeutic targets of Baiyaojian decoction in the treatment of periodontitis through network pharmacology. Then we identified the core therapeutic targets through PPI network analysis. After confirming the association of these therapeutic targets with immune cell infiltration through immune cell infiltration analysis, single‐cell RNA sequencing analysis was performed to identify key therapeutic targets involved in immune dysregulation. Finally, molecular docking was performed to elucidate the binding between active ingredients and key therapeutic targets, revealing PARP1, GSK3B and MMP2 exhibited broad interactions with active ingredients. Our results further highlight that PARP1, GSK3B and MMP2 may serve as key therapeutic targets of Baiyaojian decoction in the treatment of periodontitis.

Coumestrol, a phytoestrogen present in many Chinese herbs, has been reported to effectively reduce inflammation in diabetic retinopathy (DR) by upregulating SIRT1 expression [73]. In osteoarthritis, Coumestrol prevents the loss of proteoglycans caused by IL‐1β and effectively suppresses the expression of MMP‐1, MMP‐3, MMP‐13 [74]. In non‐alcoholic fatty liver disease, Coumestrol promotes triacylglycerol deposition over diacylglycerol and significantly reduces the expression of IL‐6, TNF‐α, TGF‐β and NF‐κβ [75]. Besides, Coumestrol has also proved to be effective in autoimmune disease. Low‐dose Coumestrol significantly decreases serum anti‐thyroglobulin IgG titers levels, the percentage of Th1 cells and IFN‐γ mRNA expression [76], indicating the immunomodulatory role of Coumestrol. Diosmetin, a natural flavonoid, exhibits significant anti‐inflammatory effects by disturbing the crosstalk between macrophages and adipocytes. Mechanically, it reduces the secretion of pro‐inflammatory mediators by downregulating inducible NO (iNOS) synthase and inhibiting the activation of MAPK/NF‐κB signalling pathway [77]. Furthermore, Diosmetin reduces mast cells infiltration through suppressing TNF‐α, IL‐4, IL‐1β and iNOS production via inhibiting MAPK and JAK/STAT signalling pathways [78]. Gallicin, a polyphenol found in various plants, can exert anti‐inflammatory properties by inhibiting inflammatory cytokines expression and signalling pathways activation. Gallicin inhibits the production of IL‐6 and IL‐8 in oral epithelial cells induced by *Fusobacterium nucleatum* in a dose‐dependent manner [79]. In addition, Gallicin can also suppress the growth of *F. nucleatum* [79], *Porphyromonas gingivalis* [80] and *Streptococcus* [81], which are the key pathogens in periodontitis. Gallicin effectively suppresses NF‐κB activity and prevents the phosphorylation of proteins in MAPK pathways, including ERK1/2, p38 and JNK [82]. Gallicin prevents the oligomerization of NLRP3 inflammasomes through the attenuation of ROS over‐generation to manage the inflammatory response [83]. The oral administration of Gallicin nanomicelles results in a notable reduction in total leukocytes, neutrophils and mononuclear cells at the inflammation site [84]. In our study, we confirmed the key ingredients through ingredient‐target network and molecular docking and found that Coumestrol, Diosmetin and Gallicin could extensively bind to key therapeutic targets. Our study further suggests the significant therapeutic value of Coumestrol, Diosmetin and Gallicin in treating periodontitis and provides a theoretical foundation for the extraction of active ingredients from Baiyaojian decoction and the optimization of its formulation.

Histological and transcriptomic investigations have substantiated that plasma cells infiltration constitutes a critical defence mechanism against periodontal microbial communities. Within periodontitis lesions, plasma cells propagate inflammatory cascades via antibody production. This dualistic response potentially mediates microbial clearance while simultaneously exacerbating tissue destruction through hyperactive immunological reactions [85]. Elevated infiltration of plasma cells correlates positively with histological inflammation severity in periodontitis lesions [44]. Pathological bone resorption ensues when dysregulation occurs within the triad of microbial invasion, immune responses and tissue regeneration, a process mediated by plasma cells activities [86]. Macrophages play a dualistic role in periodontitis pathogenesis, dynamically shifting between destructive and reparative states through phenotypic polarisation. Pro‐inflammatory M1 macrophages dominate the disease progression phase, where they recognise periodontal pathogens via TLR2/4 and secrete IL‐6, TNF‐α and IL‐12, directly amplifying inflammation and activating osteoclastogenesis through RANKL‐dependent pathways [87]. Conversely, reparative M2 macrophages prevail during tissue healing, secreting IL‐10 and TGF‐β to suppress neutrophil infiltration, resolve inflammation and promote collagen deposition [88]. The imbalance of M1/M2 ratio critically dictates clinical outcomes. Elevated M1/M2 ratios in gingiva correlate with increased probing depth and bone loss, while experimental M2 adoptive transfer reduces bone resorption in murine periodontitis models [89]. Neutrophils serve as the primary defender against bacterial invasion while simultaneously driving periodontal tissues destruction through dysregulated immune responses [90]. Upon bacterial challenge, neutrophils are recruited to periodontal pockets via chemokine gradients and adhesion molecules, where they deploy antimicrobial mechanisms including phagocytosis, degranulation of proteolytic enzymes and neutrophil extracellular trap formation. However, in periodontitis, these protective functions become pathological. Hyperactivation due to LPS stimulation exacerbates neutrophil extracellular trap release, causing damage to gingival epithelium and periodontal ligament collagen via histone cytotoxicity and MMP‐8‐mediated degradation of type I collagen [91]. Critically, neutrophil extracellular traps act as a double‐edged sword, entrapping pathogens and triggering osteoclastogenesis via RANKL signalling pathway at the same time [92]. Mast cells play a pivotal role in periodontitis pathogenesis, dynamically transitioning from immune surveillance to tissue destruction through pathogen‐induced activation. In periodontitis, mast cell infiltration and degranulation escalate with disease severity, significantly higher in severe cases than in mild or healthy cases and correlate with increased tissue fibrosis and alveolar bone loss [93]. Mast cell activation is triggered by periodontal pathogens, leading to rapid release of pro‐inflammatory cytokines including TNF‐α, IL‐6, IL‐8 and MMPs, which collectively amplify vascular permeability, neutrophil recruitment and osteoclastogenesis [94]. Notably, mast cell‐specific receptor MRGPRX2 responds to bacterial cationic peptides and sustains inflammation through NF‐κB‐dependent cytokine cascades [95]. In our study, integrated analysis of bulk RNA seq and sc‐RNA seq collectively demonstrated substantial infiltration and hyperactivation of plasma cells, neutrophils, macrophages and mast cells during periodontitis pathogenesis, with respective marker genes upregulation in periodontitis tissues validated by qRT‐PCR. Pseudotime analysis identified 18 core therapeutic targets that orchestrated pro‐inflammatory differentiation across all cell lineages, exhibiting strong co‐expression patterns along trajectories from quiescent to activated states. Functional validation further confirmed that LPS stimulation upregulated these targets' expression in macrophages and pretreatment with coumestrol, diosmetin or gallicin significantly suppressed their expression, suggesting the prominent anti‐inflammatory potential of these bioactive ingredients for treating periodontitis.

TCMs have garnered increasing scientific validation for their immunomodulatory properties, emerging as a promising therapeutic strategy across diverse inflammatory disorders [96]. Historically, TCM formulations were employed to treat ‘tooth declination’ and ‘gum atrophy’, conditions now recognised as periodontitis, though their molecular mechanisms remained unclear until modern pharmacological interrogation [24]. Within this context, Baiyaojian decoction, a tri‐herbal formula comprising Baiyaojian (*Galla Chinensis*), Pugongyin (*Taraxacum genus*) and Fangfeng (*S. divaricata*), represents a rationally designed formulation leveraging the anti‐inflammatory synergy of its constituents. Gallotannins from *Galla Chinensis* inhibit NF‐κB translocation by stabilising IκBα [97], taraxasterol in *Taraxacum genus* suppresses NLRP3 inflammasome assembly [98] and prim‐O‐glucosylcimifugin from *S. divaricata* blocks STAT3 phosphorylation [99]. In our study, network pharmacology analysis of 27 active ingredients identified 207 therapeutic targets converging on multiple signalling pathways, notably PI3K‐AKT, MAPK and HIF‐1 signalling, with a multi‐component, multi‐target and multi‐pathway topology. Integrated analysis of bulk RNA seq and sc‐RNA seq collectively demonstrated Baiyaojian decoction's capacity to modulate immune cell infiltration and activation in periodontitis tissues. Experimental validation confirmed that key ingredients, Coumestrol, Diosmetin and Gallicin, synergistically inhibited LPS‐induced NO synthesis, ROS production, pro‐inflammatory cytokine secretion and therapeutic target upregulation in macrophages. Collectively, these findings position Baiyaojian decoction as a mechanistically grounded TCM formula with high translational potential, meriting further clinical evaluation of its immunomodulatory, anti‐inflammatory and bone‐preserving capacities against periodontitis.

## 5 Conclusion

Our study integrated network pharmacology, single‐cell RNA sequencing, molecular docking and experimental validation to comprehensively and multidimensionally analyse the molecular mechanisms underlying Baiyaojian decoction in the treatment of periodontitis, elucidating the immunomodulatory and anti‐inflammatory roles of Baiyaojian decoction and establishing a theoretical foundation for optimising its formulation.

## Author Contributions

**Bing‐jun Chen:** data curation (lead), methodology (lead), software (lead), visualization (lead), writing – original draft (lead), writing – review and editing (lead). **Ming‐ming Li:** software (equal), visualization (equal), writing – review and editing (equal). **Zhao‐yu Zheng:** software (equal), visualization (equal). **Wen‐qin Jin:** data curation (equal). **Zhao Jin:** visualization (equal). **Yu‐ling Zuo:** funding acquisition (lead), project administration (lead), supervision (lead).

## Funding

This work was supported by Sichuan Science and Technology Program, Natural Science Foundation of Sichuan Province (Grant No. 2026NSFSC0492; 2026NSFSC1819), Xinglin Scholars Research Enhancement Program of Chengdu University of Traditional Chinese Medicine (Grant No. MPRC2024016), Special Research Project on Traditional Chinese Medicine of Sichuan Provincial Administration of Traditional Chinese Medicine (Grant No. 2023MS365) and Health Commission of Chengdu and Chengdu University of Traditional Chinese Medicine Joint Innovation Fund in 2024 (Grant No. WXLH202402019).

## Ethics Statement

The authors have nothing to report.

## Consent

The authors have nothing to report.

## Conflicts of Interest

The authors declare no conflicts of interest.

## Supporting information


**Figure S1:** The preprocessing, removing outlier samples and correcting for batch effects of GSE16134 dataset. (A) The box plot, PCA plot and dendrogram of transcriptome data before and after processing.
**Figure S2:** The preprocessing of GSE152042 and GSE171213 datasets. (A) Violin plots of nCount_RNA, nFeature_RNA and percent.mt before and after filtering. (B) UMAP plot of cell clusters.
**Figure S3:** The cell–cell communication analysis and pseudotime analysis of immune cells. (A) The circle plot of interaction number among immune cells. (B) Heatmaps of the expression of pseudotime‐related genes along pseudotime. (C) Expression of the rest of core therapeutic targets along the differentiation trajectories of plasma cells, neutrophils, macrophages and mast cells.

**Table S1:** The primer sequences for qRT‐PCR.

**Table S2:** Target genes of active ingredients.

**Table S3:** Periodontitis‐related pathogenic genes in disease database.

**Table S4:** Potential therapeutic targets of Baiyaojian decoction in the treatment of periodontitis.

## Data Availability

All the datasets (GSE16134, GSE152042 and GSE171213) can be downloaded from the GEO database (https://www.ncbi.nlm.nih.gov/geo/). Other relevant data and codes are available from the corresponding author on reasonable request.
