# Supplementary material for: Immunomodulatory Mechanism of Baiyaojian Decoction on Periodontitis: Network Pharmacology, Single‐Cell RNA Sequencing and Molecular Docking
Source: J Cell Mol Med. 2026 Jan 28;30(3):e71034. doi: 10.1111/jcmm.71034 (PMC12851902; doi:10.1111/jcmm.71034)
Supplement: Supplementary file 1 — Figure S1: The preprocessing, removing outlier samples and correcting for batch effects of GSE16134 dataset. (A) The box plot, PCA plot and dendrogram of transcriptome data before and after processing. Figure S2: The preprocessing of GSE152042 and GSE171213 datasets. (A) Violin plots of nCount_RNA, nFeature_RNA and percent.mt before and after filtering. (B) UMAP plot of cell clusters. Figure S3: The cell–cell communication analysis and pseudotime analysis of immune cells. (A) The circle plot of interaction number among immune cells. (B) Heatmaps of the expression of pseudotime‐related genes along pseudotime. (C) Expression of the rest of core therapeutic targets along the differentiation trajectories of plasma cells, neutrophils, macrophages and mast cells. [file JCMM-30-e71034-s003.docx]

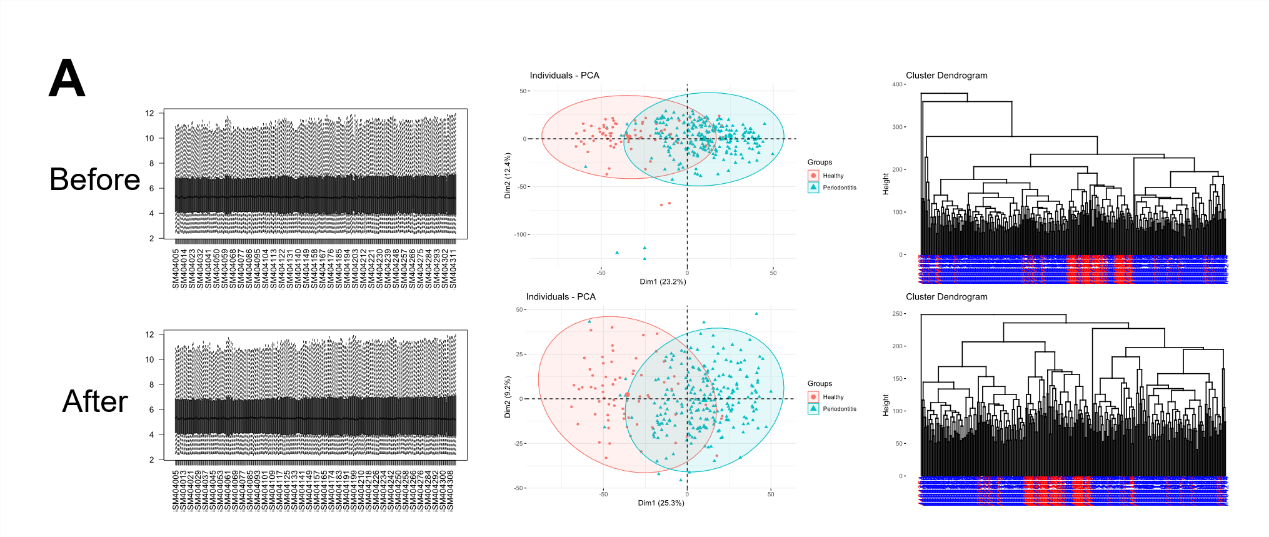


**Supplementary Figure 1 The preprocessing, removing outlier samples and correcting for batch effects of GSE16134 dataset.** (A) The box plot, PCA plot and dendrogram of transcriptome data before and after processing.


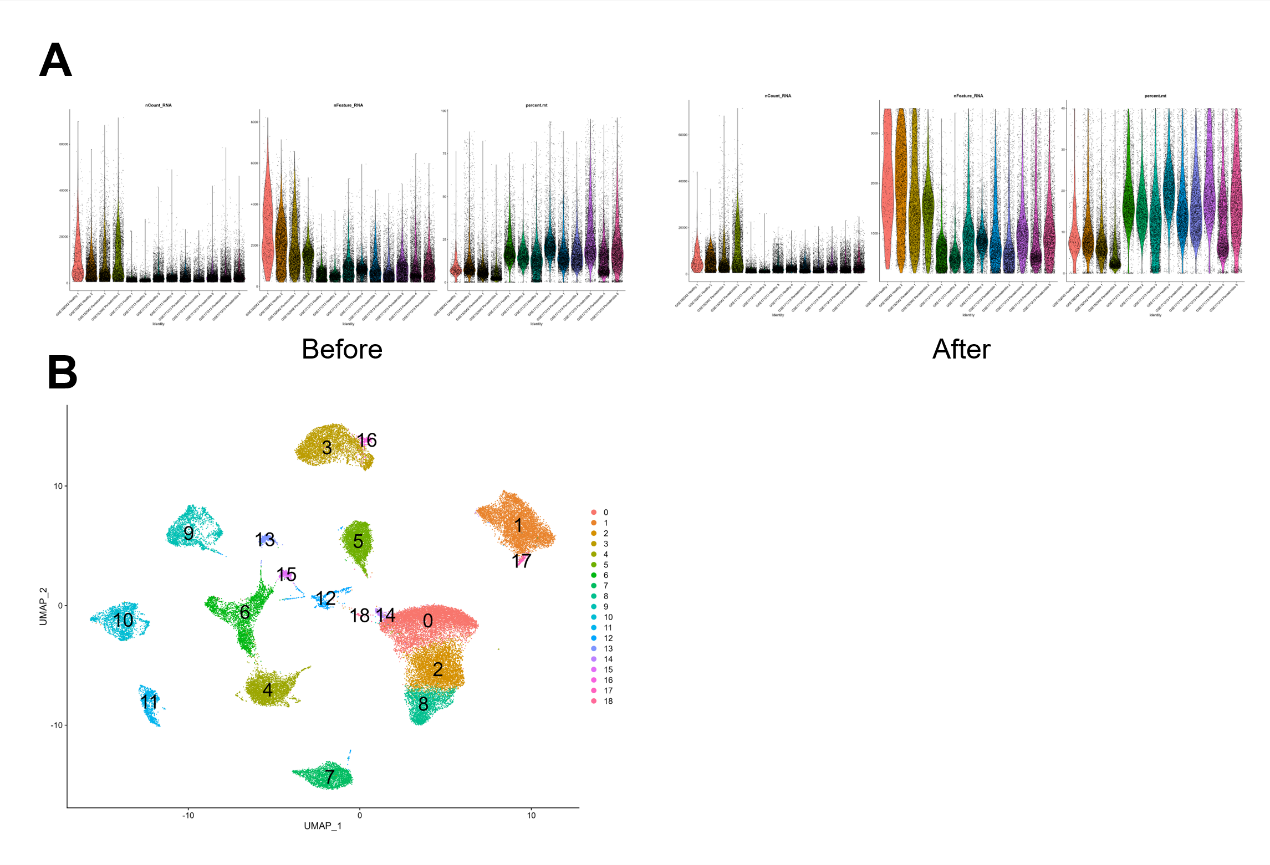


**Supplementary Figure 2 The preprocessing of GSE152042 and GSE171213 datasets.** (A) Violin plots of nCount_RNA, nFeature_RNA and percent.mt before and after filtering. (B) UMAP plot of cell clusters.

**
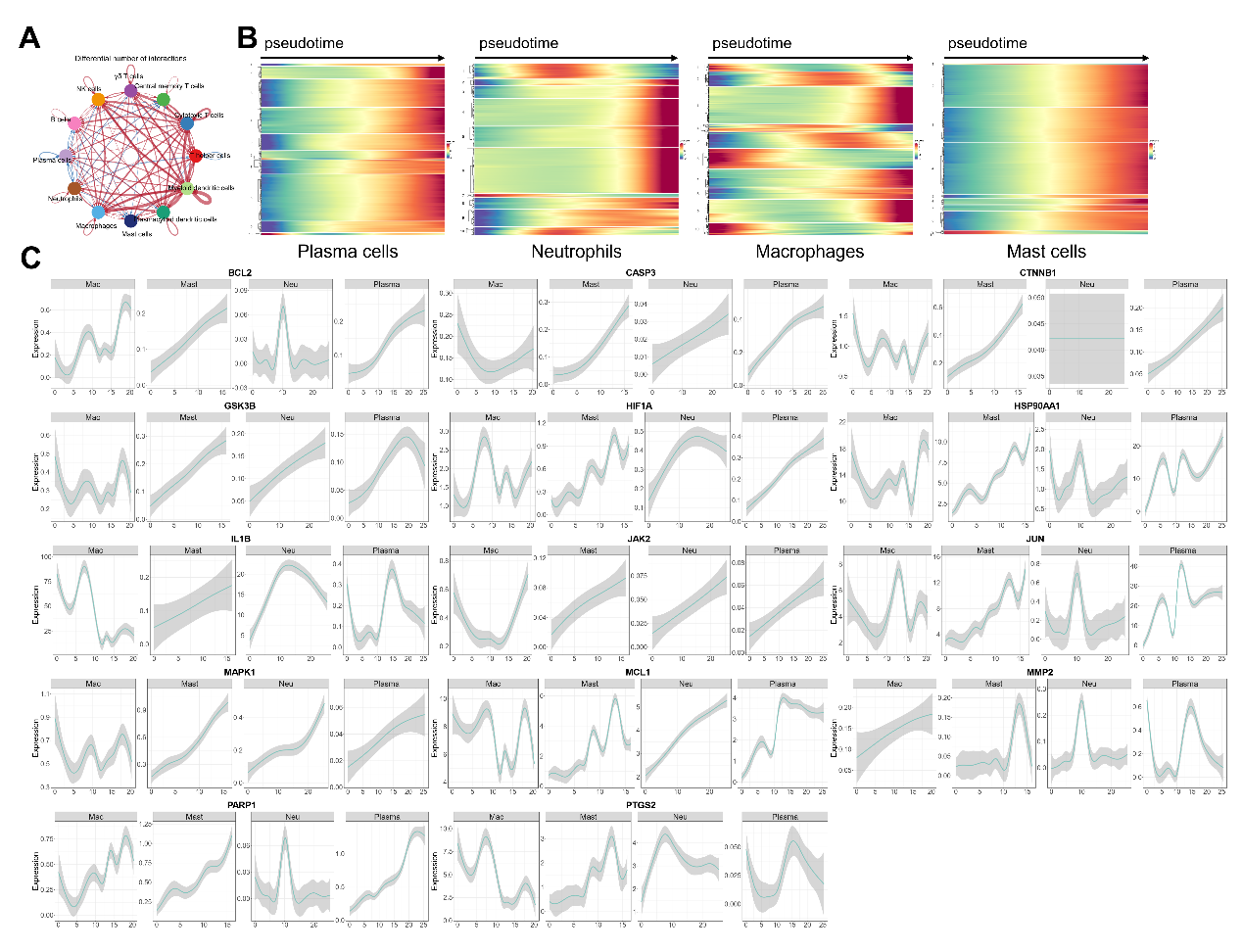
**

**Supplementary Figure 3 The cell-cell communication analysis and pseudotime analysis of immune cells.** (A) The circle plot of interaction number among immune cells. (B) Heatmaps of the expression of pseudotime-related genes along pseudotime. (C) Expression of the rest of core therapeutic targets along the differentiation trajectories of plasma cells, neutrophils, macrophages and mast cells.
