# Supplementary material for: Immunomodulatory Mechanism of Baiyaojian Decoction on Periodontitis: Network Pharmacology, Single‐Cell RNA Sequencing and Molecular Docking
Source: J Cell Mol Med. 2026 Jan 28;30(3):e71034. doi: 10.1111/jcmm.71034 (PMC12851902; doi:10.1111/jcmm.71034)
Supplement: Supplementary file 2 — Table S1: The primer sequences for qRT‐PCR. [file JCMM-30-e71034-s001.docx]

**Supplementary Table 1 The primer sequences for qRT‑PCR**

| **Gene** | **Forward** | **Reverse** |
| --- | --- | --- |
| CD38 | CAACTCTGTCTTGGCGTCAGT | CCCATACACTTTGGCAGTCTACA |
| JCHAIN | TCCTGGCGGTTTTTATTAAGGC | AGTAATCCGGGCACACTTACAT |
| XBP1 | CCCTCCAGAACATCTCCCCAT | ACATGACTGGGTCCAAGTTGT |
| IGKC | GATCTGGGACAGAATTCACTCTCA | GCCGAACGTCCAAGGGTAA |
| IGHG1 | GTTTTCGTCGTTGCCCTTTTAAG | ACCCACTGAATGAGAATCCAGAG |
| CD14 | ACGCCAGAACCTTGTGAGC | GCATGGATCTCCACCTCTACTG |
| CD80 | AAACTCGCATCTACTGGCAAA | GGTTCTTGTACTCGGGCCATA |
| C1QA | TCTGCACTGTACCCGGCTA | CCCTGGTAAATGTGACCCTTTT |
| C1QC | AGGATGGGTACGACGGACTG | GTAAGCCGGGTTCTCCCTTC |
| CCL18 | CCCAGCTCACTCTGACCACT | GTGGAATCTGCCAGGAGGTA |
| FCGR3B | CAGCTGGCATGCGGACTGA | CACTGTCCTTCTCAAGCACG |
| CSF3R | GAGCTGAGAACTACCGAACGG | GGCCTGAGGGTCTCCAAGA |
| MNDA | AACTGACATCGGAAGCAAGAG | CCTGATTCGGAGTAAACGAAGTG |
| PROK2 | GTGACAAGGACTCCCAATGTG | TGACCCAGATACTGACAGCAC |
| PDE4B | AACGCTGGAGGAATTAGACTGG | GCTCCCGGTTCAGCATTCT |
| TPSAB1 | GCAGGTGAAGGTCCCCATAAT | GACACGGGTGTAGATGCCA |
| TPSB2 | GAGAGTCCGCGACCGATACT | CTGGGCGGTGTAGAACTGTG |
| CPA3 | GGGTTTGATTGCTACCACTCTT | GCCAAGTCCTTTATGATGTCTGC |
| CMA1 | AACACTTCTACTCTTCACCACGA | GGCTTCAACACACCTGTTCTT |
| GATA1 | CTGTCCCCAATAGTGCTTATGG | GAATAGGCTGCTGAATTGAGGG |
| Akt1 | ATGAACGACGTAGCCATTGTG | TTGTAGCCAATAAAGGTGCCAT |
| Bcl2l1 | ACATCCCAGCTTCACATAACCC | CCATCCCGAAAGAGTTCATTCAC |
| Nfkb1 | ATGGCAGACGATGATCCCTAC | CGGAATCGAAATCCCCTCTGTT |
| Stat3 | CACCTTGGATTGAGAGTCAAGAC | AGGAATCGGCTATATTGCTGGT |
| Bcl2 | GCTACCGTCGTGACTTCGC | CCCCACCGAACTCAAAGAAGG |
| Hif1a | TCTCGGCGAAGCAAAGAGTC | AGCCATCTAGGGCTTTCAGATAA |
| Il1b | GAAATGCCACCTTTTGACAGTG | TGGATGCTCTCATCAGGACAG |
| Jak2 | GGAATGGCCTGCCTTACAATG | TGGCTCTATCTGCTTCACAGAAT |
| Mmp2 | ACCTGAACACTTTCTATGGCTG | CTTCCGCATGGTCTCGATG |
| Parp1 | GCTTTATCGAGTGGAGTACGC | GGAGGGAGTCCTTGGGAATAC |
| Gsk3b | ATGGCAGCAAGGTAACCACAG | TCTCGGTTCTTAAATCGCTTGTC |
| Casp3 | CTCGCTCTGGTACGGATGTG | TCCCATAAATGACCCCTTCATCA |
| Ctnnb1 | ATGGAGCCGGACAGAAAAGC | TGGGAGGTGTCAACATCTTCTT |
| Hsp90aa1 | GACGCTCTGGATAAAATCCGTT | TGGGAATGAGATTGATGTGCAG |
| Jun | TTCCTCCAGTCCGAGAGCG | TGAGAAGGTCCGAGTTCTTGG |
| Mapk1 | GGTTGTTCCCAAATGCTGACT | CAACTTCAATCCTCTTGTGAGGG |
| Mcl1 | AAAGGCGGCTGCATAAGTC | TGGCGGTATAGGTCGTCCTC |
| Ptgs2 | TTCCAATCCATGTCAAAACCGT | AGTCCGGGTACAGTCACACTT |
