# Supplementary material for: Immunomodulatory Mechanism of Baiyaojian Decoction on Periodontitis: Network Pharmacology, Single‐Cell RNA Sequencing and Molecular Docking
Source: J Cell Mol Med. 2026 Jan 28;30(3):e71034. doi: 10.1111/jcmm.71034 (PMC12851902; doi:10.1111/jcmm.71034)
Supplement: Supplementary file 3 — Table S2: Target genes of active ingredients. [file JCMM-30-e71034-s002.docx]

**Supplementary Table 2 Target genes of active ingredients**

| Ingredient | Gene |
| --- | --- |
| (2E,4E,6E,8Z)-3,7-dimethyl-9-(2,6,6-trimethyl-1-cyclohexenyl)nona-2,4,6,8-tetraen-1-ol | RBP4 |
| (2E,4E,6E,8Z)-3,7-dimethyl-9-(2,6,6-trimethyl-1-cyclohexenyl)nona-2,4,6,8-tetraen-1-ol | RXRA |
| (2E,4E,6E,8Z)-3,7-dimethyl-9-(2,6,6-trimethyl-1-cyclohexenyl)nona-2,4,6,8-tetraen-1-ol | RXRB |
| (2E,4E,6E,8Z)-3,7-dimethyl-9-(2,6,6-trimethyl-1-cyclohexenyl)nona-2,4,6,8-tetraen-1-ol | ADRA2B |
| (2E,4E,6E,8Z)-3,7-dimethyl-9-(2,6,6-trimethyl-1-cyclohexenyl)nona-2,4,6,8-tetraen-1-ol | RARG |
| (2E,4E,6E,8Z)-3,7-dimethyl-9-(2,6,6-trimethyl-1-cyclohexenyl)nona-2,4,6,8-tetraen-1-ol | RXRG |
| (2E,4E,6E,8Z)-3,7-dimethyl-9-(2,6,6-trimethyl-1-cyclohexenyl)nona-2,4,6,8-tetraen-1-ol | RARB |
| (2E,4E,6E,8Z)-3,7-dimethyl-9-(2,6,6-trimethyl-1-cyclohexenyl)nona-2,4,6,8-tetraen-1-ol | RARA |
| (2E,4E,6E,8Z)-3,7-dimethyl-9-(2,6,6-trimethyl-1-cyclohexenyl)nona-2,4,6,8-tetraen-1-ol | RORB |
| (2E,4E,6E,8Z)-3,7-dimethyl-9-(2,6,6-trimethyl-1-cyclohexenyl)nona-2,4,6,8-tetraen-1-ol | RORA |
| (2E,4E,6E,8Z)-3,7-dimethyl-9-(2,6,6-trimethyl-1-cyclohexenyl)nona-2,4,6,8-tetraen-1-ol | CYP19A1 |
| (2E,4E,6E,8Z)-3,7-dimethyl-9-(2,6,6-trimethyl-1-cyclohexenyl)nona-2,4,6,8-tetraen-1-ol | MAPK14 |
| (2E,4E,6E,8Z)-3,7-dimethyl-9-(2,6,6-trimethyl-1-cyclohexenyl)nona-2,4,6,8-tetraen-1-ol | CNR1 |
| (2E,4E,6E,8Z)-3,7-dimethyl-9-(2,6,6-trimethyl-1-cyclohexenyl)nona-2,4,6,8-tetraen-1-ol | PTPN1 |
| (2E,4E,6E,8Z)-3,7-dimethyl-9-(2,6,6-trimethyl-1-cyclohexenyl)nona-2,4,6,8-tetraen-1-ol | AR |
| (2E,4E,6E,8Z)-3,7-dimethyl-9-(2,6,6-trimethyl-1-cyclohexenyl)nona-2,4,6,8-tetraen-1-ol | MAPK1 |
| (2E,4E,6E,8Z)-3,7-dimethyl-9-(2,6,6-trimethyl-1-cyclohexenyl)nona-2,4,6,8-tetraen-1-ol | ADORA3 |
| (2E,4E,6E,8Z)-3,7-dimethyl-9-(2,6,6-trimethyl-1-cyclohexenyl)nona-2,4,6,8-tetraen-1-ol | RORC |
| (2E,4E,6E,8Z)-3,7-dimethyl-9-(2,6,6-trimethyl-1-cyclohexenyl)nona-2,4,6,8-tetraen-1-ol | EPHX2 |
| (2E,4E,6E,8Z)-3,7-dimethyl-9-(2,6,6-trimethyl-1-cyclohexenyl)nona-2,4,6,8-tetraen-1-ol | HTR2B |
| (2E,4E,6E,8Z)-3,7-dimethyl-9-(2,6,6-trimethyl-1-cyclohexenyl)nona-2,4,6,8-tetraen-1-ol | PSEN2 PSENEN NCSTN APH1A PSEN1 APH1B |
| (2E,4E,6E,8Z)-3,7-dimethyl-9-(2,6,6-trimethyl-1-cyclohexenyl)nona-2,4,6,8-tetraen-1-ol | ESR1 |
| (2E,4E,6E,8Z)-3,7-dimethyl-9-(2,6,6-trimethyl-1-cyclohexenyl)nona-2,4,6,8-tetraen-1-ol | ESR2 |
| (2E,4E,6E,8Z)-3,7-dimethyl-9-(2,6,6-trimethyl-1-cyclohexenyl)nona-2,4,6,8-tetraen-1-ol | HSD11B1 |
| (2E,4E,6E,8Z)-3,7-dimethyl-9-(2,6,6-trimethyl-1-cyclohexenyl)nona-2,4,6,8-tetraen-1-ol | PRKCD |
| (2E,4E,6E,8Z)-3,7-dimethyl-9-(2,6,6-trimethyl-1-cyclohexenyl)nona-2,4,6,8-tetraen-1-ol | C5AR1 |
| (2E,4E,6E,8Z)-3,7-dimethyl-9-(2,6,6-trimethyl-1-cyclohexenyl)nona-2,4,6,8-tetraen-1-ol | CDC25A |
| (2E,4E,6E,8Z)-3,7-dimethyl-9-(2,6,6-trimethyl-1-cyclohexenyl)nona-2,4,6,8-tetraen-1-ol | CHRM4 |
| (2E,4E,6E,8Z)-3,7-dimethyl-9-(2,6,6-trimethyl-1-cyclohexenyl)nona-2,4,6,8-tetraen-1-ol | GCGR |
| (2E,4E,6E,8Z)-3,7-dimethyl-9-(2,6,6-trimethyl-1-cyclohexenyl)nona-2,4,6,8-tetraen-1-ol | CHRM5 |
| (2E,4E,6E,8Z)-3,7-dimethyl-9-(2,6,6-trimethyl-1-cyclohexenyl)nona-2,4,6,8-tetraen-1-ol | CHRM1 |
| (2E,4E,6E,8Z)-3,7-dimethyl-9-(2,6,6-trimethyl-1-cyclohexenyl)nona-2,4,6,8-tetraen-1-ol | KCNH2 |
| (2E,4E,6E,8Z)-3,7-dimethyl-9-(2,6,6-trimethyl-1-cyclohexenyl)nona-2,4,6,8-tetraen-1-ol | CHRM3 |
| (2E,4E,6E,8Z)-3,7-dimethyl-9-(2,6,6-trimethyl-1-cyclohexenyl)nona-2,4,6,8-tetraen-1-ol | CCR1 |
| 5,7-dihydroxy-2-(3-hydroxy-4-methoxyphenyl)chroman-4-one | CA7 |
| 5,7-dihydroxy-2-(3-hydroxy-4-methoxyphenyl)chroman-4-one | CA12 |
| 5,7-dihydroxy-2-(3-hydroxy-4-methoxyphenyl)chroman-4-one | CA4 |
| 5,7-dihydroxy-2-(3-hydroxy-4-methoxyphenyl)chroman-4-one | CYP1B1 |
| 5,7-dihydroxy-2-(3-hydroxy-4-methoxyphenyl)chroman-4-one | CYP19A1 |
| 5,7-dihydroxy-2-(3-hydroxy-4-methoxyphenyl)chroman-4-one | TAS2R31 |
| 5,7-dihydroxy-2-(3-hydroxy-4-methoxyphenyl)chroman-4-one | ADORA1 |
| 5,7-dihydroxy-2-(3-hydroxy-4-methoxyphenyl)chroman-4-one | ADORA3 |
| 5,7-dihydroxy-2-(3-hydroxy-4-methoxyphenyl)chroman-4-one | ABCG2 |
| 5,7-dihydroxy-2-(3-hydroxy-4-methoxyphenyl)chroman-4-one | HSD17B1 |
| 5,7-dihydroxy-2-(3-hydroxy-4-methoxyphenyl)chroman-4-one | ESR2 |
| 5,7-dihydroxy-2-(3-hydroxy-4-methoxyphenyl)chroman-4-one | ESR1 |
| 5,7-dihydroxy-2-(3-hydroxy-4-methoxyphenyl)chroman-4-one | MAOB |
| 5,7-dihydroxy-2-(3-hydroxy-4-methoxyphenyl)chroman-4-one | ABCC1 |
| 5,7-dihydroxy-2-(3-hydroxy-4-methoxyphenyl)chroman-4-one | SHBG |
| 5,7-dihydroxy-2-(3-hydroxy-4-methoxyphenyl)chroman-4-one | CBR1 |
| 5,7-dihydroxy-2-(3-hydroxy-4-methoxyphenyl)chroman-4-one | MMP13 |
| 5,7-dihydroxy-2-(3-hydroxy-4-methoxyphenyl)chroman-4-one | PTGS1 |
| 5,7-dihydroxy-2-(3-hydroxy-4-methoxyphenyl)chroman-4-one | BACE1 |
| 5,7-dihydroxy-2-(3-hydroxy-4-methoxyphenyl)chroman-4-one | MMP12 |
| 5,7-dihydroxy-2-(3-hydroxy-4-methoxyphenyl)chroman-4-one | GRM5 |
| 5,7-dihydroxy-2-(3-hydroxy-4-methoxyphenyl)chroman-4-one | SRC |
| 5,7-dihydroxy-2-(3-hydroxy-4-methoxyphenyl)chroman-4-one | AKR1C3 |
| 5,7-dihydroxy-2-(3-hydroxy-4-methoxyphenyl)chroman-4-one | PLA2G1B |
| 5,7-dihydroxy-2-(3-hydroxy-4-methoxyphenyl)chroman-4-one | KLK1 |
| 5,7-dihydroxy-2-(3-hydroxy-4-methoxyphenyl)chroman-4-one | KLK2 |
| 5,7-dihydroxy-2-(3-hydroxy-4-methoxyphenyl)chroman-4-one | CA3 |
| 5,7-dihydroxy-2-(3-hydroxy-4-methoxyphenyl)chroman-4-one | CHRNA7 |
| 5,7-dihydroxy-2-(3-hydroxy-4-methoxyphenyl)chroman-4-one | TERT |
| 5,7-dihydroxy-2-(3-hydroxy-4-methoxyphenyl)chroman-4-one | CA2 |
| 5,7-dihydroxy-2-(3-hydroxy-4-methoxyphenyl)chroman-4-one | CA1 |
| 5,7-dihydroxy-2-(3-hydroxy-4-methoxyphenyl)chroman-4-one | CA6 |
| 5,7-dihydroxy-2-(3-hydroxy-4-methoxyphenyl)chroman-4-one | CA5A |
| 5,7-dihydroxy-2-(3-hydroxy-4-methoxyphenyl)chroman-4-one | POLB |
| 5,7-dihydroxy-2-(3-hydroxy-4-methoxyphenyl)chroman-4-one | SERPINE1 |
| 5,7-dihydroxy-2-(3-hydroxy-4-methoxyphenyl)chroman-4-one | APP |
| 5,7-dihydroxy-2-(3-hydroxy-4-methoxyphenyl)chroman-4-one | PLA2G5 |
| 5,7-dihydroxy-2-(3-hydroxy-4-methoxyphenyl)chroman-4-one | PLA2G10 |
| 5,7-dihydroxy-2-(3-hydroxy-4-methoxyphenyl)chroman-4-one | PLG |
| 5,7-dihydroxy-2-(3-hydroxy-4-methoxyphenyl)chroman-4-one | NOX4 |
| 5,7-dihydroxy-2-(3-hydroxy-4-methoxyphenyl)chroman-4-one | CA13 |
| 5,7-dihydroxy-2-(3-hydroxy-4-methoxyphenyl)chroman-4-one | CA5B |
| 5,7-dihydroxy-2-(3-hydroxy-4-methoxyphenyl)chroman-4-one | ACHE |
| 5,7-dihydroxy-2-(3-hydroxy-4-methoxyphenyl)chroman-4-one | GUSB |
| 5,7-dihydroxy-2-(3-hydroxy-4-methoxyphenyl)chroman-4-one | CES1 |
| 5,7-dihydroxy-2-(3-hydroxy-4-methoxyphenyl)chroman-4-one | SLC5A2 |
| 5,7-dihydroxy-2-(3-hydroxy-4-methoxyphenyl)chroman-4-one | CES2 |
| 5,7-dihydroxy-2-(3-hydroxy-4-methoxyphenyl)chroman-4-one | RXRA |
| 5,7-dihydroxy-2-(3-hydroxy-4-methoxyphenyl)chroman-4-one | KDR |
| 5,7-dihydroxy-2-(3-hydroxy-4-methoxyphenyl)chroman-4-one | GRM2 |
| 5,7-dihydroxy-2-(3-hydroxy-4-methoxyphenyl)chroman-4-one | AKR1B1 |
| 5,7-dihydroxy-2-(3-hydroxy-4-methoxyphenyl)chroman-4-one | CDK1 CCNB1 |
| 5,7-dihydroxy-2-(3-hydroxy-4-methoxyphenyl)chroman-4-one | CCNE1 CDK2 |
| 5,7-dihydroxy-2-(3-hydroxy-4-methoxyphenyl)chroman-4-one | DUSP3 |
| 5,7-dihydroxy-2-(3-hydroxy-4-methoxyphenyl)chroman-4-one | NTRK1 |
| 5,7-dihydroxy-2-(3-hydroxy-4-methoxyphenyl)chroman-4-one | AURKA |
| 5,7-dihydroxy-2-(3-hydroxy-4-methoxyphenyl)chroman-4-one | CA9 |
| 5,7-dihydroxy-2-(3-hydroxy-4-methoxyphenyl)chroman-4-one | TOP1 |
| 5,7-dihydroxy-2-(3-hydroxy-4-methoxyphenyl)chroman-4-one | DNMT1 |
| 5,7-dihydroxy-2-(3-hydroxy-4-methoxyphenyl)chroman-4-one | PGD |
| 5,7-dihydroxy-2-(3-hydroxy-4-methoxyphenyl)chroman-4-one | ST3GAL3 |
| 5,7-dihydroxy-2-(3-hydroxy-4-methoxyphenyl)chroman-4-one | FUT7 |
| 5,7-dihydroxy-2-(3-hydroxy-4-methoxyphenyl)chroman-4-one | FUT4 |
| 5,7-dihydroxy-2-(3-hydroxy-4-methoxyphenyl)chroman-4-one | STAT1 |
| 5,7-dihydroxy-2-(3-hydroxy-4-methoxyphenyl)chroman-4-one | SQLE |
| 5,7-dihydroxy-2-(3-hydroxy-4-methoxyphenyl)chroman-4-one | FFAR1 |
| 5,7-dihydroxy-2-(3-hydroxy-4-methoxyphenyl)chroman-4-one | PIM1 |
| 5,7-dihydroxy-2-(3-hydroxy-4-methoxyphenyl)chroman-4-one | PIM2 |
| 5,7-dihydroxy-2-(3-hydroxy-4-methoxyphenyl)chroman-4-one | PIM3 |
| 5,7-dihydroxy-2-(3-hydroxy-4-methoxyphenyl)chroman-4-one | MMP2 |
| 5,7-dihydroxy-2-(3-hydroxy-4-methoxyphenyl)chroman-4-one | IGF1R |
| 5,7-dihydroxy-2-(3-hydroxy-4-methoxyphenyl)chroman-4-one | MMP9 |
| 5,7-dihydroxy-2-(3-hydroxy-4-methoxyphenyl)chroman-4-one | ODC1 |
| 5,7-dihydroxy-2-(3-hydroxy-4-methoxyphenyl)chroman-4-one | PARP1 |
| 5,7-dihydroxy-2-(3-hydroxy-4-methoxyphenyl)chroman-4-one | DNM1 |
| 5,7-dihydroxy-2-(3-hydroxy-4-methoxyphenyl)chroman-4-one | TNKS2 |
| 5,7-dihydroxy-2-(3-hydroxy-4-methoxyphenyl)chroman-4-one | TNKS |
| 5,7-dihydroxy-2-(3-hydroxy-4-methoxyphenyl)chroman-4-one | MET |
| 5,7-dihydroxy-2-(3-hydroxy-4-methoxyphenyl)chroman-4-one | DYRK1A |
| 5,7-dihydroxy-2-(3-hydroxy-4-methoxyphenyl)chroman-4-one | ABCB1 |
| 5,7-dihydroxy-2-(3-hydroxy-4-methoxyphenyl)chroman-4-one | PPARG |
| 5,7-dihydroxy-2-(3-hydroxy-4-methoxyphenyl)chroman-4-one | MAP4K4 |
| 5,7-dihydroxy-2-(3-hydroxy-4-methoxyphenyl)chroman-4-one | PLA2G2A |
| 5,7-dihydroxy-2-(3-hydroxy-4-methoxyphenyl)chroman-4-one | CDK2 CCNA1 CCNA2 |
| 5,7-dihydroxy-2-(3-hydroxy-4-methoxyphenyl)chroman-4-one | PRMT1 |
| 5,7-dihydroxy-2-(3-hydroxy-4-methoxyphenyl)chroman-4-one | HSD17B2 |
| 5,7-dihydroxy-2-(3-hydroxy-4-methoxyphenyl)chroman-4-one | EDNRA |
| 5,7-dihydroxy-2-(3-hydroxy-4-methoxyphenyl)chroman-4-one | BCL2 |
| Ammidin | MAOA |
| Ammidin | ACHE |
| Ammidin | KCNA3 |
| Ammidin | BACE1 |
| Ammidin | CYP1A2 |
| Ammidin | GABRB3 GABRA3 GABRG2 |
| Ammidin | GABRB3 GABRG2 GABRA1 |
| Ammidin | GABRB3 GABRG2 GABRA5 |
| Ammidin | GABRA2 GABRB3 GABRG2 |
| Ammidin | CYP19A1 |
| Ammidin | MAPK14 |
| Ammidin | NOS2 |
| Ammidin | ADORA1 |
| Ammidin | PDE10A |
| Ammidin | ADORA2B |
| Ammidin | NTRK1 |
| Ammidin | HDAC3 |
| Ammidin | ADRA2A |
| Ammidin | ADRA2C |
| Ammidin | HDAC2 |
| Ammidin | ADRA2B |
| Ammidin | ADRA1B |
| Ammidin | JAK2 |
| Ammidin | HDAC1 |
| Ammidin | CSNK1D |
| Ammidin | FNTA FNTB |
| Ammidin | PGGT1B FNTA |
| Ammidin | PGGT1B |
| Ammidin | PTGER1 |
| Ammidin | CCNE2 CDK2 CCNE1 |
| Ammidin | TAAR1 |
| Ammidin | MTOR |
| Ammidin | PIK3CD |
| Ammidin | PIK3CB |
| Ammidin | GRM5 |
| Ammidin | PIK3CG |
| Ammidin | MCHR1 |
| Ammidin | PIK3CA |
| Ammidin | PDE5A |
| Ammidin | PRKDC |
| Ammidin | HSF1 |
| Ammidin | GYS1 |
| Ammidin | BRS3 |
| Ammidin | TGFBR1 |
| Ammidin | PTK2B |
| Ammidin | MAOB |
| Ammidin | FAAH |
| Ammidin | CLK4 |
| Ammidin | GSK3A |
| Ammidin | DYRK1A |
| Ammidin | PDE8B |
| Ammidin | PGR |
| Ammidin | MAPKAPK2 |
| Ammidin | MAPK8 |
| Ammidin | CSNK1G1 |
| Ammidin | CCR2 |
| Ammidin | ADRA1A |
| Ammidin | PPOX |
| Ammidin | PIM1 |
| Ammidin | CA9 |
| Ammidin | PTGER4 |
| Ammidin | PTGER2 |
| Ammidin | PTGER3 |
| Ammidin | HCRTR2 |
| Ammidin | HCRTR1 |
| Ammidin | BCHE |
| Ammidin | CNR1 |
| Ammidin | PTGS1 |
| Ammidin | PTGS2 |
| Ammidin | CNR2 |
| Ammidin | MAP2K1 |
| Ammidin | SRD5A1 |
| Ammidin | FLT3 |
| Ammidin | JAK1 |
| Ammidin | TYK2 |
| Ammidin | NAAA |
| Ammidin | CA12 |
| Ammidin | PTAFR |
| Ammidin | HRH3 |
| anomalin | CNR1 |
| anomalin | CNR2 |
| anomalin | ELANE |
| anomalin | CSF1R |
| anomalin | LCK |
| anomalin | HCRTR2 |
| anomalin | HCRTR1 |
| anomalin | GSK3B |
| anomalin | CTSS |
| anomalin | RBP4 |
| anomalin | CTSV |
| anomalin | CTSL |
| anomalin | ADORA3 |
| anomalin | SCN2A |
| anomalin | SCN10A |
| anomalin | ELOVL6 |
| anomalin | FLT1 |
| anomalin | CCND3 CCND1 CDK4 CCND2 |
| anomalin | CXCR2 |
| anomalin | KDR |
| anomalin | P2RX3 |
| anomalin | GRM5 |
| anomalin | JAK3 |
| anomalin | CFD |
| anomalin | JAK2 |
| anomalin | CHRM3 |
| anomalin | SCN9A |
| anomalin | PPIA |
| anomalin | MAPK1 |
| anomalin | PABPC1 |
| anomalin | IDH1 |
| anomalin | CHRM2 |
| anomalin | CHRM1 |
| anomalin | PRKDC |
| anomalin | TSPO |
| anomalin | PDE10A |
| anomalin | CPT1A |
| anomalin | MMP9 |
| anomalin | MAPK11 |
| anomalin | VCP |
| anomalin | BDKRB1 |
| anomalin | CCND1 CDK4 |
| anomalin | EGFR |
| anomalin | CCNE2 CDK2 CCNE1 |
| anomalin | CCNB3 CDK1 CCNB1 CCNB2 |
| anomalin | ADORA1 |
| anomalin | ADORA2A |
| anomalin | GRM4 |
| anomalin | PTGS2 |
| anomalin | FPR1 |
| anomalin | PTPN1 |
| anomalin | NOS2 |
| anomalin | HTR1A |
| anomalin | DRD2 |
| anomalin | CTSK |
| anomalin | KCNJ5 KCNJ3 |
| anomalin | KCNJ6 KCNJ3 |
| anomalin | HTR7 |
| anomalin | HTR6 |
| anomalin | NOS1 |
| anomalin | GRK2 |
| anomalin | PSMB5 |
| anomalin | TRPA1 |
| anomalin | IKBKB |
| anomalin | SAE1 UBA2 |
| anomalin | PDE4B |
| anomalin | MMP3 |
| anomalin | MMP1 |
| anomalin | STK33 |
| anomalin | ITGAV ITGB3 |
| anomalin | PIM1 |
| anomalin | DRD4 |
| anomalin | PRKCG |
| anomalin | CMA1 |
| anomalin | PIM2 |
| anomalin | NPY5R |
| anomalin | TRPV1 |
| anomalin | CHRM4 |
| anomalin | CDK5R1 CDK5 |
| anomalin | PSEN2 PSENEN NCSTN APH1A PSEN1 APH1B |
| anomalin | KCNK3 |
| anomalin | KCNK9 |
| anomalin | F10 |
| anomalin | PDE7A |
| anomalin | CDK2 CCNA1 CCNA2 |
| anomalin | FAAH |
| anomalin | NTRK1 |
| anomalin | CCKBR |
| anomalin | PDE9A |
| anomalin | TACR3 |
| anomalin | NTRK2 |
| anomalin | NTRK3 |
| anomalin | MAPK8 |
| anomalin | MAPK10 |
| anomalin | MAPK9 |
| anomalin | FAP |
| anomalin | P2RX7 |
| anomalin | PTK2 |
| anomalin | PLK1 |
| anomalin | GRM2 |
| caffeic acid ethyl ester | CA2 |
| caffeic acid ethyl ester | CA7 |
| caffeic acid ethyl ester | CA1 |
| caffeic acid ethyl ester | CA12 |
| caffeic acid ethyl ester | CA14 |
| caffeic acid ethyl ester | CA9 |
| caffeic acid ethyl ester | AKR1B1 |
| caffeic acid ethyl ester | ALOX5 |
| caffeic acid ethyl ester | MAOB |
| caffeic acid ethyl ester | MMP9 |
| caffeic acid ethyl ester | MMP1 |
| caffeic acid ethyl ester | MMP2 |
| caffeic acid ethyl ester | EGFR |
| caffeic acid ethyl ester | CA6 |
| caffeic acid ethyl ester | AKR1B10 |
| caffeic acid ethyl ester | PTPN1 |
| caffeic acid ethyl ester | CA5B |
| caffeic acid ethyl ester | CA5A |
| caffeic acid ethyl ester | LCK |
| caffeic acid ethyl ester | FYN |
| caffeic acid ethyl ester | AKR1C4 |
| caffeic acid ethyl ester | AKR1C2 |
| caffeic acid ethyl ester | ESR1 |
| caffeic acid ethyl ester | ESR2 |
| caffeic acid ethyl ester | PTGS1 |
| caffeic acid ethyl ester | SLC6A2 |
| caffeic acid ethyl ester | ERBB2 |
| caffeic acid ethyl ester | FLT1 |
| caffeic acid ethyl ester | FLT4 |
| caffeic acid ethyl ester | PDGFRA |
| caffeic acid ethyl ester | AURKB |
| caffeic acid ethyl ester | KDR |
| caffeic acid ethyl ester | AURKC |
| caffeic acid ethyl ester | AURKA |
| caffeic acid ethyl ester | CA4 |
| caffeic acid ethyl ester | GUSB |
| caffeic acid ethyl ester | PLAA |
| caffeic acid ethyl ester | TTR |
| caffeic acid ethyl ester | MET |
| caffeic acid ethyl ester | AKR1C3 |
| caffeic acid ethyl ester | CA13 |
| caffeic acid ethyl ester | ELANE |
| caffeic acid ethyl ester | CDK4 |
| caffeic acid ethyl ester | CDK2 |
| caffeic acid ethyl ester | ST6GAL1 |
| caffeic acid ethyl ester | PDGFRA PDGFRB |
| caffeic acid ethyl ester | TMIGD3 |
| caffeic acid ethyl ester | ALPL |
| caffeic acid ethyl ester | PARP1 |
| caffeic acid ethyl ester | TNKS2 |
| caffeic acid ethyl ester | TNKS |
| caffeic acid ethyl ester | ALPG |
| caffeic acid ethyl ester | DAO |
| caffeic acid ethyl ester | MAPK1 |
| caffeic acid ethyl ester | MIF |
| caffeic acid ethyl ester | ADORA1 |
| caffeic acid ethyl ester | GRM4 |
| caffeic acid ethyl ester | CA3 |
| caffeic acid ethyl ester | HDAC4 |
| caffeic acid ethyl ester | CBFB |
| caffeic acid ethyl ester | RPS6KB2 |
| caffeic acid ethyl ester | CDC25A |
| caffeic acid ethyl ester | ADORA2A |
| caffeic acid ethyl ester | CDK5R1 CDK5 |
| caffeic acid ethyl ester | GRK6 |
| caffeic acid ethyl ester | XDH |
| caffeic acid ethyl ester | PDPK1 |
| caffeic acid ethyl ester | GSK3B |
| caffeic acid ethyl ester | ALOX15B |
| caffeic acid ethyl ester | RET |
| caffeic acid ethyl ester | CYP11B2 |
| caffeic acid ethyl ester | GPR84 |
| caffeic acid ethyl ester | DYRK1A |
| caffeic acid ethyl ester | DYRK1B |
| Caffeic acid | CA2 |
| Caffeic acid | ALOX5 |
| Caffeic acid | CA7 |
| Caffeic acid | CA1 |
| Caffeic acid | CA6 |
| Caffeic acid | MMP9 |
| Caffeic acid | CA12 |
| Caffeic acid | MMP1 |
| Caffeic acid | MMP2 |
| Caffeic acid | PTPN1 |
| Caffeic acid | CA14 |
| Caffeic acid | CA9 |
| Caffeic acid | CA5B |
| Caffeic acid | CA5A |
| Caffeic acid | CA3 |
| Caffeic acid | AKR1B1 |
| Caffeic acid | ESR2 |
| Caffeic acid | CA4 |
| Caffeic acid | AKR1B10 |
| Caffeic acid | HCAR2 |
| Caffeic acid | MIF |
| Caffeic acid | CA13 |
| Caffeic acid | NQO2 |
| Caffeic acid | TLR4 |
| Caffeic acid | ERBB2 |
| Caffeic acid | ESR1 |
| Caffeic acid | SLC6A2 |
| Caffeic acid | TTR |
| Caffeic acid | MAPK1 |
| Caffeic acid | AKR1C3 |
| Caffeic acid | AKR1C4 |
| Caffeic acid | AKR1C2 |
| Caffeic acid | SYK |
| Caffeic acid | APP |
| Caffeic acid | EGFR |
| Caffeic acid | FYN |
| Caffeic acid | LCK |
| Caffeic acid | PTGS1 |
| Caffeic acid | PIK3CB |
| Caffeic acid | CYP1A2 |
| Caffeic acid | CYP2C9 |
| Caffeic acid | CYP3A4 |
| Caffeic acid | CYP2C19 |
| Caffeic acid | PIK3CA |
| Caffeic acid | ELANE |
| Caffeic acid | F3 |
| Caffeic acid | HSD11B1 |
| Caffeic acid | NFE2L2 |
| Caffeic acid | STAT3 |
| Coumestrol | ESR1 |
| Coumestrol | ESR2 |
| Coumestrol | ALOX5 |
| Coumestrol | CA12 |
| Coumestrol | XDH |
| Coumestrol | AKR1B1 |
| Coumestrol | EGFR |
| Coumestrol | CBR1 |
| Coumestrol | CA7 |
| Coumestrol | GSK3B |
| Coumestrol | CSNK2A1 |
| Coumestrol | CA4 |
| Coumestrol | AURKB |
| Coumestrol | MAOA |
| Coumestrol | CA9 |
| Coumestrol | IGF1R |
| Coumestrol | KDR |
| Coumestrol | PLK1 |
| Coumestrol | MET |
| Coumestrol | ACHE |
| Coumestrol | CA2 |
| Coumestrol | CCND1 CDK4 |
| Coumestrol | PDGFRB |
| Coumestrol | FLT4 |
| Coumestrol | CDK2 CCNA1 CCNA2 |
| Coumestrol | PLK4 |
| Coumestrol | TEK |
| Coumestrol | AURKA |
| Coumestrol | MAP3K8 |
| Coumestrol | EPHB4 |
| Coumestrol | SQLE |
| Coumestrol | FGR |
| Coumestrol | LYN |
| Coumestrol | PARP1 |
| Coumestrol | TNKS2 |
| Coumestrol | TNKS |
| Coumestrol | NFKB1 |
| Coumestrol | NOX4 |
| Coumestrol | FLT3 |
| Coumestrol | CCNB3 CDK1 CCNB1 CCNB2 |
| Coumestrol | CDK6 |
| Coumestrol | SYK |
| Coumestrol | ABCC1 |
| Coumestrol | TTR |
| Coumestrol | CFTR |
| Coumestrol | CA1 |
| Coumestrol | TYR |
| Coumestrol | PTGS2 |
| Coumestrol | GSR |
| Coumestrol | HSP90AA1 |
| Coumestrol | ALOX12 |
| Coumestrol | GPR35 |
| Coumestrol | PFKFB3 |
| Coumestrol | SRC |
| Coumestrol | PTK2 |
| Coumestrol | CA6 |
| Coumestrol | CA14 |
| Coumestrol | CA13 |
| Coumestrol | AKT1 |
| Coumestrol | CA5A |
| Coumestrol | BACE1 |
| Coumestrol | NUAK1 |
| Coumestrol | CDK5R1 CDK5 |
| Coumestrol | GLO1 |
| Coumestrol | APP |
| Coumestrol | MMP9 |
| Coumestrol | MMP2 |
| Coumestrol | MMP12 |
| Coumestrol | CD38 |
| Coumestrol | TOP1 |
| Coumestrol | ARG1 |
| Coumestrol | CDK2 |
| Coumestrol | PTPRS |
| Coumestrol | HSD17B2 |
| Coumestrol | AHR |
| Coumestrol | PTP4A3 |
| Coumestrol | CRHR1 |
| Coumestrol | DRD3 |
| Coumestrol | TERT |
| Decursin | PARP1 |
| Decursin | NAAA |
| Decursin | NPY5R |
| Decursin | GCGR |
| Decursin | ASAH1 |
| Decursin | CCNB3 CDK1 CCNB1 CCNB2 |
| Decursin | HTR1A |
| Decursin | OGT |
| Decursin | MAP4K4 |
| Decursin | IDH1 |
| Decursin | SCD |
| Decursin | HPGD |
| Decursin | ABL1 |
| Decursin | RPS6KB1 |
| Decursin | AURKA |
| Decursin | PIM1 |
| Decursin | AURKB |
| Decursin | GSK3B |
| Decursin | JAK2 |
| Decursin | ROCK2 |
| Decursin | PDE4D |
| Decursin | SCN9A |
| Decursin | TRPV1 |
| Decursin | CRHR1 |
| Decursin | CTSK |
| Decursin | PDE3A |
| Decursin | PDE3B |
| Decursin | PDE4B |
| Decursin | PIK3CG |
| Decursin | HSD11B1 |
| Decursin | PKM |
| Decursin | CFD |
| Decursin | EPHX2 |
| Decursin | MMP1 |
| Decursin | ADAMTS5 |
| Decursin | JAK1 |
| Decursin | FFAR1 |
| Decursin | MTNR1A |
| Decursin | MTNR1B |
| Decursin | SRC |
| Decursin | GRM5 |
| Decursin | GRM1 |
| Decursin | TBXAS1 |
| Decursin | FNTA FNTB |
| Decursin | KDR |
| Decursin | SLC5A1 |
| Decursin | BRD4 |
| Decursin | DUT |
| Decursin | TDP2 |
| Decursin | PDE10A |
| Decursin | IDO1 |
| Decursin | PARP2 |
| Decursin | SRD5A2 |
| Decursin | CHRM1 |
| Decursin | CHEK2 |
| Decursin | RAF1 |
| Decursin | CDK2 CCNA1 CCNA2 |
| Decursin | PDE4A |
| Decursin | PDE4C |
| Decursin | PLK1 |
| Decursin | ALOX5AP |
| Decursin | PLK3 |
| Decursin | PLK2 |
| Decursin | PDGFRB |
| Decursin | FLT4 |
| Decursin | CHRNA4 CHRNB2 |
| Decursin | EPHX1 |
| Decursin | PPARG |
| Decursin | CHRNA7 |
| Decursin | SLC10A2 |
| Decursin | HSD17B2 |
| Decursin | HSD17B1 |
| Decursin | CCNC CDK8 |
| Decursin | HCRTR2 |
| Decursin | HCRTR1 |
| Decursin | SCN10A |
| Decursin | CDK8 |
| Decursin | MMP13 |
| Decursin | MMP3 |
| Decursin | CDK2 |
| Decursin | MMP9 |
| Decursin | DYRK2 |
| Decursin | MMP8 |
| Decursin | RORC |
| Decursin | GRIN1 GRIN2B |
| Decursin | DRD1 |
| Decursin | JAK3 |
| Decursin | PLAU |
| Decursin | SIRT2 |
| Decursin | ERBB2 |
| Decursin | DRD4 |
| Decursin | ICAM1 |
| Decursin | SELE |
| Decursin | PIM2 |
| Decursin | GRM2 |
| Decursin | PTAFR |
| Decursin | CTSS |
| Decursin | CCNA2 CDK2 |
| Decursin | TTK |
| Decursin | CTSB |
| Diosmetin | ABCC1 |
| Diosmetin | CYP1B1 |
| Diosmetin | XDH |
| Diosmetin | CA2 |
| Diosmetin | CA7 |
| Diosmetin | CA12 |
| Diosmetin | CA4 |
| Diosmetin | AKR1B1 |
| Diosmetin | CDK5R1 CDK5 |
| Diosmetin | CCNB3 CDK1 CCNB1 CCNB2 |
| Diosmetin | ARG1 |
| Diosmetin | PLG |
| Diosmetin | PTPRS |
| Diosmetin | ABCB1 |
| Diosmetin | APP |
| Diosmetin | NOX4 |
| Diosmetin | MAOA |
| Diosmetin | FLT3 |
| Diosmetin | ALOX5 |
| Diosmetin | ADORA1 |
| Diosmetin | GLO1 |
| Diosmetin | GSK3B |
| Diosmetin | MMP9 |
| Diosmetin | MMP2 |
| Diosmetin | ABCG2 |
| Diosmetin | SYK |
| Diosmetin | PARP1 |
| Diosmetin | TTR |
| Diosmetin | MMP12 |
| Diosmetin | CD38 |
| Diosmetin | AKR1B10 |
| Diosmetin | TNKS2 |
| Diosmetin | TNKS |
| Diosmetin | TOP1 |
| Diosmetin | PIM1 |
| Diosmetin | ADORA2A |
| Diosmetin | ACHE |
| Diosmetin | CDK6 |
| Diosmetin | CYP19A1 |
| Diosmetin | PLA2G2A |
| Diosmetin | TERT |
| Diosmetin | ESR1 |
| Diosmetin | ESR2 |
| Diosmetin | CSNK2A1 |
| Diosmetin | HSD17B1 |
| Diosmetin | CBR1 |
| Diosmetin | OPRD1 |
| Diosmetin | IGF1R |
| Diosmetin | EGFR |
| Diosmetin | ALOX15 |
| Diosmetin | ALOX12 |
| Diosmetin | HSD17B2 |
| Diosmetin | CA1 |
| Diosmetin | CA9 |
| Diosmetin | KIT |
| Diosmetin | CDK2 |
| Diosmetin | F2 |
| Diosmetin | CDK1 |
| Diosmetin | PTGS2 |
| Diosmetin | CFTR |
| Diosmetin | AVPR2 |
| Diosmetin | AURKB |
| Diosmetin | DRD4 |
| Diosmetin | MPO |
| Diosmetin | PIK3R1 |
| Diosmetin | DAPK1 |
| Diosmetin | PYGL |
| Diosmetin | SRC |
| Diosmetin | PTK2 |
| Diosmetin | KDR |
| Diosmetin | MMP13 |
| Diosmetin | MMP3 |
| Diosmetin | CA3 |
| Diosmetin | PLK1 |
| Diosmetin | CA6 |
| Diosmetin | PKN1 |
| Diosmetin | CA14 |
| Diosmetin | MET |
| Diosmetin | NEK2 |
| Diosmetin | CXCR1 |
| Diosmetin | CAMK2B |
| Diosmetin | ALK |
| Diosmetin | AKT1 |
| Diosmetin | NEK6 |
| Diosmetin | PLA2G1B |
| Diosmetin | CA5A |
| Diosmetin | BACE1 |
| Diosmetin | AXL |
| Diosmetin | NUAK1 |
| Diosmetin | AKR1C2 |
| Diosmetin | AKR1C1 |
| Diosmetin | AKR1C3 |
| Diosmetin | AKR1C4 |
| Diosmetin | CA13 |
| Diosmetin | AKR1A1 |
| Diosmetin | MCL1 |
| Diosmetin | GPR35 |
| Diosmetin | ST6GAL1 |
| Diosmetin | AMY1A |
| Diosmetin | GRK6 |
| divaricatacid | KDM4A |
| divaricatacid | PDE5A |
| divaricatacid | MKNK2 |
| divaricatacid | MME |
| divaricatacid | CYSLTR1 |
| divaricatacid | GRK6 |
| divaricatacid | IMPDH2 |
| divaricatacid | KDM4C |
| divaricatacid | HMGCR |
| divaricatacid | IGFBP3 |
| divaricatacid | ACE |
| divaricatacid | MAPK8 |
| divaricatacid | MPI |
| divaricatacid | ANPEP |
| divaricatacid | AMPD3 |
| divaricatacid | LTA4H |
| divaricatacid | ECE1 |
| divaricatacid | IMPDH1 |
| divaricatacid | FABP4 |
| divaricatacid | CSNK2A1 |
| divaricatacid | EGLN1 |
| divaricatacid | FLT1 |
| divaricatacid | KIT |
| divaricatacid | KDR |
| divaricatacid | GPR35 |
| divaricatacid | PYGL |
| divaricatacid | MAPK1 |
| divaricatacid | ACLY |
| divaricatacid | GSK3B |
| divaricatacid | GSK3A |
| divaricatacid | PARP1 |
| divaricatacid | CXCR2 |
| divaricatacid | KDM4D |
| divaricatacid | ERBB2 |
| divaricatacid | PTGIR |
| divaricatacid | AGTR1 |
| divaricatacid | ERN1 |
| divaricatacid | CASP3 |
| divaricatacid | NOX4 |
| divaricatacid | IDE |
| divaricatacid | FOLH1 |
| divaricatacid | HAO2 |
| divaricatacid | MARS |
| divaricatacid | OGA |
| divaricatacid | SF3B3 |
| divaricatacid | GSR |
| divaricatacid | FDFT1 |
| divaricatacid | PSMB5 |
| divaricatacid | EGLN3 |
| divaricatacid | F2 |
| divaricatacid | SLC6A2 |
| divaricatacid | KDM3A |
| divaricatacid | KDM5B |
| divaricatacid | FBP1 |
| divaricatacid | CSNK2A2 |
| divaricatacid | FFAR1 |
| divaricatacid | GRIK1 |
| divaricatacid | MMP16 |
| divaricatacid | MMP9 |
| divaricatacid | MMP1 |
| divaricatacid | GRIK2 |
| divaricatacid | GRIK3 |
| divaricatacid | MMP8 |
| divaricatacid | NR4A1 |
| divaricatacid | GABRA1 GABRB2 GABRG2 |
| divaricatacid | CASP6 |
| divaricatacid | CASP7 |
| divaricatacid | CASP8 |
| divaricatacid | CASP1 |
| divaricatacid | CASP2 |
| divaricatol | HSP90AA1 |
| divaricatol | FNTA FNTB |
| divaricatol | HIF1A |
| divaricatol | CCR4 |
| divaricatol | PIK3CA |
| FERULIC ACID (CIS) | CA2 |
| FERULIC ACID (CIS) | CA7 |
| FERULIC ACID (CIS) | CA1 |
| FERULIC ACID (CIS) | CA6 |
| FERULIC ACID (CIS) | CA12 |
| FERULIC ACID (CIS) | CA14 |
| FERULIC ACID (CIS) | CA9 |
| FERULIC ACID (CIS) | CA5A |
| FERULIC ACID (CIS) | CA5B |
| FERULIC ACID (CIS) | MAOB |
| FERULIC ACID (CIS) | AKR1B1 |
| FERULIC ACID (CIS) | CA13 |
| FERULIC ACID (CIS) | ALOX5 |
| FERULIC ACID (CIS) | MMP9 |
| FERULIC ACID (CIS) | MMP1 |
| FERULIC ACID (CIS) | MMP2 |
| FERULIC ACID (CIS) | PTPN1 |
| FERULIC ACID (CIS) | CA3 |
| FERULIC ACID (CIS) | APP |
| FERULIC ACID (CIS) | NFE2L2 |
| FERULIC ACID (CIS) | STAT3 |
| FERULIC ACID (CIS) | HSD11B1 |
| FERULIC ACID (CIS) | TLR4 |
| FERULIC ACID (CIS) | MET |
| FERULIC ACID (CIS) | TTR |
| FERULIC ACID (CIS) | ESR2 |
| FERULIC ACID (CIS) | CA4 |
| FERULIC ACID (CIS) | CYP1A1 |
| FERULIC ACID (CIS) | CYP1A2 |
| FERULIC ACID (CIS) | NQO2 |
| FERULIC ACID (CIS) | CYP1B1 |
| FERULIC ACID (CIS) | PTGS1 |
| FERULIC ACID (CIS) | CPA1 |
| FERULIC ACID (CIS) | EGFR |
| FERULIC ACID (CIS) | SLC16A1 |
| FERULIC ACID (CIS) | PTGS2 |
| FERULIC ACID (CIS) | KDM4E |
| FERULIC ACID (CIS) | KDM3A |
| FERULIC ACID (CIS) | KDM6B |
| FERULIC ACID (CIS) | FTO |
| FERULIC ACID (CIS) | KDM4A |
| FERULIC ACID (CIS) | KDM4C |
| FERULIC ACID (CIS) | TUBB1 |
| FERULIC ACID (CIS) | RELA |
| FERULIC ACID (CIS) | FYN |
| FERULIC ACID (CIS) | LCK |
| FERULIC ACID (CIS) | TLR9 |
| FERULIC ACID (CIS) | AKR1B10 |
| FERULIC ACID (CIS) | ALOX15 |
| FERULIC ACID (CIS) | PRKCE |
| FERULIC ACID (CIS) | F3 |
| FERULIC ACID (CIS) | NOS2 |
| FERULIC ACID (CIS) | NGFR |
| FERULIC ACID (CIS) | CCND1 CDK4 |
| FERULIC ACID (CIS) | TUBB3 |
| FERULIC ACID (CIS) | ABCB1 |
| FERULIC ACID (CIS) | ACE |
| FERULIC ACID (CIS) | REN |
| FERULIC ACID (CIS) | FBP1 |
| FERULIC ACID (CIS) | TOP2A |
| FERULIC ACID (CIS) | SLC13A5 |
| FERULIC ACID (CIS) | ECE1 |
| FERULIC ACID (CIS) | F2 |
| FERULIC ACID (CIS) | GLO1 |
| FERULIC ACID (CIS) | PARP1 |
| FERULIC ACID (CIS) | AHR |
| FERULIC ACID (CIS) | MAPK8 |
| FERULIC ACID (CIS) | MAOA |
| FERULIC ACID (CIS) | BACE1 |
| FERULIC ACID (CIS) | KDM2A |
| FERULIC ACID (CIS) | TPMT |
| FERULIC ACID (CIS) | ACLY |
| FERULIC ACID (CIS) | CTNNB1 |
| FERULIC ACID (CIS) | MME |
| Flemiphilippinin C | PTPN1 |
| Flemiphilippinin C | ABCG2 |
| Flemiphilippinin C | RARA |
| Flemiphilippinin C | F10 |
| Frutinone A | NFKB1 |
| Frutinone A | ACHE |
| Frutinone A | CA2 |
| Frutinone A | CA1 |
| Frutinone A | CA12 |
| Frutinone A | CA9 |
| Frutinone A | CA7 |
| Frutinone A | CA5B |
| Frutinone A | CA5A |
| Frutinone A | CA4 |
| Frutinone A | CA14 |
| Frutinone A | CA13 |
| Frutinone A | CA6 |
| Frutinone A | CA3 |
| Frutinone A | MAOA |
| Frutinone A | PDE10A |
| Frutinone A | PIK3CD PIK3R1 |
| Frutinone A | ADORA1 |
| Frutinone A | CDK2 |
| Frutinone A | CDK1 |
| Frutinone A | ESR2 |
| Frutinone A | AGPAT2 |
| Frutinone A | TRAP1 |
| Frutinone A | HSP90B1 |
| Frutinone A | CTSL |
| Frutinone A | PDE8B |
| Frutinone A | ESR1 |
| Frutinone A | HSP90AA1 |
| Frutinone A | ALPL |
| Frutinone A | MAPK8 |
| Frutinone A | MAPK10 |
| Frutinone A | MAPK9 |
| Frutinone A | CRHR1 |
| Gallic acid | CA2 |
| Gallic acid | CA7 |
| Gallic acid | CA1 |
| Gallic acid | CA3 |
| Gallic acid | CA6 |
| Gallic acid | CA12 |
| Gallic acid | CA14 |
| Gallic acid | CA9 |
| Gallic acid | FUT7 |
| Gallic acid | CA4 |
| Gallic acid | CA5B |
| Gallic acid | CA5A |
| Gallic acid | CA13 |
| Gallic acid | SQLE |
| Gallic acid | LDHA |
| Gallic acid | LDHB |
| Gallic acid | TTR |
| Gallic acid | IGF1R |
| Gallic acid | ALK |
| Gallic acid | SERPINE1 |
| Gallic acid | ESR2 |
| Gallic acid | BCL2L1 |
| Gallic acid | GPR35 |
| Gallic acid | COMT |
| Gallic acid | TPMT |
| gallicin | CYP19A1 |
| gallicin | HMGCR |
| gallicin | PPARG |
| gallicin | AR |
| gallicin | PGR |
| gallicin | PDE4D |
| gallicin | HSD11B1 |
| gallicin | CDC25A |
| gallicin | FNTA FNTB |
| gallicin | PTGS2 |
| gallicin | IL1B |
| gallicin | PRKCA |
| gallicin | PRKCD |
| gallicin | PTPN1 |
| gallicin | JUN |
| gallicin | HSD17B2 |
| gallicin | NR3C2 |
| gallicin | ATP12A |
| gallicin | TBXA2R |
| gallicin | PRSS1 |
| gallicin | CTRC |
| gallicin | CCNB3 CDK1 CCNB1 CCNB2 |
| gallicin | CDK1 |
| gallicin | PPARA |
| gallicin | PPARD |
| gallicin | PTGES |
| gallicin | PYGL |
| gallicin | SF3B3 |
| gallicin | UGT2B7 |
| gallicin | CDC25B |
| gallicin | PTPN2 |
| gallicin | HSD11B2 |
| gallicin | PPP2CA |
| gallicin | PCSK7 |
| gallicin | APP |
| gallicin | TRPV3 |
| gallicin | PARP1 |
| gallicin | HMOX1 |
| gallicin | LIPA |
| gallicin | HSD17B3 |
| gallicin | RASGRP1 |
| gallicin | MMP1 |
| gallicin | SRD5A1 |
| gallicin | PRMT3 |
| gallicin | PSEN2 PSENEN NCSTN APH1A PSEN1 APH1B |
| gallicin | JAK3 |
| gallicin | JAK1 |
| gallicin | JAK2 |
| gallicin | TYK2 |
| gallicin | GSK3B |
| gallicin | ADORA3 |
| gallicin | MAPK3 |
| gallicin | PRKCE |
| gallicin | CDC25C |
| gallicin | CDK5R1 CDK5 |
| gallicin | PIM1 |
| gallicin | PIM3 |
| gallicin | ITGAL |
| gallicin | TYMS |
| gallicin | LTA4H |
| isoetin | NOX4 |
| isoetin | AKR1B1 |
| isoetin | XDH |
| isoetin | MAOA |
| isoetin | FLT3 |
| isoetin | CA2 |
| isoetin | ALOX5 |
| isoetin | ADORA1 |
| isoetin | CA7 |
| isoetin | GLO1 |
| isoetin | GSK3B |
| isoetin | MMP9 |
| isoetin | CA12 |
| isoetin | MMP2 |
| isoetin | CA4 |
| isoetin | CYP1B1 |
| isoetin | ABCG2 |
| isoetin | AVPR2 |
| isoetin | IGF1R |
| isoetin | CYP19A1 |
| isoetin | EGFR |
| isoetin | F2 |
| isoetin | PIM1 |
| isoetin | AURKB |
| isoetin | DRD4 |
| isoetin | MPO |
| isoetin | PIK3R1 |
| isoetin | ADORA2A |
| isoetin | DAPK1 |
| isoetin | PYGL |
| isoetin | CA1 |
| isoetin | SRC |
| isoetin | PTK2 |
| isoetin | HSD17B2 |
| isoetin | KDR |
| isoetin | MMP13 |
| isoetin | MMP3 |
| isoetin | CA3 |
| isoetin | ALOX15 |
| isoetin | ABCC1 |
| isoetin | PLK1 |
| isoetin | CA6 |
| isoetin | CDK1 |
| isoetin | PKN1 |
| isoetin | CA14 |
| isoetin | CA9 |
| isoetin | CSNK2A1 |
| isoetin | ALOX12 |
| isoetin | MET |
| isoetin | NEK2 |
| isoetin | CXCR1 |
| isoetin | CAMK2B |
| isoetin | ALK |
| isoetin | AKT1 |
| isoetin | ABCB1 |
| isoetin | NEK6 |
| isoetin | PLA2G1B |
| isoetin | CA5A |
| isoetin | BACE1 |
| isoetin | AXL |
| isoetin | NUAK1 |
| isoetin | AKR1C2 |
| isoetin | AKR1C1 |
| isoetin | AKR1C3 |
| isoetin | AKR1C4 |
| isoetin | CA13 |
| isoetin | AKR1A1 |
| isoetin | PTPRS |
| isoetin | GPR35 |
| isoetin | ESR2 |
| isoetin | MPG |
| isoetin | SLC22A12 |
| isoetin | SYK |
| isoetin | MAPT |
| isoetin | KDM4E |
| isoetin | TOP2A |
| isoetin | INSR |
| isoetin | ACHE |
| isoetin | MYLK |
| isoetin | PIK3CG |
| isoetin | APEX1 |
| isoetin | CDK5R1 CDK5 |
| isoetin | CCNB3 CDK1 CCNB1 CCNB2 |
| isoetin | APP |
| isoetin | PARP1 |
| isoetin | TTR |
| isoetin | MMP12 |
| isoetin | CD38 |
| isoetin | AKR1B10 |
| isoetin | TNKS2 |
| isoetin | TNKS |
| isoetin | TOP1 |
| isoetin | ARG1 |
| isoetin | CDK6 |
| isoetin | CDK2 |
| isoetin | ESR1 |
| isoetin | HSD17B1 |
| isoetin | TYR |
| isoetin | AHR |
| isoetin | ESRRA |
| isoimperatorin | BACE1 |
| isoimperatorin | KCNA3 |
| isoimperatorin | SRD5A1 |
| isoimperatorin | CA12 |
| isoimperatorin | CA9 |
| isoimperatorin | KCNA5 |
| isoimperatorin | MAOA |
| isoimperatorin | ALOX5 |
| isoimperatorin | MAOB |
| isoimperatorin | ALOX15 |
| isoimperatorin | CD38 |
| isoimperatorin | CA7 |
| isoimperatorin | CA14 |
| isoimperatorin | CA13 |
| isoimperatorin | CA5B |
| isoimperatorin | OPRK1 |
| isoimperatorin | CBR1 |
| isoimperatorin | PIK3CD |
| isoimperatorin | PIK3CB |
| isoimperatorin | PIK3CG |
| isoimperatorin | PIK3CA |
| isoimperatorin | PDE7A |
| isoimperatorin | MTNR1A |
| isoimperatorin | CA6 |
| isoimperatorin | PTGES |
| isoimperatorin | METAP2 |
| isoimperatorin | HSD11B1 |
| isoimperatorin | PDE10A |
| isoimperatorin | LIMK1 |
| isoimperatorin | CCR9 |
| isoimperatorin | PARP1 |
| isoimperatorin | P2RX7 |
| isoimperatorin | PARP2 |
| isoimperatorin | DNMT3A |
| isoimperatorin | CDK1 CCNB1 |
| isoimperatorin | HMGCR |
| isoimperatorin | MTOR |
| isoimperatorin | HPGDS |
| isoimperatorin | CA1 |
| isoimperatorin | ADORA2B |
| isoimperatorin | CA4 |
| isoimperatorin | AKR1C1 |
| isoimperatorin | ATM |
| isoimperatorin | PDE8B |
| isoimperatorin | AKR1C3 |
| isoimperatorin | BRD4 |
| isoimperatorin | FLT3 |
| isoimperatorin | JAK2 |
| ledebouriellol | PLAU |
| ledebouriellol | ALOX15 |
| ledebouriellol | HSP90AA1 |
| ledebouriellol | CCR4 |
| methyl gallate | FUT7 |
| methyl gallate | CA2 |
| methyl gallate | CA7 |
| methyl gallate | CA1 |
| methyl gallate | CA12 |
| methyl gallate | CA14 |
| methyl gallate | CA9 |
| methyl gallate | CA3 |
| methyl gallate | CA6 |
| methyl gallate | CA4 |
| methyl gallate | CA5B |
| methyl gallate | CA5A |
| methyl gallate | CA13 |
| methyl gallate | SQLE |
| methyl gallate | SERPINE1 |
| methyl gallate | IGF1R |
| methyl gallate | ALK |
| methyl gallate | ESR2 |
| methyl gallate | TYR |
| methyl gallate | BCL2L1 |
| methyl gallate | AURKB |
| methyl gallate | SRC |
| methyl gallate | PTK2 |
| methyl gallate | KDR |
| methyl gallate | MET |
| methyl gallate | NEK2 |
| methyl gallate | AXL |
| methyl gallate | CNR2 |
| methyl gallate | TTR |
| methyl gallate | POLA1 |
| methyl gallate | POLB |
| Phellopterin | BACE1 |
| Phellopterin | MAOA |
| Phellopterin | PIK3CA |
| Phellopterin | DRD4 |
| Phellopterin | GABRB3 GABRA3 GABRG2 |
| Phellopterin | GABRB3 GABRG2 GABRA1 |
| Phellopterin | GABRB3 GABRG2 GABRA5 |
| Phellopterin | GABRA2 GABRB3 GABRG2 |
| Phellopterin | MAPK14 |
| Phellopterin | CDK1 CCNB1 |
| Phellopterin | FLT3 |
| Phellopterin | ADORA2A |
| Phellopterin | JAK1 |
| Phellopterin | JAK2 |
| Phellopterin | TYK2 |
| Phellopterin | CYP19A1 |
| Phellopterin | NUDT1 |
| Phellopterin | MCHR1 |
| Phellopterin | PIK3CB |
| Phellopterin | PIK3CG |
| Phellopterin | MTOR |
| Phellopterin | PIK3CD |
| Phellopterin | PRKDC |
| Phellopterin | CCNE2 CDK2 CCNE1 |
| Phellopterin | DUT |
| Phellopterin | MAOB |
| Phellopterin | PDE8B |
| Phellopterin | GYS1 |
| Phellopterin | SRD5A1 |
| Phellopterin | KCNA3 |
| Phellopterin | ADORA2B |
| Phellopterin | PDE10A |
| Phellopterin | TAAR1 |
| Phellopterin | CA9 |
| Phellopterin | PDE5A |
| Phellopterin | HCAR2 |
| Phellopterin | HSF1 |
| Phellopterin | CYP1A2 |
| Phellopterin | SLC9A1 |
| Phellopterin | CA2 |
| Phellopterin | CA1 |
| Phellopterin | KCNH2 |
| Phellopterin | PDPK1 |
| Phellopterin | NAAA |
| Phellopterin | DNMT3A |
| Phellopterin | CCND3 CCND1 CDK4 CCND2 |
| Phellopterin | EDNRA |
| Phellopterin | RPS6KB1 |
| Phellopterin | PTGES |
| Phellopterin | KCNA5 |
| Phellopterin | ALPL |
| Phellopterin | PARP1 |
| Phellopterin | PLA2G7 |
| Phellopterin | MAP2K1 |
| Phellopterin | JAK3 |
| Phellopterin | CD38 |
| Phellopterin | APP |
| Phellopterin | CCKBR |
| Phellopterin | CDC7 |
| Phellopterin | HRH3 |
| Phellopterin | PTGER3 |
| Phellopterin | HRH4 |
| Phellopterin | HSP90AA1 |
| Phellopterin | PIM1 |
| Phellopterin | MAPK8 |
| Phellopterin | CRHR1 |
| Phellopterin | IMPDH2 |
| Phellopterin | PDE2A |
| Phellopterin | DYRK1A |
| Phellopterin | CLK4 |
| Phellopterin | ACHE |
| phelloptorin | BACE1 |
| phelloptorin | MAOA |
| phelloptorin | PIK3CA |
| phelloptorin | DRD4 |
| phelloptorin | GABRB3 GABRA3 GABRG2 |
| phelloptorin | GABRB3 GABRG2 GABRA1 |
| phelloptorin | GABRB3 GABRG2 GABRA5 |
| phelloptorin | GABRA2 GABRB3 GABRG2 |
| phelloptorin | MAPK14 |
| phelloptorin | CDK1 CCNB1 |
| phelloptorin | FLT3 |
| phelloptorin | ADORA2A |
| phelloptorin | JAK1 |
| phelloptorin | JAK2 |
| phelloptorin | TYK2 |
| phelloptorin | CYP19A1 |
| phelloptorin | NUDT1 |
| phelloptorin | MCHR1 |
| phelloptorin | PIK3CB |
| phelloptorin | PIK3CG |
| phelloptorin | MTOR |
| phelloptorin | PIK3CD |
| phelloptorin | PRKDC |
| phelloptorin | CCNE2 CDK2 CCNE1 |
| phelloptorin | DUT |
| phelloptorin | MAOB |
| phelloptorin | PDE8B |
| phelloptorin | GYS1 |
| phelloptorin | SRD5A1 |
| phelloptorin | KCNA3 |
| phelloptorin | ADORA2B |
| phelloptorin | PDE10A |
| phelloptorin | TAAR1 |
| phelloptorin | CA9 |
| phelloptorin | PDE5A |
| phelloptorin | HCAR2 |
| phelloptorin | HSF1 |
| phelloptorin | CYP1A2 |
| phelloptorin | SLC9A1 |
| phelloptorin | CA2 |
| phelloptorin | CA1 |
| phelloptorin | KCNH2 |
| phelloptorin | PDPK1 |
| phelloptorin | NAAA |
| phelloptorin | DNMT3A |
| phelloptorin | CCND3 CCND1 CDK4 CCND2 |
| phelloptorin | EDNRA |
| phelloptorin | RPS6KB1 |
| phelloptorin | PTGES |
| phelloptorin | KCNA5 |
| phelloptorin | ALPL |
| phelloptorin | PARP1 |
| phelloptorin | PLA2G7 |
| phelloptorin | MAP2K1 |
| phelloptorin | JAK3 |
| phelloptorin | CD38 |
| phelloptorin | APP |
| phelloptorin | CCKBR |
| phelloptorin | CDC7 |
| phelloptorin | HRH3 |
| phelloptorin | PTGER3 |
| phelloptorin | HRH4 |
| phelloptorin | HSP90AA1 |
| phelloptorin | PIM1 |
| phelloptorin | MAPK8 |
| phelloptorin | CRHR1 |
| phelloptorin | IMPDH2 |
| phelloptorin | PDE2A |
| phelloptorin | DYRK1A |
| phelloptorin | CLK4 |
| phelloptorin | ACHE |
| Prangenidin | SLC6A4 |
| Prangenidin | SLC6A2 |
| Prangenidin | SLC6A3 |
| Prangenidin | CA9 |
| Prangenidin | PIK3CG |
| Prangenidin | PIK3CA |
| Prangenidin | HDAC1 |
| Prangenidin | EDNRA |
| Prangenidin | MDM2 |
| Prangenidin | SRD5A1 |
| Prangenidin | HDAC6 |
| Prangenidin | HDAC2 |
| Prangenidin | CHRM4 |
| Prangenidin | ADRA2C |
| Prangenidin | CHRM5 |
| Prangenidin | CHRM2 |
| Prangenidin | OPRM1 |
| Prangenidin | OPRK1 |
| Prangenidin | CHRM3 |
| Prangenidin | SIGMAR1 |
| Prangenidin | DRD2 |
| Prangenidin | DRD4 |
| Prangenidin | DRD3 |
| Prangenidin | HDAC8 |
| Prangenidin | HSP90AB1 |
| Prangenidin | TNKS2 |
| Prangenidin | TNKS |
| Prangenidin | MMP9 |
| Prangenidin | MMP1 |
| Prangenidin | MMP2 |
| Prangenidin | RPS6KA3 |
| Prangenidin | MMP3 |
| quercetin-3',4',7-trimethyl ether | AKR1B1 |
| quercetin-3',4',7-trimethyl ether | CA2 |
| quercetin-3',4',7-trimethyl ether | CA12 |
| quercetin-3',4',7-trimethyl ether | XDH |
| quercetin-3',4',7-trimethyl ether | CA4 |
| quercetin-3',4',7-trimethyl ether | NOX4 |
| quercetin-3',4',7-trimethyl ether | CYP1B1 |
| quercetin-3',4',7-trimethyl ether | ABCG2 |
| quercetin-3',4',7-trimethyl ether | ABCB1 |
| quercetin-3',4',7-trimethyl ether | ADORA3 |
| quercetin-3',4',7-trimethyl ether | CDK1 |
| quercetin-3',4',7-trimethyl ether | OPRD1 |
| quercetin-3',4',7-trimethyl ether | CA7 |
| quercetin-3',4',7-trimethyl ether | PLG |
| quercetin-3',4',7-trimethyl ether | BACE1 |
| quercetin-3',4',7-trimethyl ether | PIK3CG |
| quercetin-3',4',7-trimethyl ether | MAPT |
| quercetin-3',4',7-trimethyl ether | KDM4E |
| quercetin-3',4',7-trimethyl ether | TOP2A |
| quercetin-3',4',7-trimethyl ether | ACHE |
| quercetin-3',4',7-trimethyl ether | MYLK |
| quercetin-3',4',7-trimethyl ether | MPO |
| quercetin-3',4',7-trimethyl ether | PIK3R1 |
| quercetin-3',4',7-trimethyl ether | DAPK1 |
| quercetin-3',4',7-trimethyl ether | PYGL |
| quercetin-3',4',7-trimethyl ether | SYK |
| quercetin-3',4',7-trimethyl ether | MMP3 |
| quercetin-3',4',7-trimethyl ether | CA3 |
| quercetin-3',4',7-trimethyl ether | PLK1 |
| quercetin-3',4',7-trimethyl ether | CSNK2A1 |
| quercetin-3',4',7-trimethyl ether | NEK2 |
| quercetin-3',4',7-trimethyl ether | NEK6 |
| quercetin-3',4',7-trimethyl ether | PLA2G1B |
| quercetin-3',4',7-trimethyl ether | CA5A |
| quercetin-3',4',7-trimethyl ether | APEX1 |
| quercetin-3',4',7-trimethyl ether | AKR1C2 |
| quercetin-3',4',7-trimethyl ether | AKR1C1 |
| quercetin-3',4',7-trimethyl ether | AKR1C3 |
| quercetin-3',4',7-trimethyl ether | AKR1C4 |
| quercetin-3',4',7-trimethyl ether | CA13 |
| quercetin-3',4',7-trimethyl ether | AKR1A1 |
| quercetin-3',4',7-trimethyl ether | CYP19A1 |
| quercetin-3',4',7-trimethyl ether | CA1 |
| quercetin-3',4',7-trimethyl ether | CA9 |
| quercetin-3',4',7-trimethyl ether | ALOX15 |
| quercetin-3',4',7-trimethyl ether | GSK3B |
| quercetin-3',4',7-trimethyl ether | AKT1 |
| quercetin-3',4',7-trimethyl ether | ADORA2A |
| quercetin-3',4',7-trimethyl ether | ALOX12 |
| quercetin-3',4',7-trimethyl ether | CA6 |
| quercetin-3',4',7-trimethyl ether | ARG1 |
| quercetin-3',4',7-trimethyl ether | DRD4 |
| quercetin-3',4',7-trimethyl ether | FLT3 |
| quercetin-3',4',7-trimethyl ether | EGFR |
| quercetin-3',4',7-trimethyl ether | F2 |
| quercetin-3',4',7-trimethyl ether | PIM1 |
| quercetin-3',4',7-trimethyl ether | PKN1 |
| quercetin-3',4',7-trimethyl ether | CAMK2B |
| quercetin-3',4',7-trimethyl ether | ALK |
| quercetin-3',4',7-trimethyl ether | AXL |
| quercetin-3',4',7-trimethyl ether | ALOX5 |
| quercetin-3',4',7-trimethyl ether | ADORA1 |
| quercetin-3',4',7-trimethyl ether | HSD17B2 |
| quercetin-3',4',7-trimethyl ether | CDK6 |
| quercetin-3',4',7-trimethyl ether | CDK2 |
| quercetin-3',4',7-trimethyl ether | TYR |
| quercetin-3',4',7-trimethyl ether | AHR |
| quercetin-3',4',7-trimethyl ether | ESRRA |
| quercetin-3',4',7-trimethyl ether | CDK5R1 CDK5 |
| quercetin-3',4',7-trimethyl ether | KIT |
| quercetin-3',4',7-trimethyl ether | MAOA |
| quercetin-3',4',7-trimethyl ether | MMP9 |
| quercetin-3',4',7-trimethyl ether | MMP2 |
| quercetin-3',4',7-trimethyl ether | PLA2G2A |
| quercetin-3',4',7-trimethyl ether | MCL1 |
| quercetin-3',4',7-trimethyl ether | CCNB3 CDK1 CCNB1 CCNB2 |
| quercetin-3',4',7-trimethyl ether | ABCC1 |
| quercetin-3',4',7-trimethyl ether | TERT |
| quercetin-3',4',7-trimethyl ether | GPR35 |
| quercetin-3',4',7-trimethyl ether | AVPR2 |
| quercetin-3',4',7-trimethyl ether | CXCR1 |
| quercetin-3',4',7-trimethyl ether | ST6GAL1 |
| quercetin-3',4',7-trimethyl ether | CA14 |
| quercetin-3',4',7-trimethyl ether | MET |
| quercetin-3',4',7-trimethyl ether | SRC |
| quercetin-3',4',7-trimethyl ether | PTPRS |
| quercetin-3',4',7-trimethyl ether | MPG |
| quercetin-3',4',7-trimethyl ether | PARP1 |
| quercetin-3',4',7-trimethyl ether | TTR |
| quercetin-3',4',7-trimethyl ether | MMP12 |
| quercetin-3',4',7-trimethyl ether | CD38 |
| quercetin-3',4',7-trimethyl ether | AKR1B10 |
| quercetin-3',4',7-trimethyl ether | TNKS2 |
| quercetin-3',4',7-trimethyl ether | TNKS |
| quercetin-3',4',7-trimethyl ether | TOP1 |
| quercetin-3',4',7-trimethyl ether | IGF1R |
| quercetin-3',4',7-trimethyl ether | INSR |
| quercetin-3',4',7-trimethyl ether | AURKB |
| quercetin-3',4',7-trimethyl ether | PTK2 |
| quercetin-3',4',7-trimethyl ether | KDR |
| rufescidride | PDE5A |
| rufescidride | TYR |
| rufescidride | GSK3B |
| rufescidride | DYRK1A |
| rufescidride | MMP9 |
| rufescidride | MMP1 |
| rufescidride | MMP2 |
| rufescidride | NOX4 |
| rufescidride | TOP1 |
| rufescidride | TDP1 |
| rufescidride | YWHAG |
| rufescidride | KDM1A |
| rufescidride | CHRNA7 |
| rufescidride | ALOX5 |
| rufescidride | GRK2 |
| taraxacin | F2 |
| taraxacin | PRSS1 |
| taraxacin | MAOA |
| taraxacin | MAOB |
| taraxacin | PREP |
| taraxacin | HDAC6 |
| taraxacin | HDAC1 |
| taraxacin | EPHX2 |
| taraxacin | P2RX7 |
| taraxacin | ACE |
| taraxacin | MTNR1A |
| taraxacin | CTSK |
| taraxacin | SRD5A1 |
| taraxacin | CHRM4 |
| taraxacin | CHRM5 |
| taraxacin | CHRM3 |
| taraxacin | SLC6A4 |
| taraxacin | CACNA1B |
| taraxacin | DRD4 |
| taraxacin | SIGMAR1 |
| taraxacin | GRM5 |
| taraxacin | CTSS |
| taraxacin | CTSB |
| taraxacin | MTNR1B |
| taraxacin | CYP11B1 |
| taraxacin | GABRB3 GABRG2 GABRA5 |
| taraxacin | CYP11B2 |
| taraxacin | NQO2 |
| taraxacin | PARP1 |
| taraxacin | CTSC |
| taraxacin | CYP19A1 |
| taraxacin | ADORA2A |
| taraxacin | ADORA3 |
| taraxacin | PSEN2 PSENEN NCSTN APH1A PSEN1 APH1B |
| taraxacin | SIRT2 |
| taraxacin | FABP4 |
| taraxacin | HMOX1 |
| taraxacin | FABP3 |
| taraxacin | BRD4 |
| taraxacin | HTR2C |
| taraxacin | CTSL |
| taraxacin | FAP |
| taraxacin | PARP2 |
| taraxacin | HTR2B |
| taraxacin | MAPK14 |
| taraxacin | GPR139 |
| taraxacin | PTGS1 |
| taraxacin | MGLL |
| taraxacin | PSMB5 |
| taraxacin | HCRTR2 |
| taraxacin | TGM2 |
| taraxacin | MALT1 |
| taraxacin | GABRB3 GABRA3 GABRG2 |
| taraxacin | GABRB3 GABRG2 GABRA1 |
| taraxacin | CCNE2 CDK2 CCNE1 |
| taraxacin | CCNB3 CDK1 CCNB1 CCNB2 |
| taraxacin | GABRA2 GABRB3 GABRG2 |
| taraxacin | GABRG2 GABRB3 GABRA6 |
| taraxacin | CDK7 CCNH |
| taraxacin | DYRK1A |
| taraxacin | SCN10A |
| taraxacin | DYRK1B |
| taraxacin | HDAC3 |
| taraxacin | CSF1R |
| taraxacin | MMP13 |
| taraxacin | MMP8 |
| taraxacin | MCL1 |
| taraxacin | TRPV1 |
| taraxacin | ADH1A |
| taraxacin | ADH1B |
| taraxacin | ADH7 |
| taraxacin | NAMPT |
| taraxacin | HTR2A |
| taraxacin | CYP3A4 |
| taraxacin | DYRK2 |
| taraxacin | DHODH |
| wogonin | PTGS2 |
| wogonin | NOS2 |
| wogonin | FLT3 |
| wogonin | AKR1B1 |
| wogonin | OPRD1 |
| wogonin | KIT |
| wogonin | ABCB1 |
| wogonin | IKBKB |
| wogonin | NTRK2 |
| wogonin | KDM4E |
| wogonin | XDH |
| wogonin | ALOX15 |
| wogonin | CDK1 |
| wogonin | ALOX12 |
| wogonin | GRK6 |
| wogonin | CYP19A1 |
| wogonin | ESR2 |
| wogonin | CYP1A1 |
| wogonin | OPRM1 |
| wogonin | ABCG2 |
| wogonin | ESR1 |
| wogonin | EGFR |
| wogonin | SLC22A12 |
| wogonin | CYP1B1 |
| wogonin | CA4 |
| wogonin | CA2 |
| wogonin | CA1 |
| wogonin | MCL1 |
| wogonin | PIK3CG |
| wogonin | PIM1 |
| wogonin | ADORA1 |
| wogonin | ADORA2A |
| wogonin | HSD17B2 |
| wogonin | HSD17B1 |
| wogonin | CA7 |
| wogonin | CA12 |
| wogonin | CDK5R1 CDK5 |
| wogonin | CCNB3 CDK1 CCNB1 CCNB2 |
| wogonin | CDK6 |
| wogonin | CA9 |
| wogonin | CBR1 |
| wogonin | TERT |
| wogonin | AR |
| wogonin | CA6 |
| wogonin | PTPRS |
| wogonin | DAPK1 |
| wogonin | MPG |
| wogonin | PFKFB3 |
| wogonin | MMP9 |
| wogonin | MMP2 |
| wogonin | LCK |
| wogonin | MMP12 |
| wogonin | CD38 |
| wogonin | TOP1 |
| wogonin | ARG1 |
| wogonin | MAPT |
| wogonin | TOP2A |
| wogonin | INSR |
| wogonin | DRD4 |
| wogonin | MYLK |
| wogonin | MPO |
| wogonin | PIK3R1 |
| wogonin | PYGL |
| wogonin | MMP13 |
| wogonin | MMP3 |
| wogonin | CA3 |
| wogonin | CA14 |
| wogonin | MET |
| wogonin | CA13 |
| wogonin | CAMK2B |
| wogonin | PLA2G1B |
| wogonin | CA5A |
| wogonin | APEX1 |
| wogonin | AKR1C2 |
| wogonin | AKR1C1 |
| wogonin | AKR1C3 |
| wogonin | AKR1C4 |
| wogonin | AKR1A1 |
| wogonin | GPR35 |
| wogonin | ODC1 |
| wogonin | HSP90AA1 |
| wogonin | ALOX5 |
| wogonin | CXCR1 |
| wogonin | PLA2G2A |
| wogonin | SRC |
| wogonin | APP |
| wogonin | CYP1A2 |
| wogonin | ADORA3 |
| wogonin | HSP90B1 |
| wogonin | ABCC1 |
| wogonin | TNKS |
| wogonin | TTR |
| wogonin | NOX4 |
| wogonin | AVPR2 |
| wogonin | NAE1 |
| wogonin | BCHE |
| wogonin | ACHE |
| wogonin | BACE1 |
| wogonin | PDE5A |
| wogonin | GSK3B |
