# Supplementary material for: Immunomodulatory Mechanism of Baiyaojian Decoction on Periodontitis: Network Pharmacology, Single‐Cell RNA Sequencing and Molecular Docking
Source: J Cell Mol Med. 2026 Jan 28;30(3):e71034. doi: 10.1111/jcmm.71034 (PMC12851902; doi:10.1111/jcmm.71034)
Supplement: Supplementary file 4 — Table S3: Periodontitis‐related pathogenic genes in disease database. [file JCMM-30-e71034-s004.docx]

**Supplementary Table 3 Periodontitis-related pathogenic genes in disease database**

| GeneCards | CTD | MalaCards | DrugBank | OMIM |
| --- | --- | --- | --- | --- |
| CTSC | ATF4 | CDKN2B-AS1 | ALB | C1R |
| LOC130006572 | CDKN2A | ENSG00000226079 | ANXA3 | GLT6D1 |
| IL1B | FTH1 | CTSC | NR3C1 | CDKN2BAS |
| FPR1 | MET | IL1B | CYP3A4 | NOD1 |
| C1R | E2F1 | IL6 | CYP3A5 | CTSC |
| C1S | KRAS | TNF | CYP2C9 | IL18 |
| IL1A | CYP3A5 | TNFSF11 | CYP2C8 | ITGB2 |
| PDON2 | GPX2 | MMP8 | UGT1A1 | PRTN3 |
| IL6 | ATM | IL1A | SELPLG | LTF |
| TNF | HSPH1 | MIR146A | CYP2A6 | IL1A |
| C1RL | IDH2 | CXCL8 | CYP3A7 | ESR1 |
| MMP8 | CCNE2 | IL10 | IL1B |  |
| TNFSF11 | RAD51 | TNFRSF11B | ALOX5 |  |
| CXCL8 | SLC6A3 | TLR4 | MMP9 |  |
| MMP1 | ALAD | MIR21 | VEGFA |  |
| IL10 | HERPUD1 | TLR2 | CASP1 |  |
| TNFRSF11B | OGG1 | IL17A | CASP3 |  |
| CDKN2B-AS1 | PTGR1 | MIR155 | CYCS |  |
| TLR4 | NFIL3 | CRP | SLC22A7 |  |
| ELANE | UBE2C | MMP9 | MAPK |  |
| ENSG00000226079 | ABCB1B | MIR17 | NOS2 |  |
| IL17A | CHEK2 | IL4 | CYP1A2 |  |
| IL4 | FADS2 | MIR142 |  |  |
| MMP9 | HDAC1 | IFNG |  |  |
| TLR2 | ENO2 | MIR125A |  |  |
| FCGR3B | HAMP | PDON2 |  |  |
| TGFB1 | HSPA1B | MMP1 |  |  |
| BGLAP | KRT19 | TGFB1 |  |  |
| IFNG | LMNB1 | MIR140 |  |  |
| LTF | MCM2 | MIR214 |  |  |
| IL1RN | CREM | BGLAP |  |  |
| MMP2 | MAP2K6 | LTF |  |  |
| CD14 | BAG3 | MIR144 |  |  |
| FCGR2A | CDC6 | CCL2 |  |  |
| CRP | MCM5 | MIR1226 |  |  |
| ITGB2 | POU5F1 | MMP3 |  |  |
| TIMP1 | SOX9 | MMP2 |  |  |
| RUNX2 | SYP | IL18 |  |  |
| VDR | ADORA1 | RUNX2 |  |  |
| MPO | CCR2 | PTGS2 |  |  |
| FCGR3A | CDC20 | ACP5 |  |  |
| DSPP | CDKN3 | TIMP1 |  |  |
| CCL2 | CENPF | MIR200B |  |  |
| CTSK | KIF20A | ELANE |  |  |
| IBSP | NR4A3 | IL1RN |  |  |
| IL18 | PIM1 | MMP13 |  |  |
| DEFB1 | ALDOC | IL2 |  |  |
| BMP2 | NCOA2 | DEFB1 |  |  |
| ASPN | PBK | FCGR3B |  |  |
| CTSG | CDC25A | GLT6D1 |  |  |
| PTGS2 | CSF1R | CTSK |  |  |
| POSTN | FOXM1 | ICAM1 |  |  |
| MMP13 | GABRA1 | DEFB4A |  |  |
| MMP3 | MCM3 | MPO |  |  |
| IL2 | PTX3 | MIR671 |  |  |
| CD36 | BMAL1 | MIR27A |  |  |
| GLT6D1 | DAPK1 |  |  |  |
| HLA-B | UHRF1 |  |  |  |
| COL1A1 | CKS2 |  |  |  |
| NOD2 | DIABLO |  |  |  |
| MIR146A | DNMT3A |  |  |  |
| ICAM1 | EEF1A1 |  |  |  |
| SPP1 | EFNA1 |  |  |  |
| CCL5 | NGFR |  |  |  |
| HLA-DQB1 | SLC11A2 |  |  |  |
| IL13 | SULT1E1 |  |  |  |
| ALPP | UCHL1 |  |  |  |
| DEFB4A | DBP |  |  |  |
| HLA-DRB1 | DHCR7 |  |  |  |
| HLA-A | GJB2 |  |  |  |
| SERPINE1 | ITGA6 |  |  |  |
| COL3A1 | LAMP2 |  |  |  |
| COL17A1 | NUSAP1 |  |  |  |
| FERMT1 | SHBG |  |  |  |
| NFKB1 | TCF4 |  |  |  |
| CERNA3 | TKT |  |  |  |
| CCL3 | AXL |  |  |  |
| NOTCH2 | CYP26B1 |  |  |  |
| ACP5 | DAB2 |  |  |  |
| CAMP | IL6R |  |  |  |
| MIR155 | RET |  |  |  |
| CEMP1 | RRM1 |  |  |  |
| VEGFA | CHRM3 |  |  |  |
| CAT | EPAS1 |  |  |  |
| AMBN | MSH2 |  |  |  |
| MIR125A | NEK2 |  |  |  |
| LNCPOIR | SERPINH1 |  |  |  |
| DMP1 | ULK1 |  |  |  |
| SIRT1 | ALCAM |  |  |  |
| SLC17A5 | FEN1 |  |  |  |
| TGFB2 | HNRNPA1 |  |  |  |
| MIR21 | IL23A |  |  |  |
| CXCL10 | IL2RA |  |  |  |
| SP7 | ITGB4 |  |  |  |
| FN1 | KIF2C |  |  |  |
| FCAR | PML |  |  |  |
| HTN3 | RIPK3 |  |  |  |
| DPP7 | S100B |  |  |  |
| PLAT | ASS1 |  |  |  |
| TIMP2 | BNIP3L |  |  |  |
| GC | CDH5 |  |  |  |
| FBN2 | DPP4 |  |  |  |
| SPARC | GLB1 |  |  |  |
| ESR1 | GRIN2A |  |  |  |
| GUSB | GSTA5 |  |  |  |
| BRINP3 | KPNA2 |  |  |  |
| CCR5 | MT1X |  |  |  |
| H19 | PROS1 |  |  |  |
| DANCR | PTTG1 |  |  |  |
| MIR17 | SLC47A1 |  |  |  |
| HAX1 | SLC6A4 |  |  |  |
| CXCR4 | TUBB6 |  |  |  |
| CYBA | GNMT |  |  |  |
| WDR72 | MFN1 |  |  |  |
| MEG3 | MUC1 |  |  |  |
| SELE | PDE5A |  |  |  |
| SLC35C1 | STAT5A |  |  |  |
| PLG | TUBB4B |  |  |  |
| MIR214 | VASP |  |  |  |
| IGF1 | WARS1 |  |  |  |
| UNC50 | ALDH7A1 |  |  |  |
| MIR1226 | B2M |  |  |  |
| NLRP10 | BMP7 |  |  |  |
| FGF2 | FBLN1 |  |  |  |
| MIR140 | GREB1 |  |  |  |
| SELL | GSDMD |  |  |  |
| TUG1 | IRAK1 |  |  |  |
| ALB | NCOR1 |  |  |  |
| ADGRG6 | POSTN |  |  |  |
| FERMT3 | SIGMAR1 |  |  |  |
| NLRP3 | TOP1 |  |  |  |
| PPARG | AKR1A1 |  |  |  |
| TGFBR2 | BUB1B |  |  |  |
| CXCR2 | C1QB |  |  |  |
| TNXB | CCNA1 |  |  |  |
| SMAD3 | CIRBP |  |  |  |
| SOD2-OT1 | CRY1 |  |  |  |
| COL1A2 | CYP4A14 |  |  |  |
| STATH | DNMT3B |  |  |  |
| LINC02605 | EFEMP1 |  |  |  |
| FCGR2B | GRIA3 |  |  |  |
| SERPINB2 | LHCGR |  |  |  |
| CASP4 | MYB |  |  |  |
| MIR142 | PSMB8 |  |  |  |
| DCN | SLC5A5 |  |  |  |
| PADI4 | AKR1B8 |  |  |  |
| CASP3 | BSG |  |  |  |
| BDNF-AS | CARS1 |  |  |  |
| GFI1 | CISH |  |  |  |
| VCAM1 | FHL1 |  |  |  |
| ESR2 | GABRG2 |  |  |  |
| EMSLR | LGALS3BP |  |  |  |
| CSF1 | LIG1 |  |  |  |
| AMELX | LTF |  |  |  |
| CD79A | NHERF1 |  |  |  |
| LTA | NOTCH2 |  |  |  |
| BMP6 | NPR3 |  |  |  |
| BMP7 | PDCD4 |  |  |  |
| FGF10 | RACGAP1 |  |  |  |
| SLC24A4 | RAD50 |  |  |  |
| MMP12 | SMAD4 |  |  |  |
| LYST | AQP4 |  |  |  |
| AGER | BUB1 |  |  |  |
| MAPK1 | CPE |  |  |  |
| FBN1 | E2F2 |  |  |  |
| COL5A1 | ENPP1 |  |  |  |
| COL5A2 | GABRB2 |  |  |  |
| MIR671 | HNRNPK |  |  |  |
| CTLA4 | HYOU1 |  |  |  |
| MKLN1 | MGST2 |  |  |  |
| SLC23A1 | PDYN |  |  |  |
| TREM1 | PRKACA |  |  |  |
| IL33 | SHC1 |  |  |  |
| DLX3 | SHH |  |  |  |
| MIR200B | SMPD3 |  |  |  |
| FAM20A | TUBB5 |  |  |  |
| FAM83H | ZWINT |  |  |  |
| EGF | ADRB1 |  |  |  |
| HMGB1 | ATP7A |  |  |  |
| B4GALT7 | CDC45 |  |  |  |
| CXCL12 | CDCA8 |  |  |  |
| KLK4 | CTSH |  |  |  |
| ENAM | CYP2C29 |  |  |  |
| TNFRSF11A | DDX17 |  |  |  |
| LTBP3 | DDX21 |  |  |  |
| GPR68 | HSPA4L |  |  |  |
| IL11 | MBNL2 |  |  |  |
| ADIPOQ | MCM4 |  |  |  |
| IL1R1 | MVD |  |  |  |
| MIR144 | NCOA3 |  |  |  |
| HSPG2 | NCOR2 |  |  |  |
| HIF1A | NOLC1 |  |  |  |
| DEFB103B | OPRK1 |  |  |  |
| LINC01672 | PAWR |  |  |  |
| CTNNB1 | PENK |  |  |  |
| MMP20 | PSMB9 |  |  |  |
| ALPL | RTN4 |  |  |  |
| CSF2 | TNFRSF10A |  |  |  |
| NOS2 | TST |  |  |  |
| CCL21 | UBD |  |  |  |
| IL12B | C1QA |  |  |  |
| PRTN3 | CCR1 |  |  |  |
| FOS | CD24 |  |  |  |
| MBL2 | CDKN2B |  |  |  |
| ELN | FHL2 |  |  |  |
| CSF3 | GARS1 |  |  |  |
| TMX2-CTNND1 | GLI1 |  |  |  |
| HSPD1 | GSTT2 |  |  |  |
| OSM | HNRNPH1 |  |  |  |
| IFIH1 | HTR2C |  |  |  |
| CTSS | LIMA1 |  |  |  |
| BPI | NPC1 |  |  |  |
| ITGB6 | PCLAF |  |  |  |
| ITGAL | PDIA6 |  |  |  |
| MAPK14 | SEMA3C |  |  |  |
| S100A8 | SLC22A3 |  |  |  |
| CTSB | SNAP25 |  |  |  |
| PGR-AS1 | TXNRD2 |  |  |  |
| FMOD | AARS1 |  |  |  |
| ITGAM | AHRR |  |  |  |
| SSUH2 | ASPM |  |  |  |
| JUN | CDCA2 |  |  |  |
| RETN | ELK1 |  |  |  |
| NPY | FLNB |  |  |  |
| IL12A | GAMT |  |  |  |
| PLAU | GTSE1 |  |  |  |
| IL1RAPL2 | HMMR |  |  |  |
| NFATC1 | IFITM3 |  |  |  |
| XIST | MT1E |  |  |  |
| CD4 | NRCAM |  |  |  |
| LBP | OGT |  |  |  |
| SLC37A4 | PNP |  |  |  |
| IL17F | PRC1 |  |  |  |
| GSTM1 | RACK1 |  |  |  |
| FBLN5 | SMARCA4 |  |  |  |
| MIR223 | SULT1B1 |  |  |  |
| HMOX1 | TM7SF2 |  |  |  |
| BDNF | TUBB2B |  |  |  |
| MIR27A | YARS1 |  |  |  |
| ODAM | AKR1C14 |  |  |  |
| FGB | APOA2 |  |  |  |
| PLOD1 | ESPL1 |  |  |  |
| ADAMTS2 | HSPA4 |  |  |  |
| FKBP14 | IRF3 |  |  |  |
| SLC39A13 | KIF23 |  |  |  |
| ARHGAP6 | NCAPG |  |  |  |
| RELT | NR2F2 |  |  |  |
| PRDM5 | PF4 |  |  |  |
| SP6 | PRDX5 |  |  |  |
| ZNF469 | PRIM1 |  |  |  |
| LOC101448202 | PSMB10 |  |  |  |
| LOC106780803 | RBM3 |  |  |  |
| AZU1 | RRAS2 |  |  |  |
| DEFA1 | SCNN1A |  |  |  |
| CD40LG | TUBB |  |  |  |
| SOD1 | CCL9 |  |  |  |
| NAMPT | CTNNA1 |  |  |  |
| LPO | CYP3A7 |  |  |  |
| PTHLH | DDX5 |  |  |  |
| TERT | GDA |  |  |  |
| CYP1A1 | HBB |  |  |  |
| HGF | LY6E |  |  |  |
| AKT1 | PDPK1 |  |  |  |
| TP53 | PLS3 |  |  |  |
| ACE | RIGI |  |  |  |
| SLURP1 | SLC39A10 |  |  |  |
| IL23A | TNFRSF25 |  |  |  |
| CTSL | ZBTB20 |  |  |  |
| AMTN | BRD2 |  |  |  |
| SOST | BRD4 |  |  |  |
| JAG1 | CANX |  |  |  |
| LACTB | CEP55 |  |  |  |
| MMP14 | DBN1 |  |  |  |
| FGFR2 | EPHA4 |  |  |  |
| IL16 | ERP29 |  |  |  |
| GREM1 | FOXP3 |  |  |  |
| SLPI | GINS2 |  |  |  |
| MAPK8 | HDAC6 |  |  |  |
| CTSH | IDO1 |  |  |  |
| NOTCH3 | IL16 |  |  |  |
| MIR543 | INMT |  |  |  |
| LEP | MANF |  |  |  |
| CCL20 | MTUS1 |  |  |  |
| MIR146B | NCAPD2 |  |  |  |
| TNFRSF1B | OPRD1 |  |  |  |
| IL6R | PARP14 |  |  |  |
| MMP7 | PSMB5 |  |  |  |
| PTEN | PSMC5 |  |  |  |
| INS | ACTG1 |  |  |  |
| FGD5-AS1 | ALAS2 |  |  |  |
| HLA-C | ALDH18A1 |  |  |  |
| SFTA3 | BOK |  |  |  |
| P2RX7 | C4B |  |  |  |
| PIK3CG | CEBPG |  |  |  |
| F2RL1 | CENPE |  |  |  |
| S100A9 | COL5A2 |  |  |  |
| CST3 | DPYSL3 |  |  |  |
| BSG | HTRA1 |  |  |  |
| CMA1 | KLF10 |  |  |  |
| ALDH2 | LAMC1 |  |  |  |
| NCF1 | LRG1 |  |  |  |
| NCF4 | MIR21 |  |  |  |
| NCF4-AS1 | MLXIPL |  |  |  |
| LOC106029312 | MT1F |  |  |  |
| MTOR | NNMT |  |  |  |
| BCL2A1 | NPAS2 |  |  |  |
| COL4A1 | NRAS |  |  |  |
| FGF7 | PRDX4 |  |  |  |
| SNRPN | PSMB1 |  |  |  |
| SERPINA1 | RASSF1 |  |  |  |
| MIR628 | RFC4 |  |  |  |
| IL1R2 | SERPINB9 |  |  |  |
| KLK11 | SHCBP1 |  |  |  |
| CD40 | SPON2 |  |  |  |
| IGF1R | STARD4 |  |  |  |
| TLR9 | TFPI |  |  |  |
| IL34 | THY1 |  |  |  |
| NOS3 | TM4SF1 |  |  |  |
| ADAM17 | TPD52L1 |  |  |  |
| IL4R | TRAP1 |  |  |  |
| DPP4 | TRIM2 |  |  |  |
| HTN1 | ZFP36L2 |  |  |  |
| LGALS3 | AK1 |  |  |  |
| GDF15 | ALDH5A1 |  |  |  |
| PTGS1 | AMBP |  |  |  |
| MIR150 | ANGPT2 |  |  |  |
| CXCR1 | C1QC |  |  |  |
| S100A4 | CD40LG |  |  |  |
| AP3B1 | CD59 |  |  |  |
| FASLG | CD81 |  |  |  |
| YAP1 | CDCA5 |  |  |  |
| PTGER2 | DTL |  |  |  |
| GSTT1 | GPRC5A |  |  |  |
| TWIST1 | HTR1B |  |  |  |
| GPT | LRRK2 |  |  |  |
| PTK2 | NCOA7 |  |  |  |
| CCN2 | NEAT1 |  |  |  |
| PTGER3 | PNRC1 |  |  |  |
| SELP | POLD1 |  |  |  |
| IL7 | PPP1R3B |  |  |  |
| NFE2L2 | PSMA5 |  |  |  |
| NOTCH1 | RBL1 |  |  |  |
| FIZ1 | RRBP1 |  |  |  |
| HP | SLC19A2 |  |  |  |
| ITGAX | SMARCA2 |  |  |  |
| DKK1 | TBXAS1 |  |  |  |
| MAPK3 | TMPO |  |  |  |
| EGFR | YWHAG |  |  |  |
| TRA-TGC7-1 | ZYX |  |  |  |
| IGF2 | BHLHE41 |  |  |  |
| NPAP1 | CALB2 |  |  |  |
| WNT5A | CALD1 |  |  |  |
| BMP4 | CREG1 |  |  |  |
| TF | DLL1 |  |  |  |
| CALCA | DSC2 |  |  |  |
| BIRC5 | DUSP8 |  |  |  |
| EDIL3 | FANCI |  |  |  |
| MIRLET7F2 | HELLS |  |  |  |
| IGF2R | HPCAL1 |  |  |  |
| AEBP1 | KCNQ1 |  |  |  |
| FAM135B | LPAR1 |  |  |  |
| SNHG1 | MACF1 |  |  |  |
| PGLYRP1 | NR1H2 |  |  |  |
| CAV1 | PDE4DIP |  |  |  |
| EDN1 | PEPD |  |  |  |
| SIGLEC5 | PGM3 |  |  |  |
| SRP54 | PLAGL1 |  |  |  |
| USB1 | PSMA1 |  |  |  |
| PIK3C3 | PSMA2 |  |  |  |
| PTGER4 | RIPK2 |  |  |  |
| MMP25 | ROBO1 |  |  |  |
| IL5 | RPS3 |  |  |  |
| ITGA4 | SOX17 |  |  |  |
| PDGFB | SYNE1 |  |  |  |
| HSP90AA1 | TMSB10 |  |  |  |
| SCN2A | TPM2 |  |  |  |
| FOXP3 | UGT2B15 |  |  |  |
| CXCL1 | YWHAQ |  |  |  |
| CTSD | ADCY7 |  |  |  |
| MIR31HG | ANLN |  |  |  |
| SOD2 | ATRX |  |  |  |
| MIR203A | B4GALT1 |  |  |  |
| GDF5 | BTG3 |  |  |  |
| ILK | CASP10 |  |  |  |
| PWAR6 | CCT8 |  |  |  |
| CXCR3 | CHAF1B |  |  |  |
| MALAT1 | CXCL13 |  |  |  |
| TNFRSF1A | EBP |  |  |  |
| SERPINF1 | EIF5 |  |  |  |
| ITGB1 | FGL2 |  |  |  |
| GAA | FKBP4 |  |  |  |
| G6PC1 | HCK |  |  |  |
| VTN | HOPX |  |  |  |
| IGFBP3 | IFNA2 |  |  |  |
| NOD1 | KDM6B |  |  |  |
| CD44 | MAFK |  |  |  |
| AGTR1 | PAK1 |  |  |  |
| PDGFA | PSIP1 |  |  |  |
| SP1 | PSMB3 |  |  |  |
| ADIPOR1 | RAD23B |  |  |  |
| HLA-DRB3 | RARG |  |  |  |
| MIR23A | SLC13A3 |  |  |  |
| CCL7 | ABCC8 |  |  |  |
| IFI16 | ADAM8 |  |  |  |
| CREB1 | ANTXR2 |  |  |  |
| LEPR | APCS |  |  |  |
| GNRH1 | CAMK2N1 |  |  |  |
| C5AR1 | CDH3 |  |  |  |
| PTGER1 | CRYL1 |  |  |  |
| SIRT6 | DDX39A |  |  |  |
| MIR381 | DHRS7 |  |  |  |
| CDKN2A | EHMT2 |  |  |  |
| TNFSF13B | ENTPD5 |  |  |  |
| IL2RA | HAT1 |  |  |  |
| SFRP1 | ITGAX |  |  |  |
| IFNGR1 | KRT15 |  |  |  |
| PLEK | LAMA1 |  |  |  |
| PWAR1 | LAMA3 |  |  |  |
| IGSF3 | MYH11 |  |  |  |
| IFNL1 | NECTIN2 |  |  |  |
| GAS5 | NFIB |  |  |  |
| PKP2 | NGEF |  |  |  |
| GHRL | NME2 |  |  |  |
| TLR5 | OLFML3 |  |  |  |
| TGIF1 | ORC6 |  |  |  |
| APOE | PABPC1 |  |  |  |
| IL6ST | PMVK |  |  |  |
| THSD4 | PSMB4 |  |  |  |
| MIR210 | PTHLH |  |  |  |
| FNDC5 | RANBP1 |  |  |  |
| FBXO38 | RPLP0 |  |  |  |
| LINC01126 | SERBP1 |  |  |  |
| NIN | UBA1 |  |  |  |
| NELL1 | UBE2L6 |  |  |  |
| EFEMP2 | WFDC2 |  |  |  |
| CNR1 | XRCC6 |  |  |  |
| TRP-AGG2-5 | C1R |  |  |  |
| MIF | CDH11 |  |  |  |
| IL22 | CXADR |  |  |  |
| RELA | FANCA |  |  |  |
| FERMT2 | FANCD2 |  |  |  |
| MIAT | FCER1G |  |  |  |
| TNFSF10 | GMNN |  |  |  |
| MIR31 | GZMB |  |  |  |
| PCSK9 | IFITM2 |  |  |  |
| MEFV | IRF8 |  |  |  |
| FDCSP | ITIH3 |  |  |  |
| P2RX5-TAX1BP3 | MFAP4 |  |  |  |
| PTGDS | NDRG4 |  |  |  |
| KRT23 | PDE10A |  |  |  |
| KCNQ1OT1 | SYNCRIP |  |  |  |
| HLA-DRB5 | XPC |  |  |  |
| HLA-DRB4 | ALDH4A1 |  |  |  |
| MIR130A | ATP6 |  |  |  |
| IL12RB2 | C5AR1 |  |  |  |
| FGF1 | C6 |  |  |  |
| TGFBR1 | CEACAM1 |  |  |  |
| DEFA3 | COL15A1 |  |  |  |
| THBD | CRHR1 |  |  |  |
| CD28 | CRIM1 |  |  |  |
| IL36G | DDIT4L |  |  |  |
| CDKN2B | EHD4 |  |  |  |
| TNFRSF10D | EIF3A |  |  |  |
| KDM4B | ERBB4 |  |  |  |
| CSMD1 | ETNK1 |  |  |  |
| PSMA8 | FILIP1L |  |  |  |
| NKAIN2 | FUS |  |  |  |
| ZNF524 | GNAO1 |  |  |  |
| LINC00907 | GRK5 |  |  |  |
| GZMB | IFI30 |  |  |  |
| VAMP3 | JDP2 |  |  |  |
| LAMB3 | MAPK13 |  |  |  |
| PIK3R1 | MAPKAPK3 |  |  |  |
| SFTPD | MSH6 |  |  |  |
| SIGLEC7 | MT1A |  |  |  |
| WWTR1 | MYADM |  |  |  |
| CEBPB | NOG |  |  |  |
| TH | NPTX1 |  |  |  |
| ADM | PDE4D |  |  |  |
| MIRLET7C | PELI1 |  |  |  |
| OMP | PLIN3 |  |  |  |
| MMP21 | PSMB2 |  |  |  |
| MMP27 | RECK |  |  |  |
| MDN1 | RPA2 |  |  |  |
| VEZF1 | RPL3 |  |  |  |
| POP5 | RPS15A |  |  |  |
| ERICH3 | RPS27 |  |  |  |
| RGS2 | RUNX3 |  |  |  |
| MEPE | SERPINI1 |  |  |  |
| AGT | SOAT1 |  |  |  |
| FCER1A | STEAP1 |  |  |  |
| CD63 | SYNPO |  |  |  |
| BCL2 | TGFBR3 |  |  |  |
| MSX2 | TNIP1 |  |  |  |
| MYD88 | TYROBP |  |  |  |
| GHSR | VSNL1 |  |  |  |
| GPX3 | WNT3A |  |  |  |
| TGFB3 | ANKRD11 |  |  |  |
| KAT2A | ATP1A3 |  |  |  |
| CDH1 | ATP6V1A |  |  |  |
| GAPDH | BMPER |  |  |  |
| TGFA | CD47 |  |  |  |
| IL37 | CLTB |  |  |  |
| HEY1 | CRK |  |  |  |
| IL3 | CSRNP1 |  |  |  |
| CALCR | CXCL16 |  |  |  |
| TLR1 | CYP2R1 |  |  |  |
| TRAF6 | DTYMK |  |  |  |
| OIP5-AS1 | EDEM1 |  |  |  |
| PECAM1 | FRZB |  |  |  |
| MIR132 | GNAI3 |  |  |  |
| MIR23B | GRM5 |  |  |  |
| S100A12 | GZMA |  |  |  |
| SNORD15A | IRF6 |  |  |  |
| XIAP | KRT7 |  |  |  |
| MIR24-1 | LAMA2 |  |  |  |
| SEMA3A | NCAPH |  |  |  |
| ANGPTL4 | NSF |  |  |  |
| ADIPOR2 | PDCD6IP |  |  |  |
| MIR30E | PERP |  |  |  |
| CCR1 | PHYH |  |  |  |
| CTHRC1 | PIK3R3 |  |  |  |
| ITLN1 | PIM3 |  |  |  |
| CP | RAB3B |  |  |  |
| MIR379 | RRM2B |  |  |  |
| SERPINB7 | RSRC2 |  |  |  |
| CST7 | TGIF1 |  |  |  |
| PRSS57 | TP53BP1 |  |  |  |
| C5 | UCK2 |  |  |  |
| TIMP3 | VKORC1 |  |  |  |
| LINC-ROR | ATP2B2 |  |  |  |
| LY96 | ATP5IF1 |  |  |  |
| CXCL6 | CCR6 |  |  |  |
| SMAD5-AS1 | CHRNB4 |  |  |  |
| IGFBP5 | DNM1 |  |  |  |
| CST1 | ETV1 |  |  |  |
| LINC01133 | FGL1 |  |  |  |
| SLC6A4 | FNBP1 |  |  |  |
| ANGPT1 | GADD45G |  |  |  |
| MIR1246 | GCNT3 |  |  |  |
| SPON1 | GNG11 |  |  |  |
| PLAUR | HAO2 |  |  |  |
| RHOA | HAS2 |  |  |  |
| CASP7 | HHIP |  |  |  |
| MIR498 | MTA1 |  |  |  |
| ADRB3 | MYLIP |  |  |  |
| CDH2 | NFE2L1 |  |  |  |
| FGFR3 | NPR2 |  |  |  |
| HAS2-AS1 | NR0B1 |  |  |  |
| LPL | PDE7B |  |  |  |
| NPM1 | PIK3C3 |  |  |  |
| TYMS | PNN |  |  |  |
| DKC1 | PPFIBP1 |  |  |  |
| FGF3 | PRDM2 |  |  |  |
| NHP2 | PRIM2 |  |  |  |
| OCRL | PSMC3 |  |  |  |
| TCIRG1 | RAD23A |  |  |  |
| CLPB | RBM47 |  |  |  |
| PARN | RPL13A |  |  |  |
| RTEL1 | SND1 |  |  |  |
| NOP10 | TRAF3 |  |  |  |
| TINF2 | TRIM25 |  |  |  |
| WRAP53 | ACSS1 |  |  |  |
| SRP19 | ACTR3 |  |  |  |
| CTC1 | ADAM10 |  |  |  |
| GORAB | BDH2 |  |  |  |
| TERC | BGLAP |  |  |  |
| PIEZO1 | CD52 |  |  |  |
| MIR30B | CRY2 |  |  |  |
| NR1I2 | CSE1L |  |  |  |
| CNR2 | CSNK2A1 |  |  |  |
| TRPA1 | CYP39A1 |  |  |  |
| VCAN | DNAJC12 |  |  |  |
| BMP1 | EIF6 |  |  |  |
| PLTP | FBLIM1 |  |  |  |
| FBLIM1 | FNDC5 |  |  |  |
| MIRLET7F1 | HNRNPM |  |  |  |
| MIR30A | KLHL21 |  |  |  |
| PCAT1 | LIMK1 |  |  |  |
| GOT1 | LMNB2 |  |  |  |
| GGT1 | LPCAT1 |  |  |  |
| PHEX | NEBL |  |  |  |
| MIR199A1 | NET1 |  |  |  |
| MIR28 | OAS3 |  |  |  |
| TFF1 | OSM |  |  |  |
| ADAMTS4 | PARP9 |  |  |  |
| MIR99A | PCOLCE |  |  |  |
| HSPA5 | PLEKHF1 |  |  |  |
| CCL11 | PPIB |  |  |  |
| GSR | PPT1 |  |  |  |
| KNG1 | PTBP1 |  |  |  |
| MEG8 | RBBP8 |  |  |  |
| ABCA1 | RPL27A |  |  |  |
| BLK | RPS15 |  |  |  |
| ACTN1 | RRP12 |  |  |  |
| ACTN2 | SAA3 |  |  |  |
| EPHA3 | SF1 |  |  |  |
| GRIK1 | SLC41A2 |  |  |  |
| NLGN1 | SLC6A12 |  |  |  |
| CAMK4 | SPAG9 |  |  |  |
| CDH13 | TAPBP |  |  |  |
| CHD1 | TCEA3 |  |  |  |
| ITGA8 | TCF19 |  |  |  |
| FOXA1 | TCP11L2 |  |  |  |
| MTHFS | TET1 |  |  |  |
| ROBO2 | TFF3 |  |  |  |
| ETS2 | UPB1 |  |  |  |
| FRG1 | YBX3 |  |  |  |
| PDCD6IP | ZFHX3 |  |  |  |
| PTPRT | ACOT4 |  |  |  |
| RYR3 | ARL4A |  |  |  |
| SUMF1 | BCL2L2 |  |  |  |
| MBD2 | CAVIN3 |  |  |  |
| RBFOX1 | CCKBR |  |  |  |
| LRP12 | CCL8 |  |  |  |
| RGMA | CHGA |  |  |  |
| RIT2 | CHRM4 |  |  |  |
| SNTB1 | CIITA |  |  |  |
| IQSEC1 | CPNE8 |  |  |  |
| KCNJ16 | DCBLD2 |  |  |  |
| LRP1B | ERCC2 |  |  |  |
| SEMA6A | GAS5 |  |  |  |
| SETMAR | GRB14 |  |  |  |
| SMURF2 | HDGF |  |  |  |
| WDR36 | HES6 |  |  |  |
| ERC2 | KALRN |  |  |  |
| ERGIC1 | KRT4 |  |  |  |
| HS6ST2 | KYAT1 |  |  |  |
| HYCC1 | LGALS9 |  |  |  |
| SS18 | LPAR6 |  |  |  |
| WDR73 | LRRFIP1 |  |  |  |
| ETNK2 | MEIS2 |  |  |  |
| GPN1 | MIR155 |  |  |  |
| HLA-DOA | MLEC |  |  |  |
| VPREB1 | MPZL2 |  |  |  |
| NMUR2 | NAT8 |  |  |  |
| PARP15 | NCALD |  |  |  |
| ADAMTS15 | NRGN |  |  |  |
| BCORL1 | OIP5 |  |  |  |
| ETAA1 | PGM1 |  |  |  |
| NRSN1 | PPP2R5C |  |  |  |
| PGPEP1 | PRKD3 |  |  |  |
| MFSD1 | PYCR1 |  |  |  |
| ZNF385D | RPL22 |  |  |  |
| C1orf87 | RPS12 |  |  |  |
| CCDC13 | SH3PXD2A |  |  |  |
| DAOA | SOX18 |  |  |  |
| PRB2 | ST3GAL6 |  |  |  |
| RAB6C | STMN2 |  |  |  |
| HMX3 | SYNE2 |  |  |  |
| NKAIN3 | UBA7 |  |  |  |
| ZNF579 | WDHD1 |  |  |  |
| FAM47A | ACTN2 |  |  |  |
| PLEKHG7 | ADCY6 |  |  |  |
| FAM180A | ADHFE1 |  |  |  |
| ST20 | ARL5B |  |  |  |
| LINC02870 | BARD1 |  |  |  |
| ST20-MTHFS | BMP5 |  |  |  |
| LINC00208 | CAMK2G |  |  |  |
| IGL | DDX1 |  |  |  |
| ETS2-AS1 | DLL4 |  |  |  |
| ROCK1P1 | DUOX2 |  |  |  |
| RYR3-DT | DUSP16 |  |  |  |
| LINC01811 | DUSP3 |  |  |  |
| NPM1P2 | EEPD1 |  |  |  |
| CDKN3 | EIF1 |  |  |  |
| FAS | ELOVL1 |  |  |  |
| ATF4 | ENPP3 |  |  |  |
| EGLN1 | G3BP1 |  |  |  |
| SOCS3 | GLIPR1 |  |  |  |
| RECK | HSP70 |  |  |  |
| GSK3B | KLF11 |  |  |  |
| SERPINH1 | MAPRE1 |  |  |  |
| LAMA2 | MECP2 |  |  |  |
| GPD1L | MFN2 |  |  |  |
| SOS2 | MIA2 |  |  |  |
| ATL1 | NNT |  |  |  |
| SEL1L | NTF3 |  |  |  |
| STT3B | PAX3 |  |  |  |
| RAB28 | PIMREG |  |  |  |
| L2HGDH | POLE2 |  |  |  |
| MAP4K5 | PPM1B |  |  |  |
| FHOD3 | RAD18 |  |  |  |
| ARHGAP18 | RPL32 |  |  |  |
| NKX3-2 | RPS24 |  |  |  |
| SAV1 | RPS5 |  |  |  |
| CDKL1 | RSRP1 |  |  |  |
| CMTM8 | SFXN2 |  |  |  |
| DMAC2L | SLC4A2 |  |  |  |
| TPGS2 | SMC3 |  |  |  |
| OSBPL10 | SON |  |  |  |
| KIAA1328 | SRSF1 |  |  |  |
| ZNF860 | SUOX |  |  |  |
| NEAT1 | THBS4 |  |  |  |
| HSP90AB2P | ADK |  |  |  |
| LOC102723409 | ANP32A |  |  |  |
| HSPB1 | ANXA11 |  |  |  |
| KRT18 | BCAT2 |  |  |  |
| ACTA2 | CCN3 |  |  |  |
| CCR4 | CDON |  |  |  |
| SMAD1 | CENPW |  |  |  |
| LIPA | CLSPN |  |  |  |
| MIR126 | CORO1C |  |  |  |
| TUFT1 | CSPG4 |  |  |  |
| PADI2 | DDX60 |  |  |  |
| SOCS1 | DNM2 |  |  |  |
| PDCD1 | DSCC1 |  |  |  |
| MIR9-1 | E2F7 |  |  |  |
| PRRX2 | EEF1B2 |  |  |  |
| CBS | EFNB2 |  |  |  |
| IDO1 | EP300 |  |  |  |
| CD274 | FERMT2 |  |  |  |
| KRT1 | FGFR2 |  |  |  |
| HOTAIRM1 | FGG |  |  |  |
| ICOS | GALNT3 |  |  |  |
| MIR182 | GBP4 |  |  |  |
| MYC | GPX8 |  |  |  |
| IL24 | HGD |  |  |  |
| FLNA | HNRNPL |  |  |  |
| MIR93 | HPD |  |  |  |
| ZEB2 | ILK |  |  |  |
| MIR125B1 | KHK |  |  |  |
| C3 | LGR4 |  |  |  |
| PTX3 | MBNL1 |  |  |  |
| CCR6 | MDM4 |  |  |  |
| KCNQ5 | MPRIP |  |  |  |
| NME8 | NMT2 |  |  |  |
| CRACR2A | NRXN2 |  |  |  |
| GPR141 | PALMD |  |  |  |
| HEY2 | RABGGTB |  |  |  |
| MIR22HG | RASGRP3 |  |  |  |
| PDGFRB | REEP5 |  |  |  |
| GRN | RFC5 |  |  |  |
| IL17RA | RPL18 |  |  |  |
| LYZ | RPS14 |  |  |  |
| HAS2 | SRSF2 |  |  |  |
| MIR383 | SYTL2 |  |  |  |
| SCN4A | TJP3 |  |  |  |
| CCR2 | TMEM37 |  |  |  |
| GH-LCR | TNFSF8 |  |  |  |
| MIR1306 | TREM2 |  |  |  |
| FTO | TROAP |  |  |  |
| GLDC | TTPA |  |  |  |
| TRPS1 | XPO1 |  |  |  |
| HTR4 | ZC3H12A |  |  |  |
| PKN2 | AHNAK2 |  |  |  |
| FZD8 | ALOX12 |  |  |  |
| GRID1 | ASRGL1 |  |  |  |
| KCNK1 | BAP1 |  |  |  |
| OTOF | BAZ1A |  |  |  |
| WWC1 | CABLES1 |  |  |  |
| CAMTA1 | CBX7 |  |  |  |
| DAB2IP | CDC34 |  |  |  |
| CLIC5 | CFP |  |  |  |
| JDP2 | CPLX2 |  |  |  |
| TBC1D1 | CTBP2 |  |  |  |
| WAPL | EFNB1 |  |  |  |
| ANKRD30A | ERCC6 |  |  |  |
| CSMD3 | FGF18 |  |  |  |
| LNPK | FLOT2 |  |  |  |
| UHRF2 | GALM |  |  |  |
| PTTG2 | GNA12 |  |  |  |
| TENM2 | HDAC4 |  |  |  |
| DEFA4 | KAT2B |  |  |  |
| EVX2 | KCNK5 |  |  |  |
| MAP3K21 | KNSTRN |  |  |  |
| TTLL11 | LDHD |  |  |  |
| CIB4 | LHX2 |  |  |  |
| TPD52L3 | MLPH |  |  |  |
| LCN2 | MSX2 |  |  |  |
| GAD2 | NISCH |  |  |  |
| CYP2E1 | NT5DC2 |  |  |  |
| PF4 | NXF1 |  |  |  |
| BACH1 | PARD3 |  |  |  |
| TNN | PCM1 |  |  |  |
| TMSB4X | PRPS2 |  |  |  |
| HDAC9 | PSMA6 |  |  |  |
| EPO | PSMD8 |  |  |  |
| MIR148A | RAD21 |  |  |  |
| RARRES2 | RAD54B |  |  |  |
| MMP26 | RBMS1 |  |  |  |
| DCST1 | RPL12 |  |  |  |
| PIK3CD | RPLP2 |  |  |  |
| DLG2 | RPS8 |  |  |  |
| SLC1A3-AS1 | SAA2 |  |  |  |
| POU5F1 | SCARA3 |  |  |  |
| ADAM28 | SEMA3E |  |  |  |
| MIR3198-1 | SH2B3 |  |  |  |
| SNHG5 | SH3BGRL |  |  |  |
| MIA3 | SLC39A6 |  |  |  |
| H3C14 | SLC44A1 |  |  |  |
| G6PC3 | SOBP |  |  |  |
| CSN1S1 | SPRED1 |  |  |  |
| BMP8A | SRGAP3 |  |  |  |
| CCND2 | TNPO1 |  |  |  |
| DELEC1 | TRIM16 |  |  |  |
| THBS1 | UBE2H |  |  |  |
| PROM1 | WNK1 |  |  |  |
| GATA3 | ZFR |  |  |  |
| RIPK2 | ACAD11 |  |  |  |
| CHRNA7 | AKR1C4 |  |  |  |
| EGR1 | ARHGAP29 |  |  |  |
| LGALS1 | ATG3 |  |  |  |
| SPRY1 | ATP6V0A1 |  |  |  |
| GSTP1 | BTF3 |  |  |  |
| HRH1 | C3AR1 |  |  |  |
| TGFBI | DAD1 |  |  |  |
| CSF3R | DCAKD |  |  |  |
| RAB27A | DDB2 |  |  |  |
| TGM1 | DNAJC2 |  |  |  |
| SNORD80 | DPT |  |  |  |
| DNM1L | EPHA1 |  |  |  |
| NRP1 | EWSR1 |  |  |  |
| CX3CL1 | GDF10 |  |  |  |
| VEGFB | GDI1 |  |  |  |
| SOCS6 | GPRC5C |  |  |  |
| PON1 | GTPBP4 |  |  |  |
| MIRLET7A1 | HELZ2 |  |  |  |
| MIR20A | HNRNPA3 |  |  |  |
| MIR520D | HNRNPU |  |  |  |
| TIMP4 | HSPA12A |  |  |  |
| C4A | HTRA3 |  |  |  |
| DSG1 | IL2RG |  |  |  |
| FGL2 | IL4RA |  |  |  |
| FCGR2C | IPO7 |  |  |  |
| MIR16-1 | KDM1A |  |  |  |
| MZB1 | LAD1 |  |  |  |
| LOC126806446 | LGALS2 |  |  |  |
| PWRN1 | LRP5 |  |  |  |
| MYLK | MALT1 |  |  |  |
| SLC52A3 | MICAL2 |  |  |  |
| VPS11 | MOXD1 |  |  |  |
| SLC52A2 | NFYB |  |  |  |
| INPP5D | NOP2 |  |  |  |
| RPS27 | OLFML2B |  |  |  |
| HTRA1 | PCCA |  |  |  |
| CX3CR1 | PCCB |  |  |  |
| EBI3 | PCDH17 |  |  |  |
| MIR29B1 | PDE2A |  |  |  |
| LINC00687 | PDP1 |  |  |  |
| ESRRA | PHF19 |  |  |  |
| PELP1 | PLEKHB1 |  |  |  |
| ITGA6 | PPRC1 |  |  |  |
| MIR511 | PREX1 |  |  |  |
| CFH | PRSS12 |  |  |  |
| MTHFR | PXMP2 |  |  |  |
| ITGA2 | RGS9 |  |  |  |
| ADCY10 | RPL11 |  |  |  |
| CD80 | RPL28 |  |  |  |
| ADAMTS9 | RPL31 |  |  |  |
| FBXO5 | SACS |  |  |  |
| NLRC5 | SHANK3 |  |  |  |
| TNFSF12 | SKP1 |  |  |  |
| MIR200A | SLC12A4 |  |  |  |
| MIR222 | SLC27A4 |  |  |  |
| TLN1 | SMIM14 |  |  |  |
| TLN2 | SPSB1 |  |  |  |
| MIR18A | SRP9 |  |  |  |
| HACD1 | SRPRA |  |  |  |
| CCL18 | SRSF6 |  |  |  |
| MIR34A | STRBP |  |  |  |
| CD19 | STX1A |  |  |  |
| FFAR1 | SYVN1 |  |  |  |
| MIR320D1 | TLE3 |  |  |  |
| GJA1 | TLR5 |  |  |  |
| TFRC | TNFRSF8 |  |  |  |
| ITGA1 | TUFM |  |  |  |
| MIR320A | ADAR |  |  |  |
| TRPV4 | ALS2 |  |  |  |
| FTH1 | ASF1A |  |  |  |
| FTL | BACH2 |  |  |  |
| KDM6B | CARHSP1 |  |  |  |
| AQP3 | CCT4 |  |  |  |
| DLX5 | CDA |  |  |  |
| SEMA3D | CDS1 |  |  |  |
| TNMD | CES3 |  |  |  |
| MDH2 | CHD4 |  |  |  |
| DUSP1 | COBL |  |  |  |
| VAV1 | CPEB4 |  |  |  |
| NCR2 | DCTPP1 |  |  |  |
| CDC42EP2 | DDX39B |  |  |  |
| ADGRE1 | DDX6 |  |  |  |
| MIR25 | EHD2 |  |  |  |
| ST8SIA6-AS1 | ELF1 |  |  |  |
| MME | ENTPD2 |  |  |  |
| KDM1A | FLI1 |  |  |  |
| METTL3 | GATA3 |  |  |  |
| ST8SIA1 | GCNT1 |  |  |  |
| KDR | GPC4 |  |  |  |
| LRG1 | GPX7 |  |  |  |
| ANGPTL2 | HFE |  |  |  |
| SOS1 | HIP1 |  |  |  |
| METTL25B | IGSF3 |  |  |  |
| CHI3L1 | IGTP |  |  |  |
| THNSL2 | KCND3 |  |  |  |
| ECE1 | KCNH1 |  |  |  |
| HSPA4 | ME3 |  |  |  |
| IL1RL1 | MPDZ |  |  |  |
| RAC2 | MPP1 |  |  |  |
| WAS | MTHFR |  |  |  |
| DOCK8 | NECTIN3 |  |  |  |
| SBDS | PGM2 |  |  |  |
| EZH2 | PIK3R5 |  |  |  |
| ACVR2B | PRRC2C |  |  |  |
| LBR | PSMD12 |  |  |  |
| NTF4 | RERE |  |  |  |
| BHLHE40 | RFC1 |  |  |  |
| CKAP2L | RHBDF1 |  |  |  |
| BEGAIN | RPF2 |  |  |  |
| UBE3D | RPL27 |  |  |  |
| MIR22 | RPL37 |  |  |  |
| PTCSC3 | RPN1 |  |  |  |
| MAP2K1 | RPS16 |  |  |  |
| NFKBIA | SLC6A15 |  |  |  |
| CTRL | SVIL |  |  |  |
| TLR10 | THOP1 |  |  |  |
| DLEU2 | TOLLIP |  |  |  |
| ODC1 | TRA2B |  |  |  |
| PINK1 | TRIM63 |  |  |  |
| ENG | TUBB4A |  |  |  |
| ITGA11 | UBA52 |  |  |  |
| CASP1 | WIPF1 |  |  |  |
| AHSG | ZFHX4 |  |  |  |
| PLXNC1 | ANK2 |  |  |  |
| HNF4A | ANXA8 |  |  |  |
| TPR | CBX5 |  |  |  |
| BRD9 | CCL12 |  |  |  |
| ENTR1 | CDCP1 |  |  |  |
| SNORD77 | CLCN4 |  |  |  |
| MIR301A | CYP2B2 |  |  |  |
| CD248 | DICER1 |  |  |  |
| SOX9 | DNAJA2 |  |  |  |
| SMURF1 | DYRK3 |  |  |  |
| MCRS1 | EIF4G2 |  |  |  |
| MIR7-1 | ELOVL7 |  |  |  |
| CYCS | ENAH |  |  |  |
| TNFAIP3 | ERP44 |  |  |  |
| FOXO1 | FCGR3 |  |  |  |
| MIR99B | FXYD6 |  |  |  |
| KRT19 | H2AC6 |  |  |  |
| CYBB | HDAC7 |  |  |  |
| CSTA | HSPB7 |  |  |  |
| MIR205 | IRF2BP2 |  |  |  |
| ETS1 | IVNS1ABP |  |  |  |
| HIF1A-AS1 | JADE2 |  |  |  |
| AKR1B1 | KANK2 |  |  |  |
| FCGR1A | KPNB1 |  |  |  |
| TNFRSF10C | LAP3 |  |  |  |
| MT-CO1 | MAPK7 |  |  |  |
| MIR133B | NOC3L |  |  |  |
| MIR335 | NUDT1 |  |  |  |
| MSC-AS1 | PDCD11 |  |  |  |
| MIR758 | PDIA3 |  |  |  |
| MMP19 | PHF20L1 |  |  |  |
| NOTUM | PLAA |  |  |  |
| PIMREG | PLEK |  |  |  |
| RPUSD2 | PRKCI |  |  |  |
| MTFR2 | PSMD2 |  |  |  |
| USF2 | PYGB |  |  |  |
| KDM7A-DT | RAB6A |  |  |  |
| LOC110806262 | RBP7 |  |  |  |
| TBXT | RPS10 |  |  |  |
| TNFSF13 | SAFB |  |  |  |
| HBA1 | SIPA1L2 |  |  |  |
| LAMC2 | SLC25A29 |  |  |  |
| KCNK2 | SLC37A2 |  |  |  |
| KLF10 | SLC38A4 |  |  |  |
| ZFP42 | SLCO4A1 |  |  |  |
| MIR30D | SORBS3 |  |  |  |
| LOXL1-AS1 | SPN |  |  |  |
| CACNA1G-AS1 | SUSD2 |  |  |  |
| KRT16 | TLE1 |  |  |  |
| TRPM2 | TMC6 |  |  |  |
| MIR141 | TMEM176A |  |  |  |
| MIR3917 | TNNC2 |  |  |  |
| OCA2 | TPD52 |  |  |  |
| HPS3 | UBA5 |  |  |  |
| IVL | UOX |  |  |  |
| RNF217-AS1 | USP10 |  |  |  |
| MYH11 | VAV2 |  |  |  |
| ANTXR2 | XPOT |  |  |  |
| TRIM25 | ACIN1 |  |  |  |
| LRP5 | ADGRE5 |  |  |  |
| SSTR2 | AKAP9 |  |  |  |
| MIR29A | ANP32B |  |  |  |
| CARMN | ARF1 |  |  |  |
| DNMT3B | ARHGAP11A |  |  |  |
| ETV1 | ARHGAP22 |  |  |  |
| SNHG14 | ATP6V0C |  |  |  |
| PWAR4 | AZGP1 |  |  |  |
| HGFAC | BANF1 |  |  |  |
| LAMA3 | BCL10 |  |  |  |
| P3H4 | C2CD2 |  |  |  |
| KRT5 | CTSE |  |  |  |
| TBX21 | CUX2 |  |  |  |
| IL7R | CYFIP2 |  |  |  |
| P2RY1 | CYP2C55 |  |  |  |
| SMAD5 | DLG2 |  |  |  |
| SCX | FARSA |  |  |  |
| SLC11A1 | FHOD3 |  |  |  |
| CCN1 | FMNL2 |  |  |  |
| SLAMF7 | FMOD |  |  |  |
| RBMS1 | GALNT1 |  |  |  |
| MFN2 | GCAT |  |  |  |
| RXRA | GFPT1 |  |  |  |
| PTPN22 | GNPDA1 |  |  |  |
| FGD5 | H4C8 |  |  |  |
| MMP28 | HERC6 |  |  |  |
| IRF4 | HNRNPC |  |  |  |
| MIRLET7D | ICA1 |  |  |  |
| MIR1305 | IRF2 |  |  |  |
| LOC109286563 | LARP1 |  |  |  |
| MIR1260B | LPAR3 |  |  |  |
| A2M | MAVS |  |  |  |
| IGFBP6 | METTL3 |  |  |  |
| ATF6 | MT1M |  |  |  |
| ALMS1 | MYO1C |  |  |  |
| IL2RB | NDUFV3 |  |  |  |
| SHH | NUP155 |  |  |  |
| HLA-DQA1 | PCSK1 |  |  |  |
| MFN1 | PIK3AP1 |  |  |  |
| NCOA4 | PKIA |  |  |  |
| ORM1 | PLXND1 |  |  |  |
| CCL13 | PTGES3 |  |  |  |
| MIR143 | RAB11FIP4 |  |  |  |
| NAT2 | RASSF2 |  |  |  |
| SELPLG | RPL14 |  |  |  |
| ICAM2 | RPL36 |  |  |  |
| RASGRP2 | RPS18 |  |  |  |
| VPS45 | RPS26 |  |  |  |
| APBB1IP | RPS6KA5 |  |  |  |
| RASSF5 | RPS9 |  |  |  |
| ITGAD | SCN1B |  |  |  |
| DNAJC21 | SDCBP |  |  |  |
| JAGN1 | SEC23B |  |  |  |
| CHGA | SFTPC |  |  |  |
| VIP | SFTPD |  |  |  |
| APCS | SFXN1 |  |  |  |
| CPQ | SGPL1 |  |  |  |
| ANO5 | SLC44A2 |  |  |  |
| PDGFRA | TRAM1 |  |  |  |
| BAX | TSPAN18 |  |  |  |
| SMC3 | USP36 |  |  |  |
| HLA-G | ZCCHC7 |  |  |  |
| BMI1 | ZFAND5 |  |  |  |
| PPBP | ACVRL1 |  |  |  |
| C4B | ADH5 |  |  |  |
| CST4 | ANKH |  |  |  |
| MIR409 | ARPP19 |  |  |  |
| ST2 | BCO1 |  |  |  |
| PYCARD | CACNB2 |  |  |  |
| TAC1 | CARTPT |  |  |  |
| PLEKHO1 | CD82 |  |  |  |
| LOC101927661 | CHST1 |  |  |  |
| LOC101927025 | CLIP1 |  |  |  |
| ALOX15 | CLYBL |  |  |  |
| HSPE1 | COMMD1 |  |  |  |
| IL36RN | CPB2 |  |  |  |
| CLC | CSGALNACT1 |  |  |  |
| MIR145 | DBF4 |  |  |  |
| TRB | DDB1 |  |  |  |
| STAT3 | DEPDC1B |  |  |  |
| ADORA3 | EGFL7 |  |  |  |
| CD180 | EIF3I |  |  |  |
| CD1C | ETNPPL |  |  |  |
| MIR200C | FABP3 |  |  |  |
| MIR92A1 | FSTL3 |  |  |  |
| STAT1 | FYN |  |  |  |
| PREP | GABRA4 |  |  |  |
| LRP2 | GAS1 |  |  |  |
| ALDH18A1 | GHITM |  |  |  |
| BGN | GMPR |  |  |  |
| B3GAT3 | GNAL |  |  |  |
| CHST14 | GRAMD1B |  |  |  |
| B3GALT6 | GREM2 |  |  |  |
| DSE | HERC5 |  |  |  |
| SLC24A5 | HEY2 |  |  |  |
| PRKN | KIF5A |  |  |  |
| MLKL | LACTB2 |  |  |  |
| MIR508 | LRPAP1 |  |  |  |
| LBX2-AS1 | MECOM |  |  |  |
| MIR496 | MPEG1 |  |  |  |
| SNODB1771 | MST1 |  |  |  |
| GJB1 | NF2 |  |  |  |
| HHIP-AS1 | PARP12 |  |  |  |
| ADAMTS14 | PCDH1 |  |  |  |
| EPAS1 | PGM5 |  |  |  |
| ITGA5 | PHACTR2 |  |  |  |
| PAX5 | PLCB3 |  |  |  |
| KLF6 | PRKD2 |  |  |  |
| SNAI2 | PSMD1 |  |  |  |
| LGALS9 | PTP4A3 |  |  |  |
| RASA2 | RAB11FIP1 |  |  |  |
| MIR185 | RAB27A |  |  |  |
| MIR26B | RBM25 |  |  |  |
| MIR370 | RPL19 |  |  |  |
| LINC00616 | RPL26 |  |  |  |
| TLR7 | RPL34 |  |  |  |
| BIRC3 | SKI |  |  |  |
| EPRS1 | SLA |  |  |  |
| TREM2 | SLC25A22 |  |  |  |
| ADAMTS1 | SLC43A3 |  |  |  |
| SIK2 | SMARCD2 |  |  |  |
| AIM2 | SPAG1 |  |  |  |
| PDGFC | SVEP1 |  |  |  |
| TFF3 | TC2N |  |  |  |
| MMRN1 | TMEM176B |  |  |  |
| CXCL5 | TOX2 |  |  |  |
| MS4A2 | TP53I3 |  |  |  |
| S100A7 | VAV1 |  |  |  |
| TLX1NB | ACP1 |  |  |  |
| MIR155HG | AFF1 |  |  |  |
| LEF1-AS1 | AP1G1 |  |  |  |
| NTRK1 | ASPN |  |  |  |
| BRCA1 | B3GNT2 |  |  |  |
| GALNS | BCL2L13 |  |  |  |
| IGFBP1 | CAMP |  |  |  |
| LMNA | CAPN6 |  |  |  |
| MUC7 | CELF1 |  |  |  |
| CSF1R | CLSTN1 |  |  |  |
| ERN1 | COL11A2 |  |  |  |
| CETP | CPA4 |  |  |  |
| MAPK10 | CYP4A11 |  |  |  |
| MAF | DMGDH |  |  |  |
| NTN1 | EIF4EBP2 |  |  |  |
| CASP5 | EMX2 |  |  |  |
| CXCR5 | ESR2B |  |  |  |
| SERPINB1 | FN3K |  |  |  |
| SLIT3 | GNAZ |  |  |  |
| CXCL13 | GPR146 |  |  |  |
| CXCL2 | HMGN1 |  |  |  |
| MUC4 | HOXB9 |  |  |  |
| DUSP2 | INPPL1 |  |  |  |
| UNC5B | LIMS1 |  |  |  |
| MIR4435-2HG | MAP3K20 |  |  |  |
| MIR124-3 | NRBP2 |  |  |  |
| MIR15A | NUP50 |  |  |  |
| MIR301B | OBSL1 |  |  |  |
| ZFY-AS1 | OLA1 |  |  |  |
| FGFR1 | OSBPL6 |  |  |  |
| F9 | P2RY6 |  |  |  |
| ITGAV | POLD3 |  |  |  |
| GPNMB | RASGRP2 |  |  |  |
| ELK1 | RBBP6 |  |  |  |
| KLF5 | RPS17 |  |  |  |
| SKAP2 | RTN4R |  |  |  |
| MIR26A1 | SAR1B |  |  |  |
| HLA-S | SEC24D |  |  |  |
| MIRLET7B | SLC24A3 |  |  |  |
| LINC01638 | SLC44A4 |  |  |  |
| FAM20C | SLC66A3 |  |  |  |
| EEA1 | SOS1 |  |  |  |
| INPP5A | SSPN |  |  |  |
| SCYL1 | SUZ12 |  |  |  |
| SNURF | THOC2 |  |  |  |
| AKT2 | TNFRSF14 |  |  |  |
| IKBKB | TNS2 |  |  |  |
| ADRB1 | TPST1 |  |  |  |
| TXN | TSPAN7 |  |  |  |
| SLIT2 | AHCYL1 |  |  |  |
| SERPINA3 | ANP32E |  |  |  |
| GPX1 | APLP1 |  |  |  |
| SOCS2 | ARHGAP26 |  |  |  |
| USP5 | ATXN2 |  |  |  |
| SOX11 | BCL9L |  |  |  |
| CCL22 | C1QTNF6 |  |  |  |
| CCL25 | CCL1 |  |  |  |
| IRGM | CDCA7L |  |  |  |
| CST2 | CDH4 |  |  |  |
| MIR499A | CENPJ |  |  |  |
| MIR299 | CLDN4 |  |  |  |
| MRLN | COL10A1 |  |  |  |
| MIR203B | CORO6 |  |  |  |
| SATB2 | CSTF3 |  |  |  |
| AXL | CTBS |  |  |  |
| FGFR4 | EMG1 |  |  |  |
| ANGPT2 | ESAM |  |  |  |
| ENTPD1 | FCGRT |  |  |  |
| NGFR | FZD5 |  |  |  |
| KAT6B | GTF2H1 |  |  |  |
| CCNE2 | H2AZ2 |  |  |  |
| PDLIM7 | HECW2 |  |  |  |
| SAA4 | HLCS |  |  |  |
| ADPRH | HLF |  |  |  |
| ADAMTS9-AS2 | HNF1B |  |  |  |
| NGF | HOXA5 |  |  |  |
| G6PD | IL6RA |  |  |  |
| PARP1 | ITPKB |  |  |  |
| VWF | KCNAB1 |  |  |  |
| SERPINI1 | KCNE3 |  |  |  |
| MB | KDM5A |  |  |  |
| IL23R | KIDINS220 |  |  |  |
| RCAN1 | KRT80 |  |  |  |
| PTGES | LSM2 |  |  |  |
| MADCAM1 | LYVE1 |  |  |  |
| SFRP2 | MAMDC2 |  |  |  |
| COG2 | MBOAT1 |  |  |  |
| TFPI2 | MEST |  |  |  |
| LINC00473 | MIR146A |  |  |  |
| MIR98 | MUTYH |  |  |  |
| MIR103A2 | NIBAN2 |  |  |  |
| LINC01433 | NIN |  |  |  |
| RGMB-AS1 | NLE1 |  |  |  |
| MAGEA8-AS1 | PABPN1 |  |  |  |
| ROCR | PDGFD |  |  |  |
| GIRGL | PNO1 |  |  |  |
| HRAT17 | POP1 |  |  |  |
| ENSG00000272851 | PRKAR1B |  |  |  |
| PIK3CA | PTGFRN |  |  |  |
| HDAC2 | RAD51C |  |  |  |
| ACVR1 | RGMA |  |  |  |
| BMPR2 | RNF13 |  |  |  |
| HDAC1 | SEMA4G |  |  |  |
| ADCY1 | SLC11A1 |  |  |  |
| CASP9 | SLC15A3 |  |  |  |
| ANXA1 | SLC16A12 |  |  |  |
| HEXA | SLC35F2 |  |  |  |
| IRAK3 | SLC45A3 |  |  |  |
| F3 | SLC6A1 |  |  |  |
| LAMP2 | SNX29 |  |  |  |
| CD86 | SRPK2 |  |  |  |
| PXN | SRSF3 |  |  |  |
| KRT13 | SSRP1 |  |  |  |
| SUV39H1 | STX11 |  |  |  |
| AP3B2 | SUMO1 |  |  |  |
| MICB | TENT5A |  |  |  |
| CCL17 | TESC |  |  |  |
| CXCR6 | TLCD1 |  |  |  |
| MICA | TOB2 |  |  |  |
| RGS12 | UAP1L1 |  |  |  |
| MOK | UROD |  |  |  |
| IL17C | WDR1 |  |  |  |
| DIRC3 | WDR26 |  |  |  |
| FAM30A | WNT1 |  |  |  |
| RNA18SN1 | ZBP1 |  |  |  |
| TEK | ADRM1 |  |  |  |
| PROS1 | AIMP1 |  |  |  |
| WNT1 | AMPD2 |  |  |  |
| GDF2 | ANAPC1 |  |  |  |
| EFNB2 | AP2B1 |  |  |  |
| BCOR | ATP6AP2 |  |  |  |
| HAS3 | B3GALNT1 |  |  |  |
| SMPD3 | BBS2 |  |  |  |
| HAS1 | BCAS3 |  |  |  |
| HIF3A | BST1 |  |  |  |
| HOXA11-AS | CD34 |  |  |  |
| MIR769 | CD3G |  |  |  |
| GASK1B-AS1 | CDK5RAP2 |  |  |  |
| LINC01030 | CDR2 |  |  |  |
| ENSG00000228401 | CLEC10A |  |  |  |
| COL7A1 | CLIP2 |  |  |  |
| NTRK2 | CMKLR1 |  |  |  |
| CASP8 | CNBP |  |  |  |
| CASR | DDAH2 |  |  |  |
| LDHA | DENND2B |  |  |  |
| PTH1R | DOCK11 |  |  |  |
| CDKN1A | EIF3B |  |  |  |
| HDAC3 | EXOSC5 |  |  |  |
| F7 | FETUB |  |  |  |
| ITGA3 | GCNT2 |  |  |  |
| TRPV1 | GRM4 |  |  |  |
| CYP27A1 | HACD4 |  |  |  |
| CYP2C8 | HNRNPH3 |  |  |  |
| IRF5 | HRH2 |  |  |  |
| WNT3A | HSPA13 |  |  |  |
| PRDM1 | IBSP |  |  |  |
| ULK1 | IL17RA |  |  |  |
| NLRP1 | IP6K2 |  |  |  |
| NLRP2 | JMJD1C |  |  |  |
| MCAM | LPCAT2 |  |  |  |
| NES | LTC4S |  |  |  |
| AFF4 | MANBA |  |  |  |
| KRT2 | MAPK8IP3 |  |  |  |
| SEMA3B | MECR |  |  |  |
| TANK | MEGF6 |  |  |  |
| CD177 | MFHAS1 |  |  |  |
| CXCL16 | MIR122 |  |  |  |
| DSC1 | MR1 |  |  |  |
| MAEA | ND5 |  |  |  |
| PLXNB1 | NHERF2 |  |  |  |
| CXCL14 | NLRP12 |  |  |  |
| RSPO4 | NPTN |  |  |  |
| MPP2 | NPTX2 |  |  |  |
| SEMA6B | NUP58 |  |  |  |
| OTUD1 | ODF2 |  |  |  |
| FER1L4 | OGFR |  |  |  |
| CASC15 | OTUB2 |  |  |  |
| MIR137HG | PAK1IP1 |  |  |  |
| NALT1 | PANX2 |  |  |  |
| MIR1469 | PDE1C |  |  |  |
| PNKY | PNPT1 |  |  |  |
| CDB2 | PPP1R2 |  |  |  |
| APOD | PPP3CC |  |  |  |
| TWIST2 | PRF1 |  |  |  |
| TMEM119 | PRKAB1 |  |  |  |
| DLL4 | PRR13 |  |  |  |
| NOTCH4 | PSCA |  |  |  |
| RBPJ | PXDN |  |  |  |
| DLL1 | R3HDM2 |  |  |  |
| JAG2 | RAB29 |  |  |  |
| LFNG | RABGAP1L |  |  |  |
| GCG | RERG |  |  |  |
| DLL3 | RGS19 |  |  |  |
| MAML1 | RNF128 |  |  |  |
| MAML3 | SCIN |  |  |  |
| MAML2 | SCN4A |  |  |  |
| HEYL | SERPINB8 |  |  |  |
| SAMD9L | SLC26A6 |  |  |  |
| TSR2 | SLC9A3 |  |  |  |
| MESP2 | SNRPG |  |  |  |
| LRRC66 | SRPRB |  |  |  |
| HDAC6 | SSR3 |  |  |  |
| PRKCD | STEAP2 |  |  |  |
| GLA | STXBP1 |  |  |  |
| KCNQ1 | TBCD |  |  |  |
| CDC25C | TBL1XR1 |  |  |  |
| CYLD | TFDP2 |  |  |  |
| IKBKG | TMEFF2 |  |  |  |
| CDH3 | TNXB |  |  |  |
| CDH5 | TTC28 |  |  |  |
| COL4A2 | UAP1 |  |  |  |
| IFNGR2 | UBASH3B |  |  |  |
| ACP1 | USP14 |  |  |  |
| SIX1 | ZDHHC2 |  |  |  |
| BMAL1 | AATK |  |  |  |
| BMP5 | ACBD4 |  |  |  |
| ITGA9 | ACSS3 |  |  |  |
| ST3GAL4 | AIMP2 |  |  |  |
| TGM3 | ANKRD33B |  |  |  |
| ELAVL1 | ARAF |  |  |  |
| PROKR2 | ARAP3 |  |  |  |
| MANF | ARHGEF4 |  |  |  |
| TP53BP2 | ARSG |  |  |  |
| BHLHE41 | ASAP1 |  |  |  |
| NAT10 | BMPR1A |  |  |  |
| SMG1 | BRD3 |  |  |  |
| SSH1 | BTD |  |  |  |
| YY1AP1 | C2CD2L |  |  |  |
| EEFSEC | CCRL2 |  |  |  |
| PRDM9 | CD5L |  |  |  |
| CGAS | CEL |  |  |  |
| IFNL3 | CLEC4E |  |  |  |
| MT-ND1 | CPEB1 |  |  |  |
| HOTTIP | DBT |  |  |  |
| MIR532 | DERA |  |  |  |
| MIR874 | DHTKD1 |  |  |  |
| MIR374B | DNAJC19 |  |  |  |
| MT-RNR1 | DOCK7 |  |  |  |
| CYP19A1 | DUSP7 |  |  |  |
| CPLANE1 | DYNC1LI2 |  |  |  |
| MAP2K2 | ECHDC1 |  |  |  |
| STAT6 | EPHB3 |  |  |  |
| EIF2AK3 | FEZ1 |  |  |  |
| EPHA4 | GALNT2 |  |  |  |
| PTK2B | GINS3 |  |  |  |
| SQSTM1 | GNE |  |  |  |
| CUL3 | GPC6 |  |  |  |
| NR2F2 | HAO1 |  |  |  |
| GABBR1 | HAP1 |  |  |  |
| DIAPH1 | HLTF |  |  |  |
| FGR | IL32 |  |  |  |
| SMAD6 | IPO4 |  |  |  |
| CHRNA5 | ITSN1 |  |  |  |
| DSC2 | JAM3 |  |  |  |
| EIF2S1 | LCP2 |  |  |  |
| FAP | LHPP |  |  |  |
| PRDX6 | LMO2 |  |  |  |
| PTH | LYSMD2 |  |  |  |
| CHRNB4 | MAN2A1 |  |  |  |
| FGF5 | MASTL |  |  |  |
| ZYX | MCM7 |  |  |  |
| DGKA | NAA15 |  |  |  |
| ID1 | ND2 |  |  |  |
| LGALS3BP | NKTR |  |  |  |
| RHD | NMT1 |  |  |  |
| ASIC3 | NSUN2 |  |  |  |
| TRPV2 | NXPH3 |  |  |  |
| ASRGL1 | OAS1A |  |  |  |
| TOMM20 | OPRL1 |  |  |  |
| TTF2 | OSBP2 |  |  |  |
| GSDMD | PITPNM1 |  |  |  |
| MYOM2 | PLCD3 |  |  |  |
| TRIM31 | PNISR |  |  |  |
| ITGA10 | PNPLA8 |  |  |  |
| TICAM2 | PPP1R13L |  |  |  |
| RLN2 | PRKRA |  |  |  |
| UCA1 | PROM1 |  |  |  |
| MIR152 | PRPF8 |  |  |  |
| MIR221 | PSMD13 |  |  |  |
| MIR96 | RASGEF1B |  |  |  |
| MIR106A | RIF1 |  |  |  |
| MIR181D | RIMS3 |  |  |  |
| MIR195 | RMDN2 |  |  |  |
| MIR137 | RNH1 |  |  |  |
| MIR505 | SAR1A |  |  |  |
| MIR18B | SCAMP5 |  |  |  |
| MIR1915 | SIX1 |  |  |  |
| MIR762 | SLC16A11 |  |  |  |
| MIR3659 | SLC29A3 |  |  |  |
| MIR4281 | SLC2A8 |  |  |  |
| CDR1-AS | SLC6A6 |  |  |  |
| MIR1973 | SOAT2 |  |  |  |
| FGF21 | SPG7 |  |  |  |
| MSN | ST6GALNAC2 |  |  |  |
| SERPINC1 | STT3A |  |  |  |
| CD151 | TAF4B |  |  |  |
| RUNX1 | TLE2 |  |  |  |
| IRF7 | TRERF1 |  |  |  |
| MIR106B | TRIM36 |  |  |  |
| MIR181B1 | UBE2D3 |  |  |  |
| MIR24-2 | UNKL |  |  |  |
| MIRLET7A3 | UTP20 |  |  |  |
| MIR181B2 | WNT2 |  |  |  |
| MIR451A | ACOT2 |  |  |  |
| MIRLET7A2 | AKNA |  |  |  |
| MIR19B1 | ATP6V0E2 |  |  |  |
| MIR372 | CD200 |  |  |  |
| MIR1260A | CDK5RAP3 |  |  |  |
| MIR486-2 | CKAP5 |  |  |  |
| GMDS | CLGN |  |  |  |
| LOC126859861 | CYP2A13 |  |  |  |
| BMP3 | DENND4A |  |  |  |
| PRPF8 | DISC1 |  |  |  |
| CXCL3 | DNPH1 |  |  |  |
| GPSM1 | EFTUD2 |  |  |  |
| TRC-GCA24-1 | EHBP1L1 |  |  |  |
| BRAF | EI24 |  |  |  |
| ITGB3 | ELMO1 |  |  |  |
| ABCG2 | EPB41L4B |  |  |  |
| HK1 | EPHB6 |  |  |  |
| PRKCA | F5 |  |  |  |
| TRPC6 | FLRT3 |  |  |  |
| TUBB | FYB1 |  |  |  |
| CACNA1A | GLRX2 |  |  |  |
| GFAP | GNA13 |  |  |  |
| MAPK9 | GPER1 |  |  |  |
| ROR2 | HTATSF1 |  |  |  |
| ENO2 | IL22 |  |  |  |
| LRP6 | IRAK4 |  |  |  |
| CYP24A1 | ITPRIP |  |  |  |
| KLK3 | LTBP3 |  |  |  |
| MSTN | LYNX1 |  |  |  |
| SOX2 | OCIAD2 |  |  |  |
| SPRY2 | PLXNB1 |  |  |  |
| DDR1 | PRPF40A |  |  |  |
| HCN2 | RBCK1 |  |  |  |
| LPAR1 | RGL1 |  |  |  |
| NR1H2 | RPL36A |  |  |  |
| SOX10 | RTKN |  |  |  |
| HES1 | SATB2 |  |  |  |
| INHBA | SERPINA10 |  |  |  |
| KPNA2 | SH3D19 |  |  |  |
| CALB1 | SLC17A5 |  |  |  |
| CANT1 | SLC29A1 |  |  |  |
| FOXM1 | SLC39A4 |  |  |  |
| FZD9 | SLC6A9 |  |  |  |
| GAP43 | SLCO1B1 |  |  |  |
| MMP10 | SMS |  |  |  |
| TAGLN | SMYD2 |  |  |  |
| CFHR1 | STK38 |  |  |  |
| SDC2 | TDRD7 |  |  |  |
| HYAL2 | TEX264 |  |  |  |
| ZBP1 | TMED9 |  |  |  |
| DGKQ | TNNT2 |  |  |  |
| IL17B | TRAF4 |  |  |  |
| RSPO2 | TTC3 |  |  |  |
| DGKB | VPS13D |  |  |  |
| IL19 | VRK1 |  |  |  |
| LIN28A | WSB2 |  |  |  |
| SPAM1 | ABTB3 |  |  |  |
| CD200 | ACTR2 |  |  |  |
| COL14A1 | AP3D1 |  |  |  |
| FUT4 | APMAP |  |  |  |
| IL25 | B4GALNT1 |  |  |  |
| KLF2 | BACE2 |  |  |  |
| KPNA4 | BAG1 |  |  |  |
| PELI1 | BEX1 |  |  |  |
| ARHGAP17 | CBFA2T3 |  |  |  |
| CCN3 | CCNI |  |  |  |
| DGKD | CD79B |  |  |  |
| DGKG | CLK3 |  |  |  |
| IER3 | DOCK9 |  |  |  |
| IFIT2 | EPS8L2 |  |  |  |
| IL17D | ERG |  |  |  |
| KPNA6 | ETV3 |  |  |  |
| NANOG | ETV6 |  |  |  |
| EMILIN1 | F2RL2 |  |  |  |
| SPRR3 | FGFRL1 |  |  |  |
| AIFM2 | FMN1 |  |  |  |
| CD200R1 | FOXG1 |  |  |  |
| L1TD1 | FOXP2 |  |  |  |
| USP12 | FRMD6 |  |  |  |
| SCRG1 | GAK |  |  |  |
| TMEM258 | GBA1 |  |  |  |
| CYTOR | GNG7 |  |  |  |
| MIRLET7I | H1-0 |  |  |  |
| MIR15B | HIRIP3 |  |  |  |
| MIR212 | IPO9 |  |  |  |
| MIRLET7E | KDM4A |  |  |  |
| MIR1207 | LARP7 |  |  |  |
| MIR2861 | LMAN1 |  |  |  |
| RNU6-1 | LMOD1 |  |  |  |
| MIR376B | LRRC20 |  |  |  |
| MIR3198-2 | MAFG |  |  |  |
| MIR630 | MALL |  |  |  |
| TRL-TAG1-1 | MANSC1 |  |  |  |
| F2R | MARK1 |  |  |  |
| MIR204 | MARK2 |  |  |  |
| MIR497 | MCOLN2 |  |  |  |
| MIR650 | MFF |  |  |  |
| MIR3141 | MGAM |  |  |  |
| MIR4271 | MKNK1 |  |  |  |
| FABP4 | MRPL23 |  |  |  |
| MX1 | MSI2 |  |  |  |
| RAPGEF3 | MSX1 |  |  |  |
| SETDB1 | MVB12B |  |  |  |
| TLL1 | NHSL1 |  |  |  |
| CD3G | NOD1 |  |  |  |
| PSMC3IP | NOL6 |  |  |  |
| TNRC6A | NPEPPS |  |  |  |
| BICD1 | NRM |  |  |  |
| SSR2 | NUMA1 |  |  |  |
| UHMK1 | OLFML1 |  |  |  |
| UBAC1 | PBX3 |  |  |  |
| GPSM3 | PCYT2 |  |  |  |
| EGFL8 | PFAS |  |  |  |
| SMIM20 | PITX2 |  |  |  |
| PRKACA | PLXNB2 |  |  |  |
| RDX | PSTPIP1 |  |  |  |
| EZR | RAB27B |  |  |  |
| HRG | RAB5C |  |  |  |
| RAB5A | RENBP |  |  |  |
| PRF1 | SCAP |  |  |  |
| CCL4 | SEC16A |  |  |  |
| KDM5B | SENP6 |  |  |  |
| SKP2 | SHROOM3 |  |  |  |
| MIR5100 | SLC44A3 |  |  |  |
| MIR4454 | SMTN |  |  |  |
| PDGFD | SP110 |  |  |  |
| GATA2 | SPCS2 |  |  |  |
| KITLG | SPI1 |  |  |  |
| STXBP2 | SPOCK1 |  |  |  |
| KCNC4 | SPRED2 |  |  |  |
| UNC13D | SRI |  |  |  |
| SIGLEC8 | SRPX2 |  |  |  |
| STX11 | STAB1 |  |  |  |
| HPS4 | SUN2 |  |  |  |
| KRT3 | THPO |  |  |  |
| RAPGEF5 | TNFSF13 |  |  |  |
| PLA2G4E | TRIM47 |  |  |  |
| FAHD2A | TRIM6 |  |  |  |
| CCDC85C | UGT2B17 |  |  |  |
| SLC35D2 | USP24 |  |  |  |
| SLC35E3 | VAPA |  |  |  |
| STX19 | XRN2 |  |  |  |
| MIR4492 | ZBTB44 |  |  |  |
| MIR3187 | ACYP2 |  |  |  |
| MIR1268B | ADAMTS15 |  |  |  |
| hsa-miR-1273d-001 | APBB3 |  |  |  |
| ACTB | ARIH2 |  |  |  |
| TNC | ARSB |  |  |  |
| MS4A1 | ATP13A3 |  |  |  |
| LINC00184 | BTC |  |  |  |
| KDM6A | BUB3 |  |  |  |
| CLSPN | C1GALT1C1 |  |  |  |
| TOB2 | CAPN3 |  |  |  |
| MIR375 | CDX2 |  |  |  |
| TCF7L2 | CES2C |  |  |  |
| HABP2 | CHST14 |  |  |  |
| PTPRS | CKLF |  |  |  |
| NR2E3 | CLUH |  |  |  |
| U2AF2 | CNTNAP1 |  |  |  |
| ZFP28 | CYP3A9 |  |  |  |
| ZNF582 | DCTD |  |  |  |
| ZNF667 | DNA2 |  |  |  |
| ZNF471 | DNAJB5 |  |  |  |
| NAT14 | DRD5 |  |  |  |
| ZNF470 | EAF2 |  |  |  |
| ZNF580 | EBF3 |  |  |  |
| ZNF667-AS1 | EIF5A2 |  |  |  |
| ZNF582-DT | ERC2 |  |  |  |
| ZNF542P | ESYT1 |  |  |  |
| RPS3AP21 | G2E3 |  |  |  |
| ZNF470-DT | GALNT12 |  |  |  |
| DEFA9P | GLA |  |  |  |
| DEFA10P | GM2A |  |  |  |
| ENSG00000233547 | H2BC21 |  |  |  |
| ENSG00000266907 | IGSF11 |  |  |  |
| lnc-UHRF1-3 | INPP4B |  |  |  |
| HSALNG0123439 | JPH2 |  |  |  |
| HSALNG0144561 | KCNS3 |  |  |  |
| LOC107986777 | LATS2 |  |  |  |
| piR-43976 | LGALS4 |  |  |  |
| LOC124903521 | LRATD2 |  |  |  |
| GLUD1 | LRBA |  |  |  |
| HCCAT5 | LSM6 |  |  |  |
| GAD1 | LY6A |  |  |  |
| PEX1 | MAS1 |  |  |  |
| CNNM4 | MCFD2 |  |  |  |
| ODAPH | MGAT4B |  |  |  |
| RASA4 | MLF1 |  |  |  |
| MIR193A | MOB3B |  |  |  |
| MIR302A | MOCS1 |  |  |  |
| DNM3OS | MSRB2 |  |  |  |
| MIR575 | NAB1 |  |  |  |
| MIR4793 | NBR1 |  |  |  |
| MIR4781 | NFKBID |  |  |  |
| MIR6088 | NKD2 |  |  |  |
| PRH1-PRR4 | NMB |  |  |  |
| FADD | NOL8 |  |  |  |
| HERC2 | NPL |  |  |  |
| PYCR1 | PAIP1 |  |  |  |
| PFAS | PBX2 |  |  |  |
| ATP6V0A2 | PJA2 |  |  |  |
| NDN | PNKP |  |  |  |
| MKRN3 | PRR11 |  |  |  |
| MAGEL2 | PSMC3IP |  |  |  |
| ENOSF1 | PSPC1 |  |  |  |
| RTEL1-TNFRSF6B | PWWP3A |  |  |  |
| IPW | RAB40B |  |  |  |
| SNORD115-1 | RALBP1 |  |  |  |
| SNORD116-1 | RCBTB2 |  |  |  |
| GORAB-AS1 | RFX5 |  |  |  |
| MKRN3-AS1 | RPL39 |  |  |  |
| LOC126806063 | SEC16B |  |  |  |
| OTDD | SH3GL2 |  |  |  |
| SPI1 | SHROOM2 |  |  |  |
| SRC | SKA2 |  |  |  |
| AQP5 | SLC12A2 |  |  |  |
| CYP1B1 | SSBP3 |  |  |  |
| AHR | SURF4 |  |  |  |
| HPX | TBC1D5 |  |  |  |
| IL18BP | TMEM135 |  |  |  |
| MET | TNRC18 |  |  |  |
| CCR3 | TPD52L2 |  |  |  |
| DNAJC3-DT | TRAF3IP2 |  |  |  |
| ARHGEF5 | TREX1 |  |  |  |
| VAMP2 | TRMT1 |  |  |  |
| MIR663A | UBR4 |  |  |  |
| ITGB4 | UNC93B1 |  |  |  |
| COMP | WDR62 |  |  |  |
| SERPINF2 | ACVR1C |  |  |  |
| DST | ADAM15 |  |  |  |
| KRT15 | AKAP8 |  |  |  |
| ATF6B | ALDH6A1 |  |  |  |
| GDPD2 | ATL3 |  |  |  |
| OR1E1 | BOC |  |  |  |
| PLGRKT | C1QTNF4 |  |  |  |
| SPINK13 | CAMK1 |  |  |  |
| ZNF98 | CD19 |  |  |  |
| LOC130065404 | CD247 |  |  |  |
| XRCC6 | CD84 |  |  |  |
| CLCN5 | CDH22 |  |  |  |
| CCK | CDH8 |  |  |  |
| GORASP1 | CETN2 |  |  |  |
| IL15 | CHRNB1 |  |  |  |
| F5 | CKMT2 |  |  |  |
| CXCL11 | CMTM6 |  |  |  |
| CDH11 | COPG1 |  |  |  |
| ISG15 | CTDSP2 |  |  |  |
| HBB | CTSF |  |  |  |
| COL6A3 | DCHS1 |  |  |  |
| CSF2RB | DEF6 |  |  |  |
| TCF12 | EIF4H |  |  |  |
| BAMBI | ELMOD1 |  |  |  |
| COL11A1 | EPDR1 |  |  |  |
| ENTPD3 | FAM111B |  |  |  |
| RBX1 | FOXF1 |  |  |  |
| WIF1 | GNG5 |  |  |  |
| DNASE1L3 | GOLGA4 |  |  |  |
| LITAF | HCRT |  |  |  |
| TCF7 | HDHD3 |  |  |  |
| COL10A1 | HERC2 |  |  |  |
| SEMA5A | HJURP |  |  |  |
| ADAMTS3 | HOGA1 |  |  |  |
| TNFRSF21 | HPCAL4 |  |  |  |
| FFAR2 | HRG |  |  |  |
| COL8A2 | KCTD15 |  |  |  |
| EXTL2 | KCTD5 |  |  |  |
| AOC2 | LRP6 |  |  |  |
| OMD | LRRC17 |  |  |  |
| CLEC11A | LYAR |  |  |  |
| COQ10B | MARCHF6 |  |  |  |
| PCOLCE | MCUB |  |  |  |
| FOXF2 | MGAT1 |  |  |  |
| RGS3 | MGAT5 |  |  |  |
| CHAD | MLLT6 |  |  |  |
| SIGLEC15 | MREG |  |  |  |
| STAC | MTDH |  |  |  |
| COPZ2 | NCK1 |  |  |  |
| GFRA4 | NPEPL1 |  |  |  |
| GNG11 | NUP37 |  |  |  |
| KDELR3 | PAQR5 |  |  |  |
| LRRC15 | PAQR8 |  |  |  |
| LRRC17 | PDSS1 |  |  |  |
| RUBCNL | PIP4K2A |  |  |  |
| CLEC2B | PLVAP |  |  |  |
| G0S2 | POLG2 |  |  |  |
| OLFML2B | PPID |  |  |  |
| C1orf54 | PPM1A |  |  |  |
| KRTAP5-8 | PPP1R18 |  |  |  |
| RPS4XP9 | PRPS1 |  |  |  |
| RPL18AP10 | PSME3 |  |  |  |
| PPIAP21 | RAB18 |  |  |  |
| RPL18AP16 | RCAN1 |  |  |  |
| ANTXR1 | RETNLA |  |  |  |
| PTGFR | RIN3 |  |  |  |
| TNFSF14 | RNF144A |  |  |  |
| PIN1 | SEMA5B |  |  |  |
| CD209 | SGTB |  |  |  |
| SEMA7A | SLC45A4 |  |  |  |
| PLAC8 | SNRPE |  |  |  |
| IRS1 | SPSB4 |  |  |  |
| GJA5 | SRR |  |  |  |
| GJA4 | TCEA2 |  |  |  |
| KRT7 | TCF3 |  |  |  |
| RNY1 | TNKS |  |  |  |
| ENSG00000202141 | TOM1 |  |  |  |
| PGF | TYRP1 |  |  |  |
| CFTR | UNC13B |  |  |  |
| GDNF | UNC13D |  |  |  |
| ANG | UNC5C |  |  |  |
| FGF4 | USO1 |  |  |  |
| CD24 | WDR46 |  |  |  |
| ERBB2 | WIF1 |  |  |  |
| CCND1 | WNT3 |  |  |  |
| AR | ZBTB4 |  |  |  |
| PRKAR1A | AGFG2 |  |  |  |
| PGR | AK5 |  |  |  |
| COL2A1 | ARGLU1 |  |  |  |
| CYP17A1 | ARMC9 |  |  |  |
| NR5A1 | ARPC5L |  |  |  |
| STXBP1 | ATN1 |  |  |  |
| CHRM3 | BMS1 |  |  |  |
| PRKACB | CCL26 |  |  |  |
| SPTAN1 | CDC37L1 |  |  |  |
| CYP11A1 | CDC42EP3 |  |  |  |
| CYP2D6 | CDC42SE1 |  |  |  |
| ESRRB | CEP152 |  |  |  |
| LOX | CPNE2 |  |  |  |
| NCOA3 | CRHR2 |  |  |  |
| NF1 | CRYBG1 |  |  |  |
| PIK3CB | DCAF11 |  |  |  |
| SLC4A1 | DDX23 |  |  |  |
| TTN | DDX46 |  |  |  |
| THBS2 | DENND3 |  |  |  |
| TP63 | DEXI |  |  |  |
| PRKACG | DIAPH1 |  |  |  |
| SKI | DNAH1 |  |  |  |
| COL6A1 | DNMT3L |  |  |  |
| CXADR | ECHDC3 |  |  |  |
| NCOA1 | ESRRA |  |  |  |
| NCOA2 | FAM3C |  |  |  |
| NCOR1 | FASTKD2 |  |  |  |
| PLOD2 | FNDC3A |  |  |  |
| PLOD3 | FRYL |  |  |  |
| PRL | FTSJ3 |  |  |  |
| PXDN | GABRD |  |  |  |
| RARS1 | GASK1B |  |  |  |
| SNAI1 | GLIS3 |  |  |  |
| STAR | GUCY1A2 |  |  |  |
| CFP | HAS1 |  |  |  |
| CNGA3 | HMGCL |  |  |  |
| CYP21A2 | HOOK2 |  |  |  |
| ESRRG | IGSF6 |  |  |  |
| HSD3B1 | LINGO1 |  |  |  |
| SEPSECS | LSR |  |  |  |
| SLC13A5 | MAP3K3 |  |  |  |
| SLC2A10 | METTL14 |  |  |  |
| SLC39A14 | MGST3 |  |  |  |
| ADAMTS10 | MIR221 |  |  |  |
| HSD17B2 | MTARC2 |  |  |  |
| SATB1 | NCSTN |  |  |  |
| SHBG | NECAB1 |  |  |  |
| TNR | NETO2 |  |  |  |
| CRTAP | NUP153 |  |  |  |
| FKBP10 | PAQR4 |  |  |  |
| HCCS | PATJ |  |  |  |
| HSD17B1 | PAX5 |  |  |  |
| P3H1 | PBRM1 |  |  |  |
| PEX6 | PDE8B |  |  |  |
| SLC7A1 | PDP2 |  |  |  |
| TBX4 | POU2AF1 |  |  |  |
| COL12A1 | PPP1R1B |  |  |  |
| GPER1 | PSTPIP2 |  |  |  |
| KISS1 | RCOR3 |  |  |  |
| ADAMTSL2 | RIN1 |  |  |  |
| DNAL1 | RND2 |  |  |  |
| LUM | RPL38 |  |  |  |
| NISCH | RTN2 |  |  |  |
| SERPINA12 | SEC62 |  |  |  |
| PIEZO2 | SF3B1 |  |  |  |
| SERPINA10 | SHC3 |  |  |  |
| CKAP4 | SIRT5 |  |  |  |
| FAM20B | SLC6A11 |  |  |  |
| GREB1 | SMAD5 |  |  |  |
| LZTS1 | SNAP23 |  |  |  |
| VSX1 | SPIN1 |  |  |  |
| ACCS | SPOCK2 |  |  |  |
| COL5A3 | STK26 |  |  |  |
| DHRS11 | STRN |  |  |  |
| P3H3 | SUPT16H |  |  |  |
| SCG5 | SYCP3 |  |  |  |
| SLC10A7 | SYT2 |  |  |  |
| ROGDI | TERF1 |  |  |  |
| ACP4 | TMEM106C |  |  |  |
| PXDNL | TNNT3 |  |  |  |
| TSPOAP1 | TOR1AIP1 |  |  |  |
| MIER1 | TSG101 |  |  |  |
| AMELY | TULP4 |  |  |  |
| TNXA | VHL |  |  |  |
| MIR378A | XPNPEP1 |  |  |  |
| MIR324 | XPO5 |  |  |  |
| MIR224 | YTHDF2 |  |  |  |
| SNORA66 | ZPR1 |  |  |  |
| COL5A1-AS1 | ABHD6 |  |  |  |
| CYP21A1P | ADAMTSL2 |  |  |  |
| MIR3609 | ADCY4 |  |  |  |
| FKBP14-AS1 | AFF3 |  |  |  |
| MIR3914-1 | AKIRIN1 |  |  |  |
| MIR3195 | AMOT |  |  |  |
| MIR3606 | AMPH |  |  |  |
| MMP20-AS1 | ANKFY1 |  |  |  |
| MIR3914-2 | ANO10 |  |  |  |
| MIR548AX | APBB1 |  |  |  |
| LOC107988032 | ARF3 |  |  |  |
| LOC126862586 | ARHGAP31 |  |  |  |
| LOC121832793 | ATG16L1 |  |  |  |
| LOC129995400 | B4GALT2 |  |  |  |
| LOC130006027 | BORA |  |  |  |
| LOC130006030 | CAB39 |  |  |  |
| LOC130006032 | CDC42EP5 |  |  |  |
| LOC130056851 | CEP85 |  |  |  |
| LOC130061152 | CHIL3 |  |  |  |
| LOC130061153 | CHP1 |  |  |  |
| LOC130061154 | CLIC6 |  |  |  |
| AIH3 | CLK4 |  |  |  |
| LOC106780804 | CNTF |  |  |  |
| LOC112997581 | CPXM1 |  |  |  |
| LOC130002964 | DDX54 |  |  |  |
| LOC130067939 | DGKI |  |  |  |
| MSMB | DLG3 |  |  |  |
| DEFB104A | DOK3 |  |  |  |
| CD38 | DPF3 |  |  |  |
| ADAMTS5 | EIF3D |  |  |  |
| IL12RB1 | EVA1B |  |  |  |
| KRT17 | FAM222A |  |  |  |
| LCP1 | FAM53B |  |  |  |
| CD59 | GDF9 |  |  |  |
| FAM201A | GORASP2 |  |  |  |
| MDM2 | GPRIN3 |  |  |  |
| ATM | HK3 |  |  |  |
| CSNK2A1 | IFRD2 |  |  |  |
| FANCA | INS2 |  |  |  |
| GNAS | KAT6B |  |  |  |
| PCSK1 | KCNIP3 |  |  |  |
| PRKDC | LARP1B |  |  |  |
| RAD51 | LAT2 |  |  |  |
| GABRB3 | MAZ |  |  |  |
| RAD50 | MCC |  |  |  |
| SCN8A | MED13L |  |  |  |
| BRIP1 | NAT8L |  |  |  |
| PIKFYVE | NUDT12 |  |  |  |
| SDHA | OPCML |  |  |  |
| TYR | P2RX2 |  |  |  |
| UBE3A | PAPOLA |  |  |  |
| FANCC | PCYOX1L |  |  |  |
| GABRA5 | PHKB |  |  |  |
| GALK1 | PHOSPHO1 |  |  |  |
| GRK2 | PIDD1 |  |  |  |
| INPPL1 | PITPNC1 |  |  |  |
| LIG4 | PLEKHA1 |  |  |  |
| MPL | PPP2R2A |  |  |  |
| MTR | PRODH2 |  |  |  |
| OXTR | PRPSAP1 |  |  |  |
| POMC | PRTN3 |  |  |  |
| RPA1 | PSMD5 |  |  |  |
| RPL11 | PTPRN2 |  |  |  |
| RPL5 | PUM3 |  |  |  |
| RPS19 | PUS1 |  |  |  |
| SCN1A | RASA4 |  |  |  |
| WRN | RCAN3 |  |  |  |
| FANCD2 | RHOBTB2 |  |  |  |
| GATA1 | RHOF |  |  |  |
| GHR | RSL24D1 |  |  |  |
| MC4R | RTN1 |  |  |  |
| MECP2 | SBSN |  |  |  |
| MVK | SFRP1 |  |  |  |
| PIP5K1C | SH3RF3 |  |  |  |
| SH2D1A | SLC14A2 |  |  |  |
| TYRP1 | SLC25A19 |  |  |  |
| ACOX1 | SMAGP |  |  |  |
| ARF1 | SNHG1 |  |  |  |
| ARRB2 | STARD10 |  |  |  |
| CD34 | STYXL1 |  |  |  |
| CEBPA | SYT17 |  |  |  |
| CUBN | TBX15 |  |  |  |
| DLK1 | TLE4 |  |  |  |
| ETV6 | TMC7 |  |  |  |
| FHL2 | TNFRSF10D |  |  |  |
| GRK6 | TPP2 |  |  |  |
| LSS | WASF3 |  |  |  |
| NAA10 | YARS2 |  |  |  |
| PI4KB | ZHX3 |  |  |  |
| RAB11A | ACOX3 |  |  |  |
| SAG | APOLD1 |  |  |  |
| SYNJ1 | ARHGEF9 |  |  |  |
| TET2 | ATP8A1 |  |  |  |
| ARRB1 | AUTS2 |  |  |  |
| ASXL1 | CARD6 |  |  |  |
| ATP6V1A | CASP8AP2 |  |  |  |
| ATP6V1E1 | CCDC69 |  |  |  |
| DCT | CCDC92 |  |  |  |
| FMR1 | CELSR1 |  |  |  |
| HTR2C | CENPI |  |  |  |
| IGFBP2 | CLEC4D |  |  |  |
| METAP2 | COPS5 |  |  |  |
| MYO5A | COPZ2 |  |  |  |
| PSMA7 | DARS2 |  |  |  |
| RUVBL1 | EFR3B |  |  |  |
| TP53BP1 | EIF2AK4 |  |  |  |
| XRCC5 | EIF3J |  |  |  |
| APPL1 | FANCG |  |  |  |
| COPB2 | FLII |  |  |  |
| DDX41 | H2-AB1 |  |  |  |
| FANCG | HEXIM1 |  |  |  |
| FANCI | HLA-C |  |  |  |
| GABRG3 | HYKK |  |  |  |
| GH1 | IKBIP |  |  |  |
| GRK3 | IMPDH1 |  |  |  |
| LAMP1 | KIRREL1 |  |  |  |
| NAGLU | LARP4B |  |  |  |
| NOP56 | LY86 |  |  |  |
| PEX19 | MAFA |  |  |  |
| POLI | MAN2A2 |  |  |  |
| PUS1 | MAP2K7 |  |  |  |
| RPS10 | METTL1 |  |  |  |
| RPS24 | MN1 |  |  |  |
| RPS26 | MRPL38 |  |  |  |
| SCN11A | MTA2 |  |  |  |
| SEC61A1 | MTAP |  |  |  |
| SEC63 | MYBPC1 |  |  |  |
| SOD3 | NAAA |  |  |  |
| THPO | NFYA |  |  |  |
| ARHGAP1 | NOC2L |  |  |  |
| DDX11 | NPHS2 |  |  |  |
| FANCE | NSL1 |  |  |  |
| FANCM | NT5C2 |  |  |  |
| FBL | PATL1 |  |  |  |
| GPR143 | PELI2 |  |  |  |
| GPX7 | PEX13 |  |  |  |
| GRB10 | PNRC2 |  |  |  |
| GZMA | POLR1H |  |  |  |
| INPP5K | POLR3G |  |  |  |
| LTBP4 | POU2F2 |  |  |  |
| PAPOLA | PRKAG1 |  |  |  |
| PEX14 | RBFOX2 |  |  |  |
| RPL15 | RIMS1 |  |  |  |
| RPL26 | RUSC2 |  |  |  |
| RPL35A | SCOC |  |  |  |
| RPS17 | SCUBE2 |  |  |  |
| RPS3 | SETD4 |  |  |  |
| RUVBL2 | SETD7 |  |  |  |
| SEC23A | SGSM3 |  |  |  |
| ACD | SNRPD2 |  |  |  |
| ACKR3 | SOX5 |  |  |  |
| ATP10A | STAC2 |  |  |  |
| COIL | STN1 |  |  |  |
| EIF3F | SUMO2 |  |  |  |
| GPX2 | SV2B |  |  |  |
| HPS1 | TAF13 |  |  |  |
| INPP5E | TEAD2 |  |  |  |
| LAMTOR2 | TIGAR |  |  |  |
| MAGED1 | TMED5 |  |  |  |
| NBEAL2 | TMEM43 |  |  |  |
| PIGK | TMEM64 |  |  |  |
| PLAGL1 | TMEM88 |  |  |  |
| POLR1D | TNFAIP1 |  |  |  |
| POT1 | TNRC6A |  |  |  |
| PSMD8 | TPST2 |  |  |  |
| PYY | TTC7B |  |  |  |
| RAB6A | TTPAL |  |  |  |
| RECQL4 | UBE2O |  |  |  |
| RPS14 | VASH1 |  |  |  |
| RPS20 | WDR77 |  |  |  |
| RPS23 | WIPF3 |  |  |  |
| SFTPC | AEBP2 |  |  |  |
| SHANK3 | AKAP6 |  |  |  |
| SLC45A2 | ANKRD22 |  |  |  |
| TAFAZZIN | BCL2L14 |  |  |  |
| TCOF1 | BLK |  |  |  |
| TERF1 | CACHD1 |  |  |  |
| TMC8 | CASQ2 |  |  |  |
| TRIP11 | CBX3 |  |  |  |
| VPS13B | CCDC50 |  |  |  |
| ANKRD26 | CCNO |  |  |  |
| CYFIP1 | CENPT |  |  |  |
| DNAJC3 | CES1F |  |  |  |
| EIF6 | CHD8 |  |  |  |
| FANCB | CIZ1 |  |  |  |
| GOLGA2 | CLCA1 |  |  |  |
| HPS5 | CPB1 |  |  |  |
| INPP4A | CRADD |  |  |  |
| MLPH | CTTN |  |  |  |
| PEX3 | CYP2C23 |  |  |  |
| PSMD6 | DHX37 |  |  |  |
| RAB33B | DLX2 |  |  |  |
| RAB8A | DNAJB2 |  |  |  |
| RPS29 | DPAGT1 |  |  |  |
| SIM1 | DTX1 |  |  |  |
| SRP72 | DUSP14 |  |  |  |
| TERF2 | DYNC1I2 |  |  |  |
| AGRP | EEF1E1 |  |  |  |
| ARF5 | EID1 |  |  |  |
| HCRT | EPHA5 |  |  |  |
| HLA-DMA | FAM171A1 |  |  |  |
| HPS6 | FAM171B |  |  |  |
| INPP5B | FCGR2A |  |  |  |
| INPP5J | FKBP9 |  |  |  |
| NIPA1 | GABRE |  |  |  |
| NMB | GJC1 |  |  |  |
| PUS7 | GNPAT |  |  |  |
| RAB35 | H4C16 |  |  |  |
| RIN2 | HAVCR2 |  |  |  |
| RPL22 | HLA-DMA |  |  |  |
| RPL31 | HOMER3 |  |  |  |
| STN1 | HOXB5 |  |  |  |
| AP3S1 | HSPBP1 |  |  |  |
| AP4M1 | IL15RA |  |  |  |
| BLOC1S6 | ILF2 |  |  |  |
| DIO3 | ISLR |  |  |  |
| EFL1 | KIF3C |  |  |  |
| EMG1 | KRTCAP2 |  |  |  |
| ERCC6L2 | LAPTM4B |  |  |  |
| EXOSC10 | LCP1 |  |  |  |
| GAR1 | LDLRAD3 |  |  |  |
| GHRH | LHX1 |  |  |  |
| GOLPH3 | LMO1 |  |  |  |
| GPX5 | M6PR |  |  |  |
| GPX8 | MARCHF2 |  |  |  |
| GSTK1 | MARCHF3 |  |  |  |
| HYDIN | MGAT4A |  |  |  |
| MEST | MRPL54 |  |  |  |
| NHLH2 | MYO1F |  |  |  |
| NOLC1 | NADK2 |  |  |  |
| OXT | NANOS1 |  |  |  |
| PAPOLG | NCAM1 |  |  |  |
| PNLDC1 | NCKAP1L |  |  |  |
| RBFOX2 | P2RY14 |  |  |  |
| RPS28 | PAPSS2 |  |  |  |
| SFTPA2 | PEG3 |  |  |  |
| SHOX | PKP1 |  |  |  |
| SLX4 | PLCB2 |  |  |  |
| SNX9 | POLR1B |  |  |  |
| TERF2IP | PPAN |  |  |  |
| TOE1 | PTK7 |  |  |  |
| CLPTM1L | RAB3C |  |  |  |
| COG7 | RCC1 |  |  |  |
| COPE | REG3G |  |  |  |
| COPS8 | RIPK4 |  |  |  |
| GH2 | RNASE4 |  |  |  |
| NIPA2 | RPL37A |  |  |  |
| RPS15 | RSU1 |  |  |  |
| SCG2 | SCG3 |  |  |  |
| SNU13 | SCN7A |  |  |  |
| SYNJ2 | SDC2 |  |  |  |
| TGOLN2 | SEC22B |  |  |  |
| ZCCHC8 | SEMA4F |  |  |  |
| AP4E1 | SFT2D2 |  |  |  |
| COPS7A | SFTPA1 |  |  |  |
| CSH1 | SH3RF2 |  |  |  |
| DCLRE1B | SH3TC2 |  |  |  |
| EIF3K | SIX4 |  |  |  |
| GOLGA1 | SLC7A3 |  |  |  |
| GORASP2 | SMARCB1 |  |  |  |
| MTREX | SPTA1 |  |  |  |
| NOP58 | STIL |  |  |  |
| PEG3 | STK40 |  |  |  |
| PLEK2 | TAGLN3 |  |  |  |
| RPL38 | TBC1D9 |  |  |  |
| SASS6 | TMEM154 |  |  |  |
| SCYL2 | TMEM156 |  |  |  |
| TRO | TNS4 |  |  |  |
| BLOC1S3 | TP53BP2 |  |  |  |
| COPS6 | TSHZ1 |  |  |  |
| GOLGA5 | TUBGCP3 |  |  |  |
| GOLGB1 | UGGT2 |  |  |  |
| LRMDA | UGT2A1 |  |  |  |
| PEX11A | VDAC3 |  |  |  |
| PIF1 | WSCD1 |  |  |  |
| SAMD9 | XPO7 |  |  |  |
| SEC62 | YES1 |  |  |  |
| SHQ1 | ZFPM1 |  |  |  |
| TENT4B | ACOT3 |  |  |  |
| TRUB1 | ALOXE3 |  |  |  |
| TUBGCP5 | APOBEC3B |  |  |  |
| BHLHE22 | ARFGAP2 |  |  |  |
| COPS4 | ARHGEF1 |  |  |  |
| GPX6 | ARK2C |  |  |  |
| NAF1 | ATF1 |  |  |  |
| PXMP2 | BAZ2B |  |  |  |
| SSR3 | BCAP31 |  |  |  |
| UTP4 | BCAS2 |  |  |  |
| ATP5MG | BCL2L10 |  |  |  |
| PAPOLB | CAMSAP1 |  |  |  |
| TRUB2 | CAMSAP2 |  |  |  |
| CSH2 | CAPZB |  |  |  |
| FHDC1 | CARMIL1 |  |  |  |
| PHETA1 | CASZ1 |  |  |  |
| SEC61A2 | CCDC77 |  |  |  |
| ZNF622 | CD6 |  |  |  |
| MBOAT4 | CDCA4 |  |  |  |
| TEN1 | CHKB |  |  |  |
| POM121L12 | CIB2 |  |  |  |
| RMRP | CLPP |  |  |  |
| RNU4ATAC | CLTA |  |  |  |
| SNORA64 | CTNS |  |  |  |
| SCARNA8 | DDX11 |  |  |  |
| SCARNA4 | DERL1 |  |  |  |
| SNORA15 | DGKZ |  |  |  |
| SNORA48 | DUSP9 |  |  |  |
| MIMT1 | DYRK1A |  |  |  |
| PWAR5 | EDEM3 |  |  |  |
| SCARNA23 | EIF2B1 |  |  |  |
| SNORA24 | ENY2 |  |  |  |
| SNORA67 | EXOC4 |  |  |  |
| SNORD108 | FGFR4 |  |  |  |
| SNORD64 | FKBP2 |  |  |  |
| SNORD109A | GBE1 |  |  |  |
| SNORD109B | GDF6 |  |  |  |
| SNORA35 | GKAP1 |  |  |  |
| SNORD107 | GLCCI1 |  |  |  |
| LINC02250 | GRAMD1A |  |  |  |
| LOC124902337 | GTF2B |  |  |  |
| PWARSN | H1F2 |  |  |  |
| SNORD115-10 | ITGB7 |  |  |  |
| SNORD115-2 | IWS1 |  |  |  |
| SNORD115-48 | KCNH2 |  |  |  |
| SNORD116-12 | KLHDC8B |  |  |  |
| SNORD116-2 | L2HGDH |  |  |  |
| SNORD116-25 | LIPE |  |  |  |
| SNORD116-26 | LPCAT4 |  |  |  |
| SNORD116-27 | LRATD1 |  |  |  |
| SNORD116-29 | ME2 |  |  |  |
| SNORD116-3 | MIR22 |  |  |  |
| SNORD115-11 | MKLN1 |  |  |  |
| SNORD115-12 | MOCS2 |  |  |  |
| SNORD115-13 | MPV17L |  |  |  |
| SNORD115-14 | MRTO4 |  |  |  |
| SNORD115-15 | NACA |  |  |  |
| SNORD115-16 | NAPG |  |  |  |
| SNORD115-17 | NLN |  |  |  |
| SNORD115-18 | NOB1 |  |  |  |
| SNORD115-19 | OS9 |  |  |  |
| SNORD115-20 | OVOL1 |  |  |  |
| SNORD115-21 | PBXIP1 |  |  |  |
| SNORD115-22 | PEAR1 |  |  |  |
| SNORD115-23 | PFDN2 |  |  |  |
| SNORD115-25 | PI16 |  |  |  |
| SNORD115-26 | PI4KA |  |  |  |
| SNORD115-29 | PLEKHO2 |  |  |  |
| SNORD115-3 | PMS2 |  |  |  |
| SNORD115-30 | PPP1R9B |  |  |  |
| SNORD115-31 | PRPF38B |  |  |  |
| SNORD115-32 | PRSS35 |  |  |  |
| SNORD115-33 | RAP1B |  |  |  |
| SNORD115-34 | RFX7 |  |  |  |
| SNORD115-35 | RNF26 |  |  |  |
| SNORD115-36 | RNF4 |  |  |  |
| SNORD115-37 | ROPN1L |  |  |  |
| SNORD115-38 | RPAP3 |  |  |  |
| SNORD115-39 | SAP30BP |  |  |  |
| SNORD115-4 | SBSPON |  |  |  |
| SNORD115-40 | SEC24A |  |  |  |
| SNORD115-41 | SGO2 |  |  |  |
| SNORD115-42 | SKIC8 |  |  |  |
| SNORD115-43 | SLC30A5 |  |  |  |
| SNORD115-44 | SLIRP |  |  |  |
| SNORD115-5 | SMARCE1 |  |  |  |
| SNORD115-6 | SNRNP40 |  |  |  |
| SNORD115-7 | SPATS2L |  |  |  |
| SNORD115-8 | SREK1 |  |  |  |
| SNORD115-9 | TBC1D1 |  |  |  |
| SNORD116-10 | TMCC1 |  |  |  |
| SNORD116-11 | TMEM150C |  |  |  |
| SNORD116-13 | TMEM30B |  |  |  |
| SNORD116-14 | TMEM44 |  |  |  |
| SNORD116-15 | TRAPPC9 |  |  |  |
| SNORD116-16 | TRDN |  |  |  |
| SNORD116-17 | TRIL |  |  |  |
| SNORD116-18 | TRIM5 |  |  |  |
| SNORD116-19 | TUBE1 |  |  |  |
| SNORD116-21 | UBAP1 |  |  |  |
| SNORD116-23 | VPS37A |  |  |  |
| SNORD116-24 | VTCN1 |  |  |  |
| SNORD116-4 | VTI1A |  |  |  |
| SNORD116-5 | WDR3 |  |  |  |
| SNORD116-6 | XRCC4 |  |  |  |
| SNORD116-7 | ZCCHC12 |  |  |  |
| SNORD116-8 | ABCF2 |  |  |  |
| SNORD116-9 | ABHD14B |  |  |  |
| LOC124902571 | ADGRL4 |  |  |  |
| SNORD115-24 | ADH1C |  |  |  |
| SNORD115-27 | AFTPH |  |  |  |
| SNORD115-28 | ARHGAP20 |  |  |  |
| SNORD115-45 | ARHGEF37 |  |  |  |
| SNORD116-20 | B3GALT1 |  |  |  |
| SNORD116-22 | BCKDK |  |  |  |
| SNORD116-28 | C15ORF48 |  |  |  |
| SNORD116-30 | CCL28 |  |  |  |
| SNORD115-46 | CD5 |  |  |  |
| SNORD115-47 | CES1E |  |  |  |
| ENSG00000277925 | CHRNA1 |  |  |  |
| ANCR | CLCN5 |  |  |  |
| LOC110806263 | CNMD |  |  |  |
| LOC110806306 | CPNE5 |  |  |  |
| ENSG00000235775 | CTNNBIP1 |  |  |  |
| LOC112272578 | CYP2U1 |  |  |  |
| LOC112272579 | DONSON |  |  |  |
| LOC121847940 | DRAP1 |  |  |  |
| LOC125078046 | EIF5A |  |  |  |
| LOC125078047 | ELMO2 |  |  |  |
| LOC126862076 | ELP4 |  |  |  |
| LOC130068886 | FBXO9 |  |  |  |
| SMAD2 | FNIP1 |  |  |  |
| FSTL1 | GLT1D1 |  |  |  |
| SNORD118 | GNPTAB |  |  |  |
| TIE1 | GRM8 |  |  |  |
| JPH2 | GSTM6 |  |  |  |
| TXNDC15 | H1-4 |  |  |  |
| NDUFA3 | HDAC10 |  |  |  |
| IQSEC3 | HLA-F |  |  |  |
| LRP5L | HSD17B10 |  |  |  |
| GATA4 | IRX3 |  |  |  |
| KAT5 | KCNIP2 |  |  |  |
| NCSTN | KCNJ5 |  |  |  |
| SLC1A3 | KPNA3 |  |  |  |
| GFPT1 | LPAR2 |  |  |  |
| POR | MDGA1 |  |  |  |
| RPS6KA5 | MMAB |  |  |  |
| UBE2N | MOK |  |  |  |
| CNTN2 | MROH1 |  |  |  |
| DDX5 | NAA38 |  |  |  |
| DMD | NDC1 |  |  |  |
| MEN1 | NRG2 |  |  |  |
| APOA2 | NTNG2 |  |  |  |
| IGF2BP2 | ORMDL3 |  |  |  |
| NUP155 | OSBPL2 |  |  |  |
| PDE2A | P4HTM |  |  |  |
| TNKS | PACSIN3 |  |  |  |
| ACADSB | PCDH9 |  |  |  |
| BST1 | PES1 |  |  |  |
| DISC1 | PFKFB2 |  |  |  |
| MPZ | PLCH2 |  |  |  |
| NDUFS2 | PLLP |  |  |  |
| NFASC | POLR2H |  |  |  |
| NIPBL | POPDC3 |  |  |  |
| NUMA1 | PPP2R5E |  |  |  |
| PLEC | RAB3IL1 |  |  |  |
| SLC22A1 | SAMD11 |  |  |  |
| ZFHX3 | SCAMP2 |  |  |  |
| CSNK1G2 | SFMBT1 |  |  |  |
| DSTYK | SH3RF1 |  |  |  |
| JUND | SLC10A6 |  |  |  |
| KDM4C | SLC9A2 |  |  |  |
| SDHC | SMARCA5 |  |  |  |
| DPF2 | SNAPIN |  |  |  |
| HMGN1 | SPECC1 |  |  |  |
| MLX | SPTBN4 |  |  |  |
| SF3B2 | ST3GAL3 |  |  |  |
| SNRPE | SYBU |  |  |  |
| SYVN1 | TLCD2 |  |  |  |
| UBIAD1 | TMEM165 |  |  |  |
| UPF1 | TOR1AIP2 |  |  |  |
| ATP5MC3 | TP53I11 |  |  |  |
| KLC2 | TREM1 |  |  |  |
| MAP3K11 | TSPAN14 |  |  |  |
| MUS81 | TSPAN9 |  |  |  |
| ASH1L | TUG1 |  |  |  |
| B4GALT3 | UBE2L3 |  |  |  |
| COLGALT1 | UBR5 |  |  |  |
| ELMO1 | VIPR2 |  |  |  |
| FCER1G | YOD1 |  |  |  |
| GFI1B | ZFP91 |  |  |  |
| GRK5 | ACOT8 |  |  |  |
| MCM3AP | AGA |  |  |  |
| NRXN2 | ALDH16A1 |  |  |  |
| PDE4C | AP3M2 |  |  |  |
| SIPA1 | ARMCX2 |  |  |  |
| ZFPM2 | ARVCF |  |  |  |
| AFAP1 | ATP6AP1 |  |  |  |
| AKAP6 | ATP6V1C2 |  |  |  |
| ARAP1 | ATXN3 |  |  |  |
| C5AR2 | BIRC6 |  |  |  |
| CCL24 | BRIX1 |  |  |  |
| ORC2 | BTAF1 |  |  |  |
| PARP6 | CCDC88A |  |  |  |
| POLA2 | CD72 |  |  |  |
| RAB1B | CD99 |  |  |  |
| RFXANK | CDC27 |  |  |  |
| RNASEH2C | CHID1 |  |  |  |
| TASP1 | CNDP1 |  |  |  |
| TOP1MT | COASY |  |  |  |
| TOP3B | COL11A1 |  |  |  |
| TRIP10 | COL6A3 |  |  |  |
| ZFP36L2 | COLGALT2 |  |  |  |
| ANXA4 | CPEB3 |  |  |  |
| ATG2A | CRTC2 |  |  |  |
| BTG1 | DDX18 |  |  |  |
| EEF1D | DLX4 |  |  |  |
| ELL | DMTF1 |  |  |  |
| FAU | EGFLAM |  |  |  |
| FMN1 | EIF2AK1 |  |  |  |
| IFI30 | EIF2B5 |  |  |  |
| KCNN1 | EIF3F |  |  |  |
| MYH4 | ELOA |  |  |  |
| MYO9A | ENTPD4 |  |  |  |
| PSMG1 | EOGT |  |  |  |
| RANBP3 | EPX |  |  |  |
| SF1 | ERLIN2 |  |  |  |
| VPS51 | ESR2A |  |  |  |
| XAB2 | F7 |  |  |  |
| B4GAT1 | FAT2 |  |  |  |
| DCTD | FBXL7 |  |  |  |
| DHX34 | FES |  |  |  |
| DRAP1 | FGF4 |  |  |  |
| ERI1 | FGFR1OP2 |  |  |  |
| GAS7 | FPR2 |  |  |  |
| KHSRP | GALNT11 |  |  |  |
| MAU2 | GIMAP4 |  |  |  |
| MRPL11 | GLYCTK |  |  |  |
| MYH13 | HYI |  |  |  |
| PGM2 | IARS2 |  |  |  |
| RIOK1 | IDS |  |  |  |
| RNF144B | IFT57 |  |  |  |
| SART1 | IL4I1 |  |  |  |
| SLC15A4 | INTS3 |  |  |  |
| SOX13 | IP6K1 |  |  |  |
| SUPT3H | KCTD10 |  |  |  |
| THADA | LCT |  |  |  |
| TIGAR | LPIN3 |  |  |  |
| TSNAX | LRRC15 |  |  |  |
| BRMS1 | MAN1B1 |  |  |  |
| COL22A1 | MAPK15 |  |  |  |
| FBXL5 | MBOAT7 |  |  |  |
| GTF2F1 | MRPS30 |  |  |  |
| IRX1 | MRPS33 |  |  |  |
| KMT5C | NACC1 |  |  |  |
| NIPSNAP3B | NAP1L4 |  |  |  |
| NUDT5 | NELFA |  |  |  |
| PLEKHH2 | NINJ2 |  |  |  |
| SSBP4 | NLRC4 |  |  |  |
| TENM3 | NSMCE1 |  |  |  |
| CNIH2 | OAZ1 |  |  |  |
| DDX49 | P2RY12 |  |  |  |
| DHX35 | PARP3 |  |  |  |
| HUNK | PFDN5 |  |  |  |
| PMFBP1 | PIP4P2 |  |  |  |
| RHBDD2 | PLEKHF2 |  |  |  |
| SENP2 | PLEKHG4 |  |  |  |
| SUGP2 | PLPP5 |  |  |  |
| UTP14A | POLR3H |  |  |  |
| YIF1A | PPFIA1 |  |  |  |
| BOD1L1 | PPP1R13B |  |  |  |
| MEF2B | PPP3CB |  |  |  |
| MIPOL1 | PRRC1 |  |  |  |
| RBMS3 | RAD9A |  |  |  |
| SLC48A1 | RORB |  |  |  |
| STARD10 | RPL7L1 |  |  |  |
| ZFPL1 | RSPO1 |  |  |  |
| CPEB2 | RTKN2 |  |  |  |
| DUSP12 | SAP30 |  |  |  |
| FERRY3 | SEC11C |  |  |  |
| GKN2 | SEPHS1 |  |  |  |
| HSBP1 | SFRP5 |  |  |  |
| IFI44L | SIGIRR |  |  |  |
| ISM1 | SLC22A11 |  |  |  |
| KCNK7 | SLIT2 |  |  |  |
| KXD1 | SNHG8 |  |  |  |
| NAALADL1 | SPTY2D1 |  |  |  |
| PELI3 | SRPK1 |  |  |  |
| RUSC1 | SSU72 |  |  |  |
| SNX32 | SUFU |  |  |  |
| TM9SF2 | SUGCT |  |  |  |
| TMEM161A | SUGP2 |  |  |  |
| USP36 | TMEM42 |  |  |  |
| ZNF521 | TMEM63A |  |  |  |
| AP5B1 | TNFAIP8L3 |  |  |  |
| C1QTNF7 | TOR4A |  |  |  |
| CCDC85B | TRH |  |  |  |
| CCDC9 | TRIM38 |  |  |  |
| DEDD | TSC22D2 |  |  |  |
| DGLUCY | UBE2A |  |  |  |
| EHBP1L1 | UBR1 |  |  |  |
| ENDOU | VAC14 |  |  |  |
| IQCN | VANGL1 |  |  |  |
| LRRC25 | WASF1 |  |  |  |
| MTPN | WDR54 |  |  |  |
| TEX29 | WDR76 |  |  |  |
| VIT | WRN |  |  |  |
| WDR70 | YLPM1 |  |  |  |
| XKR6 | ZMYND11 |  |  |  |
| ZNRD2 | ABHD10 |  |  |  |
| ABLIM2 | ACOT11 |  |  |  |
| ARRDC2 | ALPK2 |  |  |  |
| BATF2 | ARHGEF6 |  |  |  |
| EPPK1 | ARID4A |  |  |  |
| LCA5L | ARID4B |  |  |  |
| MIS18A | BGN |  |  |  |
| MRPL17 | BIK |  |  |  |
| MVB12A | CCDC141 |  |  |  |
| NR2C2AP | CCDC34 |  |  |  |
| PARP11 | CD164 |  |  |  |
| THAP10 | CDC73 |  |  |  |
| TOMM40L | CDH6 |  |  |  |
| TSNARE1 | CDK2AP2 |  |  |  |
| ZNHIT2 | CEACAM6 |  |  |  |
| FAM171A1 | CEBPE |  |  |  |
| FAT3 | CENPV |  |  |  |
| LURAP1L | CPNE3 |  |  |  |
| PALS2 | CTBP1 |  |  |  |
| PPP1R3B | DDX3Y |  |  |  |
| PWWP2B | DNAJC5 |  |  |  |
| RPL26L1 | E2F5 |  |  |  |
| SLC25A45 | FASTK |  |  |  |
| STYXL1 | FCHSD2 |  |  |  |
| TSBP1 | FIGN |  |  |  |
| C11orf68 | GABRP |  |  |  |
| EIF1AD | GAS2L3 |  |  |  |
| FAM81A | GOLGB1 |  |  |  |
| FRMD8 | GSTZ1 |  |  |  |
| MILR1 | GTF2A1 |  |  |  |
| SHISA9 | H1-5 |  |  |  |
| ZNF444 | H2AC18 |  |  |  |
| ZNF557 | H2BC12 |  |  |  |
| ZNF583 | IK |  |  |  |
| ZNRF4 | INTS6 |  |  |  |
| CFAP126 | KAT6A |  |  |  |
| PRAG1 | KLHL4 |  |  |  |
| RANBP3L | KMT5B |  |  |  |
| SAC3D1 | KPNA1 |  |  |  |
| PCNX3 | KRCC1 |  |  |  |
| OR2AG2 | KRR1 |  |  |  |
| TMEM81 | L1CAM |  |  |  |
| TSGA10IP | LILRB4 |  |  |  |
| TMEM235 | LRRK1 |  |  |  |
| TTC6 | LZTS2 |  |  |  |
| CIMIP2C | MAP3K2 |  |  |  |
| CEP295NL | MICALL1 |  |  |  |
| CLEC19A | MLST8 |  |  |  |
| IGF2BP2-AS1 | MTURN |  |  |  |
| TRA | MYO19 |  |  |  |
| DAOA-AS1 | N4BP2L2 |  |  |  |
| GVINP1 | NUDT16 |  |  |  |
| TSNAX-DISC1 | OGDHL |  |  |  |
| LINC00114 | OSBPL11 |  |  |  |
| ZNF705EP | OTUB1 |  |  |  |
| AIRN | PELO |  |  |  |
| ST20-AS1 | PHF5A |  |  |  |
| FOXP4-AS1 | PIF1 |  |  |  |
| CCND2-AS1 | POLR3E |  |  |  |
| ERLNC1 | POMP |  |  |  |
| HSP90AA4P | PPM1E |  |  |  |
| MIR612 | PREPL |  |  |  |
| MYHAS | PROSER2 |  |  |  |
| LINC01262 | RASSF6 |  |  |  |
| PPP1R3B-DT | RDH12 |  |  |  |
| XNDC1N | RER1 |  |  |  |
| IGLV8-61 | RHOJ |  |  |  |
| LINC01278 | RNF19A |  |  |  |
| MIR555 | RNFT2 |  |  |  |
| LINC01828 | ROMO1 |  |  |  |
| MBD3L2B | RREB1 |  |  |  |
| ASH1L-AS1 | RYBP |  |  |  |
| IGLV4-60 | SCAND1 |  |  |  |
| LINC01445 | SCAPER |  |  |  |
| LINC01734 | SDF4 |  |  |  |
| LINC02580 | SEMA4C |  |  |  |
| TEX51 | SF3B2 |  |  |  |
| ZNRD2-DT | SLC16A13 |  |  |  |
| ARAP1-AS2 | SLC30A4 |  |  |  |
| C1QTNF7-AS1 | SLC4A4 |  |  |  |
| DCAF8-DT | SNX17 |  |  |  |
| LINC01220 | SPEN |  |  |  |
| LINC01239 | SRSF9 |  |  |  |
| LINC01748 | SUCNR1 |  |  |  |
| MIR4686 | TAOK2 |  |  |  |
| NADK2-AS1 | TBC1D16 |  |  |  |
| PKN2-AS1 | TBC1D17 |  |  |  |
| C8orf90 | TRAF3IP3 |  |  |  |
| DPP3-DT | TSKU |  |  |  |
| LINC01019 | TSPAN31 |  |  |  |
| LINC01700 | TTLL12 |  |  |  |
| LINC01933 | UPF3B |  |  |  |
| LINC02022 | USP40 |  |  |  |
| MIR3126 | VAT1L |  |  |  |
| MIR3189 | VOPP1 |  |  |  |
| NAMPTP1 | ZDHHC21 |  |  |  |
| TRAV36DV7 | ZNRF1 |  |  |  |
| TRAV38-1 | ABHD3 |  |  |  |
| CELF2-DT | ABLIM1 |  |  |  |
| CHD1-DT | ACAP1 |  |  |  |
| CRYBB2P1 | AFG3L2 |  |  |  |
| DNM1P35 | ANGPT4 |  |  |  |
| KLC2-AS1 | ANXA9 |  |  |  |
| LINC01165 | APOL3 |  |  |  |
| LINC01799 | ARSJ |  |  |  |
| LINC01854 | ATP6V1C1 |  |  |  |
| LINC02393 | BMP8B |  |  |  |
| LINC02478 | BTRC |  |  |  |
| LINC03068 | CBARP |  |  |  |
| PGLS-DT | CCDC117 |  |  |  |
| RPAP3-DT | CCDC68 |  |  |  |
| RPL17P39 | CD200R1 |  |  |  |
| SLC25A21-AS1 | CEP170B |  |  |  |
| TALAM1 | CEP290 |  |  |  |
| THA1P | CES1G |  |  |  |
| TRD-AS1 | CHST3 |  |  |  |
| TSBP1-AS1 | CHST8 |  |  |  |
| CAPN1-AS1 | CNNM2 |  |  |  |
| CHP1P2 | COL9A1 |  |  |  |
| DEFT1P | CR1L |  |  |  |
| HMGB1P5 | DCAF13 |  |  |  |
| JDP2-AS1 | DCLK2 |  |  |  |
| LINC01919 | DGKB |  |  |  |
| LINC02337 | DLX3 |  |  |  |
| LINC02411 | EFHC1 |  |  |  |
| LINC02778 | EFNA2 |  |  |  |
| LINC02855 | EIF2B2 |  |  |  |
| LOC100128494 | EMC1 |  |  |  |
| LOC124902694 | FAXDC2 |  |  |  |
| MIR1302-7 | FBXO31 |  |  |  |
| MIR297 | FCHO2 |  |  |  |
| MIR548A1HG | GATA5 |  |  |  |
| MIR6130 | GLDN |  |  |  |
| OSTCP2 | GNPDA2 |  |  |  |
| SF1-DT | GNS |  |  |  |
| ZSCAN5A-AS1 | GOLT1B |  |  |  |
| CEDORA | GPR161 |  |  |  |
| ENSG00000254461 | GPR88 |  |  |  |
| ENSG00000255478 | GRID1 |  |  |  |
| FAM86B3P | HAND1 |  |  |  |
| FAUP4 | HDDC3 |  |  |  |
| KRT8P26 | HOXA9 |  |  |  |
| LINC01017 | HOXD1 |  |  |  |
| LINC02214 | HPS3 |  |  |  |
| LINC02736 | IKZF3 |  |  |  |
| MAP3K4-AS1 | INPP1 |  |  |  |
| RNU6-1048P | ITCH |  |  |  |
| RPL21P94 | JMY |  |  |  |
| RPS15AP30 | KCNJ10 |  |  |  |
| ABHD17AP5 | KDELR2 |  |  |  |
| BAK1P2 | KIFAP3 |  |  |  |
| ENSG00000226334 | LGALS7 |  |  |  |
| ENSG00000233928 | LMO3 |  |  |  |
| ENSG00000234936 | LRRC32 |  |  |  |
| ENSG00000236452 | MAP3K11 |  |  |  |
| ENSG00000253736 | MGAT3 |  |  |  |
| ENSG00000253925 | MIB2 |  |  |  |
| ENSG00000255038 | MRPL11 |  |  |  |
| ENSG00000259925 | MYBPH |  |  |  |
| ENSG00000267523 | NAA50 |  |  |  |
| ENSG00000268030 | NCAM2 |  |  |  |
| ENSG00000268938 | NINL |  |  |  |
| ENSG00000269191 | OSBPL9 |  |  |  |
| ENSG00000269481 | PAX2 |  |  |  |
| ENSG00000269680 | PDCD1 |  |  |  |
| GVINP2 | PDCD10 |  |  |  |
| LINC02063 | PDE1B |  |  |  |
| LOC124901882 | PGAP1 |  |  |  |
| MASCRNA | PKP3 |  |  |  |
| RNU6-1216P | PLA2G15 |  |  |  |
| RNU6-675P | PLCL2 |  |  |  |
| RPS12P20 | PLXNA2 |  |  |  |
| EIF3LP1 | PPIL3 |  |  |  |
| ENEMAL | PRKX |  |  |  |
| ENSG00000223859 | PRX |  |  |  |
| ENSG00000228944 | PTPRR |  |  |  |
| ENSG00000258857 | PTTG1IP |  |  |  |
| ENSG00000259035 | PYCR2 |  |  |  |
| ENSG00000261065 | RAB11FIP5 |  |  |  |
| ENSG00000261460 | RAB5B |  |  |  |
| ENSG00000270117 | RASSF7 |  |  |  |
| ENSG00000272247 | RNF7 |  |  |  |
| ENSG00000278217 | SAP30L |  |  |  |
| EXTL2P1 | SCYL1 |  |  |  |
| LINC02854 | SEMA3D |  |  |  |
| MAPK6P2 | SERINC5 |  |  |  |
| MIR8052 | SIGLEC1 |  |  |  |
| PRDX1P1 | SLC41A3 |  |  |  |
| RNA5SP96 | SLCO5A1 |  |  |  |
| RPL7P45 | SNX1 |  |  |  |
| RPSAP64 | SPART |  |  |  |
| SUMO2P17 | STOML2 |  |  |  |
| ARIH2P1 | SUPV3L1 |  |  |  |
| ENSG00000264067 | SYNGR3 |  |  |  |
| ENSG00000285901 | TBC1D10A |  |  |  |
| ENSG00000286110 | TDP1 |  |  |  |
| ENSG00000286756 | TEX10 |  |  |  |
| ENSG00000287387 | TM2D1 |  |  |  |
| GGTA2P | TMEM109 |  |  |  |
| GUSBP8 | TMEM40 |  |  |  |
| ICE2P2 | TPO |  |  |  |
| LINC02858 | TSPAN6 |  |  |  |
| LOC101929128 | TTLL3 |  |  |  |
| LYPLA1P2 | UBE2K |  |  |  |
| MED6P1 | UBFD1 |  |  |  |
| MTCH2P2 | UFM1 |  |  |  |
| RN7SL97P | USP25 |  |  |  |
| RPL21P61 | UTP3 |  |  |  |
| RPL23AP54 | ZDHHC13 |  |  |  |
| RPL31P44 | ABI2 |  |  |  |
| RPL35AP15 | ACRBP |  |  |  |
| RPL35AP19 | ADAMTSL1 |  |  |  |
| RPL9P6 | AHCTF1 |  |  |  |
| SEPHS1P2 | AMT |  |  |  |
| BOD1P1 | ARPC5 |  |  |  |
| CUX2P1 | ATXN7L1 |  |  |  |
| DNAJC8P3 | CASD1 |  |  |  |
| ENSG00000289259 | CCDC47 |  |  |  |
| HNRNPLP1 | CCL21 |  |  |  |
| LOC105372071 | CHMP4B |  |  |  |
| LOC107985876 | CHRND |  |  |  |
| RAB11AP2 | CNPY2 |  |  |  |
| RN7SL318P | COPZ1 |  |  |  |
| RN7SL553P | CTNNBL1 |  |  |  |
| CARS1P2 | CYRIB |  |  |  |
| DEFA1A3 | D2HGDH |  |  |  |
| ENSG00000213386 | DAZAP2 |  |  |  |
| ENSG00000273388 | DEF8 |  |  |  |
| ENSG00000274902 | DENND2A |  |  |  |
| ENSG00000280118 | DOK5 |  |  |  |
| ENSG00000287523 | DPH2 |  |  |  |
| ENSG00000288096 | DPH6 |  |  |  |
| ENSG00000290061 | EIF3L |  |  |  |
| ENSG00000290063 | ELFN1 |  |  |  |
| ENSG00000291143 | ETFBKMT |  |  |  |
| FCRL4P1 | FAM133B |  |  |  |
| HNRNPA1P58 | FGD5 |  |  |  |
| HSALNG0014529 | FGF14 |  |  |  |
| HSALNG0020226-134 | GALNT18 |  |  |  |
| LOC107983958 | GIMAP8 |  |  |  |
| MTARC2P1 | GLOD4 |  |  |  |
| MTCYBP45 | GREB1L |  |  |  |
| NDUFA5P5 | HAS3 |  |  |  |
| NECAP1P1 | HNRNPUL2 |  |  |  |
| PPIAP65 | HOXB7 |  |  |  |
| RF01684 | ISLR2 |  |  |  |
| RNA5SP228 | JAGN1 |  |  |  |
| RNU4-77P | KANK3 |  |  |  |
| RNU6-1103P | KAT2A |  |  |  |
| TMF1P1 | LEO1 |  |  |  |
| lnc-ABHD4-12 | MAGT1 |  |  |  |
| lnc-CEP295NL-1 | MCEE |  |  |  |
| lnc-CSPG4-1 | MINK1 |  |  |  |
| lnc-DAD1-5 | MIR22HG |  |  |  |
| lnc-IGLL5-1 | MIS12 |  |  |  |
| lnc-LTBP3-2-002 | MRPS31 |  |  |  |
| lnc-LTBP3-6 | NAGK |  |  |  |
| lnc-LTBP3-9 | NOSIP |  |  |  |
| lnc-SENP2-1 | NUDT19 |  |  |  |
| lnc-TOP3B-1 | NUDT6 |  |  |  |
| CM034952-146 | PARD6B |  |  |  |
| CM034952-147 | PFDN4 |  |  |  |
| CM034952-148 | PHF2 |  |  |  |
| CM034954-055 | PIGQ |  |  |  |
| CM034961-155 | PITRM1 |  |  |  |
| CM034962-140 | PLEKHA4 |  |  |  |
| ENSG00000236118 | POFUT2 |  |  |  |
| ENSG00000280091 | POGLUT1 |  |  |  |
| ENSG00000289903 | PPIG |  |  |  |
| ENSG00000291159 | PPP2R5D |  |  |  |
| ENSG00000293552 | PRTFDC1 |  |  |  |
| HSALNG0063305 | PTGDR |  |  |  |
| HSALNG0080720 | PYGM |  |  |  |
| HSALNG0084896 | RAI2 |  |  |  |
| HSALNG0084901 | RFNG |  |  |  |
| HSALNG0088703 | RGS14 |  |  |  |
| HSALNG0099941 | RILPL1 |  |  |  |
| HSALNG0104523-003 | RMDN1 |  |  |  |
| HSALNG0107565 | RNF115 |  |  |  |
| HSALNG0120542 | ROGDI |  |  |  |
| HSALNG0124619 | RPL35A |  |  |  |
| HSALNG0143000 | RUFY1 |  |  |  |
| L13304-005 | RXYLT1 |  |  |  |
| L13715-004 | SDCCAG8 |  |  |  |
| MN298114-240 | SDHAF3 |  |  |  |
| MN309280 | SEM1 |  |  |  |
| NONHSAG010240.2 | SEPTIN6 |  |  |  |
| NONHSAG015714.2 | SGSM2 |  |  |  |
| NONHSAG025130.2 | SIAE |  |  |  |
| RF00017-2916 | SLC35D1 |  |  |  |
| RF00017-6445 | SNIP1 |  |  |  |
| RF00017-7465 | SNX18 |  |  |  |
| RF00017-946 | STK17A |  |  |  |
| lnc-C12orf74-1 | TBC1D2B |  |  |  |
| lnc-C1D-14 | TMEM208 |  |  |  |
| lnc-C1QTNF1-10 | TMEM237 |  |  |  |
| lnc-C5AR1-1 | TRAK1 |  |  |  |
| lnc-C5AR1-3 | TRAPPC3 |  |  |  |
| lnc-CLLU1OS-3 | TTC14 |  |  |  |
| lnc-DDX5-3 | TWSG1 |  |  |  |
| lnc-DDX5-4 | UBE2W |  |  |  |
| lnc-FOXA1-2 | UROC1 |  |  |  |
| lnc-LRRC49-6 | USP39 |  |  |  |
| lnc-MBD3L2B-4 | VPS36 |  |  |  |
| lnc-NIPSNAP3B-4 | WDFY1 |  |  |  |
| lnc-NPY-1 | ZFYVE28 |  |  |  |
| lnc-PGPEP1-7 | ZP3 |  |  |  |
| lnc-PMFBP1-8 | ABCA13 |  |  |  |
| lnc-SS18-4 | ABHD12 |  |  |  |
| lnc-TK1-2 | ADGRB1 |  |  |  |
| lnc-TPGS2-11 | ALDH1A7 |  |  |  |
| lnc-TRA2B-2 | ANKRD34A |  |  |  |
| lnc-VTI1A-8 | ARHGEF11 |  |  |  |
| piR-57133-311 | ARL8A |  |  |  |
| CM034958-416 | ARL8B |  |  |  |
| CM034964-024 | ASTN2 |  |  |  |
| CM034965-020 | ATP6V1F |  |  |  |
| CM034965-054 | ATXN1 |  |  |  |
| CM034971-092 | B3GAT2 |  |  |  |
| ENSG00000258012 | BARX2 |  |  |  |
| HSALNG0032715 | C1QTNF5 |  |  |  |
| HSALNG0041206 | CAMK1G |  |  |  |
| HSALNG0041207 | CAND1 |  |  |  |
| HSALNG0050339 | CCDC126 |  |  |  |
| HSALNG0059030 | CCDC3 |  |  |  |
| HSALNG0069123 | CENPX |  |  |  |
| HSALNG0073417 | CHRD |  |  |  |
| HSALNG0080722 | CLEC11A |  |  |  |
| HSALNG0081795 | CREBZF |  |  |  |
| HSALNG0081800 | DCAF7 |  |  |  |
| HSALNG0085582 | DIRAS2 |  |  |  |
| HSALNG0099439 | DIS3L2 |  |  |  |
| HSALNG0100697 | DLG5 |  |  |  |
| HSALNG0112608 | DNAH11 |  |  |  |
| HSALNG0119074 | ECHS1 |  |  |  |
| HSALNG0123609 | EFR3A |  |  |  |
| HSALNG0126760 | EIF4G3 |  |  |  |
| HSALNG0127685 | ENHO |  |  |  |
| HSALNG0133095 | ERGIC1 |  |  |  |
| HSALNG0147078 | EXOC1 |  |  |  |
| HSALNG0148077 | EXOC7 |  |  |  |
| HSALNG0149719 | EXOSC4 |  |  |  |
| HSALNG0150129 | FAM135A |  |  |  |
| LOC105369905 | FAT3 |  |  |  |
| LOC124901135 | FCGR1A |  |  |  |
| RF00017-2157 | FOXF2 |  |  |  |
| RF00017-2588 | FOXRED2 |  |  |  |
| RF00017-2656 | FUT4 |  |  |  |
| RF00017-4299 | GOSR1 |  |  |  |
| RF00017-7522 | GOT2 |  |  |  |
| hsa-miR-5095-098 | GPR65 |  |  |  |
| lnc-ANKRD10-9 | H3C1 |  |  |  |
| lnc-APOA2-2 | HSBP1 |  |  |  |
| lnc-C12orf74-2 | HTR6 |  |  |  |
| lnc-CALM1-9 | ICE2 |  |  |  |
| lnc-DCTD-14 | KARS1 |  |  |  |
| lnc-DEFA1-2 | KCNJ15 |  |  |  |
| lnc-DUSP1-3 | KIF13A |  |  |  |
| lnc-ENDOU-4-001 | KLC2 |  |  |  |
| lnc-FOXA1-4 | KLF15 |  |  |  |
| lnc-GRK3-4 | KLK10 |  |  |  |
| lnc-LNPK-4 | KMT2E |  |  |  |
| lnc-PIK3C3-6 | LIAS |  |  |  |
| lnc-SLC22A2-3 | LRP2 |  |  |  |
| lnc-SLC25A21-3 | LRRN1 |  |  |  |
| lnc-VPREB1-13 | MAF1 |  |  |  |
| piR-31470-037 | MAML1 |  |  |  |
| piR-37289 | MARCHF1 |  |  |  |
| piR-39858-675 | MED20 |  |  |  |
| piR-43105-290 | MON2 |  |  |  |
| piR-49623 | MPI |  |  |  |
| piR-50437-489 | MSRB1 |  |  |  |
| piR-56229-087 | MTMR4 |  |  |  |
| piR-61945-462 | MTSS2 |  |  |  |
| 5MWI_A-159 | MYOCD |  |  |  |
| CM034965-190 | NCBP2 |  |  |  |
| CM034966-120 | NOMO1 |  |  |  |
| CM034967-041 | PADI4 |  |  |  |
| HSALNG0007917 | PALS2 |  |  |  |
| HSALNG0050335 | PCSK2 |  |  |  |
| HSALNG0050337 | PDZK1 |  |  |  |
| HSALNG0056693 | PPP1R7 |  |  |  |
| HSALNG0068943 | PPP4R4 |  |  |  |
| HSALNG0069122 | PRRG1 |  |  |  |
| HSALNG0076364 | RAB23 |  |  |  |
| HSALNG0077324 | RASL12 |  |  |  |
| HSALNG0079511 | RBM12 |  |  |  |
| HSALNG0098914 | RBM4B |  |  |  |
| HSALNG0099946 | REEP6 |  |  |  |
| HSALNG0104905 | RFLNA |  |  |  |
| HSALNG0126762 | RFTN2 |  |  |  |
| HSALNG0127682 | RHBDD2 |  |  |  |
| HSALNG0132270 | RNASE6 |  |  |  |
| HSALNG0134297 | RNF114 |  |  |  |
| HSALNG0141935 | SBF1 |  |  |  |
| HSALNG0142354 | SCLY |  |  |  |
| HSALNG0144416 | SELENOI |  |  |  |
| HSALNG0146269 | SFI1 |  |  |  |
| LM611511-006 | SLC25A28 |  |  |  |
| LOC105377572 | STXBP4 |  |  |  |
| LOC107984137 | SYDE1 |  |  |  |
| LOC107985004 | TMEM163 |  |  |  |
| LOC124901128 | TOMM22 |  |  |  |
| LOC124902239 | UBE2V1 |  |  |  |
| LOC124902983 | UBLCP1 |  |  |  |
| MN309005 | UCHL5 |  |  |  |
| RF00017-450 | UHRF2 |  |  |  |
| RF00017-7523 | UNC5D |  |  |  |
| RF00994-490 | USP8 |  |  |  |
| lnc-C2orf70-1 | VAV3 |  |  |  |
| lnc-DEFA1-1 | ZCCHC2 |  |  |  |
| lnc-EPPK1-1 | ZHX2 |  |  |  |
| lnc-FAM135B-4 | ALG13 |  |  |  |
| lnc-PSMA8-2 | ALG3 |  |  |  |
| lnc-PSMC3IP-1 | ARAP1 |  |  |  |
| lnc-SCAF4-5 | ARHGAP15 |  |  |  |
| piR-31101-002 | ARHGEF39 |  |  |  |
| piR-36809 | B3GALT4 |  |  |  |
| piR-38351-181 | BAZ2A |  |  |  |
| piR-38580-214 | BLZF1 |  |  |  |
| piR-41245-183 | BRD7 |  |  |  |
| piR-42491-063 | BRWD1 |  |  |  |
| piR-47211-071 | CCDC125 |  |  |  |
| piR-53101 | CCDC28B |  |  |  |
| piR-57461-177 | CDKN2AIP |  |  |  |
| HSALNG0063175 | CENPP |  |  |  |
| MK280073-486 | CENPQ |  |  |  |
| piR-32285-070 | CEP135 |  |  |  |
| piR-48759-273 | CEP164 |  |  |  |
| piR-59347-006 | CEP41 |  |  |  |
| ASAH1 | CERT1 |  |  |  |
| MALT1 | CHD1L |  |  |  |
| PLA2G2A | CLIP3 |  |  |  |
| CALCRL | CLN6 |  |  |  |
| TGFBR3 | CNKSR2 |  |  |  |
| MFGE8 | CYYR1 |  |  |  |
| RAMP2 | DERL2 |  |  |  |
| ZC3H12A | DNAJB13 |  |  |  |
| CEACAM7 | DOCK3 |  |  |  |
| MRGPRX2 | EIF1A |  |  |  |
| CHEK2 | EIF2B3 |  |  |  |
| TBK1 | ELF2 |  |  |  |
| PAFAH1B1 | EMC9 |  |  |  |
| HLA-DPB1 | ENPP4 |  |  |  |
| IFNB1 | EXOSC3 |  |  |  |
| CD84 | FBXL3 |  |  |  |
| PDPN | FBXL4 |  |  |  |
| RSPO1 | FMNL1 |  |  |  |
| DNMT1 | FRAT1 |  |  |  |
| ROCK1 | FYTTD1 |  |  |  |
| SUMO1 | GABRA5 |  |  |  |
| GJB2 | GDF3 |  |  |  |
| PLXNA1 | GIGYF2 |  |  |  |
| FGF14 | GORASP1 |  |  |  |
| TNFRSF12A | GPBP1 |  |  |  |
| DMBT1 | HIRA |  |  |  |
| IFNA1 | HS3ST3A1 |  |  |  |
| S100A1 | KCMF1 |  |  |  |
| HOXA2 | KCNA3 |  |  |  |
| PLA2G4A | KLHL9 |  |  |  |
| EIF2AK2 | KRT34 |  |  |  |
| ENPP1 | LMCD1 |  |  |  |
| RIGI | LONRF2 |  |  |  |
| PPARGC1A | MANEA |  |  |  |
| TACR1 | MDFIC |  |  |  |
| HBEGF | MIR205 |  |  |  |
| SEMA4D | MORF4L2 |  |  |  |
| F2RL3 | MPPE1 |  |  |  |
| MKI67 | MTCH1 |  |  |  |
| NPY1R | N4BP2 |  |  |  |
| SRD5A1 | NCBP1 |  |  |  |
| CD69 | NOXA1 |  |  |  |
| LAP3 | P3H1 |  |  |  |
| CABIN1 | PANK3 |  |  |  |
| CLEC4A | PAOX |  |  |  |
| TAS2R16 | PATZ1 |  |  |  |
| MIR34C | PAX7 |  |  |  |
| SNHG8 | PDXP |  |  |  |
| CSNK1A1 | PEX7 |  |  |  |
| GYS1 | PHKG2 |  |  |  |
| STIM1 | POGZ |  |  |  |
| FBP1 | PPP1R3G |  |  |  |
| FAH | PPP4R1 |  |  |  |
| GYG1 | PTPRD |  |  |  |
| PC | PYGO2 |  |  |  |
| PYGM | RAP2B |  |  |  |
| AGL | RBM26 |  |  |  |
| PYGL | RHBDD1 |  |  |  |
| GBE1 | RNASEH2B |  |  |  |
| GYS2 | SART3 |  |  |  |
| PHKA2 | SCAF1 |  |  |  |
| PHKG2 | SELENOF |  |  |  |
| PHKB | SH2D5 |  |  |  |
| PYGB | SH3GL3 |  |  |  |
| PHKA1 | SLC29A2 |  |  |  |
| NEK3 | SLC35G1 |  |  |  |
| GYG2 | SNRPB2 |  |  |  |
| STIM2 | SOS2 |  |  |  |
| WAC | SYNJ1 |  |  |  |
| G6PC2 | SYNRG |  |  |  |
| ORAI2 | SYTL3 |  |  |  |
| SLC37A1 | TBC1D9B |  |  |  |
| FAM83D | THAP11 |  |  |  |
| SEC16A | TSN |  |  |  |
| SLC37A3 | UBE2J1 |  |  |  |
| FAM83G | UCK1 |  |  |  |
| ORAI3 | VPS8 |  |  |  |
| FAM83A | WBP11 |  |  |  |
| FAM83B | WDR74 |  |  |  |
| FAM83E | XCR1 |  |  |  |
| DEFB103A | ADAMTS14 |  |  |  |
| EDNRB | AGPAT1 |  |  |  |
| ALOX5 | ALS2CL |  |  |  |
| DICER1 | ANKZF1 |  |  |  |
| ADORA2B | ARL5A |  |  |  |
| FGG | ATP8 |  |  |  |
| HSPA1A | B4GALT7 |  |  |  |
| WNT10B | B4GAT1 |  |  |  |
| SAT1 | BFSP1 |  |  |  |
| C1QBP | BHMT2 |  |  |  |
| CIITA | BRCC3 |  |  |  |
| LIF | CASP3A |  |  |  |
| IGFBP4 | CD180 |  |  |  |
| AMD1 | CHMP2A |  |  |  |
| PRKRA | COPS7A |  |  |  |
| LDHC | COQ5 |  |  |  |
| MEOX2 | DTWD1 |  |  |  |
| IL31 | EAF1 |  |  |  |
| MIR107 | EID2 |  |  |  |
| MIR485 | ERG28 |  |  |  |
| ST7-OT3 | FAM13A |  |  |  |
| CCAT1 | FAM13B |  |  |  |
| ABCB1 | FNIP2 |  |  |  |
| MSX1 | GGA2 |  |  |  |
| EPHB4 | GGACT |  |  |  |
| CHEK1 | GIMAP6 |  |  |  |
| DBH | GLTP |  |  |  |
| PLA2G7 | GTF2H4 |  |  |  |
| TOP2A | HYCC1 |  |  |  |
| ALDH1A2 | IDE |  |  |  |
| GLI2 | IMMP2L |  |  |  |
| ARSA | JAKMIP1 |  |  |  |
| PLCG1 | KCNJ1 |  |  |  |
| ARSB | KIF26A |  |  |  |
| FXN | KPNA6 |  |  |  |
| OTC | KRT8 |  |  |  |
| PDK1 | L3HYPDH |  |  |  |
| IKBKE | LEPROTL1 |  |  |  |
| IRF3 | LIN54 |  |  |  |
| PLCB4 | LPP |  |  |  |
| CFD | MBTD1 |  |  |  |
| S1PR1 | METTL21A |  |  |  |
| TEAD1 | MIER1 |  |  |  |
| AREG | MIF4GD |  |  |  |
| FCER2 | MKRN1 |  |  |  |
| GUCY1A1 | MRTFA |  |  |  |
| SDC1 | MS4A6A |  |  |  |
| SVIL | MYPN |  |  |  |
| GUCY1B1 | NEDD8 |  |  |  |
| NPTX1 | NEK1 |  |  |  |
| PRPF6 | NFE2L2A |  |  |  |
| RPL13A | NLRP1 |  |  |  |
| TCF7L1 | NRROS |  |  |  |
| PDLIM3 | PCF11 |  |  |  |
| LHX8 | PEX16 |  |  |  |
| BCAR4 | PGGHG |  |  |  |
| MIR26A2 | PLCB4 |  |  |  |
| RBM5-AS1 | POMGNT2 |  |  |  |
| MT-TP | POU4F2 |  |  |  |
| F2RL2 | PPTC7 |  |  |  |
| MIR7-3HG | PRPF18 |  |  |  |
| KRT14 | RAB37 |  |  |  |
| ALDH3A2 | RAB9A |  |  |  |
| ABHD5 | RNLS |  |  |  |
| ERCC5 | RUNDC3A |  |  |  |
| JAM3 | SAMD5 |  |  |  |
| PPOX | SCRN2 |  |  |  |
| FLG | SEC24B |  |  |  |
| ALOXE3 | SLC2A10 |  |  |  |
| F11R | SLC5A11 |  |  |  |
| BIVM-ERCC5 | SMYD4 |  |  |  |
| CCDST | SNX12 |  |  |  |
| LOC126861834 | SNX13 |  |  |  |
| MMP11 | SNX16 |  |  |  |
| MIR527 | STARD3NL |  |  |  |
| TLR3 | SYMPK |  |  |  |
| IRF8 | TNIP2 |  |  |  |
| TAS2R38 | TRIM69 |  |  |  |
| KRBOX4 | TRNP1 |  |  |  |
| ATP6V0CP1 | UQCC2 |  |  |  |
| CD46 | URB1 |  |  |  |
| TGM2 | VPS13C |  |  |  |
| TYMP | WDR19 |  |  |  |
| FPR2 | WDR33 |  |  |  |
| ATN1 | XPO4 |  |  |  |
| CA6 | ZBED3 |  |  |  |
| PITRM1 | ZC3H4 |  |  |  |
| NLRP6 | ABHD14A |  |  |  |
| PRB3 | ABRACL |  |  |  |
| PCNA | ACOD1 |  |  |  |
| SLC2A2 | ACTR10 |  |  |  |
| F8 | ACTR6 |  |  |  |
| VCL | ADAMTS10 |  |  |  |
| MAP2K6 | AK7 |  |  |  |
| MAP2K7 | AKIP1 |  |  |  |
| CD163 | ANAPC11 |  |  |  |
| EYA1 | ANGPTL1 |  |  |  |
| MGP | ANKRA2 |  |  |  |
| COL9A1 | ANKRD13A |  |  |  |
| FLII | APIP |  |  |  |
| JAM2 | APOO |  |  |  |
| SIL1 | ARHGAP6 |  |  |  |
| CPM | ARHGEF10L |  |  |  |
| ACP2 | ARL2BP |  |  |  |
| MUC5AC | ARMCX1 |  |  |  |
| TAS1R2 | ASXL1 |  |  |  |
| DNAI3 | BBS9 |  |  |  |
| DLEU1 | BLOC1S2 |  |  |  |
| NORAD | BSPRY |  |  |  |
| LINC00511 | C1D |  |  |  |
| MROCKI | CARF |  |  |  |
| JAK2 | CC2D1A |  |  |  |
| VIM | CCDC71L |  |  |  |
| ALK | CDIPT |  |  |  |
| RAC1 | CDK13 |  |  |  |
| TLR8 | CEP128 |  |  |  |
| ANXA5 | CEP131 |  |  |  |
| CSPG4 | CEP63 |  |  |  |
| EDN3 | CFAP20 |  |  |  |
| TLR6 | CHDH |  |  |  |
| ADH4 | CLDN12 |  |  |  |
| BAD | CLOCK |  |  |  |
| BDKRB2 | COX10 |  |  |  |
| HDC | CPSF2 |  |  |  |
| KLK6 | CRBN |  |  |  |
| POLR3A | CTXN1 |  |  |  |
| S100B | DACT3 |  |  |  |
| AMBP | DCAF8 |  |  |  |
| TYROBP | DDX20 |  |  |  |
| CD70 | DENND1A |  |  |  |
| DNAJB2 | DHX38 |  |  |  |
| SECISBP2 | DNASE1 |  |  |  |
| TNFAIP6 | DOC2B |  |  |  |
| EDN2 | DOK2 |  |  |  |
| GUK1 | DRAM2 |  |  |  |
| IL36A | DSC3 |  |  |  |
| NFKBIE | EDC3 |  |  |  |
| TRAPPC3 | EPHB1 |  |  |  |
| CCL14 | ESR1.L |  |  |  |
| TMPRSS13 | EVPL |  |  |  |
| TDRP | FAM110B |  |  |  |
| SPINK6 | FAM169A |  |  |  |
| SPRR2F | FGD2 |  |  |  |
| DEFB4B | FKBP7 |  |  |  |
| MIR193B | FLAD1 |  |  |  |
| LINC01554 | FRMD3 |  |  |  |
| MIR342 | FRS2 |  |  |  |
| MIR455 | GABARAPL1 |  |  |  |
| MIR101-1 | GIT2 |  |  |  |
| CHKB-CPT1B | GLRX5 |  |  |  |
| GACAT2 | GPC1 |  |  |  |
| SNORD44 | GPR153 |  |  |  |
| MIR101-2 | HACD2 |  |  |  |
| USP17L9P | HECA |  |  |  |
| RNA5S9 | HOXA3 |  |  |  |
| PTPN11 | IFNAR1 |  |  |  |
| SLC2A1 | IGSF8 |  |  |  |
| PTPRC | IL9 |  |  |  |
| BMPR1A | INTS7 |  |  |  |
| NT5E | IRGM2 |  |  |  |
| ADK | JOSD1 |  |  |  |
| DSP | KCNQ5 |  |  |  |
| F2 | KSR1 |  |  |  |
| CACNA1S | LHX4 |  |  |  |
| CD55 | LMTK2 |  |  |  |
| SREBF1 | LRRC45 |  |  |  |
| DES | LRRFIP2 |  |  |  |
| FOXO3 | LSAMP |  |  |  |
| HK2 | MAP3K10 |  |  |  |
| SLC2A4 | MARCHF5 |  |  |  |
| CD2 | MCMBP |  |  |  |
| KLKB1 | MDFI |  |  |  |
| HMGA2 | MED1 |  |  |  |
| PTGES3 | MED16 |  |  |  |
| SUFU | MEFV |  |  |  |
| IRF6 | METTL5 |  |  |  |
| MDK | MMGT1 |  |  |  |
| CTSE | MMRN2 |  |  |  |
| PTGES2 | MOSPD3 |  |  |  |
| DSG3 | MRE11A |  |  |  |
| PARD3 | MRPL32 |  |  |  |
| MRC1 | MUSTN1 |  |  |  |
| HSD17B13 | MYDGF |  |  |  |
| LBX1 | NARS1 |  |  |  |
| PKP4 | NDFIP1 |  |  |  |
| ARVCF | NDP |  |  |  |
| TET1 | NECAP1 |  |  |  |
| CLEC4D | NGRN |  |  |  |
| TNP1 | NIPAL4 |  |  |  |
| CCL3L1 | NLRX1 |  |  |  |
| PVT1 | NOVA2 |  |  |  |
| SNHG7 | NSD1 |  |  |  |
| PAPPA-AS1 | NUP88 |  |  |  |
| MIR302D | NXPH4 |  |  |  |
| CLRN1-AS1 | OSGEPL1 |  |  |  |
| PLUT | PAPLN |  |  |  |
| MIR1825 | PEX5 |  |  |  |
| CCL15-CCL14 | PFDN1 |  |  |  |
| GAS6-DT | PGBD5 |  |  |  |
| THORLNC | PIKFYVE |  |  |  |
| MIR3689E | PIN4 |  |  |  |
| MIR4291 | PLXNA4 |  |  |  |
| MIR4477A | PODXL2 |  |  |  |
| MIR6512 | POMT1 |  |  |  |
| TRV-AAC1-4 | PPM1M |  |  |  |
| XS | PPP2R5B |  |  |  |
| KIT | PRSS8 |  |  |  |
| FLT4 | RABEPK |  |  |  |
| COMT | RASL10B |  |  |  |
| CBL | RMND1 |  |  |  |
| CHUK | RNF167 |  |  |  |
| FLT1 | RNF170 |  |  |  |
| MAPT | RPGRIP1 |  |  |  |
| RIPK1 | RPH3AL |  |  |  |
| ANPEP | SCAMP4 |  |  |  |
| FGA | SHC4 |  |  |  |
| IRAK1 | SIM2 |  |  |  |
| CSNK2B | SLC35E4 |  |  |  |
| GLB1 | SLC37A4 |  |  |  |
| MITF | SLC38A7 |  |  |  |
| OPRM1 | SMC6 |  |  |  |
| SDHB | SNX27 |  |  |  |
| TTR | SOX3 |  |  |  |
| ADORA1 | SPAG4 |  |  |  |
| CAD | SPTSSB |  |  |  |
| FOLH1 | STK3 |  |  |  |
| HTR2A | SYT3 |  |  |  |
| MUC1 | SZRD1 |  |  |  |
| SETD2 | TBX6 |  |  |  |
| ADORA2A | TCF20 |  |  |  |
| BECN1 | TDH |  |  |  |
| CACNA2D1 | TMBIM4 |  |  |  |
| CCND3 | TMEM41B |  |  |  |
| CFI | TMEM69 |  |  |  |
| COL18A1 | TMEM87A |  |  |  |
| DDIT3 | TTC12 |  |  |  |
| HSP90B1 | UBIAD1 |  |  |  |
| HTR1A | UBL7 |  |  |  |
| MYOD1 | UFL1 |  |  |  |
| NOG | VCPIP1 |  |  |  |
| NR1H3 | VKORC1L1 |  |  |  |
| PLA2G6 | WDR44 |  |  |  |
| NKX2-5 | YIPF5 |  |  |  |
| NR1D1 | ZBTB11 |  |  |  |
| PPIA | ZFAND2B |  |  |  |
| TJP1 | ZFX |  |  |  |
| VDAC1 | ZKSCAN3 |  |  |  |
| ACTC1 | ZZEF1 |  |  |  |
| ATF1 | AIDA |  |  |  |
| BCL2L11 | AIP |  |  |  |
| CHIT1 | ALMS1 |  |  |  |
| EIF2AK4 | AMOTL1 |  |  |  |
| MST1 | APLN |  |  |  |
| GAB1 | ASPSCR1 |  |  |  |
| LTBP2 | B3GNT3 |  |  |  |
| SLC26A2 | BLCAP |  |  |  |
| LYVE1 | BMPR2 |  |  |  |
| MUC5B | CD244 |  |  |  |
| PTPN3 | CDK11B |  |  |  |
| ROM1 | CDKL1 |  |  |  |
| VAPA | CGAS |  |  |  |
| ADAMTSL1 | CHMP4C |  |  |  |
| AGK | CLASP2 |  |  |  |
| BDKRB1 | CLINT1 |  |  |  |
| FOXO4 | CNIH3 |  |  |  |
| KCNK4 | CPNE4 |  |  |  |
| MAP1LC3B | CWC27 |  |  |  |
| NTS | DDA1 |  |  |  |
| PAWR | DDN |  |  |  |
| PLIN3 | DNAH12 |  |  |  |
| PTMA | DPY19L1 |  |  |  |
| BNIP3 | EDEM2 |  |  |  |
| C4BPA | ESF1 |  |  |  |
| CD83 | ESRRG |  |  |  |
| NR4A3 | EXOC3L4 |  |  |  |
| CD74 | EXOC6 |  |  |  |
| EN1 | FAM13C |  |  |  |
| GRHL2 | FAM83H |  |  |  |
| IGF2BP1 | FAM91A1 |  |  |  |
| LAMP3 | FARP1 |  |  |  |
| MAP1LC3A | FCGR1 |  |  |  |
| MUC2 | GET1 |  |  |  |
| SDC3 | GGA3 |  |  |  |
| CLEC12A | H2AJ |  |  |  |
| MSI1 | H2BC4 |  |  |  |
| PRELP | HOXA4 |  |  |  |
| ATP13A1 | IFT140 |  |  |  |
| C4BPB | IQCG |  |  |  |
| RBM14 | KATNBL1 |  |  |  |
| SDS | KCNF1 |  |  |  |
| NLRX1 | KLRK1 |  |  |  |
| DOK3 | LAMTOR3 |  |  |  |
| AHRR | LRRC34 |  |  |  |
| STK16 | MAEA |  |  |  |
| NBPF14 | MAGEH1 |  |  |  |
| CCL3L3 | MAMLD1 |  |  |  |
| MIR211 | MIR132 |  |  |  |
| MIR135B | MRPS35 |  |  |  |
| MIR27B | MTERF2 |  |  |  |
| MIR215 | MTMR10 |  |  |  |
| MIR296 | MYOC |  |  |  |
| MIR29B2 | NDUFAF3 |  |  |  |
| MIR29C | NOL11 |  |  |  |
| LINC00958 | NOP14 |  |  |  |
| MIR494 | NSUN5 |  |  |  |
| BICDL3P | OPHN1 |  |  |  |
| KCNK15-AS1 | PAFAH1B2 |  |  |  |
| LINC00460 | PAIP2 |  |  |  |
| RNY3 | PEX26 |  |  |  |
| BANCR | PHF10 |  |  |  |
| FAM225A | PIGT |  |  |  |
| INHBA-AS1 | POGLUT2 |  |  |  |
| LINC00857 | RHEBL1 |  |  |  |
| LINC00968 | RNF220 |  |  |  |
| HCG18 | SEPTIN3 |  |  |  |
| IGKV2D-29 | SHPRH |  |  |  |
| MIR4435-2 | SLAIN2 |  |  |  |
| RNU1-4 | SNX25 |  |  |  |
| UPK1A-AS1 | ST6GALNAC6 |  |  |  |
| LINC00278 | TAGAP |  |  |  |
| NR4A1AS | TEX261 |  |  |  |
| TRD-GTC9-1 | TGDS |  |  |  |
| ADGRL3-AS1 | TIE1 |  |  |  |
| LOC148696 | TM2D2 |  |  |  |
| HOXA@ | TRIAP1 |  |  |  |
| HOXB@ | TXLNG |  |  |  |
|  | USP33 |  |  |  |
|  | VPS25 |  |  |  |
|  | WDR35 |  |  |  |
|  | ZFP62 |  |  |  |
|  | ZYG11B |  |  |  |
|  | AASS |  |  |  |
|  | ACVR1B |  |  |  |
|  | ADAMTS20 |  |  |  |
|  | ALG5 |  |  |  |
|  | ANKRD13C |  |  |  |
|  | ANKRD24 |  |  |  |
|  | ANKRD28 |  |  |  |
|  | APLP2 |  |  |  |
|  | ARNT |  |  |  |
|  | ASB13 |  |  |  |
|  | ATG4C |  |  |  |
|  | AVEN |  |  |  |
|  | BCCIP |  |  |  |
|  | BRPF1 |  |  |  |
|  | CAMSAP3 |  |  |  |
|  | CC2D1B |  |  |  |
|  | CCDC112 |  |  |  |
|  | CENPL |  |  |  |
|  | CLEC4A3 |  |  |  |
|  | CNTFR |  |  |  |
|  | COA3 |  |  |  |
|  | COA5 |  |  |  |
|  | COX5B |  |  |  |
|  | CRYBG2 |  |  |  |
|  | CXXC1 |  |  |  |
|  | DAPP1 |  |  |  |
|  | DENR |  |  |  |
|  | DYNC2I2 |  |  |  |
|  | ERI2 |  |  |  |
|  | ESCO1 |  |  |  |
|  | FRMD8 |  |  |  |
|  | GADD45GIP1 |  |  |  |
|  | GHDC |  |  |  |
|  | GINS4 |  |  |  |
|  | GPC3 |  |  |  |
|  | GPR19 |  |  |  |
|  | GRAMD4 |  |  |  |
|  | GSAP |  |  |  |
|  | H2BC8 |  |  |  |
|  | HAUS7 |  |  |  |
|  | HDGFL2 |  |  |  |
|  | HEATR3 |  |  |  |
|  | HOMEZ |  |  |  |
|  | IARS1 |  |  |  |
|  | INO80B |  |  |  |
|  | KCNQ4 |  |  |  |
|  | LAMB2 |  |  |  |
|  | LAMTOR5 |  |  |  |
|  | LIMCH1 |  |  |  |
|  | LRRC40 |  |  |  |
|  | LRSAM1 |  |  |  |
|  | LSM14A |  |  |  |
|  | LSM7 |  |  |  |
|  | LYL1 |  |  |  |
|  | LYZ |  |  |  |
|  | MAML2 |  |  |  |
|  | MFNG |  |  |  |
|  | MICB |  |  |  |
|  | MLIP |  |  |  |
|  | MRPL39 |  |  |  |
|  | MTHFD2L |  |  |  |
|  | MYLK2 |  |  |  |
|  | NEB |  |  |  |
|  | NFX1 |  |  |  |
|  | NFXL1 |  |  |  |
|  | NUDT11 |  |  |  |
|  | NUDT14 |  |  |  |
|  | ORC3 |  |  |  |
|  | PARVB |  |  |  |
|  | PCDHAC2 |  |  |  |
|  | PCNP |  |  |  |
|  | PDAP1 |  |  |  |
|  | PDE4C |  |  |  |
|  | PEAK1 |  |  |  |
|  | PITX3 |  |  |  |
|  | PKHD1L1 |  |  |  |
|  | POFUT1 |  |  |  |
|  | PRMT6 |  |  |  |
|  | PRPSAP2 |  |  |  |
|  | QSER1 |  |  |  |
|  | RAB3GAP2 |  |  |  |
|  | RABL6 |  |  |  |
|  | RAP1GAP |  |  |  |
|  | RAP1GDS1 |  |  |  |
|  | RARRES2 |  |  |  |
|  | RGMB |  |  |  |
|  | RNF130 |  |  |  |
|  | SBF2 |  |  |  |
|  | SFSWAP |  |  |  |
|  | SHISA3 |  |  |  |
|  | SIX3 |  |  |  |
|  | SLC30A6 |  |  |  |
|  | SLC35F1 |  |  |  |
|  | SLC38A9 |  |  |  |
|  | SLCO1B3 |  |  |  |
|  | SPATA7 |  |  |  |
|  | ST8SIA5 |  |  |  |
|  | STAU1 |  |  |  |
|  | TAPBPL |  |  |  |
|  | TBL3 |  |  |  |
|  | TESK1 |  |  |  |
|  | TLNRD1 |  |  |  |
|  | TMEM107 |  |  |  |
|  | TMEM169 |  |  |  |
|  | TRIM29 |  |  |  |
|  | TRMT2A |  |  |  |
|  | TTBK1 |  |  |  |
|  | TXNL4B |  |  |  |
|  | USP20 |  |  |  |
|  | UTP11 |  |  |  |
|  | VEGFB |  |  |  |
|  | VPS52 |  |  |  |
|  | YDJC |  |  |  |
|  | ZFPM2 |  |  |  |
|  | ZFYVE27 |  |  |  |
|  | ZNRF2 |  |  |  |
|  | ADAM32 |  |  |  |
|  | ADAMTS3 |  |  |  |
|  | ADGRA3 |  |  |  |
|  | ADH1A |  |  |  |
|  | AIFM2 |  |  |  |
|  | ALKBH5 |  |  |  |
|  | ANKLE2 |  |  |  |
|  | ARHGAP21 |  |  |  |
|  | ASH1L |  |  |  |
|  | CBL |  |  |  |
|  | CCDC120 |  |  |  |
|  | CCDC127 |  |  |  |
|  | CCDC59 |  |  |  |
|  | CDIP1 |  |  |  |
|  | CEP76 |  |  |  |
|  | COQ8B |  |  |  |
|  | CPTP |  |  |  |
|  | CRHBP |  |  |  |
|  | CYP4F4 |  |  |  |
|  | DCDC2 |  |  |  |
|  | DISP2 |  |  |  |
|  | DOCK2 |  |  |  |
|  | DOK6 |  |  |  |
|  | DSE |  |  |  |
|  | DSTYK |  |  |  |
|  | DUSP11 |  |  |  |
|  | DZIP1 |  |  |  |
|  | EBPL |  |  |  |
|  | ELMOD2 |  |  |  |
|  | ELP1 |  |  |  |
|  | ELP5 |  |  |  |
|  | GPATCH8 |  |  |  |
|  | GPR84 |  |  |  |
|  | HACL1 |  |  |  |
|  | IL21 |  |  |  |
|  | IL21R |  |  |  |
|  | IL27 |  |  |  |
|  | IQCK |  |  |  |
|  | IRGM1 |  |  |  |
|  | ISM1 |  |  |  |
|  | KIF2A |  |  |  |
|  | KLHL22 |  |  |  |
|  | LAMTOR1 |  |  |  |
|  | LARS2 |  |  |  |
|  | LGR6 |  |  |  |
|  | LRAT |  |  |  |
|  | LRRC2 |  |  |  |
|  | LSM10 |  |  |  |
|  | MBLAC2 |  |  |  |
|  | METTL8 |  |  |  |
|  | MNDA |  |  |  |
|  | MRPS10 |  |  |  |
|  | MTA3 |  |  |  |
|  | MYO16 |  |  |  |
|  | ND4L |  |  |  |
|  | NDNF |  |  |  |
|  | NICN1 |  |  |  |
|  | NID2 |  |  |  |
|  | NSMCE2 |  |  |  |
|  | NTMT1 |  |  |  |
|  | NTPCR |  |  |  |
|  | NUP43 |  |  |  |
|  | PARN |  |  |  |
|  | PCDH7 |  |  |  |
|  | PCYT1B |  |  |  |
|  | PITPNM3 |  |  |  |
|  | PPIP5K1 |  |  |  |
|  | PSMG2 |  |  |  |
|  | PTPRT |  |  |  |
|  | RAB21 |  |  |  |
|  | RFX2 |  |  |  |
|  | RGS18 |  |  |  |
|  | RIMS4 |  |  |  |
|  | RMI1 |  |  |  |
|  | RPL36AL |  |  |  |
|  | SEL1L |  |  |  |
|  | SETD3 |  |  |  |
|  | SLC25A5 |  |  |  |
|  | SLC6A17 |  |  |  |
|  | SSC5D |  |  |  |
|  | STMN4 |  |  |  |
|  | STXBP3 |  |  |  |
|  | STXBP5 |  |  |  |
|  | STYX |  |  |  |
|  | TARBP2 |  |  |  |
|  | TMEM254 |  |  |  |
|  | TOMM20 |  |  |  |
|  | TRABD |  |  |  |
|  | TSPOAP1 |  |  |  |
|  | TSR1 |  |  |  |
|  | UGP2 |  |  |  |
|  | USP11 |  |  |  |
|  | VPS53 |  |  |  |
|  | VSIG4 |  |  |  |
|  | ZBTB48 |  |  |  |
|  | ZCCHC17 |  |  |  |
|  | ZFTRAF1 |  |  |  |
|  | ZNF367 |  |  |  |
|  | ABLIM3 |  |  |  |
|  | AMMECR1 |  |  |  |
|  | AMN1 |  |  |  |
|  | AMY1 |  |  |  |
|  | ANAPC5 |  |  |  |
|  | AP3B2 |  |  |  |
|  | APBA1 |  |  |  |
|  | ARL6IP4 |  |  |  |
|  | ATP10B |  |  |  |
|  | BATF2 |  |  |  |
|  | BLOC1S1 |  |  |  |
|  | BRD1 |  |  |  |
|  | CA8 |  |  |  |
|  | CCDC171 |  |  |  |
|  | CDC40 |  |  |  |
|  | CEP350 |  |  |  |
|  | CFAP43 |  |  |  |
|  | COX20 |  |  |  |
|  | CPQ |  |  |  |
|  | CYB5R4 |  |  |  |
|  | DGCR6 |  |  |  |
|  | DHDH |  |  |  |
|  | DOLK |  |  |  |
|  | EIF1AD |  |  |  |
|  | EMC6 |  |  |  |
|  | EYA3 |  |  |  |
|  | FAM120B |  |  |  |
|  | FBXL5 |  |  |  |
|  | FBXO11 |  |  |  |
|  | GLMP |  |  |  |
|  | GOLPH3L |  |  |  |
|  | GRHL3 |  |  |  |
|  | GRPR |  |  |  |
|  | GUCD1 |  |  |  |
|  | H6PD |  |  |  |
|  | IFFO2 |  |  |  |
|  | IL17RC |  |  |  |
|  | IL36B |  |  |  |
|  | INO80 |  |  |  |
|  | INO80C |  |  |  |
|  | ITGA2B |  |  |  |
|  | ITGA8 |  |  |  |
|  | KIZ |  |  |  |
|  | KLHDC2 |  |  |  |
|  | LGI2 |  |  |  |
|  | LMX1B |  |  |  |
|  | LNX2 |  |  |  |
|  | LONP1 |  |  |  |
|  | LONRF1 |  |  |  |
|  | LRRC8E |  |  |  |
|  | LTBR |  |  |  |
|  | LTV1 |  |  |  |
|  | LYRM7 |  |  |  |
|  | MACO1 |  |  |  |
|  | MED10 |  |  |  |
|  | MID1IP1 |  |  |  |
|  | MLX |  |  |  |
|  | MORC3 |  |  |  |
|  | MRPS24 |  |  |  |
|  | MTX2 |  |  |  |
|  | NEUROG1 |  |  |  |
|  | NIF3L1 |  |  |  |
|  | NLK |  |  |  |
|  | NR6A1 |  |  |  |
|  | OSGEP |  |  |  |
|  | OST4 |  |  |  |
|  | PDHB |  |  |  |
|  | PDIA2 |  |  |  |
|  | PDIA5 |  |  |  |
|  | PIGF |  |  |  |
|  | PIGM |  |  |  |
|  | PLAG1 |  |  |  |
|  | PLD6 |  |  |  |
|  | PLEKHG6 |  |  |  |
|  | PLS1 |  |  |  |
|  | POLR1G |  |  |  |
|  | POLR3B |  |  |  |
|  | PPP4R3B |  |  |  |
|  | PRR12 |  |  |  |
|  | PUM1 |  |  |  |
|  | RAP2C |  |  |  |
|  | RCHY1 |  |  |  |
|  | RIMKLA |  |  |  |
|  | RNASEH1 |  |  |  |
|  | RNF181 |  |  |  |
|  | SAMSN1 |  |  |  |
|  | SEH1L |  |  |  |
|  | SESN3 |  |  |  |
|  | SEZ6L2 |  |  |  |
|  | SHISAL1 |  |  |  |
|  | SKIL |  |  |  |
|  | SLMAP |  |  |  |
|  | SNAPC3 |  |  |  |
|  | SOCS6 |  |  |  |
|  | SP140 |  |  |  |
|  | SP6 |  |  |  |
|  | SPAG6 |  |  |  |
|  | SPATA2 |  |  |  |
|  | SPDYA |  |  |  |
|  | SRA1 |  |  |  |
|  | TBC1D8B |  |  |  |
|  | TDRD3 |  |  |  |
|  | TENM3 |  |  |  |
|  | TM4SF20 |  |  |  |
|  | TM7SF3 |  |  |  |
|  | TMBIM6 |  |  |  |
|  | TMCO6 |  |  |  |
|  | TMEM201 |  |  |  |
|  | TNN |  |  |  |
|  | TRIM31 |  |  |  |
|  | URI1 |  |  |  |
|  | USP28 |  |  |  |
|  | WNT10A |  |  |  |
|  | ZDHHC9 |  |  |  |
|  | ZMIZ2 |  |  |  |
|  | ZMYND12 |  |  |  |
|  | ZNF165 |  |  |  |
|  | ANAPC7 |  |  |  |
|  | ANKRD16 |  |  |  |
|  | ANKRD2 |  |  |  |
|  | ANKRD50 |  |  |  |
|  | ARPIN |  |  |  |
|  | BIVM |  |  |  |
|  | CAPN9 |  |  |  |
|  | CCDC85B |  |  |  |
|  | CHST6 |  |  |  |
|  | CLN5 |  |  |  |
|  | CNOT2 |  |  |  |
|  | CNOT8 |  |  |  |
|  | CNPY3 |  |  |  |
|  | COCH |  |  |  |
|  | COL27A1 |  |  |  |
|  | CRTAM |  |  |  |
|  | DGCR2 |  |  |  |
|  | DNAH2 |  |  |  |
|  | DNAJC4 |  |  |  |
|  | ECE2 |  |  |  |
|  | EFNA4 |  |  |  |
|  | FAM110A |  |  |  |
|  | FAM193B |  |  |  |
|  | FBRSL1 |  |  |  |
|  | FBXO41 |  |  |  |
|  | FER1L4 |  |  |  |
|  | FIGNL1 |  |  |  |
|  | FOXN4 |  |  |  |
|  | FTCD |  |  |  |
|  | GALK1 |  |  |  |
|  | GALR1 |  |  |  |
|  | GCC2 |  |  |  |
|  | GET4 |  |  |  |
|  | GNB1L |  |  |  |
|  | GPATCH11 |  |  |  |
|  | HACE1 |  |  |  |
|  | HES4 |  |  |  |
|  | HOXD3 |  |  |  |
|  | IL19 |  |  |  |
|  | INPP5K |  |  |  |
|  | IPO13 |  |  |  |
|  | IRAG2 |  |  |  |
|  | IST1 |  |  |  |
|  | KIAA1671 |  |  |  |
|  | L3MBTL2 |  |  |  |
|  | LMF2 |  |  |  |
|  | LNPK |  |  |  |
|  | LY9 |  |  |  |
|  | MACIR |  |  |  |
|  | MARS2 |  |  |  |
|  | MAST1 |  |  |  |
|  | MEGF11 |  |  |  |
|  | MGRN1 |  |  |  |
|  | MIB1 |  |  |  |
|  | MIR146B |  |  |  |
|  | MTF2 |  |  |  |
|  | MYNN |  |  |  |
|  | MYOZ1 |  |  |  |
|  | NEURL1 |  |  |  |
|  | NUB1 |  |  |  |
|  | OLIG3 |  |  |  |
|  | PIK3CA |  |  |  |
|  | PLXNA1 |  |  |  |
|  | PPCDC |  |  |  |
|  | PPIL4 |  |  |  |
|  | PYCR3 |  |  |  |
|  | PYROXD1 |  |  |  |
|  | RADIL |  |  |  |
|  | RANGRF |  |  |  |
|  | RASA2 |  |  |  |
|  | RASEF |  |  |  |
|  | SCML1 |  |  |  |
|  | SIAH1 |  |  |  |
|  | SLU7 |  |  |  |
|  | SPG21 |  |  |  |
|  | SUN1 |  |  |  |
|  | TARS2 |  |  |  |
|  | TATDN2 |  |  |  |
|  | TBCA |  |  |  |
|  | TBX18 |  |  |  |
|  | TCN2 |  |  |  |
|  | TMEM258 |  |  |  |
|  | TMEM65 |  |  |  |
|  | TMEM67 |  |  |  |
|  | TRPC4AP |  |  |  |
|  | TSPAN11 |  |  |  |
|  | UBXN6 |  |  |  |
|  | UNC80 |  |  |  |
|  | UPRT |  |  |  |
|  | UTRN |  |  |  |
|  | VSTM2L |  |  |  |
|  | WAC |  |  |  |
|  | WHRN |  |  |  |
|  | ZBTB8OS |  |  |  |
|  | ZFAS1 |  |  |  |
|  | ZNF185 |  |  |  |
|  | ZNF277 |  |  |  |
|  | ZNF385B |  |  |  |
|  | ZNF93 |  |  |  |
|  | ACOXL |  |  |  |
|  | ADAMTS17 |  |  |  |
|  | ADAT2 |  |  |  |
|  | AMZ1 |  |  |  |
|  | ANGEL2 |  |  |  |
|  | APEX1 |  |  |  |
|  | ARL11 |  |  |  |
|  | ATP5MC2 |  |  |  |
|  | BABAM2 |  |  |  |
|  | BCL7B |  |  |  |
|  | BSDC1 |  |  |  |
|  | CALY |  |  |  |
|  | CCN5 |  |  |  |
|  | CCP110 |  |  |  |
|  | CD79A |  |  |  |
|  | CDKN2AIPNL |  |  |  |
|  | CENPO |  |  |  |
|  | CETP |  |  |  |
|  | CHM |  |  |  |
|  | CHMP1A |  |  |  |
|  | CILP2 |  |  |  |
|  | CLEC4N |  |  |  |
|  | CNNM1 |  |  |  |
|  | COMMD9 |  |  |  |
|  | CSNK1G2 |  |  |  |
|  | CWC25 |  |  |  |
|  | CWF19L2 |  |  |  |
|  | CYTB |  |  |  |
|  | CYTH2 |  |  |  |
|  | DCLK3 |  |  |  |
|  | DENND2C |  |  |  |
|  | DISP1 |  |  |  |
|  | DMAP1 |  |  |  |
|  | DMRT2 |  |  |  |
|  | FAM184B |  |  |  |
|  | GDF5 |  |  |  |
|  | GNGT1 |  |  |  |
|  | HGSNAT |  |  |  |
|  | HOXB4 |  |  |  |
|  | IAH1 |  |  |  |
|  | IDO2 |  |  |  |
|  | IFIT5 |  |  |  |
|  | KCNN1 |  |  |  |
|  | KLHL25 |  |  |  |
|  | KMT2B |  |  |  |
|  | LRWD1 |  |  |  |
|  | MELTF |  |  |  |
|  | MFSD3 |  |  |  |
|  | MINDY1 |  |  |  |
|  | MIR19A |  |  |  |
|  | MOB3C |  |  |  |
|  | NAT8F5 |  |  |  |
|  | NCDN |  |  |  |
|  | NR1D1 |  |  |  |
|  | NXPH1 |  |  |  |
|  | OLFM2 |  |  |  |
|  | OXNAD1 |  |  |  |
|  | PCSK6 |  |  |  |
|  | PHPT1 |  |  |  |
|  | POLR2K |  |  |  |
|  | PWP2 |  |  |  |
|  | RFESD |  |  |  |
|  | RSRC1 |  |  |  |
|  | SEL1L3 |  |  |  |
|  | SGK2 |  |  |  |
|  | SPX |  |  |  |
|  | ST14 |  |  |  |
|  | STARD7 |  |  |  |
|  | TERF2IP |  |  |  |
|  | TFCP2 |  |  |  |
|  | THAP1 |  |  |  |
|  | THNSL1 |  |  |  |
|  | TIMMDC1 |  |  |  |
|  | TRAPPC2L |  |  |  |
|  | TSNAX |  |  |  |
|  | TXNDC12 |  |  |  |
|  | USP49 |  |  |  |
|  | VIT |  |  |  |
|  | VPS33B |  |  |  |
|  | XPNPEP3 |  |  |  |
|  | ZC3H7B |  |  |  |
|  | ZDHHC3 |  |  |  |
|  | ZGPAT |  |  |  |
|  | ZSCAN31 |  |  |  |
|  | AKIRIN2 |  |  |  |
|  | APBA3 |  |  |  |
|  | ARB2A |  |  |  |
|  | ARHGAP27 |  |  |  |
|  | ATAD2B |  |  |  |
|  | ATCAY |  |  |  |
|  | ATG9B |  |  |  |
|  | ATRIP |  |  |  |
|  | BBS4 |  |  |  |
|  | CADM3 |  |  |  |
|  | CCDC167 |  |  |  |
|  | CCDC65 |  |  |  |
|  | CCNK |  |  |  |
|  | CD177 |  |  |  |
|  | CD2 |  |  |  |
|  | CHCHD7 |  |  |  |
|  | CHN2 |  |  |  |
|  | CNPY4 |  |  |  |
|  | COIL |  |  |  |
|  | CREB3L4 |  |  |  |
|  | CTSV |  |  |  |
|  | CYC1 |  |  |  |
|  | CYP2D9 |  |  |  |
|  | DDC |  |  |  |
|  | DDO |  |  |  |
|  | DNAAF5 |  |  |  |
|  | DOCK8 |  |  |  |
|  | DPP8 |  |  |  |
|  | DPY19L3 |  |  |  |
|  | EMB |  |  |  |
|  | EMC3 |  |  |  |
|  | EPHA10 |  |  |  |
|  | FAM234B |  |  |  |
|  | GPATCH2 |  |  |  |
|  | GTF2IRD2 |  |  |  |
|  | HCFC2 |  |  |  |
|  | HSPBAP1 |  |  |  |
|  | HVCN1 |  |  |  |
|  | IFFO1 |  |  |  |
|  | IFT25 |  |  |  |
|  | IQSEC2 |  |  |  |
|  | JAM2 |  |  |  |
|  | KLHL23 |  |  |  |
|  | LEMD2 |  |  |  |
|  | LMBR1 |  |  |  |
|  | LRRC42 |  |  |  |
|  | LRRC7 |  |  |  |
|  | MBD6 |  |  |  |
|  | MCRS1 |  |  |  |
|  | MED23 |  |  |  |
|  | MED7 |  |  |  |
|  | MIR125A |  |  |  |
|  | MIR145 |  |  |  |
|  | MIR192 |  |  |  |
|  | MPV17L2 |  |  |  |
|  | MRPL36 |  |  |  |
|  | MS4A6D |  |  |  |
|  | NAA30 |  |  |  |
|  | NAA40 |  |  |  |
|  | NADSYN1 |  |  |  |
|  | NDRG3 |  |  |  |
|  | NGDN |  |  |  |
|  | NIPSNAP2 |  |  |  |
|  | NUDCD1 |  |  |  |
|  | NXT1 |  |  |  |
|  | NYNRIN |  |  |  |
|  | OASL1 |  |  |  |
|  | PAM16 |  |  |  |
|  | PARD6A |  |  |  |
|  | PGLYRP2 |  |  |  |
|  | PI3 |  |  |  |
|  | PLCH1 |  |  |  |
|  | PLIN4 |  |  |  |
|  | PLXNC1 |  |  |  |
|  | POM121 |  |  |  |
|  | PPT2 |  |  |  |
|  | RAB11FIP2 |  |  |  |
|  | RAB26 |  |  |  |
|  | RABGGTA |  |  |  |
|  | RBM18 |  |  |  |
|  | RSBN1 |  |  |  |
|  | RWDD3 |  |  |  |
|  | SCAF8 |  |  |  |
|  | SENP2 |  |  |  |
|  | SLC1A7 |  |  |  |
|  | SLC35E3 |  |  |  |
|  | SLC49A4 |  |  |  |
|  | SNTB2 |  |  |  |
|  | SPPL2A |  |  |  |
|  | STIM2 |  |  |  |
|  | STXBP5L |  |  |  |
|  | TACSTD2 |  |  |  |
|  | TAF12 |  |  |  |
|  | TASOR |  |  |  |
|  | TBCK |  |  |  |
|  | TLCD3A |  |  |  |
|  | TMEM131 |  |  |  |
|  | TMEM132C |  |  |  |
|  | TMEM147 |  |  |  |
|  | TMEM170B |  |  |  |
|  | TMEM30A |  |  |  |
|  | TMSB15A |  |  |  |
|  | TOP3A |  |  |  |
|  | TRIM37 |  |  |  |
|  | TRPM1 |  |  |  |
|  | TSGA10 |  |  |  |
|  | TSHZ3 |  |  |  |
|  | TSPYL1 |  |  |  |
|  | TTC33 |  |  |  |
|  | TTC7A |  |  |  |
|  | TXNL4A |  |  |  |
|  | UBALD2 |  |  |  |
|  | URB2 |  |  |  |
|  | VANGL2 |  |  |  |
|  | VWA7 |  |  |  |
|  | WDFY4 |  |  |  |
|  | WDR47 |  |  |  |
|  | ZBTB5 |  |  |  |
|  | ZC3H10 |  |  |  |
|  | ZFAND6 |  |  |  |
|  | ZNHIT3 |  |  |  |
|  | AAGAB |  |  |  |
|  | ADAL |  |  |  |
|  | AGXT |  |  |  |
|  | AMIGO3 |  |  |  |
|  | ANKRD13B |  |  |  |
|  | ARMH4 |  |  |  |
|  | ASB9 |  |  |  |
|  | ATP5PO |  |  |  |
|  | ATP6AP1L |  |  |  |
|  | ATXN7 |  |  |  |
|  | BAALC |  |  |  |
|  | BBS5 |  |  |  |
|  | BBX |  |  |  |
|  | CASS4 |  |  |  |
|  | CCDC186 |  |  |  |
|  | CCM2L |  |  |  |
|  | CEP95 |  |  |  |
|  | CGGBP1 |  |  |  |
|  | CNN2 |  |  |  |
|  | COA7 |  |  |  |
|  | CSN1S1 |  |  |  |
|  | CWC22 |  |  |  |
|  | DAAM2 |  |  |  |
|  | DAZAP1 |  |  |  |
|  | DEUP1 |  |  |  |
|  | DLST |  |  |  |
|  | DNAAF3 |  |  |  |
|  | ECSIT |  |  |  |
|  | ENOPH1 |  |  |  |
|  | FAN1 |  |  |  |
|  | FEM1B |  |  |  |
|  | FRAS1 |  |  |  |
|  | FZD6 |  |  |  |
|  | GALR2 |  |  |  |
|  | GATAD1 |  |  |  |
|  | GDF2 |  |  |  |
|  | GGT7 |  |  |  |
|  | HAUS2 |  |  |  |
|  | IMP3 |  |  |  |
|  | INO80D |  |  |  |
|  | KLHL14 |  |  |  |
|  | KPNA5 |  |  |  |
|  | LRRC56 |  |  |  |
|  | LYRM9 |  |  |  |
|  | MATN1 |  |  |  |
|  | MED18 |  |  |  |
|  | MED29 |  |  |  |
|  | MED4 |  |  |  |
|  | MID1 |  |  |  |
|  | MOB3A |  |  |  |
|  | MRPL27 |  |  |  |
|  | MTPAP |  |  |  |
|  | MYF5 |  |  |  |
|  | N6AMT1 |  |  |  |
|  | NAPB |  |  |  |
|  | ND1 |  |  |  |
|  | NEDD1 |  |  |  |
|  | OPN3 |  |  |  |
|  | OTX1 |  |  |  |
|  | P2RY10 |  |  |  |
|  | PCSK7 |  |  |  |
|  | PHF24 |  |  |  |
|  | PLPPR1 |  |  |  |
|  | PPFIA4 |  |  |  |
|  | RASGEF1C |  |  |  |
|  | REG3B |  |  |  |
|  | RHCG |  |  |  |
|  | RIPOR3 |  |  |  |
|  | RPRD1B |  |  |  |
|  | RUFY2 |  |  |  |
|  | SERPINA9 |  |  |  |
|  | SLC15A1 |  |  |  |
|  | SLC6A19 |  |  |  |
|  | SLFN2 |  |  |  |
|  | SPTBN2 |  |  |  |
|  | SSTR4 |  |  |  |
|  | TBC1D10C |  |  |  |
|  | TBCC |  |  |  |
|  | TCF23 |  |  |  |
|  | TGM3 |  |  |  |
|  | THOC7 |  |  |  |
|  | THUMPD3 |  |  |  |
|  | TM9SF4 |  |  |  |
|  | TMC8 |  |  |  |
|  | UBE3B |  |  |  |
|  | VPS28 |  |  |  |
|  | VPS54 |  |  |  |
|  | ZDHHC17 |  |  |  |
|  | ZNF608 |  |  |  |
|  | ACSBG2 |  |  |  |
|  | AFG2A |  |  |  |
|  | AIRE |  |  |  |
|  | ARHGAP39 |  |  |  |
|  | BMP10 |  |  |  |
|  | CILK1 |  |  |  |
|  | CLDN18 |  |  |  |
|  | CLEC4A |  |  |  |
|  | CNIH4 |  |  |  |
|  | COLEC11 |  |  |  |
|  | CPN2 |  |  |  |
|  | CSRNP2 |  |  |  |
|  | CWF19L1 |  |  |  |
|  | ELAC1 |  |  |  |
|  | FAM20B |  |  |  |
|  | FAM32A |  |  |  |
|  | FANK1 |  |  |  |
|  | FCRL2 |  |  |  |
|  | FRMPD4 |  |  |  |
|  | GDAP1 |  |  |  |
|  | GDAP1L1 |  |  |  |
|  | GDAP2 |  |  |  |
|  | GPM6A |  |  |  |
|  | GPN1 |  |  |  |
|  | GPR135 |  |  |  |
|  | GPR87 |  |  |  |
|  | HMCN1 |  |  |  |
|  | HS3ST5 |  |  |  |
|  | IFI203 |  |  |  |
|  | IFITM6 |  |  |  |
|  | INVS |  |  |  |
|  | KCTD3 |  |  |  |
|  | KLHDC9 |  |  |  |
|  | LAG3 |  |  |  |
|  | LRRC25 |  |  |  |
|  | LTBP4 |  |  |  |
|  | MAP4K3 |  |  |  |
|  | MAPKBP1 |  |  |  |
|  | MEP1A |  |  |  |
|  | MICU3 |  |  |  |
|  | MPP3 |  |  |  |
|  | MYG1 |  |  |  |
|  | NANP |  |  |  |
|  | NAXD |  |  |  |
|  | NFAM1 |  |  |  |
|  | OPLAH |  |  |  |
|  | PCNX3 |  |  |  |
|  | PDCD7 |  |  |  |
|  | PDCL |  |  |  |
|  | PELI3 |  |  |  |
|  | PIGK |  |  |  |
|  | PIGS |  |  |  |
|  | PLPPR2 |  |  |  |
|  | POLR3GL |  |  |  |
|  | RABGAP1 |  |  |  |
|  | RNF38 |  |  |  |
|  | RPL39L |  |  |  |
|  | RTL8A |  |  |  |
|  | SDF2 |  |  |  |
|  | SERPINB6A |  |  |  |
|  | SH2D2A |  |  |  |
|  | SH3GLB2 |  |  |  |
|  | SSH2 |  |  |  |
|  | SSH3 |  |  |  |
|  | TBC1D10B |  |  |  |
|  | TERF2 |  |  |  |
|  | TMEM104 |  |  |  |
|  | TMEM60 |  |  |  |
|  | TNFAIP8L2 |  |  |  |
|  | TOMM40 |  |  |  |
|  | TUBGCP5 |  |  |  |
|  | UNC50 |  |  |  |
|  | VRK3 |  |  |  |
|  | VTA1 |  |  |  |
|  | WASHC3 |  |  |  |
|  | ZNF618 |  |  |  |
|  | ZNF638 |  |  |  |
|  | ADSS1 |  |  |  |
|  | AKR1B7 |  |  |  |
|  | ANGPTL2 |  |  |  |
|  | APOOL |  |  |  |
|  | ARMC8 |  |  |  |
|  | ASMTL |  |  |  |
|  | AZI2 |  |  |  |
|  | BEAN1 |  |  |  |
|  | BICRAL |  |  |  |
|  | C1ORF116 |  |  |  |
|  | CA13 |  |  |  |
|  | CCDC107 |  |  |  |
|  | CCDC82 |  |  |  |
|  | CCDC90B |  |  |  |
|  | CFAP53 |  |  |  |
|  | CHURC1 |  |  |  |
|  | CIAO2A |  |  |  |
|  | CYB5D2 |  |  |  |
|  | CYP2J2 |  |  |  |
|  | DCTN3 |  |  |  |
|  | DDX19B |  |  |  |
|  | DUSP15 |  |  |  |
|  | DZIP1L |  |  |  |
|  | FAM193A |  |  |  |
|  | FUT11 |  |  |  |
|  | GP1BA |  |  |  |
|  | GPR108 |  |  |  |
|  | GRIP2 |  |  |  |
|  | GRIPAP1 |  |  |  |
|  | HHAT |  |  |  |
|  | IGFLR1 |  |  |  |
|  | LMBR1L |  |  |  |
|  | LRIF1 |  |  |  |
|  | LTB4R |  |  |  |
|  | MAP3K9 |  |  |  |
|  | MCAT |  |  |  |
|  | MFSD1 |  |  |  |
|  | MGME1 |  |  |  |
|  | MINDY2 |  |  |  |
|  | MIR99A |  |  |  |
|  | MSTO1 |  |  |  |
|  | NELL2 |  |  |  |
|  | NRDC |  |  |  |
|  | PAGR1 |  |  |  |
|  | PDE7A |  |  |  |
|  | PGM2L1 |  |  |  |
|  | PHB2 |  |  |  |
|  | PLEKHG2 |  |  |  |
|  | POF1B |  |  |  |
|  | PPP1R3A |  |  |  |
|  | PTK6 |  |  |  |
|  | RAB42 |  |  |  |
|  | RADX |  |  |  |
|  | RCC1L |  |  |  |
|  | RDH13 |  |  |  |
|  | RGS7BP |  |  |  |
|  | RNF122 |  |  |  |
|  | RNF135 |  |  |  |
|  | RNF185 |  |  |  |
|  | RP9 |  |  |  |
|  | RPP38 |  |  |  |
|  | SLC26A7 |  |  |  |
|  | SLC38A3 |  |  |  |
|  | SMG5 |  |  |  |
|  | SNHG6 |  |  |  |
|  | SPAG16 |  |  |  |
|  | SPIN4 |  |  |  |
|  | SPPL3 |  |  |  |
|  | TAF6L |  |  |  |
|  | TCHP |  |  |  |
|  | THAP3 |  |  |  |
|  | TINAGL1 |  |  |  |
|  | TM4SF4 |  |  |  |
|  | TMEM199 |  |  |  |
|  | TMEM230 |  |  |  |
|  | TMEM245 |  |  |  |
|  | TRIM16L |  |  |  |
|  | UPF3A |  |  |  |
|  | USP31 |  |  |  |
|  | USP6NL |  |  |  |
|  | WFS1 |  |  |  |
|  | YIF1B |  |  |  |
|  | ZBTB26 |  |  |  |
|  | ZBTB42 |  |  |  |
|  | ZIC4 |  |  |  |
|  | ZNF827 |  |  |  |
|  | ZUP1 |  |  |  |
|  | ACAP2 |  |  |  |
|  | ADAM9 |  |  |  |
|  | AICDA |  |  |  |
|  | ANKS1A |  |  |  |
|  | ATPAF1 |  |  |  |
|  | BAIAP2L2 |  |  |  |
|  | BRD9 |  |  |  |
|  | BTBD10 |  |  |  |
|  | CA5B |  |  |  |
|  | CAPN7 |  |  |  |
|  | CFAP70 |  |  |  |
|  | CINP |  |  |  |
|  | CLEC1B |  |  |  |
|  | CNOT7 |  |  |  |
|  | COMMD6 |  |  |  |
|  | CRB2 |  |  |  |
|  | CRYBB3 |  |  |  |
|  | DDX50 |  |  |  |
|  | DOLPP1 |  |  |  |
|  | E2F6 |  |  |  |
|  | GBP7 |  |  |  |
|  | GCNT4 |  |  |  |
|  | GJA4 |  |  |  |
|  | GPR37L1 |  |  |  |
|  | GSPT2 |  |  |  |
|  | GTF3C6 |  |  |  |
|  | H3C10 |  |  |  |
|  | HEG1 |  |  |  |
|  | HESX1 |  |  |  |
|  | HIC2 |  |  |  |
|  | HLA-DQA1 |  |  |  |
|  | HMCN2 |  |  |  |
|  | HOXC9 |  |  |  |
|  | HYPK |  |  |  |
|  | INTS9 |  |  |  |
|  | IPO11 |  |  |  |
|  | IQCD |  |  |  |
|  | KCNK13 |  |  |  |
|  | KIAA1549L |  |  |  |
|  | KLHL32 |  |  |  |
|  | LCMT2 |  |  |  |
|  | MAB21L1 |  |  |  |
|  | MAGI1 |  |  |  |
|  | MBL1 |  |  |  |
|  | MEAF6 |  |  |  |
|  | METTL16 |  |  |  |
|  | MIER3 |  |  |  |
|  | MIGA1 |  |  |  |
|  | MIR27A |  |  |  |
|  | MIR29A |  |  |  |
|  | MIR93 |  |  |  |
|  | MORN2 |  |  |  |
|  | MS4A2 |  |  |  |
|  | MSANTD4 |  |  |  |
|  | MTMR9 |  |  |  |
|  | NMBR |  |  |  |
|  | OCIAD1 |  |  |  |
|  | P2RY13 |  |  |  |
|  | PALB2 |  |  |  |
|  | PDZD7 |  |  |  |
|  | PHF12 |  |  |  |
|  | PHOSPHO2 |  |  |  |
|  | POLDIP3 |  |  |  |
|  | PPFIBP2 |  |  |  |
|  | PTGR3 |  |  |  |
|  | PTP4A2 |  |  |  |
|  | PTPRCAP |  |  |  |
|  | RAB39B |  |  |  |
|  | RARS2 |  |  |  |
|  | RBM45 |  |  |  |
|  | RBMS3 |  |  |  |
|  | REX1BD |  |  |  |
|  | RIOK1 |  |  |  |
|  | RIPOR2 |  |  |  |
|  | RPP14 |  |  |  |
|  | RPRD1A |  |  |  |
|  | SCAF4 |  |  |  |
|  | SEC23IP |  |  |  |
|  | SLC25A44 |  |  |  |
|  | SLC35C1 |  |  |  |
|  | SLC44A5 |  |  |  |
|  | SNX30 |  |  |  |
|  | SPCS1 |  |  |  |
|  | SPSB3 |  |  |  |
|  | STK35 |  |  |  |
|  | TAF5 |  |  |  |
|  | TBX5 |  |  |  |
|  | TGM5 |  |  |  |
|  | TIMM8A1 |  |  |  |
|  | TINAG |  |  |  |
|  | TMEM115 |  |  |  |
|  | TMEM167A |  |  |  |
|  | TMEM192 |  |  |  |
|  | TMEM50A |  |  |  |
|  | TMX3 |  |  |  |
|  | TPCN2 |  |  |  |
|  | UNC13A |  |  |  |
|  | UVSSA |  |  |  |
|  | VASN |  |  |  |
|  | VPS39 |  |  |  |
|  | WRAP53 |  |  |  |
|  | XKRX |  |  |  |
|  | YME1L1 |  |  |  |
|  | ZBTB24 |  |  |  |
|  | ZFP90 |  |  |  |
|  | ACP2 |  |  |  |
|  | ALKBH1 |  |  |  |
|  | ATP13A4 |  |  |  |
|  | ATP6V0E1 |  |  |  |
|  | BRMS1L |  |  |  |
|  | CCSAP |  |  |  |
|  | CDC42BPA |  |  |  |
|  | CEP112 |  |  |  |
|  | CFHR1 |  |  |  |
|  | CHRNA5 |  |  |  |
|  | COG3 |  |  |  |
|  | CRACD |  |  |  |
|  | CRACDL |  |  |  |
|  | DAB1 |  |  |  |
|  | DHDDS |  |  |  |
|  | ERICH5 |  |  |  |
|  | EVC |  |  |  |
|  | EXOC3L2 |  |  |  |
|  | FAM221A |  |  |  |
|  | FTSJ1 |  |  |  |
|  | GTF3A |  |  |  |
|  | H2AC15 |  |  |  |
|  | HAMP2 |  |  |  |
|  | HEPACAM2 |  |  |  |
|  | HOXD11 |  |  |  |
|  | HSCB |  |  |  |
|  | KIF3B |  |  |  |
|  | LPAR5 |  |  |  |
|  | LRRC4C |  |  |  |
|  | LZIC |  |  |  |
|  | MAML3 |  |  |  |
|  | MAP7D2 |  |  |  |
|  | MAP7D3 |  |  |  |
|  | MDH1B |  |  |  |
|  | METTL17 |  |  |  |
|  | METTL23 |  |  |  |
|  | MFAP5 |  |  |  |
|  | MIR15B |  |  |  |
|  | MIR182 |  |  |  |
|  | MTMR12 |  |  |  |
|  | MYZAP |  |  |  |
|  | NALF2 |  |  |  |
|  | NDUFA2 |  |  |  |
|  | NKX2-2 |  |  |  |
|  | PDCD1LG2 |  |  |  |
|  | PDE6C |  |  |  |
|  | PFKFB1 |  |  |  |
|  | PHACTR4 |  |  |  |
|  | PSMG4 |  |  |  |
|  | RANBP3L |  |  |  |
|  | RHPN1 |  |  |  |
|  | RPAIN |  |  |  |
|  | SERGEF |  |  |  |
|  | SLC25A3 |  |  |  |
|  | SMARCD1 |  |  |  |
|  | SNX15 |  |  |  |
|  | SNX20 |  |  |  |
|  | STK33 |  |  |  |
|  | TAF1B |  |  |  |
|  | TBC1D2 |  |  |  |
|  | TBR1 |  |  |  |
|  | TEX11 |  |  |  |
|  | TOP1MT |  |  |  |
|  | TRIM72 |  |  |  |
|  | TRMT11 |  |  |  |
|  | UBQLN4 |  |  |  |
|  | YIPF1 |  |  |  |
|  | ZBTB32 |  |  |  |
|  | ZFP42 |  |  |  |
|  | ZHX1 |  |  |  |
|  | ZNF207 |  |  |  |
|  | ABHD17B |  |  |  |
|  | ADGRB3 |  |  |  |
|  | AOC2 |  |  |  |
|  | BIRC7 |  |  |  |
|  | C3ORF52 |  |  |  |
|  | C4BP |  |  |  |
|  | C8A |  |  |  |
|  | CHIC1 |  |  |  |
|  | CRIPT |  |  |  |
|  | CYTOR |  |  |  |
|  | DAPL1 |  |  |  |
|  | DDX28 |  |  |  |
|  | DRG2 |  |  |  |
|  | E4F1 |  |  |  |
|  | EPS15L1 |  |  |  |
|  | FAM131B |  |  |  |
|  | FBXL18 |  |  |  |
|  | FBXW2 |  |  |  |
|  | FFAR2 |  |  |  |
|  | FSTL5 |  |  |  |
|  | FUZ |  |  |  |
|  | GIMAP5 |  |  |  |
|  | GOLGA7 |  |  |  |
|  | H2BC18 |  |  |  |
|  | H3C13 |  |  |  |
|  | HMCES |  |  |  |
|  | HOXD4 |  |  |  |
|  | IFNAR2 |  |  |  |
|  | INTU |  |  |  |
|  | JAG2 |  |  |  |
|  | KCND1 |  |  |  |
|  | LARGE2 |  |  |  |
|  | LRRC23 |  |  |  |
|  | MED22 |  |  |  |
|  | MRPL21 |  |  |  |
|  | MS4A6C |  |  |  |
|  | NATD1 |  |  |  |
|  | NBL1 |  |  |  |
|  | NCCRP1 |  |  |  |
|  | NEK4 |  |  |  |
|  | NHLH1 |  |  |  |
|  | NKX6-2 |  |  |  |
|  | OSCP1 |  |  |  |
|  | PCGF6 |  |  |  |
|  | PLBD1 |  |  |  |
|  | PM20D2 |  |  |  |
|  | PRDM10 |  |  |  |
|  | RAB22A |  |  |  |
|  | REM1 |  |  |  |
|  | RHOBTB3 |  |  |  |
|  | RNF146 |  |  |  |
|  | SIM1 |  |  |  |
|  | SLC25A33 |  |  |  |
|  | SRRM3 |  |  |  |
|  | SSNA1 |  |  |  |
|  | ST18 |  |  |  |
|  | STAG3 |  |  |  |
|  | STX18 |  |  |  |
|  | SULT1C3 |  |  |  |
|  | TCAIM |  |  |  |
|  | THOC5 |  |  |  |
|  | THSD1 |  |  |  |
|  | TMEM178A |  |  |  |
|  | TMOD1 |  |  |  |
|  | TYW5 |  |  |  |
|  | UBAC2 |  |  |  |
|  | WDSUB1 |  |  |  |
|  | YIPF6 |  |  |  |
|  | ZC3H7A |  |  |  |
|  | ZNF362 |  |  |  |
|  | ZNF518A |  |  |  |
|  | ADCY10 |  |  |  |
|  | ALDH3B2 |  |  |  |
|  | ANKRD46 |  |  |  |
|  | BCDIN3D |  |  |  |
|  | BLOC1S3 |  |  |  |
|  | BOD1L1 |  |  |  |
|  | C2CD4B |  |  |  |
|  | CAPSL |  |  |  |
|  | CHAMP1 |  |  |  |
|  | CHGB |  |  |  |
|  | CHST12 |  |  |  |
|  | CNGB1 |  |  |  |
|  | CPN1 |  |  |  |
|  | CREB3L3 |  |  |  |
|  | CTC1 |  |  |  |
|  | CYP2B3 |  |  |  |
|  | DNAJC14 |  |  |  |
|  | DTD1 |  |  |  |
|  | ECSCR |  |  |  |
|  | ERAL1 |  |  |  |
|  | F12 |  |  |  |
|  | FAM174A |  |  |  |
|  | FOXN2 |  |  |  |
|  | GSTCD |  |  |  |
|  | H2AC8 |  |  |  |
|  | HBE1 |  |  |  |
|  | HSDL1 |  |  |  |
|  | IGLON5 |  |  |  |
|  | JAKMIP2 |  |  |  |
|  | KCNV1 |  |  |  |
|  | KLHL28 |  |  |  |
|  | KRT6B |  |  |  |
|  | LDAF1 |  |  |  |
|  | LRRTM1 |  |  |  |
|  | METTL18 |  |  |  |
|  | MYSM1 |  |  |  |
|  | NAT9 |  |  |  |
|  | NECAP2 |  |  |  |
|  | NHLH2 |  |  |  |
|  | NRSN1 |  |  |  |
|  | OAS1B |  |  |  |
|  | PCID2 |  |  |  |
|  | PDX1 |  |  |  |
|  | PIGH |  |  |  |
|  | PIP4K2C |  |  |  |
|  | PRPF40B |  |  |  |
|  | RBAK |  |  |  |
|  | RBM7 |  |  |  |
|  | RETREG3 |  |  |  |
|  | RUSC1 |  |  |  |
|  | SIT1 |  |  |  |
|  | SLC35A2 |  |  |  |
|  | SOCS4 |  |  |  |
|  | SREK1IP1 |  |  |  |
|  | TAFA2 |  |  |  |
|  | TM9SF1 |  |  |  |
|  | TMEM234 |  |  |  |
|  | TMEM242 |  |  |  |
|  | TRAPPC2 |  |  |  |
|  | TSTD2 |  |  |  |
|  | UBE2R2 |  |  |  |
|  | UGT2B |  |  |  |
|  | UNC13C |  |  |  |
|  | VGLL2 |  |  |  |
|  | VSX1 |  |  |  |
|  | ZNF22 |  |  |  |
|  | ZNF467 |  |  |  |
|  | ZNF521 |  |  |  |
|  | ZP2 |  |  |  |
|  | ZSCAN26 |  |  |  |
|  | AAR2 |  |  |  |
|  | AARS2 |  |  |  |
|  | ACOT5 |  |  |  |
|  | AKR1C20 |  |  |  |
|  | ANAPC15 |  |  |  |
|  | CCDC138 |  |  |  |
|  | CCDC9B |  |  |  |
|  | CCNQ |  |  |  |
|  | CDNF |  |  |  |
|  | CELSR3 |  |  |  |
|  | CHML |  |  |  |
|  | CHMP3 |  |  |  |
|  | CIART |  |  |  |
|  | CLBA1 |  |  |  |
|  | CLSTN3 |  |  |  |
|  | CNPY1 |  |  |  |
|  | COMMD2 |  |  |  |
|  | COMMD7 |  |  |  |
|  | COQ2 |  |  |  |
|  | CRB1 |  |  |  |
|  | DNAAF11 |  |  |  |
|  | DRC1 |  |  |  |
|  | EPHX4 |  |  |  |
|  | FAM78B |  |  |  |
|  | FAM81A |  |  |  |
|  | FBXL14 |  |  |  |
|  | FBXO44 |  |  |  |
|  | FBXW10 |  |  |  |
|  | FEM1A |  |  |  |
|  | GNGT2 |  |  |  |
|  | GPR34 |  |  |  |
|  | H2BC6 |  |  |  |
|  | H3C7 |  |  |  |
|  | H4C11 |  |  |  |
|  | JPH4 |  |  |  |
|  | KATNIP |  |  |  |
|  | KCNIP1 |  |  |  |
|  | KCNQ1OT1 |  |  |  |
|  | LEFTY2 |  |  |  |
|  | MARCHF8 |  |  |  |
|  | MCEMP1 |  |  |  |
|  | MMP16 |  |  |  |
|  | MORN1 |  |  |  |
|  | MPPED1 |  |  |  |
|  | MTO1 |  |  |  |
|  | MYL5 |  |  |  |
|  | NRSN2 |  |  |  |
|  | PCGF2 |  |  |  |
|  | PDCD2L |  |  |  |
|  | PKD2L1 |  |  |  |
|  | PLPP1 |  |  |  |
|  | PRAME |  |  |  |
|  | PTPRJ |  |  |  |
|  | RAG2 |  |  |  |
|  | RASSF9 |  |  |  |
|  | RHD |  |  |  |
|  | RTF1 |  |  |  |
|  | SLC12A8 |  |  |  |
|  | SYF2 |  |  |  |
|  | THAP2 |  |  |  |
|  | TIMM10B |  |  |  |
|  | TMEM79 |  |  |  |
|  | TNIP3 |  |  |  |
|  | TPPP3 |  |  |  |
|  | TRIM34 |  |  |  |
|  | TUBGCP6 |  |  |  |
|  | ULK3 |  |  |  |
|  | USB1 |  |  |  |
|  | WNT10B |  |  |  |
|  | ZCCHC9 |  |  |  |
|  | ZER1 |  |  |  |
|  | ZFP395 |  |  |  |
|  | ZNF281 |  |  |  |
|  | ZNF410 |  |  |  |
|  | ZNF598 |  |  |  |
|  | AKR7A5 |  |  |  |
|  | APOBEC3C |  |  |  |
|  | ARFRP1 |  |  |  |
|  | ASPG |  |  |  |
|  | BBS10 |  |  |  |
|  | BMT2 |  |  |  |
|  | CCDC15 |  |  |  |
|  | CCDC51 |  |  |  |
|  | CLK2 |  |  |  |
|  | COQ3 |  |  |  |
|  | CRNKL1 |  |  |  |
|  | CYB561A3 |  |  |  |
|  | CYSRT1 |  |  |  |
|  | EDRF1 |  |  |  |
|  | ENTR1 |  |  |  |
|  | ERMP1 |  |  |  |
|  | FAM161B |  |  |  |
|  | FBXL17 |  |  |  |
|  | GAR1 |  |  |  |
|  | GEMIN2 |  |  |  |
|  | GGNBP2 |  |  |  |
|  | GIN1 |  |  |  |
|  | GJA5 |  |  |  |
|  | GTF2A2 |  |  |  |
|  | GTF2E2 |  |  |  |
|  | H2BC3 |  |  |  |
|  | H3-3B |  |  |  |
|  | HERPUD2 |  |  |  |
|  | HEXIM2 |  |  |  |
|  | HMGXB4 |  |  |  |
|  | KCTD6 |  |  |  |
|  | KLHL31 |  |  |  |
|  | MIR10A |  |  |  |
|  | NEUROD1 |  |  |  |
|  | NKIRAS1 |  |  |  |
|  | ORMDL1 |  |  |  |
|  | PRRG3 |  |  |  |
|  | RALGAPB |  |  |  |
|  | RBM43 |  |  |  |
|  | RPUSD4 |  |  |  |
|  | SELENON |  |  |  |
|  | SEZ6L |  |  |  |
|  | SPATS2 |  |  |  |
|  | STARD9 |  |  |  |
|  | TIMM10 |  |  |  |
|  | TMEM267 |  |  |  |
|  | TNFRSF13B |  |  |  |
|  | TPRG1L |  |  |  |
|  | TRARG1 |  |  |  |
|  | TRNT1 |  |  |  |
|  | TTC39B |  |  |  |
|  | TTLL9 |  |  |  |
|  | TULP3 |  |  |  |
|  | TUSC2 |  |  |  |
|  | ZNF224 |  |  |  |
|  | ZRANB1 |  |  |  |
|  | ALX1 |  |  |  |
|  | ANKRD36B |  |  |  |
|  | ARMT1 |  |  |  |
|  | AUNIP |  |  |  |
|  | B3GNT9 |  |  |  |
|  | BTBD6 |  |  |  |
|  | C1ORF21 |  |  |  |
|  | CCDC181 |  |  |  |
|  | CD300LG |  |  |  |
|  | CHMP5 |  |  |  |
|  | CHST13 |  |  |  |
|  | CHST4 |  |  |  |
|  | CNTN5 |  |  |  |
|  | COL6A5 |  |  |  |
|  | DCAF12 |  |  |  |
|  | DIPK1A |  |  |  |
|  | DLX5 |  |  |  |
|  | DNAI7 |  |  |  |
|  | EFCAB6 |  |  |  |
|  | EFHB |  |  |  |
|  | EMX1 |  |  |  |
|  | ESPN |  |  |  |
|  | FAM76A |  |  |  |
|  | FBH1 |  |  |  |
|  | GP5 |  |  |  |
|  | IL20 |  |  |  |
|  | IL23R |  |  |  |
|  | KBTBD12 |  |  |  |
|  | KCTD20 |  |  |  |
|  | KLHL17 |  |  |  |
|  | KMT2D |  |  |  |
|  | LRP1B |  |  |  |
|  | MED17 |  |  |  |
|  | MICOS10 |  |  |  |
|  | MIDEAS |  |  |  |
|  | MIR130A |  |  |  |
|  | MIR23A |  |  |  |
|  | MS4A4A |  |  |  |
|  | NAA25 |  |  |  |
|  | NEK3 |  |  |  |
|  | NT5C3B |  |  |  |
|  | NWD2 |  |  |  |
|  | PABPC4L |  |  |  |
|  | PGAP3 |  |  |  |
|  | PHF21B |  |  |  |
|  | PLG |  |  |  |
|  | POMK |  |  |  |
|  | PRDM11 |  |  |  |
|  | PROKR2 |  |  |  |
|  | PSKH1 |  |  |  |
|  | QRSL1 |  |  |  |
|  | R3HDM4 |  |  |  |
|  | RAB4B |  |  |  |
|  | RRP8 |  |  |  |
|  | RXFP1 |  |  |  |
|  | SLC25A24 |  |  |  |
|  | SLC35D3 |  |  |  |
|  | SPRN |  |  |  |
|  | SRMS |  |  |  |
|  | SRSF12 |  |  |  |
|  | STYK1 |  |  |  |
|  | SUDS3 |  |  |  |
|  | TBC1D22A |  |  |  |
|  | TBC1D22B |  |  |  |
|  | TCF21 |  |  |  |
|  | TECTA |  |  |  |
|  | TMEM179B |  |  |  |
|  | TMPRSS15 |  |  |  |
|  | TNRC6C |  |  |  |
|  | TSPAN15 |  |  |  |
|  | TTC21A |  |  |  |
|  | UEVLD |  |  |  |
|  | VPS9D1 |  |  |  |
|  | WDR12 |  |  |  |
|  | ZMAT5 |  |  |  |
|  | ZNF750 |  |  |  |
|  | ALG9 |  |  |  |
|  | ASB2 |  |  |  |
|  | BBOF1 |  |  |  |
|  | BMP8A |  |  |  |
|  | BORCS6 |  |  |  |
|  | C1ORF54 |  |  |  |
|  | C2CD4C |  |  |  |
|  | C4ORF19 |  |  |  |
|  | CCDC61 |  |  |  |
|  | CD209 |  |  |  |
|  | CDH12 |  |  |  |
|  | CFAP298 |  |  |  |
|  | COX8A |  |  |  |
|  | DENND10 |  |  |  |
|  | DOP1A |  |  |  |
|  | DPM2 |  |  |  |
|  | ELAPOR2 |  |  |  |
|  | FLT3LG |  |  |  |
|  | FPGT |  |  |  |
|  | GLIS1 |  |  |  |
|  | GYPC |  |  |  |
|  | H4C2 |  |  |  |
|  | HIGD2A |  |  |  |
|  | HOXC5 |  |  |  |
|  | IL11RA |  |  |  |
|  | LRFN5 |  |  |  |
|  | LY6I |  |  |  |
|  | MIR142 |  |  |  |
|  | MRPL58 |  |  |  |
|  | MRRF |  |  |  |
|  | NDUFAF8 |  |  |  |
|  | PEX10 |  |  |  |
|  | PHF13 |  |  |  |
|  | PLEKHH3 |  |  |  |
|  | PLSCR3 |  |  |  |
|  | POC1B |  |  |  |
|  | PRPF38A |  |  |  |
|  | RASGRP4 |  |  |  |
|  | RBM12B |  |  |  |
|  | RFT1 |  |  |  |
|  | RHOT1 |  |  |  |
|  | SCGN |  |  |  |
|  | SCN11A |  |  |  |
|  | SCRN3 |  |  |  |
|  | SFMBT2 |  |  |  |
|  | SIGLEC5 |  |  |  |
|  | SLC2A4RG |  |  |  |
|  | SLFN4 |  |  |  |
|  | SMPX |  |  |  |
|  | STUM |  |  |  |
|  | SVBP |  |  |  |
|  | TBC1D13 |  |  |  |
|  | TSEN34 |  |  |  |
|  | TTC4 |  |  |  |
|  | U2AF1L4 |  |  |  |
|  | VEZT |  |  |  |
|  | WDCP |  |  |  |
|  | WDR37 |  |  |  |
|  | WWC2 |  |  |  |
|  | ZAN |  |  |  |
|  | ZBTB43 |  |  |  |
|  | ZDHHC19 |  |  |  |
|  | ADAMTS13 |  |  |  |
|  | ANAPC16 |  |  |  |
|  | B3GNT8 |  |  |  |
|  | BBS12 |  |  |  |
|  | CEP120 |  |  |  |
|  | CHST9 |  |  |  |
|  | COPS8 |  |  |  |
|  | CYP4F16 |  |  |  |
|  | DIMT1 |  |  |  |
|  | DNAAF4 |  |  |  |
|  | DNAJC22 |  |  |  |
|  | EIF2S3 |  |  |  |
|  | FAM151A |  |  |  |
|  | FANCF |  |  |  |
|  | FRG1 |  |  |  |
|  | GFM2 |  |  |  |
|  | GH |  |  |  |
|  | GLMN |  |  |  |
|  | GPR180 |  |  |  |
|  | GPSM1 |  |  |  |
|  | GSG1L |  |  |  |
|  | HAGH |  |  |  |
|  | HOXC6 |  |  |  |
|  | HSH2D |  |  |  |
|  | IFI27L1 |  |  |  |
|  | IGHA |  |  |  |
|  | IL37 |  |  |  |
|  | ISL1 |  |  |  |
|  | KCNK3 |  |  |  |
|  | KEL |  |  |  |
|  | KIAA0513 |  |  |  |
|  | LINGO2 |  |  |  |
|  | LTB4R1 |  |  |  |
|  | MASP2 |  |  |  |
|  | MED31 |  |  |  |
|  | MICA |  |  |  |
|  | MLANA |  |  |  |
|  | MPZL3 |  |  |  |
|  | MYL7 |  |  |  |
|  | NDUFS7 |  |  |  |
|  | NETO1 |  |  |  |
|  | PAK5 |  |  |  |
|  | PARP10 |  |  |  |
|  | PNLIP |  |  |  |
|  | PPP2R3A |  |  |  |
|  | PRND |  |  |  |
|  | PRSS3 |  |  |  |
|  | RASGRF2 |  |  |  |
|  | RFLNB |  |  |  |
|  | RNF166 |  |  |  |
|  | SCD5 |  |  |  |
|  | SEMA4A |  |  |  |
|  | SHISA9 |  |  |  |
|  | SLC2A13 |  |  |  |
|  | SLC4A11 |  |  |  |
|  | SPRR2D |  |  |  |
|  | SUPT3H |  |  |  |
|  | TAT |  |  |  |
|  | THADA |  |  |  |
|  | TMEM121B |  |  |  |
|  | TNP1 |  |  |  |
|  | ZNF365 |  |  |  |
|  | ADAT3 |  |  |  |
|  | AK8 |  |  |  |
|  | ANKRD49 |  |  |  |
|  | ARL16 |  |  |  |
|  | ASB10 |  |  |  |
|  | ASTL |  |  |  |
|  | BTN3A1 |  |  |  |
|  | CCDC184 |  |  |  |
|  | CEACAM20 |  |  |  |
|  | COL19A1 |  |  |  |
|  | COX18 |  |  |  |
|  | CRKL |  |  |  |
|  | DCLRE1B |  |  |  |
|  | EN1 |  |  |  |
|  | ETNK2 |  |  |  |
|  | FAM114A2 |  |  |  |
|  | FAM229B |  |  |  |
|  | FASTKD3 |  |  |  |
|  | FBXL15 |  |  |  |
|  | FBXO39 |  |  |  |
|  | FCRLB |  |  |  |
|  | FHIP1B |  |  |  |
|  | GAL3ST2 |  |  |  |
|  | GIMAP1 |  |  |  |
|  | HCAR1 |  |  |  |
|  | HOXC8 |  |  |  |
|  | HSD17B2 |  |  |  |
|  | IGIP |  |  |  |
|  | IL36A |  |  |  |
|  | INTS13 |  |  |  |
|  | JMJD8 |  |  |  |
|  | KLRC1 |  |  |  |
|  | LIN28B |  |  |  |
|  | LRRTM3 |  |  |  |
|  | MMAA |  |  |  |
|  | MRFAP1 |  |  |  |
|  | NAPEPLD |  |  |  |
|  | NDUFA9 |  |  |  |
|  | NPAS3 |  |  |  |
|  | NPAT |  |  |  |
|  | NPHP3 |  |  |  |
|  | PADI3 |  |  |  |
|  | PCDHB16 |  |  |  |
|  | PCNX2 |  |  |  |
|  | PDIK1L |  |  |  |
|  | PLEKHG3 |  |  |  |
|  | PPP1R42 |  |  |  |
|  | PPP4R3A |  |  |  |
|  | RGN |  |  |  |
|  | SLC25A18 |  |  |  |
|  | SLC25A43 |  |  |  |
|  | SLC9A4 |  |  |  |
|  | SLN |  |  |  |
|  | SOCS7 |  |  |  |
|  | STX19 |  |  |  |
|  | SYNPO2 |  |  |  |
|  | TAF3 |  |  |  |
|  | TARBP1 |  |  |  |
|  | TRIM66 |  |  |  |
|  | TXK |  |  |  |
|  | ZC2HC1C |  |  |  |
|  | ZNF296 |  |  |  |
|  | ZNF451 |  |  |  |
|  | ZNF706 |  |  |  |
|  | ACMSD |  |  |  |
|  | ADGRF4 |  |  |  |
|  | AGO4 |  |  |  |
|  | ALX4 |  |  |  |
|  | ARV1 |  |  |  |
|  | ATP2C2 |  |  |  |
|  | CCDC103 |  |  |  |
|  | CCDC174 |  |  |  |
|  | CNPPD1 |  |  |  |
|  | COLQ |  |  |  |
|  | CYP2F4 |  |  |  |
|  | DNAJA3 |  |  |  |
|  | DNAL1 |  |  |  |
|  | EML6 |  |  |  |
|  | FAM50A |  |  |  |
|  | GLUD1 |  |  |  |
|  | GSKIP |  |  |  |
|  | KIF3A |  |  |  |
|  | KLHL15 |  |  |  |
|  | LEPROT |  |  |  |
|  | MAP4K1 |  |  |  |
|  | MAPRE3 |  |  |  |
|  | MIOX |  |  |  |
|  | MIR140 |  |  |  |
|  | MIR34B |  |  |  |
|  | NSUN3 |  |  |  |
|  | OXSM |  |  |  |
|  | PARP8 |  |  |  |
|  | PAXBP1 |  |  |  |
|  | PCLO |  |  |  |
|  | PLEKHO1 |  |  |  |
|  | ROM1 |  |  |  |
|  | SAMD14 |  |  |  |
|  | SEC22C |  |  |  |
|  | SETDB2 |  |  |  |
|  | SLC17A8 |  |  |  |
|  | SPACA9 |  |  |  |
|  | TCERG1L |  |  |  |
|  | THUMPD2 |  |  |  |
|  | TP53RK |  |  |  |
|  | UCKL1 |  |  |  |
|  | UHMK1 |  |  |  |
|  | ZMYM4 |  |  |  |
|  | ZMYND19 |  |  |  |
|  | ADNP2 |  |  |  |
|  | AGPAT9 |  |  |  |
|  | ATP9B |  |  |  |
|  | BBLN |  |  |  |
|  | BUD13 |  |  |  |
|  | C1QL3 |  |  |  |
|  | CALCRL |  |  |  |
|  | CCDC134 |  |  |  |
|  | CD207 |  |  |  |
|  | CD37 |  |  |  |
|  | CNTNAP4 |  |  |  |
|  | COL4A4 |  |  |  |
|  | CYP2C18 |  |  |  |
|  | CYP4F1 |  |  |  |
|  | CYP4F22 |  |  |  |
|  | DPCD |  |  |  |
|  | ENTPD6 |  |  |  |
|  | EPB41L4A |  |  |  |
|  | EPGN |  |  |  |
|  | FAM227B |  |  |  |
|  | FBXO42 |  |  |  |
|  | FCER2 |  |  |  |
|  | FOXL1 |  |  |  |
|  | GDPGP1 |  |  |  |
|  | GPA33 |  |  |  |
|  | GPR173 |  |  |  |
|  | GPR50 |  |  |  |
|  | H2AC1 |  |  |  |
|  | HPX |  |  |  |
|  | IFT43 |  |  |  |
|  | IL17F |  |  |  |
|  | INSRR |  |  |  |
|  | KANSL3 |  |  |  |
|  | KCNG2 |  |  |  |
|  | KGD4 |  |  |  |
|  | KLHL42 |  |  |  |
|  | KREMEN2 |  |  |  |
|  | KRT20 |  |  |  |
|  | LTN1 |  |  |  |
|  | LYSMD1 |  |  |  |
|  | MIEN1 |  |  |  |
|  | MIR100HG |  |  |  |
|  | MIR342 |  |  |  |
|  | MOCS3 |  |  |  |
|  | MPP4 |  |  |  |
|  | MSL2 |  |  |  |
|  | NMUR1 |  |  |  |
|  | NOVA1 |  |  |  |
|  | OTUD3 |  |  |  |
|  | OVAL |  |  |  |
|  | PARS2 |  |  |  |
|  | PDLIM4 |  |  |  |
|  | POLR2J |  |  |  |
|  | PPP2R5A |  |  |  |
|  | PRR14L |  |  |  |
|  | PSTK |  |  |  |
|  | RAB33B |  |  |  |
|  | RBSN |  |  |  |
|  | RGS13 |  |  |  |
|  | RNF215 |  |  |  |
|  | SERPINB10 |  |  |  |
|  | SIRT6 |  |  |  |
|  | SLC25A40 |  |  |  |
|  | SMIM11 |  |  |  |
|  | SNHG32 |  |  |  |
|  | SNURF |  |  |  |
|  | SS18 |  |  |  |
|  | STUB1 |  |  |  |
|  | TAC3 |  |  |  |
|  | TANGO6 |  |  |  |
|  | TASOR2 |  |  |  |
|  | TBX19 |  |  |  |
|  | TIFAB |  |  |  |
|  | TM6SF1 |  |  |  |
|  | TMEM181 |  |  |  |
|  | TMEM219 |  |  |  |
|  | TP53TG1 |  |  |  |
|  | UTS2R |  |  |  |
|  | WDR48 |  |  |  |
|  | WDR89 |  |  |  |
|  | ZBTB9 |  |  |  |
|  | ACTL6B |  |  |  |
|  | ADAMDEC1 |  |  |  |
|  | AHSA2 |  |  |  |
|  | ANAPC13 |  |  |  |
|  | ANKRD35 |  |  |  |
|  | AP5B1 |  |  |  |
|  | ARL14EP |  |  |  |
|  | ASB6 |  |  |  |
|  | BACH1 |  |  |  |
|  | CARNS1 |  |  |  |
|  | CCDC57 |  |  |  |
|  | CCDC66 |  |  |  |
|  | CCDC71 |  |  |  |
|  | CDK15 |  |  |  |
|  | CFAP410 |  |  |  |
|  | CLCN1 |  |  |  |
|  | CNOT11 |  |  |  |
|  | COX14 |  |  |  |
|  | COX8B |  |  |  |
|  | DBR1 |  |  |  |
|  | DCAF12L2 |  |  |  |
|  | DNAI2 |  |  |  |
|  | ERI3 |  |  |  |
|  | FAM50B |  |  |  |
|  | GASK1A |  |  |  |
|  | GCNA |  |  |  |
|  | GKN2 |  |  |  |
|  | GPATCH3 |  |  |  |
|  | GPR3 |  |  |  |
|  | HS2ST1 |  |  |  |
|  | IFT22 |  |  |  |
|  | INKA1 |  |  |  |
|  | IQCH |  |  |  |
|  | KLRC2 |  |  |  |
|  | LOXHD1 |  |  |  |
|  | LY6G |  |  |  |
|  | MC5R |  |  |  |
|  | MIR25 |  |  |  |
|  | MIR335 |  |  |  |
|  | MIRLET7B |  |  |  |
|  | NAA16 |  |  |  |
|  | NDUFA10 |  |  |  |
|  | P4HA3 |  |  |  |
|  | PIGG |  |  |  |
|  | PNPLA1 |  |  |  |
|  | POGK |  |  |  |
|  | PPY |  |  |  |
|  | PRR14 |  |  |  |
|  | PTGDS |  |  |  |
|  | RETNLB |  |  |  |
|  | SNRNP48 |  |  |  |
|  | SS18L1 |  |  |  |
|  | SYPL2 |  |  |  |
|  | THAP4 |  |  |  |
|  | THSD7A |  |  |  |
|  | TMEM126B |  |  |  |
|  | TMEM229A |  |  |  |
|  | TNFSF18 |  |  |  |
|  | TSTD3 |  |  |  |
|  | UBXN10 |  |  |  |
|  | USP38 |  |  |  |
|  | VPS26C |  |  |  |
|  | WDR83 |  |  |  |
|  | ZDHHC4 |  |  |  |
|  | ZKSCAN7 |  |  |  |
|  | ZNF146 |  |  |  |
|  | ZNF189 |  |  |  |
|  | ZNF23 |  |  |  |
|  | ZNF263 |  |  |  |
|  | ZNF562 |  |  |  |
|  | ZNF787 |  |  |  |
|  | BCL2A1D |  |  |  |
|  | BPI |  |  |  |
|  | C4BPB |  |  |  |
|  | C8G |  |  |  |
|  | CAMKMT |  |  |  |
|  | CCDC152 |  |  |  |
|  | CDH26 |  |  |  |
|  | CFAP210 |  |  |  |
|  | CRX |  |  |  |
|  | CYB5RL |  |  |  |
|  | DAB2IP |  |  |  |
|  | ERBIN |  |  |  |
|  | FAAP100 |  |  |  |
|  | GBX1 |  |  |  |
|  | GK5 |  |  |  |
|  | GZF1 |  |  |  |
|  | IRX6 |  |  |  |
|  | ITGA9 |  |  |  |
|  | KBTBD6 |  |  |  |
|  | MIOS |  |  |  |
|  | MIR126 |  |  |  |
|  | OFD1 |  |  |  |
|  | PPP1R3D |  |  |  |
|  | QNG1 |  |  |  |
|  | RBM48 |  |  |  |
|  | RFX6 |  |  |  |
|  | RNASE1 |  |  |  |
|  | RPP30 |  |  |  |
|  | SCN3A |  |  |  |
|  | SERHL2 |  |  |  |
|  | SERPINA3G |  |  |  |
|  | SIX2 |  |  |  |
|  | SKAP1 |  |  |  |
|  | SLAMF6 |  |  |  |
|  | TCF24 |  |  |  |
|  | TIMM13 |  |  |  |
|  | TSHZ2 |  |  |  |
|  | VWC2 |  |  |  |
|  | ZBTB34 |  |  |  |
|  | ZNF114 |  |  |  |
|  | ZNF516 |  |  |  |
|  | ZSCAN2 |  |  |  |
|  | ZSWIM8 |  |  |  |
|  | ADAMTS12 |  |  |  |
|  | ATRNL1 |  |  |  |
|  | CIAO3 |  |  |  |
|  | CITED1 |  |  |  |
|  | CRNDE |  |  |  |
|  | DMPK |  |  |  |
|  | ENKD1 |  |  |  |
|  | FBXO34 |  |  |  |
|  | GUCA2B |  |  |  |
|  | HMOX1A |  |  |  |
|  | IGHG1 |  |  |  |
|  | LRCH2 |  |  |  |
|  | LRP11 |  |  |  |
|  | MIR10B |  |  |  |
|  | MIR18A |  |  |  |
|  | MPC2 |  |  |  |
|  | PAXX |  |  |  |
|  | RANBP2 |  |  |  |
|  | RWDD1 |  |  |  |
|  | SAMD13 |  |  |  |
|  | SLC29A4 |  |  |  |
|  | USP43 |  |  |  |
|  | UTP23 |  |  |  |
|  | XIRP2 |  |  |  |
|  | ZKSCAN1 |  |  |  |
|  | ZNF580 |  |  |  |
|  | ZNF692 |  |  |  |
|  | ZSWIM3 |  |  |  |
|  | ACAA1A |  |  |  |
|  | AFG3L1 |  |  |  |
|  | AGXT2 |  |  |  |
|  | AKNAD1 |  |  |  |
|  | ANO8 |  |  |  |
|  | ARHGEF10 |  |  |  |
|  | BRD10 |  |  |  |
|  | BTBD3 |  |  |  |
|  | CCDC170 |  |  |  |
|  | CPNE9 |  |  |  |
|  | DBF4B |  |  |  |
|  | DENND5B |  |  |  |
|  | DOCK6 |  |  |  |
|  | DTD2 |  |  |  |
|  | E2F4 |  |  |  |
|  | EID2B |  |  |  |
|  | FBXO24 |  |  |  |
|  | FHIP2A |  |  |  |
|  | FSD2 |  |  |  |
|  | GABPA |  |  |  |
|  | GPR156 |  |  |  |
|  | INTS11 |  |  |  |
|  | KCNG4 |  |  |  |
|  | LRRC8A |  |  |  |
|  | LRRIQ3 |  |  |  |
|  | MBIP |  |  |  |
|  | MED28 |  |  |  |
|  | MIR130B |  |  |  |
|  | MIR1915HG |  |  |  |
|  | MIR203 |  |  |  |
|  | MIR23B |  |  |  |
|  | MIR31 |  |  |  |
|  | MIR483 |  |  |  |
|  | MIRLET7D |  |  |  |
|  | MTG2 |  |  |  |
|  | NCKAP5 |  |  |  |
|  | NEU4 |  |  |  |
|  | PEX5L |  |  |  |
|  | PLEKHA7 |  |  |  |
|  | PMFBP1 |  |  |  |
|  | RC3H2 |  |  |  |
|  | RIMS2 |  |  |  |
|  | S100A12 |  |  |  |
|  | SLC22A16 |  |  |  |
|  | SLC30A2 |  |  |  |
|  | TARS3 |  |  |  |
|  | TBC1D12 |  |  |  |
|  | TIMM44 |  |  |  |
|  | TMEM14C |  |  |  |
|  | TMEM221 |  |  |  |
|  | TOGARAM1 |  |  |  |
|  | TREML4 |  |  |  |
|  | TRPM3 |  |  |  |
|  | UBXN2B |  |  |  |
|  | WDR5B |  |  |  |
|  | XXYLT1 |  |  |  |
|  | YJU2B |  |  |  |
|  | ZFP37 |  |  |  |
|  | ZNF83 |  |  |  |
|  | ACTRT3 |  |  |  |
|  | ANKRD34B |  |  |  |
|  | ARFGEF2 |  |  |  |
|  | ARHGAP42 |  |  |  |
|  | ATP6V1E1 |  |  |  |
|  | CCDC190 |  |  |  |
|  | CCL18 |  |  |  |
|  | CDKL5 |  |  |  |
|  | CFAP46 |  |  |  |
|  | DIS3L |  |  |  |
|  | FAM200C |  |  |  |
|  | GGN |  |  |  |
|  | IFI27L2 |  |  |  |
|  | KCNK7 |  |  |  |
|  | KCNS2 |  |  |  |
|  | KCTD21 |  |  |  |
|  | LINGO3 |  |  |  |
|  | LRRN4CL |  |  |  |
|  | MAT1A |  |  |  |
|  | MED19 |  |  |  |
|  | MIR100 |  |  |  |
|  | MIR1224 |  |  |  |
|  | MS4A4D |  |  |  |
|  | MYF6 |  |  |  |
|  | MZB1 |  |  |  |
|  | NOX3 |  |  |  |
|  | POU2AF3 |  |  |  |
|  | PPP1R27 |  |  |  |
|  | SAXO2 |  |  |  |
|  | SERPINB4 |  |  |  |
|  | SNORD22 |  |  |  |
|  | STYXL2 |  |  |  |
|  | TADA1 |  |  |  |
|  | TIMM23 |  |  |  |
|  | TM4SF18 |  |  |  |
|  | TMEM232 |  |  |  |
|  | TRAPPC13 |  |  |  |
|  | TTC32 |  |  |  |
|  | UBTD2 |  |  |  |
|  | UMODL1 |  |  |  |
|  | ZDHHC23 |  |  |  |
|  | ACAD10 |  |  |  |
|  | ADH6 |  |  |  |
|  | ARK2N |  |  |  |
|  | ATP6V1G3 |  |  |  |
|  | C2ORF68 |  |  |  |
|  | CKMT1A |  |  |  |
|  | DCP1B |  |  |  |
|  | DYRK4 |  |  |  |
|  | EPN3 |  |  |  |
|  | H4C12 |  |  |  |
|  | KCNJ4 |  |  |  |
|  | KCTD8 |  |  |  |
|  | KLHDC7B |  |  |  |
|  | MIR532 |  |  |  |
|  | MROH7 |  |  |  |
|  | NBEAL1 |  |  |  |
|  | NCR3LG1 |  |  |  |
|  | NOPCHAP1 |  |  |  |
|  | OGFOD3 |  |  |  |
|  | PRTG |  |  |  |
|  | RAB1B |  |  |  |
|  | RAB40C |  |  |  |
|  | RABL2A |  |  |  |
|  | RAET1E |  |  |  |
|  | RAMAC |  |  |  |
|  | RNF113A |  |  |  |
|  | SLC9A7 |  |  |  |
|  | TM6SF2 |  |  |  |
|  | UBXN2A |  |  |  |
|  | ZBTB6 |  |  |  |
|  | ZFP1 |  |  |  |
|  | ZNF287 |  |  |  |
|  | ZNF32 |  |  |  |
|  | ZNF682 |  |  |  |
|  | AHSA2P |  |  |  |
|  | B3GLCT |  |  |  |
|  | BORCS7 |  |  |  |
|  | C12ORF57 |  |  |  |
|  | CARNMT1 |  |  |  |
|  | CASP5 |  |  |  |
|  | CD101 |  |  |  |
|  | CMTR2 |  |  |  |
|  | DANCR |  |  |  |
|  | FAM219B |  |  |  |
|  | GAL3ST4 |  |  |  |
|  | GOLGA7B |  |  |  |
|  | GSDMC |  |  |  |
|  | GTF3C5 |  |  |  |
|  | H2BC17 |  |  |  |
|  | KIAA0232 |  |  |  |
|  | KIAA1614 |  |  |  |
|  | KLHL10 |  |  |  |
|  | KLK2 |  |  |  |
|  | KPTN |  |  |  |
|  | LHFPL3 |  |  |  |
|  | LILRB1 |  |  |  |
|  | LRTOMT |  |  |  |
|  | LTO1 |  |  |  |
|  | MIR186 |  |  |  |
|  | MIRLET7E |  |  |  |
|  | NCBP3 |  |  |  |
|  | NOL4L |  |  |  |
|  | NOM1 |  |  |  |
|  | NPIPA1 |  |  |  |
|  | PCDHB14 |  |  |  |
|  | PIERCE2 |  |  |  |
|  | PPFIA2 |  |  |  |
|  | RAB15 |  |  |  |
|  | RNF32 |  |  |  |
|  | SCARNA17 |  |  |  |
|  | SCIMP |  |  |  |
|  | SH2D3A |  |  |  |
|  | SNX31 |  |  |  |
|  | SPMIP6 |  |  |  |
|  | STFA3 |  |  |  |
|  | SURF6 |  |  |  |
|  | TMED6 |  |  |  |
|  | TMEM119 |  |  |  |
|  | TMEM198B |  |  |  |
|  | TRABD2A |  |  |  |
|  | UBAP1L |  |  |  |
|  | WDR83OS |  |  |  |
|  | XKR6 |  |  |  |
|  | ZDHHC11 |  |  |  |
|  | ZFP53 |  |  |  |
|  | ZNF397 |  |  |  |
|  | ZSCAN9 |  |  |  |
|  | APC2 |  |  |  |
|  | DGKE |  |  |  |
|  | GGTA1 |  |  |  |
|  | GNAT2 |  |  |  |
|  | HTR2A |  |  |  |
|  | LONP2 |  |  |  |
|  | LOXL1 |  |  |  |
|  | SLC7A10 |  |  |  |
|  | ZBTB7B |  |  |  |
|  | ADGRG7 |  |  |  |
|  | AMHR2 |  |  |  |
|  | ARL6IP6 |  |  |  |
|  | ART4 |  |  |  |
|  | ASIC5 |  |  |  |
|  | BTBD19 |  |  |  |
|  | CLDN23 |  |  |  |
|  | CYP27C1 |  |  |  |
|  | DYDC2 |  |  |  |
|  | EMX2OS |  |  |  |
|  | F8A1 |  |  |  |
|  | FAM222B |  |  |  |
|  | FNDC7 |  |  |  |
|  | FOLR1 |  |  |  |
|  | FUOM |  |  |  |
|  | GAPT |  |  |  |
|  | GBGT1 |  |  |  |
|  | H4C15 |  |  |  |
|  | HMX1 |  |  |  |
|  | IGH |  |  |  |
|  | IGLV1 |  |  |  |
|  | KLHL36 |  |  |  |
|  | KLK7 |  |  |  |
|  | KLRG2 |  |  |  |
|  | LINC-PINT |  |  |  |
|  | LRCH3 |  |  |  |
|  | MCMDC2 |  |  |  |
|  | MIR185 |  |  |  |
|  | MORC1 |  |  |  |
|  | MUL1 |  |  |  |
|  | MYO15A |  |  |  |
|  | NDUFAF5 |  |  |  |
|  | NLRC3 |  |  |  |
|  | NRDE2 |  |  |  |
|  | NT5C1A |  |  |  |
|  | NUDT17 |  |  |  |
|  | SERPINB13 |  |  |  |
|  | SKIC2 |  |  |  |
|  | SMIM20 |  |  |  |
|  | STAMBP |  |  |  |
|  | TAL2 |  |  |  |
|  | UPK2 |  |  |  |
|  | UQCC3 |  |  |  |
|  | WDR7 |  |  |  |
|  | WFDC17 |  |  |  |
|  | ZNF142 |  |  |  |
|  | ZNF264 |  |  |  |
|  | ZNF282 |  |  |  |
|  | ZNF544 |  |  |  |
|  | AFM |  |  |  |
|  | ALT |  |  |  |
|  | BCL2A1B |  |  |  |
|  | BEST2 |  |  |  |
|  | BTN2A2 |  |  |  |
|  | C6ORF132 |  |  |  |
|  | CABP4 |  |  |  |
|  | CCZ1 |  |  |  |
|  | CHMP4A |  |  |  |
|  | CIMAP1C |  |  |  |
|  | COL25A1 |  |  |  |
|  | CTLA2B |  |  |  |
|  | CTTNBP2 |  |  |  |
|  | CYP4F12 |  |  |  |
|  | DLEU7 |  |  |  |
|  | DRC7 |  |  |  |
|  | DZANK1 |  |  |  |
|  | EFCAB3 |  |  |  |
|  | ERICH2 |  |  |  |
|  | FAM151B |  |  |  |
|  | GCSAM |  |  |  |
|  | GPAT2 |  |  |  |
|  | GPR61 |  |  |  |
|  | GPRASP3 |  |  |  |
|  | H4C4 |  |  |  |
|  | HTR5A |  |  |  |
|  | IFI205 |  |  |  |
|  | IFNG1 |  |  |  |
|  | IL25 |  |  |  |
|  | IPPK |  |  |  |
|  | KCTD16 |  |  |  |
|  | KICS2 |  |  |  |
|  | KTI12 |  |  |  |
|  | MIR324 |  |  |  |
|  | NDUFAF7 |  |  |  |
|  | NKX2-6 |  |  |  |
|  | PPP1R3F |  |  |  |
|  | PSME3IP1 |  |  |  |
|  | REG3A |  |  |  |
|  | RSPH14 |  |  |  |
|  | SGCD |  |  |  |
|  | SIRPB2 |  |  |  |
|  | SLC35E2B |  |  |  |
|  | TAF4 |  |  |  |
|  | TBATA |  |  |  |
|  | TRHDE |  |  |  |
|  | TRIM50 |  |  |  |
|  | TTBK2 |  |  |  |
|  | VPS51 |  |  |  |
|  | WDR20 |  |  |  |
|  | XKR9 |  |  |  |
|  | XRN1 |  |  |  |
|  | ZFP354A |  |  |  |
|  | ZKSCAN4 |  |  |  |
|  | ZNF160 |  |  |  |
|  | ZNF438 |  |  |  |
|  | ZNF507 |  |  |  |
|  | ZNF600 |  |  |  |
|  | ZNF641 |  |  |  |
|  | ZNF689 |  |  |  |
|  | ZSCAN22 |  |  |  |
|  | ATP12A |  |  |  |
|  | BCS1L |  |  |  |
|  | C21ORF91 |  |  |  |
|  | C6ORF62 |  |  |  |
|  | CLDN11 |  |  |  |
|  | CLEC14A |  |  |  |
|  | CSTPP1 |  |  |  |
|  | DEDD |  |  |  |
|  | FAM184A |  |  |  |
|  | GNB3 |  |  |  |
|  | H2BC7 |  |  |  |
|  | IGSF9B |  |  |  |
|  | LINS1 |  |  |  |
|  | MAL |  |  |  |
|  | MC4R |  |  |  |
|  | MIR107 |  |  |  |
|  | MTERF1 |  |  |  |
|  | PACC1 |  |  |  |
|  | RBKS |  |  |  |
|  | RUNDC1 |  |  |  |
|  | SNHG3 |  |  |  |
|  | SP140L |  |  |  |
|  | SRFBP1 |  |  |  |
|  | SUSD1 |  |  |  |
|  | TRIM54 |  |  |  |
|  | ZNF12 |  |  |  |
|  | ZNF45 |  |  |  |
|  | ZNF627 |  |  |  |
|  | ANXA8L1 |  |  |  |
|  | C14ORF93 |  |  |  |
|  | C6ORF47 |  |  |  |
|  | CAPNS2 |  |  |  |
|  | CER1 |  |  |  |
|  | CLDN16 |  |  |  |
|  | CXORF38 |  |  |  |
|  | CYP4F6 |  |  |  |
|  | DHFR2 |  |  |  |
|  | DLEC1 |  |  |  |
|  | FABP2 |  |  |  |
|  | GPALPP1 |  |  |  |
|  | IL1F10 |  |  |  |
|  | KIFC2 |  |  |  |
|  | KLRC3 |  |  |  |
|  | LINC00312 |  |  |  |
|  | LUZP2 |  |  |  |
|  | MBOAT4 |  |  |  |
|  | MGL2 |  |  |  |
|  | MRPS2 |  |  |  |
|  | MTRF1 |  |  |  |
|  | NDUFA4L2 |  |  |  |
|  | NPY4R |  |  |  |
|  | PSMA8 |  |  |  |
|  | REP15 |  |  |  |
|  | SERHL |  |  |  |
|  | SERPINB12 |  |  |  |
|  | SLC46A2 |  |  |  |
|  | STK31 |  |  |  |
|  | SUPT4H1 |  |  |  |
|  | TREML2 |  |  |  |
|  | TTC36 |  |  |  |
|  | CDKL2 |  |  |  |
|  | CDYL2 |  |  |  |
|  | COA1 |  |  |  |
|  | HIGD1A |  |  |  |
|  | ITPRIPL1 |  |  |  |
|  | MIR362 |  |  |  |
|  | MIR425 |  |  |  |
|  | NBPF1 |  |  |  |
|  | NHSL3 |  |  |  |
|  | PIK3R6 |  |  |  |
|  | SPPL2B |  |  |  |
|  | TRIM68 |  |  |  |
|  | TTLL4 |  |  |  |
|  | ZNF561 |  |  |  |
|  | ZNF79 |  |  |  |
|  | ANKRD53 |  |  |  |
|  | ANKS4B |  |  |  |
|  | BEND7 |  |  |  |
|  | BTNL8 |  |  |  |
|  | C2ORF49 |  |  |  |
|  | CALCB |  |  |  |
|  | CCDC110 |  |  |  |
|  | CCDC38 |  |  |  |
|  | CRIP1 |  |  |  |
|  | EFCAB12 |  |  |  |
|  | EGLN2 |  |  |  |
|  | ENO4 |  |  |  |
|  | FDXACB1 |  |  |  |
|  | GLP2R |  |  |  |
|  | GPATCH1 |  |  |  |
|  | GPRC5D |  |  |  |
|  | H2AC17 |  |  |  |
|  | KIAA0319L |  |  |  |
|  | KIAA1143 |  |  |  |
|  | KLK13 |  |  |  |
|  | KRT81 |  |  |  |
|  | LRRIQ1 |  |  |  |
|  | MCF2L2 |  |  |  |
|  | OAS1G |  |  |  |
|  | PCDHGA4 |  |  |  |
|  | RSKR |  |  |  |
|  | SBK3 |  |  |  |
|  | SOX2-OT |  |  |  |
|  | SOX30 |  |  |  |
|  | SPATS1 |  |  |  |
|  | STEEP1 |  |  |  |
|  | TBCCD1 |  |  |  |
|  | YIPF7 |  |  |  |
|  | ZFP24 |  |  |  |
|  | ZNF248 |  |  |  |
|  | ZNF280D |  |  |  |
|  | ZNF317 |  |  |  |
|  | ZNF354A |  |  |  |
|  | ZNF660 |  |  |  |
|  | ZNF688 |  |  |  |
|  | ZNF70 |  |  |  |
|  | ZNF804A |  |  |  |
|  | ZNF84 |  |  |  |
|  | APOBEC3A |  |  |  |
|  | APOC2 |  |  |  |
|  | ELOVL4 |  |  |  |
|  | FABP9 |  |  |  |
|  | FENDRR |  |  |  |
|  | FGF11 |  |  |  |
|  | GAN |  |  |  |
|  | GINS1 |  |  |  |
|  | GRM6 |  |  |  |
|  | H2BC10 |  |  |  |
|  | HBB-B2 |  |  |  |
|  | HBD |  |  |  |
|  | IZUMO1R |  |  |  |
|  | LAYN |  |  |  |
|  | LIPT1 |  |  |  |
|  | LMLN |  |  |  |
|  | LPA |  |  |  |
|  | MAP1LC3C |  |  |  |
|  | MIR128-1 |  |  |  |
|  | MIR330 |  |  |  |
|  | MIR331 |  |  |  |
|  | MPIG6B |  |  |  |
|  | NKX3-2 |  |  |  |
|  | NPFF |  |  |  |
|  | NPIPB3 |  |  |  |
|  | PDE6G |  |  |  |
|  | PER1B |  |  |  |
|  | PGBD1 |  |  |  |
|  | PILRB2 |  |  |  |
|  | PPARB |  |  |  |
|  | RSBN1L |  |  |  |
|  | RSPH10B |  |  |  |
|  | RWDD4 |  |  |  |
|  | SHROOM4 |  |  |  |
|  | SPDL1 |  |  |  |
|  | TEPSIN |  |  |  |
|  | TMEM86B |  |  |  |
|  | TMPRSS5 |  |  |  |
|  | ZBED2 |  |  |  |
|  | ZBTB3 |  |  |  |
|  | ZNF436-AS1 |  |  |  |
|  | ZNF557 |  |  |  |
|  | ZNF572 |  |  |  |
|  | ZNF587 |  |  |  |
|  | ZNF654 |  |  |  |
|  | ADPRH |  |  |  |
|  | AP1S1 |  |  |  |
|  | CDC14B |  |  |  |
|  | COG8 |  |  |  |
|  | DDX41 |  |  |  |
|  | DLGAP2 |  |  |  |
|  | IAPP |  |  |  |
|  | LAIR1 |  |  |  |
|  | NALCN |  |  |  |
|  | RPS6KA2 |  |  |  |
|  | SLC26A11 |  |  |  |
|  | TXLNB |  |  |  |
|  | ZMYND10 |  |  |  |
|  | ADAM21 |  |  |  |
|  | AGBL3 |  |  |  |
|  | ANKRD13C-DT |  |  |  |
|  | C1ORF159 |  |  |  |
|  | CARD16 |  |  |  |
|  | CENPC |  |  |  |
|  | CHRNB2 |  |  |  |
|  | CNGA4 |  |  |  |
|  | CRACR2A |  |  |  |
|  | CST1 |  |  |  |
|  | DMP1 |  |  |  |
|  | EAR1 |  |  |  |
|  | EBLN2 |  |  |  |
|  | EPD |  |  |  |
|  | FAM156A |  |  |  |
|  | FBXL8 |  |  |  |
|  | GPR63 |  |  |  |
|  | H3F4 |  |  |  |
|  | H4C5 |  |  |  |
|  | HSD3B |  |  |  |
|  | ICOSL |  |  |  |
|  | KLHL1 |  |  |  |
|  | KPNA7 |  |  |  |
|  | KRT31 |  |  |  |
|  | LRRC10 |  |  |  |
|  | MIR30B |  |  |  |
|  | MIRLET7C |  |  |  |
|  | MRPL2 |  |  |  |
|  | MTCL2 |  |  |  |
|  | NICOL1 |  |  |  |
|  | PCDHA11 |  |  |  |
|  | PPEF2 |  |  |  |
|  | SERPINA4 |  |  |  |
|  | SH2D6 |  |  |  |
|  | TIGIT |  |  |  |
|  | TMIGD1 |  |  |  |
|  | TNFRSF17 |  |  |  |
|  | VCF2 |  |  |  |
|  | ZNF225 |  |  |  |
|  | ZNF324 |  |  |  |
|  | ZNF33A |  |  |  |
|  | ZNF384 |  |  |  |
|  | ZNF746 |  |  |  |
|  | ZNF91 |  |  |  |
|  | ZSCAN5A |  |  |  |
|  | ATOH1 |  |  |  |
|  | C14ORF28 |  |  |  |
|  | C8ORF58 |  |  |  |
|  | CADM2 |  |  |  |
|  | CHRNA7 |  |  |  |
|  | FAM200B |  |  |  |
|  | GCM1 |  |  |  |
|  | MIR374B |  |  |  |
|  | MRPL55 |  |  |  |
|  | MRPL57 |  |  |  |
|  | OIT3 |  |  |  |
|  | PVRL1 |  |  |  |
|  | SCGB1D2 |  |  |  |
|  | SLC13A2 |  |  |  |
|  | TACR3 |  |  |  |
|  | USP37 |  |  |  |
|  | ZFP82 |  |  |  |
|  | ZNF131 |  |  |  |
|  | ZNF180 |  |  |  |
|  | ZNF200 |  |  |  |
|  | ZNF226 |  |  |  |
|  | ZNF286A |  |  |  |
|  | ZNF37A |  |  |  |
|  | ZNF469 |  |  |  |
|  | ZNF518B |  |  |  |
|  | ZNF92 |  |  |  |
|  | ZSCAN16 |  |  |  |
|  | AKR1B15 |  |  |  |
|  | ANKRD63 |  |  |  |
|  | C5ORF15 |  |  |  |
|  | CCL15 |  |  |  |
|  | CPO |  |  |  |
|  | CTRL |  |  |  |
|  | DMRTB1 |  |  |  |
|  | ERMARD |  |  |  |
|  | ERVMER34-1 |  |  |  |
|  | GCH2 |  |  |  |
|  | HEPHL1 |  |  |  |
|  | ICAM3 |  |  |  |
|  | LDHAL6B |  |  |  |
|  | LRP4 |  |  |  |
|  | MIR503HG |  |  |  |
|  | MIR9-1HG |  |  |  |
|  | PCP4L1 |  |  |  |
|  | POLR2J2 |  |  |  |
|  | QRFPR |  |  |  |
|  | RPL26L1 |  |  |  |
|  | STARD6 |  |  |  |
|  | STFA2 |  |  |  |
|  | SYT16 |  |  |  |
|  | THRA.L |  |  |  |
|  | TMEM170A |  |  |  |
|  | TRIM30A |  |  |  |
|  | TSACC |  |  |  |
|  | TSKS |  |  |  |
|  | WDR93 |  |  |  |
|  | ZNF414 |  |  |  |
|  | ZNF513 |  |  |  |
|  | ZNF543 |  |  |  |
|  | ZNF549 |  |  |  |
|  | ZNF761 |  |  |  |
|  | BTN2A1 |  |  |  |
|  | CA5A |  |  |  |
|  | CBLIF |  |  |  |
|  | FUT3 |  |  |  |
|  | GRIA1 |  |  |  |
|  | HAPLN2 |  |  |  |
|  | OTP |  |  |  |
|  | PROZ |  |  |  |
|  | SCRT1 |  |  |  |
|  | TLR10 |  |  |  |
|  | ZFP69 |  |  |  |
|  | ZFY |  |  |  |
|  | ZNF211 |  |  |  |
|  | ZNF485 |  |  |  |
|  | ZNF514 |  |  |  |
|  | ZNF569 |  |  |  |
|  | ANKRD39 |  |  |  |
|  | AOX4 |  |  |  |
|  | ASIC3 |  |  |  |
|  | ATF7IP2 |  |  |  |
|  | C1QL2 |  |  |  |
|  | C22ORF39 |  |  |  |
|  | CDHR3 |  |  |  |
|  | CFAP47 |  |  |  |
|  | CFAP58 |  |  |  |
|  | CNGA2 |  |  |  |
|  | CRISP3 |  |  |  |
|  | DEFB4 |  |  |  |
|  | DHRS4-AS1 |  |  |  |
|  | FBXO47 |  |  |  |
|  | GABRG3 |  |  |  |
|  | GNAT1 |  |  |  |
|  | GTPBP2 |  |  |  |
|  | IRG1L |  |  |  |
|  | KIFC3 |  |  |  |
|  | LYZL4 |  |  |  |
|  | MIR137 |  |  |  |
|  | MIR215 |  |  |  |
|  | MIR224 |  |  |  |
|  | MRPS25 |  |  |  |
|  | MTRFR |  |  |  |
|  | NLRP1A |  |  |  |
|  | OR8B8 |  |  |  |
|  | PCDHGA2 |  |  |  |
|  | PHAF1 |  |  |  |
|  | POTEF |  |  |  |
|  | PTK2B |  |  |  |
|  | RTL9 |  |  |  |
|  | SH2D7 |  |  |  |
|  | SMIM30 |  |  |  |
|  | SNHG14 |  |  |  |
|  | SULT2A2 |  |  |  |
|  | THA1 |  |  |  |
|  | THAP8 |  |  |  |
|  | TMPRSS11F |  |  |  |
|  | TRIM58 |  |  |  |
|  | TUBB8 |  |  |  |
|  | TVP23C |  |  |  |
|  | YWHAZ |  |  |  |
|  | ZFP30 |  |  |  |
|  | ZFP423 |  |  |  |
|  | ZFP809 |  |  |  |
|  | ZNF542P |  |  |  |
|  | ZNF614 |  |  |  |
|  | ZNF670 |  |  |  |
|  | CHRM1 |  |  |  |
|  | DPYSL4 |  |  |  |
|  | GRB7 |  |  |  |
|  | GRIA2 |  |  |  |
|  | MID2 |  |  |  |
|  | ONECUT1 |  |  |  |
|  | OXT |  |  |  |
|  | PAK2 |  |  |  |
|  | PAQR9 |  |  |  |
|  | PRKCSH |  |  |  |
|  | TFR2 |  |  |  |
|  | TOMM70A |  |  |  |
|  | BAIAP2-DT |  |  |  |
|  | BTNL2 |  |  |  |
|  | BXDC2 |  |  |  |
|  | C17ORF49 |  |  |  |
|  | C2ORF72 |  |  |  |
|  | C8ORF34 |  |  |  |
|  | CHRNG |  |  |  |
|  | COX7B2 |  |  |  |
|  | DNAJC3 |  |  |  |
|  | FAM135B |  |  |  |
|  | GPR82 |  |  |  |
|  | H1F3 |  |  |  |
|  | IGSF23 |  |  |  |
|  | KCNU1 |  |  |  |
|  | LILRA2 |  |  |  |
|  | LINC01554 |  |  |  |
|  | LRRC38 |  |  |  |
|  | LRRC57 |  |  |  |
|  | LRRC74B |  |  |  |
|  | MIR28 |  |  |  |
|  | MIR361 |  |  |  |
|  | MRPL44 |  |  |  |
|  | MT-RNR2 |  |  |  |
|  | NBPF3 |  |  |  |
|  | NEUROD6 |  |  |  |
|  | NKAPD1 |  |  |  |
|  | OCA2 |  |  |  |
|  | PCDHB8 |  |  |  |
|  | PCDHGA7 |  |  |  |
|  | PRL2C2 |  |  |  |
|  | RABL2 |  |  |  |
|  | REG4 |  |  |  |
|  | RITA1 |  |  |  |
|  | RNASEH2A |  |  |  |
|  | RNPEP |  |  |  |
|  | SLITRK4 |  |  |  |
|  | SLURP1 |  |  |  |
|  | USP6 |  |  |  |
|  | ZNF138 |  |  |  |
|  | ZNF202 |  |  |  |
|  | ZNF227 |  |  |  |
|  | ZNF43 |  |  |  |
|  | ZNF567 |  |  |  |
|  | ZNF738 |  |  |  |
|  | ZNF821 |  |  |  |
|  | ZSWIM7 |  |  |  |
|  | ACBD3 |  |  |  |
|  | ASXL3 |  |  |  |
|  | CBLN2 |  |  |  |
|  | FIS1 |  |  |  |
|  | GSTP2 |  |  |  |
|  | IGKC |  |  |  |
|  | KCNMB2 |  |  |  |
|  | LYRM1 |  |  |  |
|  | RAMP2 |  |  |  |
|  | TIMM17B |  |  |  |
|  | ARHGAP5-AS1 |  |  |  |
|  | ATP4B |  |  |  |
|  | C6ORF136 |  |  |  |
|  | CCDC83 |  |  |  |
|  | DRD3 |  |  |  |
|  | EIF4A1A |  |  |  |
|  | KIF12 |  |  |  |
|  | KRTAP2-3 |  |  |  |
|  | LCN15 |  |  |  |
|  | NAALADL2 |  |  |  |
|  | PABPC3 |  |  |  |
|  | SMLR1 |  |  |  |
|  | SNHG4 |  |  |  |
|  | SUSD5 |  |  |  |
|  | TADA2B |  |  |  |
|  | TEX36 |  |  |  |
|  | TIGD6 |  |  |  |
|  | TRAPPC2B |  |  |  |
|  | TYK2 |  |  |  |
|  | ZNF222 |  |  |  |
|  | ZNF268 |  |  |  |
|  | ZNF280B |  |  |  |
|  | ZNF419 |  |  |  |
|  | ZNF443 |  |  |  |
|  | ZNF529 |  |  |  |
|  | ZNF555 |  |  |  |
|  | ZNF639 |  |  |  |
|  | ZNF7 |  |  |  |
|  | ZNF76 |  |  |  |
|  | ANKRD31 |  |  |  |
|  | ARID3C |  |  |  |
|  | ASPDH |  |  |  |
|  | C1ORF131 |  |  |  |
|  | CFAP73 |  |  |  |
|  | CMKLR2 |  |  |  |
|  | CYP3A43 |  |  |  |
|  | DNAAF9 |  |  |  |
|  | DNM3OS |  |  |  |
|  | HEBP2 |  |  |  |
|  | IL17D |  |  |  |
|  | INSYN2B |  |  |  |
|  | LRP2BP |  |  |  |
|  | MIR148B |  |  |  |
|  | MIR381 |  |  |  |
|  | MIRLET7G |  |  |  |
|  | MTNAP1 |  |  |  |
|  | NEXMIF |  |  |  |
|  | ODF1 |  |  |  |
|  | PCDHGB6 |  |  |  |
|  | PHF11D |  |  |  |
|  | RTL3 |  |  |  |
|  | SLC22A25 |  |  |  |
|  | TMEM215 |  |  |  |
|  | ZNF236 |  |  |  |
|  | ZNF337 |  |  |  |
|  | ZNF550 |  |  |  |
|  | ZNF680 |  |  |  |
|  | ZNF77 |  |  |  |
|  | ZNF844 |  |  |  |
|  | ABO |  |  |  |
|  | AFG3L1P |  |  |  |
|  | AMDHD2 |  |  |  |
|  | ANKRD45 |  |  |  |
|  | ANXA2P3 |  |  |  |
|  | C11ORF71 |  |  |  |
|  | CAR4 |  |  |  |
|  | CNTNAP5B |  |  |  |
|  | GOLGA2P5 |  |  |  |
|  | MAPK8IP2 |  |  |  |
|  | PTAR1 |  |  |  |
|  | RPS6KA6 |  |  |  |
|  | SELENOM |  |  |  |
|  | ZNF358 |  |  |  |
|  | ZSCAN32 |  |  |  |
|  | ARRB2 |  |  |  |
|  | ATG4B |  |  |  |
|  | BCKDHA |  |  |  |
|  | C2ORF42 |  |  |  |
|  | C2ORF69 |  |  |  |
|  | CARD14 |  |  |  |
|  | CLXN |  |  |  |
|  | CMTM1 |  |  |  |
|  | FCRL6 |  |  |  |
|  | FPR3 |  |  |  |
|  | GPRC6A |  |  |  |
|  | H1-6 |  |  |  |
|  | LCE1C |  |  |  |
|  | LY6C |  |  |  |
|  | MAEL |  |  |  |
|  | MIR24-1 |  |  |  |
|  | MT-RNR1 |  |  |  |
|  | NF1 |  |  |  |
|  | NPBWR1 |  |  |  |
|  | OR2W3 |  |  |  |
|  | PITPNA |  |  |  |
|  | RAD17 |  |  |  |
|  | SERPINI2 |  |  |  |
|  | TIGD4 |  |  |  |
|  | TIGD7 |  |  |  |
|  | TMEM273 |  |  |  |
|  | ZNF14 |  |  |  |
|  | ZNF280A |  |  |  |
|  | ZNF334 |  |  |  |
|  | ZNF623 |  |  |  |
|  | ZNF658 |  |  |  |
|  | ABCA9 |  |  |  |
|  | CYP2A4 |  |  |  |
|  | MYORG |  |  |  |
|  | RINL |  |  |  |
|  | RTL5 |  |  |  |
|  | USF3 |  |  |  |
|  | ZKSCAN8 |  |  |  |
|  | ACSL6 |  |  |  |
|  | ADAD1 |  |  |  |
|  | AKAP4 |  |  |  |
|  | BCL6B |  |  |  |
|  | C1ORF162 |  |  |  |
|  | C1ORF174 |  |  |  |
|  | C2ORF76 |  |  |  |
|  | C6ORF89 |  |  |  |
|  | CCDC198 |  |  |  |
|  | CEP126 |  |  |  |
|  | CES5A |  |  |  |
|  | CHIL4 |  |  |  |
|  | CIDECP1 |  |  |  |
|  | CLDND2 |  |  |  |
|  | CSN2 |  |  |  |
|  | DMWD |  |  |  |
|  | EIF4E2 |  |  |  |
|  | EZH1 |  |  |  |
|  | FAM81B |  |  |  |
|  | GPR25 |  |  |  |
|  | GPR32 |  |  |  |
|  | LAIR2 |  |  |  |
|  | LYG1 |  |  |  |
|  | MIR424 |  |  |  |
|  | NKX6-3 |  |  |  |
|  | PANX3 |  |  |  |
|  | PCDHGA12 |  |  |  |
|  | POU5F1B |  |  |  |
|  | PSMB11 |  |  |  |
|  | PSMG3-AS1 |  |  |  |
|  | SEPN1 |  |  |  |
|  | SRSF8 |  |  |  |
|  | TMEM253 |  |  |  |
|  | WASH5P |  |  |  |
|  | ZBED6 |  |  |  |
|  | ZBTB37 |  |  |  |
|  | ZFP472 |  |  |  |
|  | ZNF441 |  |  |  |
|  | ZSWIM9 |  |  |  |
|  | CYP3A62 |  |  |  |
|  | DKKL1 |  |  |  |
|  | NELFE |  |  |  |
|  | NPHP4 |  |  |  |
|  | PHETA1 |  |  |  |
|  | RAPGEF5 |  |  |  |
|  | ZNF117 |  |  |  |
|  | ZSCAN20 |  |  |  |
|  | ACP4 |  |  |  |
|  | ARL17A |  |  |  |
|  | C12ORF76 |  |  |  |
|  | CIMIP2B |  |  |  |
|  | GUSBP4 |  |  |  |
|  | H2BC12L |  |  |  |
|  | IGHD |  |  |  |
|  | LCE1E |  |  |  |
|  | LINC01000 |  |  |  |
|  | METTL2A |  |  |  |
|  | MIR452 |  |  |  |
|  | MIR582 |  |  |  |
|  | MUC3A |  |  |  |
|  | NBPF14 |  |  |  |
|  | OIP5-AS1 |  |  |  |
|  | PPIAL4A |  |  |  |
|  | PVRIG |  |  |  |
|  | RCVRN |  |  |  |
|  | RPS2P32 |  |  |  |
|  | SLC25A15 |  |  |  |
|  | SUN3 |  |  |  |
|  | SUPT7L |  |  |  |
|  | TRPC6 |  |  |  |
|  | UGT2A3 |  |  |  |
|  | WBP2NL |  |  |  |
|  | ZNF184 |  |  |  |
|  | ZNF2 |  |  |  |
|  | ZNF304 |  |  |  |
|  | ZNF506 |  |  |  |
|  | ZNF526 |  |  |  |
|  | ZNF684 |  |  |  |
|  | ARHGAP11B |  |  |  |
|  | BRSK1 |  |  |  |
|  | CBY3 |  |  |  |
|  | CD1C |  |  |  |
|  | CFAP92 |  |  |  |
|  | CNTNAP3B |  |  |  |
|  | FAM124B |  |  |  |
|  | GJD4 |  |  |  |
|  | GSEC |  |  |  |
|  | IFNA4 |  |  |  |
|  | INSL4 |  |  |  |
|  | KRT79 |  |  |  |
|  | LBHD2 |  |  |  |
|  | LINC00520 |  |  |  |
|  | MAT2B |  |  |  |
|  | MCUR1 |  |  |  |
|  | MLNR |  |  |  |
|  | MRFAP1L1 |  |  |  |
|  | NEDD4 |  |  |  |
|  | NKX2-8 |  |  |  |
|  | PIK3C2G |  |  |  |
|  | PP2D1 |  |  |  |
|  | PPIL6 |  |  |  |
|  | QRFP |  |  |  |
|  | RAB4A |  |  |  |
|  | RHO |  |  |  |
|  | SARAF |  |  |  |
|  | SNORD89 |  |  |  |
|  | SPAG11B |  |  |  |
|  | STAC3 |  |  |  |
|  | AOX3 |  |  |  |
|  | BPIFB6 |  |  |  |
|  | BSCL2 |  |  |  |
|  | CACNA1G |  |  |  |
|  | FH1 |  |  |  |
|  | G6PC3 |  |  |  |
|  | GABBR1 |  |  |  |
|  | GDE1 |  |  |  |
|  | LCE1B |  |  |  |
|  | MIR501 |  |  |  |
|  | MUG1 |  |  |  |
|  | NRXN1 |  |  |  |
|  | PLCG2 |  |  |  |
|  | POMZP3 |  |  |  |
|  | RTL10 |  |  |  |
|  | RXFP2 |  |  |  |
|  | RYR2 |  |  |  |
|  | SNHG10 |  |  |  |
|  | TXNDC16 |  |  |  |
|  | ZNF107 |  |  |  |
|  | ZNF302 |  |  |  |
|  | ZNF350 |  |  |  |
|  | ZNF596 |  |  |  |
|  | ZNF675 |  |  |  |
|  | CST5 |  |  |  |
|  | DIS3 |  |  |  |
|  | DNAJB11 |  |  |  |
|  | GALNS |  |  |  |
|  | GUSBP2 |  |  |  |
|  | JMJD7-PLA2G4B |  |  |  |
|  | LAMTOR2 |  |  |  |
|  | RDH16 |  |  |  |
|  | SCAND2P |  |  |  |
|  | SEPW1 |  |  |  |
|  | SLITRK2 |  |  |  |
|  | SNTG2 |  |  |  |
|  | STAG3L1 |  |  |  |
|  | TTLL2 |  |  |  |
|  | ZNF17 |  |  |  |
|  | ZNF271P |  |  |  |
|  | ZNF875 |  |  |  |
|  | ACSM2A |  |  |  |
|  | AKAP1 |  |  |  |
|  | ATP2A1 |  |  |  |
|  | CACNA1A |  |  |  |
|  | CALB1 |  |  |  |
|  | CEACAM4 |  |  |  |
|  | CPLX4 |  |  |  |
|  | FRMD1 |  |  |  |
|  | FRMD7 |  |  |  |
|  | GTDC1 |  |  |  |
|  | HEYL |  |  |  |
|  | HMSD |  |  |  |
|  | IFNL2 |  |  |  |
|  | KLRF1 |  |  |  |
|  | NLGN4X |  |  |  |
|  | OSER1-DT |  |  |  |
|  | OXCT2 |  |  |  |
|  | PIP5K1A |  |  |  |
|  | PTH |  |  |  |
|  | PTPN7 |  |  |  |
|  | RPA4 |  |  |  |
|  | SCAND3 |  |  |  |
|  | SHD |  |  |  |
|  | UBQLNL |  |  |  |
|  | ZNF253 |  |  |  |
|  | ZNF343 |  |  |  |
|  | ZNF420 |  |  |  |
|  | ZNF491 |  |  |  |
|  | ZNF764 |  |  |  |
|  | APOL4 |  |  |  |
|  | CACNA1H |  |  |  |
|  | FGFR1A |  |  |  |
|  | GUSBP11 |  |  |  |
|  | HNRNPCL1 |  |  |  |
|  | LINC00339 |  |  |  |
|  | NPIPB5 |  |  |  |
|  | PIP4K2B |  |  |  |
|  | PSG8 |  |  |  |
|  | RCOR1 |  |  |  |
|  | ZNF713 |  |  |  |
|  | AMZ2P1 |  |  |  |
|  | ANKRD7 |  |  |  |
|  | B9D1 |  |  |  |
|  | BNIP5 |  |  |  |
|  | C10ORF95 |  |  |  |
|  | C11ORF21 |  |  |  |
|  | C8ORF48 |  |  |  |
|  | CLRN3 |  |  |  |
|  | FAM49A |  |  |  |
|  | GNAT3 |  |  |  |
|  | GTPBP1 |  |  |  |
|  | GUSBP1 |  |  |  |
|  | IFNL1 |  |  |  |
|  | IL17REL |  |  |  |
|  | KIF25 |  |  |  |
|  | LYPLAL1 |  |  |  |
|  | LZTFL1 |  |  |  |
|  | MIR155HG |  |  |  |
|  | MRVI1 |  |  |  |
|  | NLGN4Y |  |  |  |
|  | NUTM1 |  |  |  |
|  | NXF7 |  |  |  |
|  | PRM2 |  |  |  |
|  | RAB19 |  |  |  |
|  | RN7SL1 |  |  |  |
|  | SLC5A4 |  |  |  |
|  | SNORA21 |  |  |  |
|  | SRGAP2C |  |  |  |
|  | ST8SIA2 |  |  |  |
|  | TCP10L |  |  |  |
|  | TMEM212 |  |  |  |
|  | TNNC1 |  |  |  |
|  | TOB1-AS1 |  |  |  |
|  | TTC28-AS1 |  |  |  |
|  | ZNF212 |  |  |  |
|  | ZNF35 |  |  |  |
|  | ZNF611 |  |  |  |
|  | ZNF649 |  |  |  |
|  | ZNF75A |  |  |  |
|  | ZNF766 |  |  |  |
|  | ANO1 |  |  |  |
|  | GOT1L1 |  |  |  |
|  | KIAA0040 |  |  |  |
|  | PAFAH1B1 |  |  |  |
|  | ACLYA |  |  |  |
|  | ACSM1 |  |  |  |
|  | ANKRD36C |  |  |  |
|  | C19ORF18 |  |  |  |
|  | CCDC144BP |  |  |  |
|  | CENPBD1P |  |  |  |
|  | CSNK1A1 |  |  |  |
|  | CYP3A2 |  |  |  |
|  | EXOSC9 |  |  |  |
|  | FXR2 |  |  |  |
|  | GAST |  |  |  |
|  | GOLGA8IP |  |  |  |
|  | GPR39 |  |  |  |
|  | HAPLN1A |  |  |  |
|  | ICN |  |  |  |
|  | LINC00597 |  |  |  |
|  | LINC01140 |  |  |  |
|  | MYEOV2 |  |  |  |
|  | NBPF11 |  |  |  |
|  | P2RY2 |  |  |  |
|  | PMS2P5 |  |  |  |
|  | RAB43 |  |  |  |
|  | RDH10B |  |  |  |
|  | RGPD6 |  |  |  |
|  | SNAI3-AS1 |  |  |  |
|  | SULT5A1 |  |  |  |
|  | WDFY3-AS2 |  |  |  |
|  | ADGRF2 |  |  |  |
|  | C16ORF54 |  |  |  |
|  | CCL14 |  |  |  |
|  | CCL3L3 |  |  |  |
|  | CDK11A |  |  |  |
|  | CEBPA-DT |  |  |  |
|  | CLDN15 |  |  |  |
|  | CYP2C37 |  |  |  |
|  | DCAF4L1 |  |  |  |
|  | DEFB2 |  |  |  |
|  | GRASP |  |  |  |
|  | GZMH |  |  |  |
|  | HSPE1-MOB4 |  |  |  |
|  | KIAA1958 |  |  |  |
|  | KIR3DL2 |  |  |  |
|  | MIR33A |  |  |  |
|  | MIR431 |  |  |  |
|  | NBPF19 |  |  |  |
|  | NHLRC4 |  |  |  |
|  | PCDH11Y |  |  |  |
|  | PHLPP |  |  |  |
|  | PSG6 |  |  |  |
|  | SERF1A |  |  |  |
|  | SMIM38 |  |  |  |
|  | SSTR5-AS1 |  |  |  |
|  | THAP10 |  |  |  |
|  | ZNF536 |  |  |  |
|  | ZNF776 |  |  |  |
|  | ADCYAP1 |  |  |  |
|  | BTBD16 |  |  |  |
|  | DPM1 |  |  |  |
|  | GGPS1 |  |  |  |
|  | GTF2H3 |  |  |  |
|  | NAALAD2 |  |  |  |
|  | PGAP4 |  |  |  |
|  | RNASE3 |  |  |  |
|  | ZDHHC15 |  |  |  |
|  | C15ORF61 |  |  |  |
|  | C8ORF82 |  |  |  |
|  | C9ORF50 |  |  |  |
|  | DGCR6L |  |  |  |
|  | DTX3 |  |  |  |
|  | FOXN3-AS1 |  |  |  |
|  | GDF11 |  |  |  |
|  | GJA1B |  |  |  |
|  | HSPB9 |  |  |  |
|  | IGFBP5B |  |  |  |
|  | LINC00265 |  |  |  |
|  | LINC03040 |  |  |  |
|  | MIR652 |  |  |  |
|  | PABIR3 |  |  |  |
|  | PDXDC2P |  |  |  |
|  | PRKACG |  |  |  |
|  | PTTG3P |  |  |  |
|  | RAD51-AS1 |  |  |  |
|  | SCARNA2 |  |  |  |
|  | SLC16A12B |  |  |  |
|  | SMIM10L1 |  |  |  |
|  | SNORA74A |  |  |  |
|  | STAG3L2 |  |  |  |
|  | STAM |  |  |  |
|  | TASL |  |  |  |
|  | TMPRSS11D |  |  |  |
|  | TTC23 |  |  |  |
|  | TTTY14 |  |  |  |
|  | ZNF112 |  |  |  |
|  | ZNF134 |  |  |  |
|  | ZNF329 |  |  |  |
|  | CDC42SE2 |  |  |  |
|  | EPCAM |  |  |  |
|  | GLRX3 |  |  |  |
|  | ITIH2 |  |  |  |
|  | PABIR2 |  |  |  |
|  | S100G |  |  |  |
|  | TMEM116 |  |  |  |
|  | TNF |  |  |  |
|  | ATP2B1-AS1 |  |  |  |
|  | FGFBP2 |  |  |  |
|  | FRG2 |  |  |  |
|  | FURIN |  |  |  |
|  | GPC2 |  |  |  |
|  | HRH3 |  |  |  |
|  | IGK |  |  |  |
|  | LINC00511 |  |  |  |
|  | MIR181A2HG |  |  |  |
|  | PAQR7 |  |  |  |
|  | PRECSIT |  |  |  |
|  | RGPD1 |  |  |  |
|  | SPATA45 |  |  |  |
|  | ST7-AS1 |  |  |  |
|  | THEM7 |  |  |  |
|  | TUBA3FP |  |  |  |
|  | TWISTNB |  |  |  |
|  | UBE2D2A |  |  |  |
|  | ACP5A |  |  |  |
|  | AGPAT4-IT1 |  |  |  |
|  | ANKRD36BP1 |  |  |  |
|  | APOF |  |  |  |
|  | BICD2 |  |  |  |
|  | C18ORF32 |  |  |  |
|  | C8ORF44 |  |  |  |
|  | CASP3 |  |  |  |
|  | CROCCP2 |  |  |  |
|  | DIO3OS |  |  |  |
|  | FUT8-AS1 |  |  |  |
|  | HMGB1P1 |  |  |  |
|  | HOXB-AS3 |  |  |  |
|  | KIR3DL1 |  |  |  |
|  | LARS1B |  |  |  |
|  | LDAH |  |  |  |
|  | LRRC37A2 |  |  |  |
|  | LYPD6 |  |  |  |
|  | MORC2-AS1 |  |  |  |
|  | MTHFS |  |  |  |
|  | MTMR9LP |  |  |  |
|  | N6AMT2 |  |  |  |
|  | NPRL3 |  |  |  |
|  | NSUN5P1 |  |  |  |
|  | OGT.1 |  |  |  |
|  | PPIEL |  |  |  |
|  | PRKCG |  |  |  |
|  | RAB35 |  |  |  |
|  | RGS8 |  |  |  |
|  | RNASE7 |  |  |  |
|  | RPSAP52 |  |  |  |
|  | RUSC1-AS1 |  |  |  |
|  | SEC14L4 |  |  |  |
|  | SLC52A1 |  |  |  |
|  | SMPD5 |  |  |  |
|  | SYN2 |  |  |  |
|  | TAS2R31 |  |  |  |
|  | TRPC3 |  |  |  |
|  | TSPEAR-AS2 |  |  |  |
|  | TTL |  |  |  |
|  | VPS24 |  |  |  |
|  | WFDC5 |  |  |  |
|  | ZNF322 |  |  |  |
|  | ZNF700 |  |  |  |
|  | ZUFSP |  |  |  |
|  | C4ORF3 |  |  |  |
|  | DHX29 |  |  |  |
|  | LCTL |  |  |  |
|  | PLA2G2D |  |  |  |
|  | SOHLH2 |  |  |  |
|  | SPTLC1 |  |  |  |
|  | VIP |  |  |  |
|  | AATBC |  |  |  |
|  | ADAM18 |  |  |  |
|  | ARRDC5 |  |  |  |
|  | ARXES2 |  |  |  |
|  | C12ORF56 |  |  |  |
|  | C6ORF58 |  |  |  |
|  | CASR |  |  |  |
|  | CCDC153 |  |  |  |
|  | DNAJC5B |  |  |  |
|  | EXO5 |  |  |  |
|  | FXYD7 |  |  |  |
|  | GADD45 |  |  |  |
|  | GVINP1 |  |  |  |
|  | HEATR9 |  |  |  |
|  | HHLA2 |  |  |  |
|  | HYMAI |  |  |  |
|  | IGHG3 |  |  |  |
|  | KCNJ5-AS1 |  |  |  |
|  | LINC01619 |  |  |  |
|  | MAGEA4 |  |  |  |
|  | MAGEB1 |  |  |  |
|  | MEP1B |  |  |  |
|  | MGAT4D |  |  |  |
|  | MIR26A |  |  |  |
|  | RTP2 |  |  |  |
|  | SIGLEC9 |  |  |  |
|  | SLC7A11-AS1 |  |  |  |
|  | SLFN12L |  |  |  |
|  | SNORA14B |  |  |  |
|  | SNORA64 |  |  |  |
|  | SSX1 |  |  |  |
|  | STARD4-AS1 |  |  |  |
|  | UNC5B-AS1 |  |  |  |
|  | ZNF177 |  |  |  |
|  | ZNF230 |  |  |  |
|  | ZSCAN4 |  |  |  |
|  | CERS2 |  |  |  |
|  | HEMK1 |  |  |  |
|  | MCU |  |  |  |
|  | RTCA |  |  |  |
|  | SLC12A5 |  |  |  |
|  | ADIG |  |  |  |
|  | ANXA5B |  |  |  |
|  | ASMTL-AS1 |  |  |  |
|  | C11ORF24 |  |  |  |
|  | COX19 |  |  |  |
|  | EPHA2A |  |  |  |
|  | FOSL1A |  |  |  |
|  | GOLGA6L9 |  |  |  |
|  | HERC2P2 |  |  |  |
|  | NEU3 |  |  |  |
|  | PKD2 |  |  |  |
|  | PLEKHG7 |  |  |  |
|  | RPL23AP32 |  |  |  |
|  | SLCO1A6 |  |  |  |
|  | SPANXA1 |  |  |  |
|  | SULT2ST2 |  |  |  |
|  | TERB1 |  |  |  |
|  | UBE2Q2P1 |  |  |  |
|  | ZNF681 |  |  |  |
|  | ZP2.3 |  |  |  |
|  | CST4 |  |  |  |
|  | DIRC3 |  |  |  |
|  | H4C14 |  |  |  |
|  | KRTAP2-4 |  |  |  |
|  | LYPLA1 |  |  |  |
|  | MIR374A |  |  |  |
|  | NT5DC4 |  |  |  |
|  | OR51B4 |  |  |  |
|  | PPP3R2 |  |  |  |
|  | RGPD5 |  |  |  |
|  | ST20 |  |  |  |
|  | ZNF182 |  |  |  |
|  | ZNF20 |  |  |  |
|  | ZNF571 |  |  |  |
|  | ZNF786 |  |  |  |
|  | ADCK5 |  |  |  |
|  | ATP5S |  |  |  |
|  | BTBD2 |  |  |  |
|  | C22ORF42 |  |  |  |
|  | CCDC185 |  |  |  |
|  | CCDC27 |  |  |  |
|  | CD74A |  |  |  |
|  | CRLF3 |  |  |  |
|  | EEF1A1P5 |  |  |  |
|  | EEF2KMT |  |  |  |
|  | GLB1L |  |  |  |
|  | IL12 |  |  |  |
|  | KIR2DL3 |  |  |  |
|  | KLK1 |  |  |  |
|  | LAMB4 |  |  |  |
|  | LINC02593 |  |  |  |
|  | MFSD10 |  |  |  |
|  | MS4A10 |  |  |  |
|  | NEXN-AS1 |  |  |  |
|  | PPDPFL |  |  |  |
|  | PRELID3B |  |  |  |
|  | RAPGEF3 |  |  |  |
|  | RHNO1 |  |  |  |
|  | RNF183 |  |  |  |
|  | RPL17-C18ORF32 |  |  |  |
|  | RPP38-DT |  |  |  |
|  | SCT |  |  |  |
|  | SNORD17 |  |  |  |
|  | SYT13 |  |  |  |
|  | TLR21 |  |  |  |
|  | TRPM2 |  |  |  |
|  | WT1-AS |  |  |  |
|  | B3GALT5-AS1 |  |  |  |
|  | B3GNT4 |  |  |  |
|  | BLOC1S6 |  |  |  |
|  | CACNA1I |  |  |  |
|  | CUEDC2 |  |  |  |
|  | CYP21A1 |  |  |  |
|  | CYP2A7 |  |  |  |
|  | EXOSC10 |  |  |  |
|  | FXYD2 |  |  |  |
|  | GAD1B |  |  |  |
|  | GRK6 |  |  |  |
|  | IGFBP1A |  |  |  |
|  | KRBOX5 |  |  |  |
|  | MRPS36 |  |  |  |
|  | OR10J5 |  |  |  |
|  | PASK |  |  |  |
|  | PHB |  |  |  |
|  | PITPNAA |  |  |  |
|  | S100A13 |  |  |  |
|  | SLC6A20 |  |  |  |
|  | SMEK1 |  |  |  |
|  | STMN1A |  |  |  |
|  | TBC1D21 |  |  |  |
|  | TTC1 |  |  |  |
|  | USP30-AS1 |  |  |  |
|  | UST4R |  |  |  |
|  | ZRSR2P1 |  |  |  |
|  | C7ORF25 |  |  |  |
|  | CBFB |  |  |  |
|  | CYP2C38 |  |  |  |
|  | EDN2 |  |  |  |
|  | FSIP2 |  |  |  |
|  | GARIN2 |  |  |  |
|  | GPR137C |  |  |  |
|  | HMGCRA |  |  |  |
|  | HSPB3 |  |  |  |
|  | JUB |  |  |  |
|  | KRTAP19-1 |  |  |  |
|  | LGALS9C |  |  |  |
|  | MIR181B1 |  |  |  |
|  | MIR450A1 |  |  |  |
|  | PKD1P1 |  |  |  |
|  | PRSS16 |  |  |  |
|  | SCGB1A1 |  |  |  |
|  | SNORA28 |  |  |  |
|  | SPRR4 |  |  |  |
|  | LINGO4 |  |  |  |
|  | RYR3 |  |  |  |
|  | SLC25A17 |  |  |  |
|  | TMEM231 |  |  |  |
|  | ZNF175 |  |  |  |
|  | ABCC12 |  |  |  |
|  | ADORA2A-AS1 |  |  |  |
|  | ASIC1 |  |  |  |
|  | C5ORF58 |  |  |  |
|  | CEACAM21 |  |  |  |
|  | CFLAR-AS1 |  |  |  |
|  | CYP4F18 |  |  |  |
|  | DDX59-AS1 |  |  |  |
|  | F8A3 |  |  |  |
|  | FAM177B |  |  |  |
|  | HLA-J |  |  |  |
|  | JMJD1C-AS1 |  |  |  |
|  | KRT17P3 |  |  |  |
|  | LINC01270 |  |  |  |
|  | MIR219A1 |  |  |  |
|  | MIR622 |  |  |  |
|  | MIR942 |  |  |  |
|  | MROH9 |  |  |  |
|  | PEAK3 |  |  |  |
|  | RNU6ATAC |  |  |  |
|  | RPL13AP5 |  |  |  |
|  | SHISAL2A |  |  |  |
|  | SNORD15A |  |  |  |
|  | TEX41 |  |  |  |
|  | TLR2A |  |  |  |
|  | TREML3P |  |  |  |
|  | TRNS1 |  |  |  |
|  | ANP32A-IT1 |  |  |  |
|  | AVPR1B |  |  |  |
|  | BRD2A |  |  |  |
|  | CFAP107 |  |  |  |
|  | CIMIP2A |  |  |  |
|  | CXCR5 |  |  |  |
|  | EGFRA |  |  |  |
|  | FAM170B |  |  |  |
|  | FBXW12 |  |  |  |
|  | FOXB2 |  |  |  |
|  | IL6 |  |  |  |
|  | KRT18P55 |  |  |  |
|  | LINC00965 |  |  |  |
|  | MFSD11 |  |  |  |
|  | OSTN |  |  |  |
|  | PABPC1A |  |  |  |
|  | PFN1P2 |  |  |  |
|  | POM121L8P |  |  |  |
|  | PRKCBB |  |  |  |
|  | SDCBP2-AS1 |  |  |  |
|  | SDR9C7 |  |  |  |
|  | TEKT4P2 |  |  |  |
|  | TGTP2 |  |  |  |
|  | UBE2NL |  |  |  |
|  | VSTM1 |  |  |  |
|  | ZNF709 |  |  |  |
|  | CERK |  |  |  |
|  | CYP26C1 |  |  |  |
|  | CYP3A44 |  |  |  |
|  | FEZF2 |  |  |  |
|  | TXN1 |  |  |  |
|  | UBTF |  |  |  |
|  | ACSF3 |  |  |  |
|  | ALG2 |  |  |  |
|  | CACNA1B |  |  |  |
|  | CACNA2D2 |  |  |  |
|  | CAV3 |  |  |  |
|  | GATA1 |  |  |  |
|  | IL1B |  |  |  |
|  | SHMT2 |  |  |  |
|  | SLC22A10 |  |  |  |
|  | SLC26A5 |  |  |  |
|  | TRPV3 |  |  |  |
|  | AFAP1-AS1 |  |  |  |
|  | APS |  |  |  |
|  | C12ORF50 |  |  |  |
|  | CACNA1E |  |  |  |
|  | CFAP74 |  |  |  |
|  | CXCL8B.1 |  |  |  |
|  | CYP2C39 |  |  |  |
|  | CYP4F14 |  |  |  |
|  | DEFB124 |  |  |  |
|  | DIRC1 |  |  |  |
|  | DUPD1 |  |  |  |
|  | FCGR2C |  |  |  |
|  | GBP1P1 |  |  |  |
|  | GHET1 |  |  |  |
|  | GNA11 |  |  |  |
|  | HHIP-AS1 |  |  |  |
|  | HLA-DQA2 |  |  |  |
|  | IGSF22 |  |  |  |
|  | KEG1 |  |  |  |
|  | KIR3DX1 |  |  |  |
|  | KRT16P2 |  |  |  |
|  | KRTAP5-AS1 |  |  |  |
|  | LINC00299 |  |  |  |
|  | LINC00477 |  |  |  |
|  | LINC01094 |  |  |  |
|  | LINC01132 |  |  |  |
|  | LINC02870 |  |  |  |
|  | LINC02910 |  |  |  |
|  | LNCOG |  |  |  |
|  | MAATS1 |  |  |  |
|  | MIR146 |  |  |  |
|  | NUDT9P1 |  |  |  |
|  | PICART1 |  |  |  |
|  | RNF212B |  |  |  |
|  | SIGLEC16 |  |  |  |
|  | SMIM10 |  |  |  |
|  | SMIM32 |  |  |  |
|  | SNORA20 |  |  |  |
|  | SNORA54 |  |  |  |
|  | SPATA41 |  |  |  |
|  | TARS1 |  |  |  |
|  | TRGV9 |  |  |  |
|  | ZIC5 |  |  |  |
|  | FAM106A |  |  |  |
|  | KCNE1 |  |  |  |
|  | MIR454 |  |  |  |
|  | PROK2 |  |  |  |
|  | SMAD5-AS1 |  |  |  |
|  | SPDYE1 |  |  |  |
|  | ZNF283 |  |  |  |
|  | ARL17B |  |  |  |
|  | CAPRIN1B |  |  |  |
|  | EMC3-AS1 |  |  |  |
|  | GCSHB |  |  |  |
|  | HARS |  |  |  |
|  | HOXA3A |  |  |  |
|  | IFITM4P |  |  |  |
|  | IGLL3P |  |  |  |
|  | LRRC4B |  |  |  |
|  | MVDA |  |  |  |
|  | PDE6A |  |  |  |
|  | PRINS |  |  |  |
|  | RBM25B |  |  |  |
|  | SPEM2 |  |  |  |
|  | SUGT1 |  |  |  |
|  | THAP7-AS1 |  |  |  |
|  | TNNT2A |  |  |  |
|  | TRMT44 |  |  |  |
|  | ZNF157 |  |  |  |
|  | CA4 |  |  |  |
|  | CYP2J9 |  |  |  |
|  | IGFBP1B |  |  |  |
|  | PLA2G1B |  |  |  |
|  | BCL2 |  |  |  |
|  | MAPK1 |  |  |  |
|  | RT1-M3-1 |  |  |  |
|  | ZCWPW2 |  |  |  |
|  | AKR1C8 |  |  |  |
|  | ARHGEF26-AS1 |  |  |  |
|  | C10ORF67 |  |  |  |
|  | C1ORF127 |  |  |  |
|  | CCDC194 |  |  |  |
|  | COMMD3-BMI1 |  |  |  |
|  | CSNK1G3 |  |  |  |
|  | EGOT |  |  |  |
|  | ETV3L |  |  |  |
|  | FAM225A |  |  |  |
|  | FAM95C |  |  |  |
|  | FOXP4-AS1 |  |  |  |
|  | GYPE |  |  |  |
|  | HRC |  |  |  |
|  | KLK8 |  |  |  |
|  | LINC00899 |  |  |  |
|  | LINC00900 |  |  |  |
|  | LRMP |  |  |  |
|  | MAPK3 |  |  |  |
|  | MBL1P |  |  |  |
|  | MEIOSIN |  |  |  |
|  | MFSD4B |  |  |  |
|  | MIR3074 |  |  |  |
|  | MIR3911 |  |  |  |
|  | P2RX3 |  |  |  |
|  | PCDHGB3 |  |  |  |
|  | PRDM7 |  |  |  |
|  | PSA |  |  |  |
|  | PXT1 |  |  |  |
|  | RPL34-DT |  |  |  |
|  | SDHAP2 |  |  |  |
|  | SERPINB9P1 |  |  |  |
|  | SLC8A3 |  |  |  |
|  | SMIM2 |  |  |  |
|  | SNORD46 |  |  |  |
|  | SOD2-OT1 |  |  |  |
|  | SUGT1P1 |  |  |  |
|  | TAS2R38 |  |  |  |
|  | TEDDM1 |  |  |  |
|  | TMSB4XP8 |  |  |  |
|  | TOLLIP-DT |  |  |  |
|  | WASH3P |  |  |  |
|  | WEE2-AS1 |  |  |  |
|  | WHAMMP2 |  |  |  |
|  | ZKSCAN8P1 |  |  |  |
|  | ZNF295-AS1 |  |  |  |
|  | CCDC32 |  |  |  |
|  | MYL10 |  |  |  |
|  | RBP3 |  |  |  |
|  | AMY1C |  |  |  |
|  | BIRC5A |  |  |  |
|  | CELA2B |  |  |  |
|  | CLDND |  |  |  |
|  | CLDNI |  |  |  |
|  | CRABP1A |  |  |  |
|  | CRYBA1B |  |  |  |
|  | CRYBA1L1 |  |  |  |
|  | CRYGM4 |  |  |  |
|  | CRYGN2 |  |  |  |
|  | DHRS13A.1 |  |  |  |
|  | DIPK1C |  |  |  |
|  | DNAJC11A |  |  |  |
|  | DPY19L1P1 |  |  |  |
|  | ERBB3A |  |  |  |
|  | INHBAB |  |  |  |
|  | LORICRIN |  |  |  |
|  | NAIP |  |  |  |
|  | PCDH19 |  |  |  |
|  | PMS2P9 |  |  |  |
|  | RPL17P33 |  |  |  |
|  | SEC22BA |  |  |  |
|  | AHSG |  |  |  |
|  | AKAP11 |  |  |  |
|  | BAX |  |  |  |
|  | CFAP276 |  |  |  |
|  | DNAJB7 |  |  |  |
|  | HPCA |  |  |  |
|  | RIDA |  |  |  |
|  | CEP20 |  |  |  |
|  | KRTAP2-1 |  |  |  |
|  | LINC00707 |  |  |  |
|  | MIR485 |  |  |  |
|  | MIR660 |  |  |  |
|  | NBEAP1 |  |  |  |
|  | RSPRY1 |  |  |  |
|  | SIRPG |  |  |  |
|  | SKIV2L2 |  |  |  |
|  | SNORD8 |  |  |  |
|  | ZNF565 |  |  |  |
|  | ABCA8B |  |  |  |
|  | CSTDC5 |  |  |  |
|  | DLEU2L |  |  |  |
|  | DOCK8-AS1 |  |  |  |
|  | H2-M5 |  |  |  |
|  | HCG4B |  |  |  |
|  | IFT88 |  |  |  |
|  | LHX1-DT |  |  |  |
|  | LINC00632 |  |  |  |
|  | LINC01121 |  |  |  |
|  | LINC02015 |  |  |  |
|  | LINC03122 |  |  |  |
|  | MIR34AHG |  |  |  |
|  | NOX2 |  |  |  |
|  | OR2AT4 |  |  |  |
|  | OR52K1 |  |  |  |
|  | PRR34 |  |  |  |
|  | RIMBP3C |  |  |  |
|  | RPL6P27 |  |  |  |
|  | SCARNA12 |  |  |  |
|  | SIGLEC11 |  |  |  |
|  | SNORD56 |  |  |  |
|  | SNORD59A |  |  |  |
|  | SNORD63 |  |  |  |
|  | SNORD87 |  |  |  |
|  | SPAAR |  |  |  |
|  | TRNC |  |  |  |
|  | TRNN |  |  |  |
|  | WASIR2 |  |  |  |
|  | ZNF300P1 |  |  |  |
|  | ZNF815P |  |  |  |
|  | ZNF890P |  |  |  |
|  | AVPR2 |  |  |  |
|  | CAT |  |  |  |
|  | CESL1 |  |  |  |
|  | CNNM4 |  |  |  |
|  | CYP4A12B |  |  |  |
|  | ERICH6 |  |  |  |
|  | GIP |  |  |  |
|  | GUCY1B3 |  |  |  |
|  | NGB |  |  |  |
|  | NPVF |  |  |  |
|  | PROSER3 |  |  |  |
|  | PVALB8 |  |  |  |
|  | TTLL10 |  |  |  |
|  | TUT1 |  |  |  |
|  | UGT3A2 |  |  |  |
|  | AGAP7P |  |  |  |
|  | AMOTL2B |  |  |  |
|  | ATF4B |  |  |  |
|  | ATP5MC3B |  |  |  |
|  | CRYGM5 |  |  |  |
|  | ENTPD2A.1 |  |  |  |
|  | EOMESA |  |  |  |
|  | EPHX5 |  |  |  |
|  | FDPSP2 |  |  |  |
|  | GOLGA6L5P |  |  |  |
|  | HIBADHB |  |  |  |
|  | INSRA |  |  |  |
|  | KDM5BA |  |  |  |
|  | LOH12CR2 |  |  |  |
|  | MED14OS |  |  |  |
|  | MYH11A |  |  |  |
|  | NDUFS8A |  |  |  |
|  | OR7E91P |  |  |  |
|  | PDCD4A |  |  |  |
|  | PTGES3B |  |  |  |
|  | RDH10A |  |  |  |
|  | SAP130A |  |  |  |
|  | SPON1B |  |  |  |
|  | CYLC1 |  |  |  |
|  | ERV3-2 |  |  |  |
|  | FAM218A |  |  |  |
|  | KEAP1A |  |  |  |
|  | LINC00941 |  |  |  |
|  | MAGI2-AS3 |  |  |  |
|  | MYL3 |  |  |  |
|  | NR2E1 |  |  |  |
|  | PTGS2 |  |  |  |
|  | TEX13A |  |  |  |
|  | TRPV5 |  |  |  |
|  | ATP2A3 |  |  |  |
|  | MAO |  |  |  |
|  | PFKFB4B |  |  |  |
|  | CYP2J5 |  |  |  |
|  | HMOX1 |  |  |  |
|  | RELA |  |  |  |
|  | C16ORF46 |  |  |  |
|  | C1GALT1C1L |  |  |  |
|  | CACNG2 |  |  |  |
|  | CASC19 |  |  |  |
|  | CD1B |  |  |  |
|  | CD1E |  |  |  |
|  | CIBAR1-DT |  |  |  |
|  | CLEC19A |  |  |  |
|  | CTSA |  |  |  |
|  | CXORF58 |  |  |  |
|  | CYP2K18 |  |  |  |
|  | CYP2T1P |  |  |  |
|  | DEFB36 |  |  |  |
|  | DHRS4L1 |  |  |  |
|  | DNAJC5G |  |  |  |
|  | EDDM3B |  |  |  |
|  | EMSLR |  |  |  |
|  | GIHCG |  |  |  |
|  | GLIS3-AS1 |  |  |  |
|  | GOLGA8O |  |  |  |
|  | H2BU1 |  |  |  |
|  | HMGN2P46 |  |  |  |
|  | LINC00964 |  |  |  |
|  | LY6B |  |  |  |
|  | LY86-AS1 |  |  |  |
|  | LYC2 |  |  |  |
|  | MIR124-1HG |  |  |  |
|  | MIR3142HG |  |  |  |
|  | MKNK1-AS1 |  |  |  |
|  | RBM12B-AS1 |  |  |  |
|  | SCOC-AS1 |  |  |  |
|  | SH3TC2-DT |  |  |  |
|  | SNORA72 |  |  |  |
|  | SNORD93 |  |  |  |
|  | SNRPGP2 |  |  |  |
|  | SPATA12 |  |  |  |
|  | SPATA31E1 |  |  |  |
|  | TBC1D29P |  |  |  |
|  | TRG-AS1 |  |  |  |
|  | TRNP |  |  |  |
|  | UMODL1-AS1 |  |  |  |
|  | VCX |  |  |  |
|  | CYP2D3 |  |  |  |
|  | GADD45BA |  |  |  |
|  | RPN2 |  |  |  |
|  | ATP5MEA |  |  |  |
|  | AZIN1B |  |  |  |
|  | C12ORF54 |  |  |  |
|  | C17ORF99 |  |  |  |
|  | C1ORF167 |  |  |  |
|  | CALD1B |  |  |  |
|  | CCDC192 |  |  |  |
|  | CPXCR1 |  |  |  |
|  | CYP2X9 |  |  |  |
|  | EFNA1A |  |  |  |
|  | EIF2S1A |  |  |  |
|  | EML2-AS1 |  |  |  |
|  | EPHB3A |  |  |  |
|  | FABP4A |  |  |  |
|  | GLRXP3 |  |  |  |
|  | HSPD1P6 |  |  |  |
|  | IFRG15 |  |  |  |
|  | KRCP |  |  |  |
|  | MARCKSL1A |  |  |  |
|  | MIR151A |  |  |  |
|  | MIR181A1 |  |  |  |
|  | NBEAA |  |  |  |
|  | NDRG3B |  |  |  |
|  | NDUFA9A |  |  |  |
|  | NDUFAB1A |  |  |  |
|  | NFIL3-6 |  |  |  |
|  | NR2E3 |  |  |  |
|  | OCLNA |  |  |  |
|  | OR7E13P |  |  |  |
|  | OTPA |  |  |  |
|  | PCMTL |  |  |  |
|  | RPL23AP7 |  |  |  |
|  | SH2D3CA |  |  |  |
|  | SNRPGP10 |  |  |  |
|  | SNX10A |  |  |  |
|  | TCIRG1B |  |  |  |
|  | TEX47 |  |  |  |
|  | TIMM23A |  |  |  |
|  | TRPC5 |  |  |  |
|  | UBALD1A |  |  |  |
|  | YWHAEP1 |  |  |  |
|  | ZNF826P |  |  |  |
|  | ABCC6P1 |  |  |  |
|  | ANG |  |  |  |
|  | CCR9 |  |  |  |
|  | MYH14 |  |  |  |
|  | OR4B1D |  |  |  |
|  | PICK1 |  |  |  |
|  | RAB7 |  |  |  |
|  | SLC22A22 |  |  |  |
|  | ABCA1A |  |  |  |
|  | ATP2A2B |  |  |  |
|  | DIO1 |  |  |  |
|  | HSD3B4 |  |  |  |
|  | KRT16 |  |  |  |
|  | NHERF4 |  |  |  |
|  | NOS2 |  |  |  |
|  | TXNDC5 |  |  |  |
|  | CASP9 |  |  |  |
|  | CPLANE2 |  |  |  |
|  | CYP4A22 |  |  |  |
|  | EIF4EBP3L |  |  |  |
|  | GADD45AA |  |  |  |
|  | ITGB1BP2 |  |  |  |
|  | P2RY4 |  |  |  |
|  | PDILT |  |  |  |
|  | SLC8A2 |  |  |  |
|  | SYN1 |  |  |  |
|  | ANKRD26P3 |  |  |  |
|  | C19ORF84 |  |  |  |
|  | CCL4L2 |  |  |  |
|  | CHURC1-FNTB |  |  |  |
|  | CSTDC4 |  |  |  |
|  | DDIT4-AS1 |  |  |  |
|  | FAM27B |  |  |  |
|  | GNL3LP1 |  |  |  |
|  | GTSF1 |  |  |  |
|  | IFNG1R |  |  |  |
|  | IGKV4-1 |  |  |  |
|  | IL1 |  |  |  |
|  | KRTAP2-2 |  |  |  |
|  | KRTAP4-7 |  |  |  |
|  | LINC00158 |  |  |  |
|  | LINC00240 |  |  |  |
|  | LINC00616 |  |  |  |
|  | LINC00659 |  |  |  |
|  | LINC00971 |  |  |  |
|  | LINC01093 |  |  |  |
|  | LINC01303 |  |  |  |
|  | LINC01589 |  |  |  |
|  | LINC02541 |  |  |  |
|  | LSP2 |  |  |  |
|  | MIR181A-1 |  |  |  |
|  | MIR3189 |  |  |  |
|  | MIRLET7A-1 |  |  |  |
|  | MROCKI |  |  |  |
|  | NFE4 |  |  |  |
|  | NR1I2 |  |  |  |
|  | P53 |  |  |  |
|  | SCAT8 |  |  |  |
|  | SNORD61 |  |  |  |
|  | TAF1A-AS1 |  |  |  |
|  | TBC1D27P |  |  |  |
|  | TENM3-AS1 |  |  |  |
|  | TRGV2 |  |  |  |
|  | TTC3P1 |  |  |  |
|  | TWF1 |  |  |  |
|  | USP9 |  |  |  |
|  | VTRNA1-1 |  |  |  |
|  | ZFP128 |  |  |  |
|  | ZNF534 |  |  |  |
|  | ZSWIM2 |  |  |  |
|  | ACBD5A |  |  |  |
|  | ADM2A |  |  |  |
|  | AKR1A1B |  |  |  |
|  | ANKRD36BP2 |  |  |  |
|  | AQP7P2 |  |  |  |
|  | ARPP19B |  |  |  |
|  | BIDA |  |  |  |
|  | CACNA1F |  |  |  |
|  | CLDN11A |  |  |  |
|  | CRABP2A |  |  |  |
|  | CYTH1A |  |  |  |
|  | DOK1B |  |  |  |
|  | EEF1A1P22 |  |  |  |
|  | EHD1B |  |  |  |
|  | FAUB |  |  |  |
|  | GNG12 |  |  |  |
|  | GOLGA8EP |  |  |  |
|  | HLA-DRB1 |  |  |  |
|  | KCNJ1A.1 |  |  |  |
|  | KCNMB1 |  |  |  |
|  | KLF5A |  |  |  |
|  | MAP1LC3BP1 |  |  |  |
|  | MARCKSB |  |  |  |
|  | MAT2AL |  |  |  |
|  | MICAL3B |  |  |  |
|  | MRPL42P5 |  |  |  |
|  | MYO1CB |  |  |  |
|  | NOA1 |  |  |  |
|  | NPIPB1P |  |  |  |
|  | PARP1 |  |  |  |
|  | PBXIP1B |  |  |  |
|  | PHB2A |  |  |  |
|  | PLEKHG5A |  |  |  |
|  | PPP1CC |  |  |  |
|  | PRELID1A |  |  |  |
|  | PTPN23A |  |  |  |
|  | RAP1AA |  |  |  |
|  | RNF128A |  |  |  |
|  | RORAA |  |  |  |
|  | RPSAP47 |  |  |  |
|  | SELENOJ |  |  |  |
|  | SELO |  |  |  |
|  | SMFN |  |  |  |
|  | SOWAHCB |  |  |  |
|  | SPDYE21 |  |  |  |
|  | SPSB3A |  |  |  |
|  | STMN1B |  |  |  |
|  | SUMO1P3 |  |  |  |
|  | SURF4L |  |  |  |
|  | SYT2A |  |  |  |
|  | TMT1A.1 |  |  |  |
|  | TNRC6C1 |  |  |  |
|  | TPRB |  |  |  |
|  | TUBA4L |  |  |  |
|  | ZNF731P |  |  |  |
|  | ATP2A2A |  |  |  |
|  | CCR8 |  |  |  |
|  | GJD2 |  |  |  |
|  | PMM2 |  |  |  |
|  | RALB |  |  |  |
|  | RBP2B |  |  |  |
|  | SELENOT |  |  |  |
|  | AKT2 |  |  |  |
|  | FAM126B |  |  |  |
|  | LELP1 |  |  |  |
|  | MCPT9 |  |  |  |
|  | POU6F1 |  |  |  |
|  | NFKBIAB |  |  |  |
|  | CCL2 |  |  |  |
|  | HNRNPH2 |  |  |  |
|  | ICAM2 |  |  |  |
|  | ANKRD1B |  |  |  |
|  | ATTA |  |  |  |
|  | BAGE |  |  |  |
|  | CLEC4C |  |  |  |
|  | CLUL1 |  |  |  |
|  | CTSL.1 |  |  |  |
|  | CYP2K19 |  |  |  |
|  | CYP4E2 |  |  |  |
|  | FABP1B.1 |  |  |  |
|  | GJA8 |  |  |  |
|  | KRTAP4-9 |  |  |  |
|  | LINC00474 |  |  |  |
|  | LINC01224 |  |  |  |
|  | LSP1BETA |  |  |  |
|  | MCL1B |  |  |  |
|  | MIR29B |  |  |  |
|  | MIR3472 |  |  |  |
|  | MTND |  |  |  |
|  | SNHG33 |  |  |  |
|  | ZFAND5A |  |  |  |
|  | ZNF578 |  |  |  |
|  | ANKRD20A1 |  |  |  |
|  | ARHB |  |  |  |
|  | BBOX1-AS1 |  |  |  |
|  | C5ORF67 |  |  |  |
|  | CASC2 |  |  |  |
|  | CKLF-CMTM1 |  |  |  |
|  | CSTA2 |  |  |  |
|  | DEFB24 |  |  |  |
|  | DLG5-AS1 |  |  |  |
|  | EEF1DP4 |  |  |  |
|  | FCGR1CP |  |  |  |
|  | HMGB1P5 |  |  |  |
|  | IL12BA |  |  |  |
|  | KLHL7-DT |  |  |  |
|  | LHFPL3-AS2 |  |  |  |
|  | LINC00426 |  |  |  |
|  | LINC01269 |  |  |  |
|  | LINC01410 |  |  |  |
|  | LINC01484 |  |  |  |
|  | LINC02159 |  |  |  |
|  | LINC02273 |  |  |  |
|  | LINC02340 |  |  |  |
|  | LINC02363 |  |  |  |
|  | LINC02561 |  |  |  |
|  | LINC02757 |  |  |  |
|  | LINC02908 |  |  |  |
|  | LINC02913 |  |  |  |
|  | LINC02915 |  |  |  |
|  | LRRC53 |  |  |  |
|  | LRRC77P |  |  |  |
|  | M17 |  |  |  |
|  | MAP2K4P1 |  |  |  |
|  | MIR3150BHG |  |  |  |
|  | MIR3916 |  |  |  |
|  | MIR5188 |  |  |  |
|  | MIR669N |  |  |  |
|  | MTND2P28 |  |  |  |
|  | NAMPTP1 |  |  |  |
|  | OR4C6 |  |  |  |
|  | OR7E47P |  |  |  |
|  | OR8G5 |  |  |  |
|  | OXCT2P1 |  |  |  |
|  | PPBPP2 |  |  |  |
|  | RARA-AS1 |  |  |  |
|  | RGPD2 |  |  |  |
|  | RIMBP3B |  |  |  |
|  | ROR1-AS1 |  |  |  |
|  | SIGLECL1 |  |  |  |
|  | SNORD20 |  |  |  |
|  | SNORD94 |  |  |  |
|  | STPG3-AS1 |  |  |  |
|  | TAK1 |  |  |  |
|  | TEX50 |  |  |  |
|  | TLR15 |  |  |  |
|  | TRDC |  |  |  |
|  | TRPC5OS |  |  |  |
|  | TRPC7 |  |  |  |
|  | VOM2R44 |  |  |  |
|  | XKR3 |  |  |  |
|  | ELOVL4B |  |  |  |
|  | KLF11A |  |  |  |
|  | LGALS2B |  |  |  |
|  | NPM2 |  |  |  |
|  | NR1D2A |  |  |  |
|  | OAZ2B |  |  |  |
|  | PIK3R3A |  |  |  |
|  | SMPD2 |  |  |  |
|  | SPIN2B |  |  |  |
|  | TGFB1 |  |  |  |
|  | ADCY2B |  |  |  |
|  | ANP32BP1 |  |  |  |
|  | ATP6V1C1A |  |  |  |
|  | BMPR2A |  |  |  |
|  | CFL1L |  |  |  |
|  | CYP2P8 |  |  |  |
|  | DEFB104B |  |  |  |
|  | DNAJC3B |  |  |  |
|  | EIF4E2RS1 |  |  |  |
|  | EVLB |  |  |  |
|  | EXT1A |  |  |  |
|  | EZRB |  |  |  |
|  | FAAH2A |  |  |  |
|  | FKBP1AA |  |  |  |
|  | FRYB |  |  |  |
|  | GPM6AB |  |  |  |
|  | HER4.5 |  |  |  |
|  | HSPD1P5 |  |  |  |
|  | HTR1AB |  |  |  |
|  | IFT70 |  |  |  |
|  | KISS1RA |  |  |  |
|  | MCTP2B |  |  |  |
|  | MIPA |  |  |  |
|  | MSRB1A |  |  |  |
|  | MTBL |  |  |  |
|  | MYCLB |  |  |  |
|  | NLK2 |  |  |  |
|  | NOP56P2 |  |  |  |
|  | PGLYRP5 |  |  |  |
|  | PPP1CAB |  |  |  |
|  | PRKAR2AA |  |  |  |
|  | PRKCEA |  |  |  |
|  | PRR12B |  |  |  |
|  | PRRX1A |  |  |  |
|  | RAB3AA |  |  |  |
|  | RPL15P11 |  |  |  |
|  | RPS2P28 |  |  |  |
|  | RPS7P5 |  |  |  |
|  | RPSAP30 |  |  |  |
|  | RRBP1A |  |  |  |
|  | SDK1A |  |  |  |
|  | SHDB |  |  |  |
|  | SMAD3B |  |  |  |
|  | SRSF2A |  |  |  |
|  | TCF3B |  |  |  |
|  | TEAD1B |  |  |  |
|  | TIAM1A |  |  |  |
|  | TMCC1B |  |  |  |
|  | TOMM20A |  |  |  |
|  | TUSC2B |  |  |  |
|  | UBE2DNL |  |  |  |
|  | UBE2IA |  |  |  |
|  | YWHAQA |  |  |  |
|  | CYP2C6 |  |  |  |
|  | CYP2D13 |  |  |  |
|  | FTH1A |  |  |  |
|  | SAGB |  |  |  |
|  | SERPINB6 |  |  |  |
|  | SRF |  |  |  |
|  | CACNG5 |  |  |  |
|  | CYP1A2 |  |  |  |
|  | IFNG |  |  |  |
|  | BZW1B |  |  |  |
|  | CYP11B2 |  |  |  |
|  | F13A1B |  |  |  |
|  | FOS |  |  |  |
|  | FRG1BP |  |  |  |
|  | MIR1187 |  |  |  |
|  | NFE2L1A |  |  |  |
|  | NFYC-AS1 |  |  |  |
|  | NPC1B |  |  |  |
|  | PABPC1B |  |  |  |
|  | PER1A |  |  |  |
|  | PNP4B |  |  |  |
|  | RDH12L |  |  |  |
|  | SID4 |  |  |  |
|  | TEP2 |  |  |  |
|  | VEGFA |  |  |  |
|  | AIFM4 |  |  |  |
|  | B3GALT5 |  |  |  |
|  | CASP8 |  |  |  |
|  | CLDN7A |  |  |  |
|  | CTSBB |  |  |  |
|  | DHDH.1 |  |  |  |
|  | KEAP1B |  |  |  |
|  | MYBPHA |  |  |  |
|  | NAT2 |  |  |  |
|  | NFKBIA |  |  |  |
|  | TM9SF2 |  |  |  |
|  | AFP4 |  |  |  |
|  | ATP6V1BA |  |  |  |
|  | C1ORF220 |  |  |  |
|  | CDC20P1 |  |  |  |
|  | CRTC3-AS1 |  |  |  |
|  | CSTDC6 |  |  |  |
|  | DEFB27 |  |  |  |
|  | DISC1FP1 |  |  |  |
|  | ECE1-AS1 |  |  |  |
|  | FALEC |  |  |  |
|  | FLNC-AS1 |  |  |  |
|  | GPRC5D-AS1 |  |  |  |
|  | GTF2IP20 |  |  |  |
|  | H2 |  |  |  |
|  | HIF1A-AS1 |  |  |  |
|  | INKA2-AS1 |  |  |  |
|  | KRT17P2 |  |  |  |
|  | LGALS8-AS1 |  |  |  |
|  | LINC00683 |  |  |  |
|  | LINC00896 |  |  |  |
|  | LINC00973 |  |  |  |
|  | LINC00987 |  |  |  |
|  | LINC01176 |  |  |  |
|  | LINC01257 |  |  |  |
|  | LINC01504 |  |  |  |
|  | LINC01705 |  |  |  |
|  | LINC01778 |  |  |  |
|  | LINC01852 |  |  |  |
|  | LINC01907 |  |  |  |
|  | LINC02413 |  |  |  |
|  | LINC02585 |  |  |  |
|  | LINC02611 |  |  |  |
|  | LINC02724 |  |  |  |
|  | LINC02897 |  |  |  |
|  | LIPJ |  |  |  |
|  | LRRC8C-DT |  |  |  |
|  | LYPLAL1-DT |  |  |  |
|  | MANEA-DT |  |  |  |
|  | MIR7976 |  |  |  |
|  | MYLIPB |  |  |  |
|  | NAP1 |  |  |  |
|  | NREP-AS1 |  |  |  |
|  | PABPC2 |  |  |  |
|  | PFKMB |  |  |  |
|  | PYCARD-AS1 |  |  |  |
|  | RHCGL1 |  |  |  |
|  | SEPTIN7P9 |  |  |  |
|  | SEPTIN9-DT |  |  |  |
|  | SNORD11 |  |  |  |
|  | SNORD3B-2 |  |  |  |
|  | SNORD72 |  |  |  |
|  | TBILA |  |  |  |
|  | TCTEX1D4 |  |  |  |
|  | TMEM92-AS1 |  |  |  |
|  | UGT5A2 |  |  |  |
|  | VWA8-AS1 |  |  |  |
|  | ALCAMA |  |  |  |
|  | ATG4DA |  |  |  |
|  | ATP6V0A1A |  |  |  |
|  | CCL19B |  |  |  |
|  | CDHR1A |  |  |  |
|  | CHCHD4A |  |  |  |
|  | CTDSPLB |  |  |  |
|  | EEIG1B |  |  |  |
|  | EIF4BB |  |  |  |
|  | EP300A |  |  |  |
|  | FGD |  |  |  |
|  | FOXG1A |  |  |  |
|  | GOLGA2P2Y |  |  |  |
|  | HMBSA |  |  |  |
|  | HOXA2B |  |  |  |
|  | HOXC6A |  |  |  |
|  | HTRA1A |  |  |  |
|  | IL10 |  |  |  |
|  | KDM2AB |  |  |  |
|  | KIF26BA |  |  |  |
|  | KRT18P65 |  |  |  |
|  | KSR1B |  |  |  |
|  | LPAR6A |  |  |  |
|  | MAPRE1A |  |  |  |
|  | MARK2A |  |  |  |
|  | MFSD14A2 |  |  |  |
|  | MGAT1A |  |  |  |
|  | MYO9AA |  |  |  |
|  | NPM1P8 |  |  |  |
|  | OTUD5A |  |  |  |
|  | PAQR5A |  |  |  |
|  | PRMT8B |  |  |  |
|  | PTPRFB |  |  |  |
|  | RABL6A |  |  |  |
|  | RAC3A |  |  |  |
|  | RPL26P11 |  |  |  |
|  | RPL7AP36 |  |  |  |
|  | RPS2P18 |  |  |  |
|  | RPS3P6 |  |  |  |
|  | SCAF4A |  |  |  |
|  | SELENOW2A |  |  |  |
|  | SIX4B |  |  |  |
|  | SLC22A7B.3 |  |  |  |
|  | SLC25A1A |  |  |  |
|  | SMAD6B |  |  |  |
|  | SPINT1A |  |  |  |
|  | SPOPLA |  |  |  |
|  | STK35L |  |  |  |
|  | SUB1A |  |  |  |
|  | SYPL2A |  |  |  |
|  | TMX2B |  |  |  |
|  | TRIM35-27 |  |  |  |
|  | WT1B |  |  |  |
|  | ALDH9A1A.1 |  |  |  |
|  | ASCL1A |  |  |  |
|  | CYP2A12 |  |  |  |
|  | MBPB |  |  |  |
|  | MCL1A |  |  |  |
|  | RS1A |  |  |  |
|  | RTN2B |  |  |  |
|  | DRS |  |  |  |
|  | FAM83C-AS1 |  |  |  |
|  | HBBP1 |  |  |  |
|  | IFNA1 |  |  |  |
|  | LINC01169 |  |  |  |
|  | LSP1GAMMA |  |  |  |
|  | MIR125B-2 |  |  |  |
|  | MIR301 |  |  |  |
|  | MRCL3 |  |  |  |
|  | OTX2-AS1 |  |  |  |
|  | SNORD116-4 |  |  |  |
|  | TOTA |  |  |  |
|  | TPT1P8 |  |  |  |
|  | ADGRD2 |  |  |  |
|  | DNAJB8 |  |  |  |
|  | GGCTB |  |  |  |
|  | LGALS9L1 |  |  |  |
|  | MAX |  |  |  |
|  | NR5A1 |  |  |  |
|  | PRL8A9 |  |  |  |
|  | PROKR1 |  |  |  |
|  | ROM1B |  |  |  |
|  | SLC2A11L |  |  |  |
|  | SLC2A15B |  |  |  |
|  | UGT5A1 |  |  |  |
|  | ACHE |  |  |  |
|  | MAPK8 |  |  |  |
|  | ACSL1B |  |  |  |
|  | APOA4B.1 |  |  |  |
|  | ATP6V1AB |  |  |  |
|  | CAVIN4B |  |  |  |
|  | DESI1A |  |  |  |
|  | ICAM5 |  |  |  |
|  | ITLN3 |  |  |  |
|  | PDK2A |  |  |  |
|  | PFKMA |  |  |  |
|  | PRPS1B |  |  |  |
|  | SLC22A7A |  |  |  |
|  | SLC26A3.2 |  |  |  |
|  | SLC2A11B |  |  |  |
|  | SOCS1A |  |  |  |
|  | TLDC2 |  |  |  |
|  | TRP53INP1 |  |  |  |
|  | DDTL |  |  |  |
|  | MYC |  |  |  |
|  | ABCB1 |  |  |  |
|  | AMH |  |  |  |
|  | BCL2L1 |  |  |  |
|  | ICAM1 |  |  |  |
|  | ABCB6A |  |  |  |
|  | ATF5B |  |  |  |
|  | BRF1A |  |  |  |
|  | CCNI2 |  |  |  |
|  | COX5AA |  |  |  |
|  | CYP2AA12 |  |  |  |
|  | GSDMEB |  |  |  |
|  | IGFN1.3 |  |  |  |
|  | KLHL38B |  |  |  |
|  | MEP1A.1 |  |  |  |
|  | MFSD12A |  |  |  |
|  | PHKG1A |  |  |  |
|  | PLCZ1 |  |  |  |
|  | PLP1B |  |  |  |
|  | TPMT.2 |  |  |  |
|  | ADH5P4 |  |  |  |
|  | AKR1C7P |  |  |  |
|  | ARHGAP31-AS1 |  |  |  |
|  | ATE1-AS1 |  |  |  |
|  | BEAN1-AS1 |  |  |  |
|  | BLACE |  |  |  |
|  | BSN-DT |  |  |  |
|  | C17ORF113 |  |  |  |
|  | C4ORF54 |  |  |  |
|  | CDRT1 |  |  |  |
|  | CERNA1 |  |  |  |
|  | CRIM1-DT |  |  |  |
|  | CSAG3 |  |  |  |
|  | CSTA3 |  |  |  |
|  | DGAT2L7P |  |  |  |
|  | DNAJB5-DT |  |  |  |
|  | DND1P1 |  |  |  |
|  | EIF4BP7 |  |  |  |
|  | EPCIP-AS1 |  |  |  |
|  | EXOC1L |  |  |  |
|  | GDNF-AS1 |  |  |  |
|  | H2A |  |  |  |
|  | H2AX |  |  |  |
|  | H2BP2 |  |  |  |
|  | HCG20 |  |  |  |
|  | HEXA-AS1 |  |  |  |
|  | HIGD2B |  |  |  |
|  | IER3-AS1 |  |  |  |
|  | IFNA9 |  |  |  |
|  | IL6-AS1 |  |  |  |
|  | INO80-AS1 |  |  |  |
|  | ITPR1-DT |  |  |  |
|  | KLRC4-KLRK1 |  |  |  |
|  | KRT17P1 |  |  |  |
|  | LINC00355 |  |  |  |
|  | LINC00431 |  |  |  |
|  | LINC00484 |  |  |  |
|  | LINC01181 |  |  |  |
|  | LINC01198 |  |  |  |
|  | LINC01215 |  |  |  |
|  | LINC01229 |  |  |  |
|  | LINC01275 |  |  |  |
|  | LINC01358 |  |  |  |
|  | LINC01524 |  |  |  |
|  | LINC01537 |  |  |  |
|  | LINC01635 |  |  |  |
|  | LINC01719 |  |  |  |
|  | LINC02009 |  |  |  |
|  | LINC02202 |  |  |  |
|  | LINC02487 |  |  |  |
|  | LINC02547 |  |  |  |
|  | LINC02562 |  |  |  |
|  | LINC02575 |  |  |  |
|  | LINC02842 |  |  |  |
|  | LINC02872 |  |  |  |
|  | LRRC37A5P |  |  |  |
|  | MIR193BHG |  |  |  |
|  | MIR3685 |  |  |  |
|  | MIR4500HG |  |  |  |
|  | MIR599 |  |  |  |
|  | MIR6730 |  |  |  |
|  | MKRN4P |  |  |  |
|  | MYRF-AS1 |  |  |  |
|  | NAMA |  |  |  |
|  | NDUFA4A |  |  |  |
|  | NOS |  |  |  |
|  | P2RX6P |  |  |  |
|  | PAQR9-AS1 |  |  |  |
|  | PDCL3P4 |  |  |  |
|  | PPP1R2P1 |  |  |  |
|  | PRR20G |  |  |  |
|  | PSAT1P1 |  |  |  |
|  | RASAL2-AS1 |  |  |  |
|  | RNF157-AS1 |  |  |  |
|  | RORA-AS1 |  |  |  |
|  | RPL10P16 |  |  |  |
|  | RPL3P4 |  |  |  |
|  | RPL7P50 |  |  |  |
|  | RPS13P2 |  |  |  |
|  | RUNDC3A-AS1 |  |  |  |
|  | SERPINA2 |  |  |  |
|  | SGO1-AS1 |  |  |  |
|  | STK4-DT |  |  |  |
|  | TLE1P1 |  |  |  |
|  | TRNI |  |  |  |
|  | TRPC7-AS1 |  |  |  |
|  | UBE2E2-DT |  |  |  |
|  | UBE2SP1 |  |  |  |
|  | ZNF426-DT |  |  |  |
|  | ABHD10A |  |  |  |
|  | ABHD17AB |  |  |  |
|  | ADRA1AB |  |  |  |
|  | ANKRD10A |  |  |  |
|  | APBA1B |  |  |  |
|  | ARL4AA |  |  |  |
|  | ARMC1L |  |  |  |
|  | ASS1P13 |  |  |  |
|  | ATP5MC1P5 |  |  |  |
|  | ATTC |  |  |  |
|  | BRD7P3 |  |  |  |
|  | BTBD6B |  |  |  |
|  | CAP1P1 |  |  |  |
|  | CEP170AA |  |  |  |
|  | CERS2A |  |  |  |
|  | CHT4 |  |  |  |
|  | CRATB |  |  |  |
|  | CYP11A1.2 |  |  |  |
|  | DAMM |  |  |  |
|  | DIP2BA |  |  |  |
|  | ERFL3 |  |  |  |
|  | FRS2B |  |  |  |
|  | FTCDNL1 |  |  |  |
|  | FZD8B |  |  |  |
|  | GIT2B |  |  |  |
|  | GPC1A |  |  |  |
|  | HOXA1A |  |  |  |
|  | HS3ST3L |  |  |  |
|  | KCNC3B |  |  |  |
|  | LINGO4A |  |  |  |
|  | MAST1A |  |  |  |
|  | MEF2CB |  |  |  |
|  | MEKK1 |  |  |  |
|  | MINPP1A |  |  |  |
|  | MPO |  |  |  |
|  | NDEL1A |  |  |  |
|  | NR4A2B |  |  |  |
|  | NRSN2-AS1 |  |  |  |
|  | PARP12B |  |  |  |
|  | PHB1P11 |  |  |  |
|  | PHLDB2B |  |  |  |
|  | PLEKHA7A |  |  |  |
|  | PRKACAA |  |  |  |
|  | PTENP1 |  |  |  |
|  | PTK6A |  |  |  |
|  | RAB3AB |  |  |  |
|  | RBMS1A |  |  |  |
|  | RFX1A |  |  |  |
|  | RNF34A |  |  |  |
|  | RPL17P50 |  |  |  |
|  | RPL21P62 |  |  |  |
|  | RPS16P5 |  |  |  |
|  | RPS27AP17 |  |  |  |
|  | SCP2B |  |  |  |
|  | SEC11B |  |  |  |
|  | SENP3B |  |  |  |
|  | SLC25A5P7 |  |  |  |
|  | SNORD26 |  |  |  |
|  | SOUL4 |  |  |  |
|  | SRSF10B |  |  |  |
|  | ST6GAL2A |  |  |  |
|  | STOX2A |  |  |  |
|  | TLCD3BA |  |  |  |
|  | TMEM54A |  |  |  |
|  | TNS2A |  |  |  |
|  | TOX4A |  |  |  |
|  | TRAF4B |  |  |  |
|  | TTTY8 |  |  |  |
|  | VSIG8B |  |  |  |
|  | ZDHHC15B |  |  |  |
|  | ZDHHC16A |  |  |  |
|  | ZDHHC16B |  |  |  |
|  | ATP1A3A |  |  |  |
|  | BMI1A |  |  |  |
|  | CYP2AA3 |  |  |  |
|  | ETV5B |  |  |  |
|  | GUCA1D |  |  |  |
|  | MEP1A.2 |  |  |  |
|  | NUAK1A |  |  |  |
|  | OSGN1 |  |  |  |
|  | SAGA |  |  |  |
|  | SLC1A8B |  |  |  |
|  | SLC25A38A |  |  |  |
|  | LY49I4 |  |  |  |
|  | NFKB1 |  |  |  |
|  | PPAR |  |  |  |
|  | ALDOCA |  |  |  |
|  | APOA4B.3 |  |  |  |
|  | BLF |  |  |  |
|  | EHBP1L1B |  |  |  |
|  | GPR151 |  |  |  |
|  | HIST2H2L |  |  |  |
|  | ITPR1A |  |  |  |
|  | LDLRAP1A |  |  |  |
|  | PPM1NA |  |  |  |
|  | RASGEF1BA |  |  |  |
|  | RTN4RL2A |  |  |  |
|  | RTN4RL2B |  |  |  |
|  | STC1L |  |  |  |
|  | STK24A |  |  |  |
|  | TMEFF1A |  |  |  |
|  | TMPRSS13A |  |  |  |
|  | UGT2A4 |  |  |  |
|  | ZBTB2A |  |  |  |
|  | COL1A1 |  |  |  |
|  | ACTA2 |  |  |  |
|  | IL4 |  |  |  |
|  | ANO9A |  |  |  |
|  | BCO2A |  |  |  |
|  | CAMK1GA |  |  |  |
|  | CEBP1 |  |  |  |
|  | CSL |  |  |  |
|  | CYP2K21 |  |  |  |
|  | DAAM1B |  |  |  |
|  | DUSP22B |  |  |  |
|  | ELNB |  |  |  |
|  | FAS |  |  |  |
|  | FKBP1AB |  |  |  |
|  | GATA2A |  |  |  |
|  | INAA |  |  |  |
|  | MS4A17A.8 |  |  |  |
|  | NHERF4A |  |  |  |
|  | PHKG1B |  |  |  |
|  | TAB1 |  |  |  |
|  | TRPM4A |  |  |  |
|  | GLNS-PS1 |  |  |  |
|  | HSTRPA |  |  |  |
|  | KLK1C6 |  |  |  |
|  | OR5D18 |  |  |  |
|  | VOM1R90 |  |  |  |
|  | CCNB1 |  |  |  |
|  | MTOR |  |  |  |
|  | NFKBIL1 |  |  |  |
|  | SERPINE1 |  |  |  |
|  | BARAA2 |  |  |  |
|  | CKMT2B |  |  |  |
|  | CYP2AE1 |  |  |  |
|  | FKSG49 |  |  |  |
|  | FLVCR2B |  |  |  |
|  | HOXA-AS3 |  |  |  |
|  | KCNJ18 |  |  |  |
|  | LINC01126 |  |  |  |
|  | LINC01514 |  |  |  |
|  | LINC02112 |  |  |  |
|  | LYSX |  |  |  |
|  | MIR128 |  |  |  |
|  | MIR509 |  |  |  |
|  | MIR650 |  |  |  |
|  | MIRLET7F |  |  |  |
|  | PPP1R3DB |  |  |  |
|  | REREB |  |  |  |
|  | RTN4B |  |  |  |
|  | SMYD2A |  |  |  |
|  | SOX19B |  |  |  |
|  | SPE |  |  |  |
|  | SPON1A |  |  |  |
|  | TOTC |  |  |  |
|  | TOTM |  |  |  |
|  | TSF1 |  |  |  |
|  | XIRP2A |  |  |  |
|  | AQP5 |  |  |  |
|  | CYCS |  |  |  |
|  | MAPK12 |  |  |  |
|  | ACTG1P17 |  |  |  |
|  | AGGF1P2 |  |  |  |
|  | AK4P3 |  |  |  |
|  | ARHGAP26-IT1 |  |  |  |
|  | ARHGEF2-AS1 |  |  |  |
|  | BMS1P17 |  |  |  |
|  | CD44-AS1 |  |  |  |
|  | CELF2-DT |  |  |  |
|  | CFAP95-DT |  |  |  |
|  | DEFB21 |  |  |  |
|  | DNAJC19P5 |  |  |  |
|  | EEF1B2P6 |  |  |  |
|  | EHMT2-AS1 |  |  |  |
|  | EIF5AP4 |  |  |  |
|  | EPS15P1 |  |  |  |
|  | ERICD |  |  |  |
|  | FGF10-AS1 |  |  |  |
|  | FTH1P15 |  |  |  |
|  | GAGE12J |  |  |  |
|  | GAPDHP44 |  |  |  |
|  | GOLGA2P11 |  |  |  |
|  | HMGB3P32 |  |  |  |
|  | HMGN2P3 |  |  |  |
|  | HNRNPCP4 |  |  |  |
|  | IKBKE-AS1 |  |  |  |
|  | IMPDH1P5 |  |  |  |
|  | ITFG2-AS1 |  |  |  |
|  | KCTD21-AS1 |  |  |  |
|  | KRT18P32 |  |  |  |
|  | LCT-AS1 |  |  |  |
|  | LINC00298 |  |  |  |
|  | LINC00398 |  |  |  |
|  | LINC00920 |  |  |  |
|  | LINC01424 |  |  |  |
|  | LINC01526 |  |  |  |
|  | LINC01539 |  |  |  |
|  | LINC01602 |  |  |  |
|  | LINC01632 |  |  |  |
|  | LINC01633 |  |  |  |
|  | LINC01949 |  |  |  |
|  | LINC02021 |  |  |  |
|  | LINC02108 |  |  |  |
|  | LINC02139 |  |  |  |
|  | LINC02154 |  |  |  |
|  | LINC02198 |  |  |  |
|  | LINC02234 |  |  |  |
|  | LINC02290 |  |  |  |
|  | LINC02328 |  |  |  |
|  | LINC02362 |  |  |  |
|  | LINC02384 |  |  |  |
|  | LINC02392 |  |  |  |
|  | LINC02427 |  |  |  |
|  | LINC02432 |  |  |  |
|  | LINC02453 |  |  |  |
|  | LINC02478 |  |  |  |
|  | LINC02569 |  |  |  |
|  | LINC02608 |  |  |  |
|  | LINC02621 |  |  |  |
|  | LINC02765 |  |  |  |
|  | LINC02812 |  |  |  |
|  | LINC02898 |  |  |  |
|  | LRRC3-DT |  |  |  |
|  | MAP3K5-AS1 |  |  |  |
|  | MBNL1-AS1 |  |  |  |
|  | MIR3176 |  |  |  |
|  | MIR4442 |  |  |  |
|  | MIR4734 |  |  |  |
|  | MIR548V |  |  |  |
|  | MIR7111 |  |  |  |
|  | MTCO1P4 |  |  |  |
|  | NBPF17P |  |  |  |
|  | NDUFB4P11 |  |  |  |
|  | OR2S1P |  |  |  |
|  | OVAAL |  |  |  |
|  | P3H2-AS1 |  |  |  |
|  | PCDHB17P |  |  |  |
|  | PLBD1-AS1 |  |  |  |
|  | PPIAP46 |  |  |  |
|  | PPP1R35-AS1 |  |  |  |
|  | PTGES3P1 |  |  |  |
|  | RANP4 |  |  |  |
|  | RCN1P2 |  |  |  |
|  | RNU6-403P |  |  |  |
|  | RNU6-807P |  |  |  |
|  | RNU6ATAC18P |  |  |  |
|  | RPL23AP42 |  |  |  |
|  | RPL35P5 |  |  |  |
|  | RPL7AP6 |  |  |  |
|  | RPL7P38 |  |  |  |
|  | RPL7P9 |  |  |  |
|  | RPS28P7 |  |  |  |
|  | RTCA-AS1 |  |  |  |
|  | SMC2-DT |  |  |  |
|  | SMYD1 |  |  |  |
|  | SNAP23P1 |  |  |  |
|  | SNORD103C |  |  |  |
|  | SNORD14C |  |  |  |
|  | SNORD62B |  |  |  |
|  | SNX18P3 |  |  |  |
|  | TET2-AS1 |  |  |  |
|  | TLR4 |  |  |  |
|  | TMPRSS11GP |  |  |  |
|  | TNKS2-DT |  |  |  |
|  | TNPO1-DT |  |  |  |
|  | TRAJ23 |  |  |  |
|  | TRBV7-3 |  |  |  |
|  | TRNS2 |  |  |  |
|  | UVRAG-DT |  |  |  |
|  | WNT5A-AS1 |  |  |  |
|  | YAP1P1 |  |  |  |
|  | ZEB2-AS1 |  |  |  |
|  | ZNF516-DT |  |  |  |
|  | ZNF687-AS1 |  |  |  |
|  | VCAM1 |  |  |  |
|  | ADIPOQA |  |  |  |
|  | ADRB2B |  |  |  |
|  | ANKRD52A |  |  |  |
|  | ANO5B |  |  |  |
|  | ARVCFA |  |  |  |
|  | ASB12A |  |  |  |
|  | ATAD5A |  |  |  |
|  | CA4A |  |  |  |
|  | CCSER2A |  |  |  |
|  | CDK1 |  |  |  |
|  | CHMP2BB |  |  |  |
|  | CLDN34A |  |  |  |
|  | CREBBPA |  |  |  |
|  | CRISPLD1A |  |  |  |
|  | CTDNEP1A |  |  |  |
|  | DAB2IPB |  |  |  |
|  | DHX32A |  |  |  |
|  | DMRT3A |  |  |  |
|  | DRD4A |  |  |  |
|  | EIF3HA |  |  |  |
|  | FBXO30B |  |  |  |
|  | FMN2B |  |  |  |
|  | FRRS1B |  |  |  |
|  | GALR2B |  |  |  |
|  | GMPPAA |  |  |  |
|  | GUK1A |  |  |  |
|  | HMGB1A |  |  |  |
|  | HOXC5A |  |  |  |
|  | ILLR1 |  |  |  |
|  | ITGA3A |  |  |  |
|  | JUPB |  |  |  |
|  | KAT5A |  |  |  |
|  | KCNJ12B |  |  |  |
|  | LSM12B |  |  |  |
|  | MAP6A |  |  |  |
|  | MED19A |  |  |  |
|  | MYBPC2A |  |  |  |
|  | NDFIP1L |  |  |  |
|  | NPFFR1L3 |  |  |  |
|  | NPTX2B |  |  |  |
|  | NRD1B |  |  |  |
|  | OAFA |  |  |  |
|  | PARVAA |  |  |  |
|  | PAX1B |  |  |  |
|  | PCDH2AA15 |  |  |  |
|  | PHF20A |  |  |  |
|  | PIP4K2AB |  |  |  |
|  | PITPNBL |  |  |  |
|  | PKN1B |  |  |  |
|  | PLD1B |  |  |  |
|  | PRTGA |  |  |  |
|  | PTGER2A |  |  |  |
|  | RALAA |  |  |  |
|  | RBPJB |  |  |  |
|  | RCAN1A |  |  |  |
|  | RGS3B |  |  |  |
|  | RHOL |  |  |  |
|  | RIMS2A |  |  |  |
|  | SB |  |  |  |
|  | SCG2A |  |  |  |
|  | SDR16C5A |  |  |  |
|  | SFXN5B |  |  |  |
|  | SIAH2L |  |  |  |
|  | SLC35A3B |  |  |  |
|  | SLC7A14A |  |  |  |
|  | SLKA |  |  |  |
|  | STOML3A |  |  |  |
|  | SYNCRIPL |  |  |  |
|  | SYTL2A |  |  |  |
|  | TMEM222B |  |  |  |
|  | TMEM237B |  |  |  |
|  | TNKSB |  |  |  |
|  | TRPN1 |  |  |  |
|  | TSPAN13B |  |  |  |
|  | TSPAN2A |  |  |  |
|  | TSPAN34A |  |  |  |
|  | TXLNBB |  |  |  |
|  | ZFAND5B |  |  |  |
|  | ZNF207B |  |  |  |
|  | ZNF281B |  |  |  |
|  | ZP3C |  |  |  |
|  | ZPCX |  |  |  |
|  | C15H11ORF42 |  |  |  |
|  | C1H21ORF91 |  |  |  |
|  | CASP1 |  |  |  |
|  | GAB2 |  |  |  |
|  | CDKN1B |  |  |  |
|  | GADD45A |  |  |  |
|  | TIMP1 |  |  |  |
|  | SPP1 |  |  |  |
|  | BACH1A |  |  |  |
|  | CSPG5B |  |  |  |
|  | CYP2AA11 |  |  |  |
|  | CYP4V2B |  |  |  |
|  | EEF1DB |  |  |  |
|  | FLOT2B |  |  |  |
|  | GJA9B |  |  |  |
|  | KCTD9A |  |  |  |
|  | MHC1ZBA |  |  |  |
|  | NAMPT1 |  |  |  |
|  | NPFFR2A |  |  |  |
|  | NSFB |  |  |  |
|  | PCGF5A |  |  |  |
|  | PPM1LB |  |  |  |
|  | RAP1GAP2A |  |  |  |
|  | RGS7BPB |  |  |  |
|  | RHOBTB2B |  |  |  |
|  | RNF150B |  |  |  |
|  | SCG2B |  |  |  |
|  | SDHDA |  |  |  |
|  | SIX4A |  |  |  |
|  | SOUL5L |  |  |  |
|  | SPECC1LB |  |  |  |
|  | SULF2A |  |  |  |
|  | TCRA-V54 |  |  |  |
|  | CDK2 |  |  |  |
|  | CLCA4 |  |  |  |
|  | MAP1LC3B |  |  |  |
|  | CCL5 |  |  |  |
|  | CXCL2 |  |  |  |
|  | MAPK9 |  |  |  |
|  | TCIRG1 |  |  |  |
|  | ASB13A.2 |  |  |  |
|  | ASIC4B |  |  |  |
|  | C1QL3A |  |  |  |
|  | C1QL3B |  |  |  |
|  | CAMKK1B |  |  |  |
|  | CHST3A |  |  |  |
|  | CMASA |  |  |  |
|  | CRFB15 |  |  |  |
|  | DBNLB |  |  |  |
|  | HECW2A |  |  |  |
|  | LPAR2B |  |  |  |
|  | LRRC30A |  |  |  |
|  | LRRC8DB |  |  |  |
|  | MC5RA |  |  |  |
|  | MFSD6B |  |  |  |
|  | MGAA |  |  |  |
|  | NAPAA |  |  |  |
|  | NCANA |  |  |  |
|  | PCYT1AB |  |  |  |
|  | PCYT1BA |  |  |  |
|  | PIP5K1BB |  |  |  |
|  | SHISA9A |  |  |  |
|  | SLC1A9 |  |  |  |
|  | TUBAL3 |  |  |  |
|  | XGB |  |  |  |
|  | CCNA2 |  |  |  |
|  | VOM2R-PS45 |  |  |  |
|  | ABCC1 |  |  |  |
|  | CPR49AC |  |  |  |
|  | DNASEII |  |  |  |
|  | DPTB |  |  |  |
|  | IM4 |  |  |  |
|  | IRC |  |  |  |
|  | LINC01527 |  |  |  |
|  | LINC01854 |  |  |  |
|  | LSP1ALPHA |  |  |  |
|  | MESH |  |  |  |
|  | MIR520F |  |  |  |
|  | MTHL2 |  |  |  |
|  | MTK |  |  |  |
|  | P38B |  |  |  |
|  | P38C |  |  |  |
|  | PTOV1-AS2 |  |  |  |
|  | RNA45SN4 |  |  |  |
|  | RSG7 |  |  |  |
|  | SPRNP1 |  |  |  |
|  | TOTX |  |  |  |
|  | WTAPP1 |  |  |  |
|  | ZNF451-AS1 |  |  |  |
|  | BID |  |  |  |
|  | ARHGEF9A |  |  |  |
|  | CAX2 |  |  |  |
|  | CCN2 |  |  |  |
|  | CYP2K20 |  |  |  |
|  | HIPK3A |  |  |  |
|  | MHC1LAA |  |  |  |
|  | NLRP3 |  |  |  |
|  | NOTUM1B |  |  |  |
|  | OR64B4 |  |  |  |
|  | SLC2A9L2 |  |  |  |
|  | TRIM25L |  |  |  |
|  | RT1-DMB |  |  |  |
|  | UGT1A2 |  |  |  |
|  | MCL1 |  |  |  |
|  | OAMB |  |  |  |
|  | OR1D2 |  |  |  |
|  | TNFB |  |  |  |
|  | TNFSF10 |  |  |  |
|  | ABCC5-AS1 |  |  |  |
|  | ABHD17AP5 |  |  |  |
|  | ADGRF5P1 |  |  |  |
|  | ADORA2BP1 |  |  |  |
|  | AGKP1 |  |  |  |
|  | ALG1L |  |  |  |
|  | ARHGAP15-AS1 |  |  |  |
|  | ARHGAP23P1 |  |  |  |
|  | ARMC2-AS1 |  |  |  |
|  | B3GALNT2P1 |  |  |  |
|  | BCL2L1-AS1 |  |  |  |
|  | BCORP1 |  |  |  |
|  | BEND3P2 |  |  |  |
|  | BNIP3P2 |  |  |  |
|  | CALR4P |  |  |  |
|  | CARS1P2 |  |  |  |
|  | CDK2AP2P2 |  |  |  |
|  | CKS1BP1 |  |  |  |
|  | CNIH3-AS2 |  |  |  |
|  | CT75 |  |  |  |
|  | CUBNP2 |  |  |  |
|  | CYP51A1P3 |  |  |  |
|  | E2F3P1 |  |  |  |
|  | EIF1AXP1 |  |  |  |
|  | EIF4A1P10 |  |  |  |
|  | EIF4HP1 |  |  |  |
|  | ELF3-AS1 |  |  |  |
|  | ELL2P1 |  |  |  |
|  | ELN-AS1 |  |  |  |
|  | ETV7-AS1 |  |  |  |
|  | FAM71A |  |  |  |
|  | FKBP1BP1 |  |  |  |
|  | FOSL1P1 |  |  |  |
|  | FUNDC2P4 |  |  |  |
|  | GAPDHP22 |  |  |  |
|  | GAPDHP36 |  |  |  |
|  | GAPDHP75 |  |  |  |
|  | GGTA2P |  |  |  |
|  | GKN3P |  |  |  |
|  | GPR176-DT |  |  |  |
|  | GTF2IP7 |  |  |  |
|  | H2BW3P |  |  |  |
|  | HIGD1AP4 |  |  |  |
|  | HMGB1P3 |  |  |  |
|  | HMGN1P26 |  |  |  |
|  | HNRNPA1P27 |  |  |  |
|  | HSPA8P14 |  |  |  |
|  | HSPA8P4 |  |  |  |
|  | HYI-AS1 |  |  |  |
|  | IFIT1P1 |  |  |  |
|  | IGH-VS107 |  |  |  |
|  | IGKV1OR9-2 |  |  |  |
|  | KCNIP1-OT1 |  |  |  |
|  | KNOP1P1 |  |  |  |
|  | KNOP1P3 |  |  |  |
|  | KRT17P6 |  |  |  |
|  | KRT8P31 |  |  |  |
|  | LAP |  |  |  |
|  | LILRP1 |  |  |  |
|  | LINC00333 |  |  |  |
|  | LINC00412 |  |  |  |
|  | LINC00462 |  |  |  |
|  | LINC00604 |  |  |  |
|  | LINC00629 |  |  |  |
|  | LINC00677 |  |  |  |
|  | LINC01035 |  |  |  |
|  | LINC01077 |  |  |  |
|  | LINC01087 |  |  |  |
|  | LINC01127 |  |  |  |
|  | LINC01202 |  |  |  |
|  | LINC01230 |  |  |  |
|  | LINC01232 |  |  |  |
|  | LINC01283 |  |  |  |
|  | LINC01585 |  |  |  |
|  | LINC01624 |  |  |  |
|  | LINC01701 |  |  |  |
|  | LINC01736 |  |  |  |
|  | LINC01740 |  |  |  |
|  | LINC01768 |  |  |  |
|  | LINC01857 |  |  |  |
|  | LINC01888 |  |  |  |
|  | LINC01991 |  |  |  |
|  | LINC02005 |  |  |  |
|  | LINC02090 |  |  |  |
|  | LINC02132 |  |  |  |
|  | LINC02185 |  |  |  |
|  | LINC02254 |  |  |  |
|  | LINC02357 |  |  |  |
|  | LINC02416 |  |  |  |
|  | LINC02599 |  |  |  |
|  | LINC02600 |  |  |  |
|  | LINC02605 |  |  |  |
|  | LINC02606 |  |  |  |
|  | LINC02612 |  |  |  |
|  | LINC02733 |  |  |  |
|  | LINC02752 |  |  |  |
|  | LINC02848 |  |  |  |
|  | LINC02865 |  |  |  |
|  | LPP-AS1 |  |  |  |
|  | LRP1-AS |  |  |  |
|  | LRRK2-DT |  |  |  |
|  | METTL14-DT |  |  |  |
|  | MIR3945HG |  |  |  |
|  | MIR4477B |  |  |  |
|  | MIR6505 |  |  |  |
|  | MIR6753 |  |  |  |
|  | MMP2-AS1 |  |  |  |
|  | MRPS15P1 |  |  |  |
|  | MTND1P11 |  |  |  |
|  | NAP1L4P1 |  |  |  |
|  | NCK1-DT |  |  |  |
|  | NDUFA5P11 |  |  |  |
|  | NLGN4Y-AS1 |  |  |  |
|  | NRBF2P3 |  |  |  |
|  | NRIR |  |  |  |
|  | OLFM5P |  |  |  |
|  | OR6R1P |  |  |  |
|  | PARAL1 |  |  |  |
|  | PCNPP3 |  |  |  |
|  | PRAL |  |  |  |
|  | PRKACB-DT |  |  |  |
|  | PTCHD3P2 |  |  |  |
|  | PTCHD3P3 |  |  |  |
|  | RAP2CP1 |  |  |  |
|  | RHOT1P1 |  |  |  |
|  | RN7SKP203 |  |  |  |
|  | RN7SKP26 |  |  |  |
|  | RN7SKP276 |  |  |  |
|  | RN7SL124P |  |  |  |
|  | RN7SL138P |  |  |  |
|  | RN7SL326P |  |  |  |
|  | RN7SL32P |  |  |  |
|  | RN7SL470P |  |  |  |
|  | RN7SL559P |  |  |  |
|  | RN7SL751P |  |  |  |
|  | RNA5SP268 |  |  |  |
|  | RNU6-1316P |  |  |  |
|  | RNU6-173P |  |  |  |
|  | RNU6-37P |  |  |  |
|  | RNU6-638P |  |  |  |
|  | RNU6-63P |  |  |  |
|  | RNU6-925P |  |  |  |
|  | RNU6ATAC16P |  |  |  |
|  | RNU7-40P |  |  |  |
|  | RNU7-45P |  |  |  |
|  | RNVU1-32 |  |  |  |
|  | RPL10P7 |  |  |  |
|  | RPL12P27 |  |  |  |
|  | RPL23AP93 |  |  |  |
|  | RPL35AP26 |  |  |  |
|  | RPL41P5 |  |  |  |
|  | RPL7P1 |  |  |  |
|  | RPL7P24 |  |  |  |
|  | RPLP1P6 |  |  |  |
|  | RPS26P21 |  |  |  |
|  | RPS2P36 |  |  |  |
|  | RPS7P12 |  |  |  |
|  | SAPCD1-AS1 |  |  |  |
|  | SIGLEC18P |  |  |  |
|  | SLC7A14-AS1 |  |  |  |
|  | SLC9A7P1 |  |  |  |
|  | SNORD19C |  |  |  |
|  | SPATA13-AS1 |  |  |  |
|  | SPECC1P1 |  |  |  |
|  | ST3GAL5-AS1 |  |  |  |
|  | TCF3P1 |  |  |  |
|  | TMEM271 |  |  |  |
|  | TRAJ1 |  |  |  |
|  | TRAJ10 |  |  |  |
|  | TRAJ14 |  |  |  |
|  | TRAJ19 |  |  |  |
|  | TRAJ6 |  |  |  |
|  | TRBV8-2 |  |  |  |
|  | TRGV1 |  |  |  |
|  | TRGV6 |  |  |  |
|  | TRNH |  |  |  |
|  | TXNP6 |  |  |  |
|  | UBE2E1-AS1 |  |  |  |
|  | UBE2V1P2 |  |  |  |
|  | USP12-DT |  |  |  |
|  | USP12PX |  |  |  |
|  | USP12PY |  |  |  |
|  | VN1R96P |  |  |  |
|  | VTA1P1 |  |  |  |
|  | VTI1BP2 |  |  |  |
|  | WHSC1L2P |  |  |  |
|  | XBP1P1 |  |  |  |
|  | ZFY-AS1 |  |  |  |
|  | ACVR1BB |  |  |  |
|  | ANO10A |  |  |  |
|  | ANO11 |  |  |  |
|  | BAIAP2L2B |  |  |  |
|  | CBX6B |  |  |  |
|  | CCNL1B |  |  |  |
|  | CPLX3A |  |  |  |
|  | DNM3A |  |  |  |
|  | EFNA2A |  |  |  |
|  | F11R.1 |  |  |  |
|  | GLCEA |  |  |  |
|  | GRID2IPA |  |  |  |
|  | HTR2AB |  |  |  |
|  | HUG |  |  |  |
|  | KANSL1B |  |  |  |
|  | KCNN1A |  |  |  |
|  | LHFPL4B |  |  |  |
|  | MOB1BA |  |  |  |
|  | NFYBA |  |  |  |
|  | NLGN4XA |  |  |  |
|  | NPM2A |  |  |  |
|  | NYAP2A |  |  |  |
|  | PALD1B |  |  |  |
|  | PANX1A |  |  |  |
|  | PCDH15A |  |  |  |
|  | PGLYRP6 |  |  |  |
|  | PHKA1B |  |  |  |
|  | PPARAB |  |  |  |
|  | PPFIBP1A |  |  |  |
|  | PRP19 |  |  |  |
|  | RC3H1A |  |  |  |
|  | SMARCD3B |  |  |  |
|  | TMEM144B |  |  |  |
|  | TMEM238B |  |  |  |
|  | TSPAN5A |  |  |  |
|  | UBE2NB |  |  |  |
|  | ZNF687B |  |  |  |
|  | AMY2A3 |  |  |  |
|  | XIAP |  |  |  |
|  | COL3A1 |  |  |  |
|  | TNFRSF10B |  |  |  |
|  | CRP |  |  |  |
|  | OR10D5J |  |  |  |
|  | BIRC3 |  |  |  |
|  | BARX1-DT |  |  |  |
|  | CADM3-AS1 |  |  |  |
|  | EXEX |  |  |  |
|  | GBA1A |  |  |  |
|  | IM2 |  |  |  |
|  | IM3 |  |  |  |
|  | LECTIN-33A |  |  |  |
|  | LINC01924 |  |  |  |
|  | MIR101 |  |  |  |
|  | NIMB3 |  |  |  |
|  | PGRP-SD |  |  |  |
|  | PRKAR1B-AS1 |  |  |  |
|  | PRO1804 |  |  |  |
|  | SER7 |  |  |  |
|  | SOCS36E |  |  |  |
|  | SSK |  |  |  |
|  | STRICA |  |  |  |
|  | TSP2A |  |  |  |
|  | UBAC2-AS1 |  |  |  |
|  | NPPB |  |  |  |
|  | COX7A |  |  |  |
|  | HSPD1-PS7 |  |  |  |
|  | OR6C232-PS1 |  |  |  |
|  | BID |  |  |  |
|  | CFLAR |  |  |  |
|  | TRP53 |  |  |  |
|  | BIRC2 |  |  |  |
|  | HAVCR1 |  |  |  |
|  | ITGB2 |  |  |  |
|  | OR5P68 |  |  |  |
|  | COX6C2 |  |  |  |
|  | CYP2B6 |  |  |  |
|  | TRPL |  |  |  |
|  | TGFBR1 |  |  |  |
|  | CKMA |  |  |  |
|  | DRD1 |  |  |  |
|  | CCNE1 |  |  |  |
|  | TXNRD1 |  |  |  |
|  | ACAT2L1 |  |  |  |
|  | MAP1 |  |  |  |
|  | CDK4 |  |  |  |
|  | EIF4EP2 |  |  |  |
|  | FABP5P7 |  |  |  |
|  | HMGA1P3 |  |  |  |
|  | MIR5581 |  |  |  |
|  | RNA5SP82 |  |  |  |
|  | RPS20P14 |  |  |  |
|  | SPAG11BL1 |  |  |  |
|  | TNFRSF10A |  |  |  |
|  | YBX1P10 |  |  |  |
|  | BRAF |  |  |  |
|  | CACNG1B |  |  |  |
|  | CASP8L1 |  |  |  |
|  | CHTOPB |  |  |  |
|  | CNN1A |  |  |  |
|  | CYSLTR2B |  |  |  |
|  | FC18A08 |  |  |  |
|  | HECW1B |  |  |  |
|  | IM |  |  |  |
|  | KOP |  |  |  |
|  | MCL1 |  |  |  |
|  | NXPH2B |  |  |  |
|  | OLFCK2 |  |  |  |
|  | RIC3B |  |  |  |
|  | RPL5A |  |  |  |
|  | SC |  |  |  |
|  | SCOCA |  |  |  |
|  | SEPTIN7B |  |  |  |
|  | TLCD5A |  |  |  |
|  | TMEM169B |  |  |  |
|  | TMEM182A |  |  |  |
|  | TMEM264 |  |  |  |
|  | TOR1 |  |  |  |
|  | ZMP:0000001200 |  |  |  |
|  | AHRRA |  |  |  |
|  | BIB |  |  |  |
|  | IL2RA |  |  |  |
|  | FGF2 |  |  |  |
|  | CKM |  |  |  |
|  | PDE5A |  |  |  |
|  | GSDMD |  |  |  |
|  | POR |  |  |  |
|  | ACBP6 |  |  |  |
|  | AMNIONLESS |  |  |  |
|  | ARPC3B |  |  |  |
|  | BOMBC1 |  |  |  |
|  | CECC |  |  |  |
|  | CHT9 |  |  |  |
|  | CRY |  |  |  |
|  | FAM27D1 |  |  |  |
|  | GNBP-LIKE3 |  |  |  |
|  | HDLY |  |  |  |
|  | IM1 |  |  |  |
|  | INX7 |  |  |  |
|  | KIZ-AS1 |  |  |  |
|  | LEMD1-AS1 |  |  |  |
|  | LINC00226 |  |  |  |
|  | LINC00572 |  |  |  |
|  | LINC00919 |  |  |  |
|  | LINC01242 |  |  |  |
|  | LINC01922 |  |  |  |
|  | LINC02114 |  |  |  |
|  | LINC02232 |  |  |  |
|  | LINC02352 |  |  |  |
|  | LINC02683 |  |  |  |
|  | MIR550 |  |  |  |
|  | MSL-1 |  |  |  |
|  | NAZO |  |  |  |
|  | NIMB1 |  |  |  |
|  | NPC2E |  |  |  |
|  | PGRP-SC1A |  |  |  |
|  | RPS4XP3 |  |  |  |
|  | SCB |  |  |  |
|  | SID |  |  |  |
|  | SP212 |  |  |  |
|  | SPN88EB |  |  |  |
|  | CD69 |  |  |  |
|  | PSMA1 |  |  |  |
|  | MAP3K7 |  |  |  |
|  | CDK3 |  |  |  |
|  | F3 |  |  |  |
|  | PSMC6 |  |  |  |
|  | THRB |  |  |  |
|  | TOP2A |  |  |  |
|  | MIR34A |  |  |  |
|  | PRF1 |  |  |  |
|  | MT2A |  |  |  |
|  | PSMA3 |  |  |  |
|  | CDH2 |  |  |  |
|  | BIRC3 |  |  |  |
|  | APOA1 |  |  |  |
|  | HRAS |  |  |  |
|  | PINK-1 |  |  |  |
|  | DNMT1 |  |  |  |
|  | RAP1B |  |  |  |
|  | RUNX2 |  |  |  |
|  | ACE |  |  |  |
|  | AKR1C3 |  |  |  |
|  | THRA |  |  |  |
|  | CAC |  |  |  |
|  | CTSB |  |  |  |
|  | SCD1 |  |  |  |
|  | MAPT |  |  |  |
|  | AKR1C1 |  |  |  |
|  | ATF6 |  |  |  |
|  | CCN1 |  |  |  |
|  | ERBB2 |  |  |  |
|  | MAOA |  |  |  |
|  | TYMS |  |  |  |
|  | GJA1 |  |  |  |
|  | PRL |  |  |  |
|  | TGFB2 |  |  |  |
|  | TNFAIP3 |  |  |  |
|  | PLAUR |  |  |  |
|  | MT1 |  |  |  |
|  | CYP2D6 |  |  |  |
|  | CCNB2 |  |  |  |
|  | LPR |  |  |  |
|  | NRG1 |  |  |  |
|  | DRD2 |  |  |  |
|  | GSTA4 |  |  |  |
|  | KDR |  |  |  |
|  | ATG5 |  |  |  |
|  | HSF1 |  |  |  |
|  | NOTCH1 |  |  |  |
|  | AKR1C2 |  |  |  |
|  | BRCA1 |  |  |  |
|  | GSTA1 |  |  |  |
|  | SP1 |  |  |  |
|  | CYP3A11 |  |  |  |
|  | HSP90AA1 |  |  |  |
|  | FLT1 |  |  |  |
|  | ENO1 |  |  |  |
|  | HSD3B1 |  |  |  |
|  | PON1 |  |  |  |
|  | MIRLET7B |  |  |  |
|  | HSP90AB1 |  |  |  |
|  | PRDX1 |  |  |  |
|  | SNCA |  |  |  |
|  | LSS |  |  |  |
|  | PHGDH |  |  |  |
|  | IDI1 |  |  |  |
|  | MIR26B |  |  |  |
|  | STAT1A |  |  |  |
|  | ID3 |  |  |  |
|  | INSR |  |  |  |
|  | MGST1 |  |  |  |
|  | PRKCD |  |  |  |
|  | AURKA |  |  |  |
|  | SMAD2 |  |  |  |
|  | BIRC2 |  |  |  |
|  | DNAJB1 |  |  |  |
|  | GRIN2B |  |  |  |
|  | CASP2 |  |  |  |
|  | CYBA |  |  |  |
|  | A2M |  |  |  |
|  | CES1 |  |  |  |
|  | MMP14 |  |  |  |
|  | CYP2B1 |  |  |  |
|  | GGT1 |  |  |  |
|  | NR0B2 |  |  |  |
|  | JUND |  |  |  |
|  | HSD11B2 |  |  |  |
|  | IGFBP1 |  |  |  |
|  | FOSB |  |  |  |
|  | UGT1A6 |  |  |  |
|  | CCND3 |  |  |  |
|  | EDNRB |  |  |  |
|  | KIT |  |  |  |
|  | PER2 |  |  |  |
|  | PLA2G4A |  |  |  |
|  | NPY |  |  |  |
|  | CD68 |  |  |  |
|  | DHCR24 |  |  |  |
|  | SLC40A1 |  |  |  |
|  | MAP3K5 |  |  |  |
|  | SOX2 |  |  |  |
|  | CA-ALPHA1D |  |  |  |
|  | DHFR |  |  |  |
|  | PDGFA |  |  |  |
|  | SP120 |  |  |  |
|  | AOX1 |  |  |  |
|  | CALR |  |  |  |
|  | RAF1 |  |  |  |
|  | ABCB1A |  |  |  |
|  | ATG7 |  |  |  |
|  | CASP4 |  |  |  |
|  | EGR2 |  |  |  |
|  | NR1H3 |  |  |  |
|  | PRKN |  |  |  |
|  | NAMPT |  |  |  |
|  | NFKB2 |  |  |  |
|  | FDPS |  |  |  |
|  | MCM6 |  |  |  |
|  | PINK1 |  |  |  |
|  | SLC1A2 |  |  |  |
|  | TGFBR1 |  |  |  |
|  | TSC22D3 |  |  |  |
|  | ABCC4 |  |  |  |
|  | CASP12 |  |  |  |
|  | PTGES |  |  |  |
|  | SLC16A1 |  |  |  |
|  | TSC22D1 |  |  |  |
|  | AQP3 |  |  |  |
|  | RARB |  |  |  |
|  | BMP6 |  |  |  |
|  | ENC1 |  |  |  |
|  | HMGB2 |  |  |  |
|  | CA2 |  |  |  |
|  | FADD |  |  |  |
|  | HSPB8 |  |  |  |
|  | GATA4 |  |  |  |
|  | LGALS3 |  |  |  |
|  | NPM1 |  |  |  |
|  | STMN1 |  |  |  |
|  | FABP5 |  |  |  |
|  | FOXO3 |  |  |  |
|  | AIF1 |  |  |  |
|  | GLS |  |  |  |
|  | SRD5A1 |  |  |  |
|  | EIF2S1 |  |  |  |
|  | MME |  |  |  |
|  | TPI1 |  |  |  |
|  | DNAJA1 |  |  |  |
|  | STS |  |  |  |
|  | TGFBI |  |  |  |
|  | CTSC |  |  |  |
|  | DNAJB4 |  |  |  |
|  | FBN1 |  |  |  |
|  | GLRX |  |  |  |
|  | SLPI |  |  |  |
|  | TUBB3 |  |  |  |
|  | APC |  |  |  |
|  | CNR2 |  |  |  |
|  | SDC4 |  |  |  |
|  | SFN |  |  |  |
|  | CES2 |  |  |  |
|  | FTL |  |  |  |
|  | KITLG |  |  |  |
|  | SOD3 |  |  |  |
|  | ALDH3A1 |  |  |  |
|  | COX1 |  |  |  |
|  | DCN |  |  |  |
|  | PDIA4 |  |  |  |
|  | SERPINA1 |  |  |  |
|  | ABCG1 |  |  |  |
|  | CAPN2 |  |  |  |
|  | ITPR1 |  |  |  |
|  | NRP1 |  |  |  |
|  | PTPN1 |  |  |  |
|  | FGF1 |  |  |  |
|  | CD55 |  |  |  |
|  | FGA |  |  |  |
|  | MDH1 |  |  |  |
|  | ACTN1 |  |  |  |
|  | ECH1 |  |  |  |
|  | MMP12 |  |  |  |
|  | NCOA1 |  |  |  |
|  | OPRM1 |  |  |  |
|  | TERT |  |  |  |
|  | AR |  |  |  |
|  | BRCA2 |  |  |  |
|  | FGB |  |  |  |
|  | SMAD7 |  |  |  |
|  | TRIB1 |  |  |  |
|  | TUBA4A |  |  |  |
|  | ALDH1A3 |  |  |  |
|  | HSPE1 |  |  |  |
|  | OSGIN1 |  |  |  |
|  | ANXA4 |  |  |  |
|  | APOA4 |  |  |  |
|  | IGF2R |  |  |  |
|  | STIP1 |  |  |  |
|  | CYP2A6 |  |  |  |
|  | GRN |  |  |  |
|  | IFIT3 |  |  |  |
|  | KYNU |  |  |  |
|  | PRKDC |  |  |  |
|  | SLC7A1 |  |  |  |
|  | UGT1A7 |  |  |  |
|  | VCL |  |  |  |
|  | BCL3 |  |  |  |
|  | CPT1B |  |  |  |
|  | DKK1 |  |  |  |
|  | IFIT1 |  |  |  |
|  | KLF5 |  |  |  |
|  | KLF9 |  |  |  |
|  | TPX2 |  |  |  |
|  | DLG4 |  |  |  |
|  | ITGB3 |  |  |  |
|  | MX1 |  |  |  |
|  | TMPRSS2 |  |  |  |
|  | TNFRSF9 |  |  |  |
|  | BACE1 |  |  |  |
|  | FGFR3 |  |  |  |
|  | PARK7 |  |  |  |
|  | SPTBN1 |  |  |  |
|  | TSPO |  |  |  |
|  | CX3CL1 |  |  |  |
|  | MAD2L1 |  |  |  |
|  | NDC80 |  |  |  |
|  | NFKBIZ |  |  |  |
|  | PLCB1 |  |  |  |
|  | PTGER4 |  |  |  |
|  | SREBF2 |  |  |  |
|  | TAP1 |  |  |  |
|  | CXCL14 |  |  |  |
|  | CXCL3 |  |  |  |
|  | OAT |  |  |  |
|  | NRF1 |  |  |  |
|  | RASD1 |  |  |  |
|  | UGT1A9 |  |  |  |
|  | NME1 |  |  |  |
|  | RGS16 |  |  |  |
|  | SLC18A2 |  |  |  |
|  | ZEB1 |  |  |  |
|  | AHNAK |  |  |  |
|  | ATP5F1B |  |  |  |
|  | CD9 |  |  |  |
|  | FGF21 |  |  |  |
|  | FLNA |  |  |  |
|  | PRKAA2 |  |  |  |
|  | UPP1 |  |  |  |
|  | ARC |  |  |  |
|  | DLGAP5 |  |  |  |
|  | NEFL |  |  |  |
|  | OPA1 |  |  |  |
|  | PCSK9 |  |  |  |
|  | SFPQ |  |  |  |
|  | EMP1 |  |  |  |
|  | ERCC1 |  |  |  |
|  | LUM |  |  |  |
|  | MITF |  |  |  |
|  | PGD |  |  |  |
|  | RSAD2 |  |  |  |
|  | SLC16A3 |  |  |  |
|  | HSPA6 |  |  |  |
|  | MRC1 |  |  |  |
|  | SC5D |  |  |  |
|  | VCP |  |  |  |
|  | EEF2 |  |  |  |
|  | MAFB |  |  |  |
|  | RORA |  |  |  |
|  | ITGA4 |  |  |  |
|  | RPSA |  |  |  |
|  | AHCY |  |  |  |
|  | ALDH1A2 |  |  |  |
|  | DUSP4 |  |  |  |
|  | GNRH1 |  |  |  |
|  | CYP51 |  |  |  |
|  | EREG |  |  |  |
|  | GSTO1 |  |  |  |
|  | KLHL24 |  |  |  |
|  | NCL |  |  |  |
|  | TPM4 |  |  |  |
|  | ABAT |  |  |  |
|  | AQP9 |  |  |  |
|  | IFITM1 |  |  |  |
|  | MAP1B |  |  |  |
|  | MB |  |  |  |
|  | MYH6 |  |  |  |
|  | RRAD |  |  |  |
|  | ALOX15 |  |  |  |
|  | CCNF |  |  |  |
|  | DRD1 |  |  |  |
|  | GK |  |  |  |
|  | RGCC |  |  |  |
|  | TLR3 |  |  |  |
|  | TUBA1B |  |  |  |
|  | ZEB2 |  |  |  |
|  | BLVRB |  |  |  |
|  | CHRNA4 |  |  |  |
|  | MCAM |  |  |  |
|  | MVP |  |  |  |
|  | NAT1 |  |  |  |
|  | WEE1 |  |  |  |
|  | GATM |  |  |  |
|  | PPIA |  |  |  |
|  | PRLR |  |  |  |
|  | RBP4 |  |  |  |
|  | AXIN2 |  |  |  |
|  | GHR |  |  |  |
|  | HNRNPA2B1 |  |  |  |
|  | MYL9 |  |  |  |
|  | NANOG |  |  |  |
|  | PPL |  |  |  |
|  | PRSS23 |  |  |  |
|  | VNN1 |  |  |  |
|  | WT1 |  |  |  |
|  | BMF |  |  |  |
|  | EPHA2 |  |  |  |
|  | JUP |  |  |  |
|  | SDC1 |  |  |  |
|  | HSD17B1 |  |  |  |
|  | HSD3B2 |  |  |  |
|  | NFIA |  |  |  |
|  | RAC2 |  |  |  |
|  | TFPI2 |  |  |  |
|  | USP18 |  |  |  |
|  | CCR5 |  |  |  |
|  | EGR3 |  |  |  |
|  | EZR |  |  |  |
|  | IL15 |  |  |  |
|  | LDHB |  |  |  |
|  | PRDX6 |  |  |  |
|  | TFAP2A |  |  |  |
|  | TNNI3 |  |  |  |
|  | CKM |  |  |  |
|  | DGAT1 |  |  |  |
|  | DST |  |  |  |
|  | MAF |  |  |  |
|  | MVK |  |  |  |
|  | PGF |  |  |  |
|  | TUBA1C |  |  |  |
|  | DDAH1 |  |  |  |
|  | GCK |  |  |  |
|  | HTR1A |  |  |  |
|  | INHBB |  |  |  |
|  | PTPN11 |  |  |  |
|  | SLC39A14 |  |  |  |
|  | YAP1 |  |  |  |
|  | DUSP10 |  |  |  |
|  | IGFBP6 |  |  |  |
|  | ITGB5 |  |  |  |
|  | SLC31A1 |  |  |  |
|  | WNT5A |  |  |  |
|  | CBR3 |  |  |  |
|  | COTL1 |  |  |  |
|  | MAP2K3 |  |  |  |
|  | SLC25A4 |  |  |  |
|  | SLC39A8 |  |  |  |
|  | ALDH1B1 |  |  |  |
|  | HMGA2 |  |  |  |
|  | PNPLA2 |  |  |  |
|  | XRCC5 |  |  |  |
|  | DECR1 |  |  |  |
|  | IL7R |  |  |  |
|  | CSRP2 |  |  |  |
|  | ACSL5 |  |  |  |
|  | ANGPT1 |  |  |  |
|  | ARHGDIB |  |  |  |
|  | ATP1A2 |  |  |  |
|  | CFH |  |  |  |
|  | CTPS1 |  |  |  |
|  | HAL |  |  |  |
|  | IER2 |  |  |  |
|  | IFI44 |  |  |  |
|  | IRAK2 |  |  |  |
|  | SERPINF1 |  |  |  |
|  | SHMT1 |  |  |  |
|  | TMEM97 |  |  |  |
|  | TNFRSF19 |  |  |  |
|  | TYMP |  |  |  |
|  | UBC |  |  |  |
|  | FAH |  |  |  |
|  | GLDC |  |  |  |
|  | MAP2K4 |  |  |  |
|  | BAMBI |  |  |  |
|  | CLIC4 |  |  |  |
|  | CLK1 |  |  |  |
|  | FOXP1 |  |  |  |
|  | MALAT1 |  |  |  |
|  | MYBL2 |  |  |  |
|  | NFIX |  |  |  |
|  | RHOC |  |  |  |
|  | SLCO1A4 |  |  |  |
|  | AMPD3 |  |  |  |
|  | BST2 |  |  |  |
|  | FMO3 |  |  |  |
|  | GATA2 |  |  |  |
|  | HRH1 |  |  |  |
|  | NOCT |  |  |  |
|  | PID1 |  |  |  |
|  | PLEC |  |  |  |
|  | RBL2 |  |  |  |
|  | SCP2 |  |  |  |
|  | CAMK2A |  |  |  |
|  | CAPG |  |  |  |
|  | DUT |  |  |  |
|  | MAP4K4 |  |  |  |
|  | NR5A2 |  |  |  |
|  | IDH3A |  |  |  |
|  | NRIP1 |  |  |  |
|  | PTPRC |  |  |  |
|  | SLC1A4 |  |  |  |
|  | TCF7L2 |  |  |  |
|  | ADAM17 |  |  |  |
|  | GRB10 |  |  |  |
|  | IKBKG |  |  |  |
|  | LGMN |  |  |  |
|  | MAPKAPK2 |  |  |  |
|  | MSN |  |  |  |
|  | TNFSF9 |  |  |  |
|  | TP63 |  |  |  |
|  | ADGRE1 |  |  |  |
|  | BCAT1 |  |  |  |
|  | DSP |  |  |  |
|  | FLNC |  |  |  |
|  | GAP43 |  |  |  |
|  | HDAC5 |  |  |  |
|  | ISG20 |  |  |  |
|  | PRKACB |  |  |  |
|  | SIRT3 |  |  |  |
|  | SLC16A6 |  |  |  |
|  | TRIP13 |  |  |  |
|  | ZFP36L1 |  |  |  |
|  | ACKR3 |  |  |  |
|  | CALM1 |  |  |  |
|  | COX2 |  |  |  |
|  | CRISPLD2 |  |  |  |
|  | CYP2C8 |  |  |  |
|  | FMO5 |  |  |  |
|  | KMT2A |  |  |  |
|  | LAMB1 |  |  |  |
|  | MX2 |  |  |  |
|  | UGT1A3 |  |  |  |
|  | YWHAE |  |  |  |
|  | ZMAT3 |  |  |  |
|  | ADH1 |  |  |  |
|  | ALDH1L2 |  |  |  |
|  | APOD |  |  |  |
|  | FCGR2B |  |  |  |
|  | GDNF |  |  |  |
|  | MYH9 |  |  |  |
|  | NOX1 |  |  |  |
|  | PAICS |  |  |  |
|  | PAX6 |  |  |  |
|  | S100A11 |  |  |  |
|  | TNFRSF21 |  |  |  |
|  | VTN |  |  |  |
|  | DBI |  |  |  |
|  | EIF4G1 |  |  |  |
|  | FSHR |  |  |  |
|  | GREM1 |  |  |  |
|  | MTHFD1L |  |  |  |
|  | NR1D2 |  |  |  |
|  | RPL6 |  |  |  |
|  | SERTAD1 |  |  |  |
|  | SRSF5 |  |  |  |
|  | STAT2 |  |  |  |
|  | CCL6 |  |  |  |
|  | SDHB |  |  |  |
|  | SPRY4 |  |  |  |
|  | TEK |  |  |  |
|  | CKS1B |  |  |  |
|  | IER5 |  |  |  |
|  | LAMB3 |  |  |  |
|  | PRMT1 |  |  |  |
|  | ATG12 |  |  |  |
|  | CSTB |  |  |  |
|  | CYP2C11 |  |  |  |
|  | HEY1 |  |  |  |
|  | RPL13 |  |  |  |
|  | TMT1A |  |  |  |
|  | ARRB1 |  |  |  |
|  | CD69 |  |  |  |
|  | CORO1A |  |  |  |
|  | LAMA5 |  |  |  |
|  | P2RX7 |  |  |  |
|  | RETN |  |  |  |
|  | SLC22A5 |  |  |  |
|  | CLDN3 |  |  |  |
|  | FOXA2 |  |  |  |
|  | LTB |  |  |  |
|  | LTBP1 |  |  |  |
|  | RPS6KA1 |  |  |  |
|  | SLC7A2 |  |  |  |
|  | TDO2 |  |  |  |
|  | TSLP |  |  |  |
|  | VAT1 |  |  |  |
|  | ARPC1B |  |  |  |
|  | CRABP2 |  |  |  |
|  | ELN |  |  |  |
|  | GLI2 |  |  |  |
|  | POLA1 |  |  |  |
|  | SPRY2 |  |  |  |
|  | TALDO1 |  |  |  |
|  | TNFAIP8 |  |  |  |
|  | TSC2 |  |  |  |
|  | BCLAF1 |  |  |  |
|  | CAR3 |  |  |  |
|  | ENG |  |  |  |
|  | H19 |  |  |  |
|  | HNMT |  |  |  |
|  | NABP1 |  |  |  |
|  | SRRM2 |  |  |  |
|  | TRAF2 |  |  |  |
|  | UGCG |  |  |  |
|  | RPS2 |  |  |  |
|  | SH3KBP1 |  |  |  |
|  | ADD3 |  |  |  |
|  | ASPH |  |  |  |
|  | BASP1 |  |  |  |
|  | DCLK1 |  |  |  |
|  | DUSP2 |  |  |  |
|  | EEF2K |  |  |  |
|  | ELAVL1 |  |  |  |
|  | SPON1 |  |  |  |
|  | C5 |  |  |  |
|  | ELF3 |  |  |  |
|  | MFGE8 |  |  |  |
|  | MFSD2A |  |  |  |
|  | OPTN |  |  |  |
|  | PROC |  |  |  |
|  | RELB |  |  |  |
|  | RFC3 |  |  |  |
|  | RPS19 |  |  |  |
|  | STK11 |  |  |  |
|  | THBS2 |  |  |  |
|  | TRAF1 |  |  |  |
|  | CCL22 |  |  |  |
|  | CLCF1 |  |  |  |
|  | FUBP1 |  |  |  |
|  | NAV2 |  |  |  |
|  | PFKM |  |  |  |
|  | PRDM1 |  |  |  |
|  | S100A10 |  |  |  |
|  | SMAD1 |  |  |  |
|  | ALDH1L1 |  |  |  |
|  | COL6A2 |  |  |  |
|  | ESD |  |  |  |
|  | HPSE |  |  |  |
|  | IL7 |  |  |  |
|  | INPP5D |  |  |  |
|  | ITPR3 |  |  |  |
|  | MMP7 |  |  |  |
|  | PSAP |  |  |  |
|  | QSOX1 |  |  |  |
|  | RPL10A |  |  |  |
|  | CD38 |  |  |  |
|  | COL8A1 |  |  |  |
|  | GHRL |  |  |  |
|  | MYO1B |  |  |  |
|  | NAP1L1 |  |  |  |
|  | RASGRP1 |  |  |  |
|  | SLC8A1 |  |  |  |
|  | ANXA6 |  |  |  |
|  | CPEB2 |  |  |  |
|  | FBN2 |  |  |  |
|  | GART |  |  |  |
|  | HBP1 |  |  |  |
|  | IFIH1 |  |  |  |
|  | KIF1B |  |  |  |
|  | PSEN1 |  |  |  |
|  | PSRC1 |  |  |  |
|  | SLC25A10 |  |  |  |
|  | TPP1 |  |  |  |
|  | CDO1 |  |  |  |
|  | CREBRF |  |  |  |
|  | CYB5R3 |  |  |  |
|  | ELOVL5 |  |  |  |
|  | FSTL1 |  |  |  |
|  | HADHB |  |  |  |
|  | ROCK1 |  |  |  |
|  | SLC7A7 |  |  |  |
|  | SLCO2A1 |  |  |  |
|  | TIA1 |  |  |  |
|  | ANK3 |  |  |  |
|  | CACNA1C |  |  |  |
|  | CAMK4 |  |  |  |
|  | CGA |  |  |  |
|  | CSAD |  |  |  |
|  | DHRS4 |  |  |  |
|  | FOXQ1 |  |  |  |
|  | FSCN1 |  |  |  |
|  | ITGA2 |  |  |  |
|  | PFKFB4 |  |  |  |
|  | PTPRK |  |  |  |
|  | RARRES1 |  |  |  |
|  | SMOC1 |  |  |  |
|  | AEN |  |  |  |
|  | ARHGEF2 |  |  |  |
|  | CDH13 |  |  |  |
|  | CREB5 |  |  |  |
|  | EPS8 |  |  |  |
|  | ETV4 |  |  |  |
|  | JMJD6 |  |  |  |
|  | MTSS1 |  |  |  |
|  | ST6GAL1 |  |  |  |
|  | ABCF1 |  |  |  |
|  | CNN3 |  |  |  |
|  | HNRNPAB |  |  |  |
|  | MASP1 |  |  |  |
|  | NEU1 |  |  |  |
|  | PRKCH |  |  |  |
|  | RPL17 |  |  |  |
|  | SMPD1 |  |  |  |
|  | SPINK1 |  |  |  |
|  | HNRNPDL |  |  |  |
|  | MAP3K7 |  |  |  |
|  | NCEH1 |  |  |  |
|  | OXCT1 |  |  |  |
|  | PPP2CA |  |  |  |
|  | TNFRSF1B |  |  |  |
|  | ABCC9 |  |  |  |
|  | CHKA |  |  |  |
|  | CLDN2 |  |  |  |
|  | CNN1 |  |  |  |
|  | CSK |  |  |  |
|  | CYP4A10 |  |  |  |
|  | ITGA1 |  |  |  |
|  | UGT1A10 |  |  |  |
|  | UNC5B |  |  |  |
|  | XAF1 |  |  |  |
|  | ARNT2 |  |  |  |
|  | CCT2 |  |  |  |
|  | FABP7 |  |  |  |
|  | RBPMS |  |  |  |
|  | SLC7A8 |  |  |  |
|  | SYK |  |  |  |
|  | XPA |  |  |  |
|  | ACTG2 |  |  |  |
|  | ANTXR1 |  |  |  |
|  | ARID5B |  |  |  |
|  | CD163 |  |  |  |
|  | CMPK2 |  |  |  |
|  | COL7A1 |  |  |  |
|  | DIAPH3 |  |  |  |
|  | ESM1 |  |  |  |
|  | FOXA1 |  |  |  |
|  | IFI6 |  |  |  |
|  | LIPA |  |  |  |
|  | MTR |  |  |  |
|  | VDAC2 |  |  |  |
|  | AGTR2 |  |  |  |
|  | ALOX5AP |  |  |  |
|  | CBLB |  |  |  |
|  | NDRG2 |  |  |  |
|  | PNPLA3 |  |  |  |
|  | SLC25A25 |  |  |  |
|  | STEAP4 |  |  |  |
|  | TARDBP |  |  |  |
|  | UBE2T |  |  |  |
|  | BAAT |  |  |  |
|  | CELF2 |  |  |  |
|  | CYLD |  |  |  |
|  | FZD7 |  |  |  |
|  | LFNG |  |  |  |
|  | LRP8 |  |  |  |
|  | PDK2 |  |  |  |
|  | STAT4 |  |  |  |
|  | TGM1 |  |  |  |
|  | ACSF2 |  |  |  |
|  | ARRDC4 |  |  |  |
|  | EBF1 |  |  |  |
|  | GALE |  |  |  |
|  | GNB1 |  |  |  |
|  | HDAC9 |  |  |  |
|  | TRIO |  |  |  |
|  | WSB1 |  |  |  |
|  | WWTR1 |  |  |  |
|  | ADAMTS5 |  |  |  |
|  | ARID1A |  |  |  |
|  | ATP5MC1 |  |  |  |
|  | CA12 |  |  |  |
|  | CEMIP2 |  |  |  |
|  | EGLN1 |  |  |  |
|  | IFNB1 |  |  |  |
|  | LPO |  |  |  |
|  | MCF2L |  |  |  |
|  | NEFH |  |  |  |
|  | RBBP4 |  |  |  |
|  | TCF7 |  |  |  |
|  | UGT1A8 |  |  |  |
|  | DLC1 |  |  |  |
|  | FKBP1A |  |  |  |
|  | MAP4 |  |  |  |
|  | PDLIM7 |  |  |  |
|  | USP7 |  |  |  |
|  | ADAM19 |  |  |  |
|  | CEMIP |  |  |  |
|  | CLDN5 |  |  |  |
|  | COX17 |  |  |  |
|  | HSPG2 |  |  |  |
|  | LRRC59 |  |  |  |
|  | MERTK |  |  |  |
|  | NCOA4 |  |  |  |
|  | PEG10 |  |  |  |
|  | PRODH |  |  |  |
|  | RAB31 |  |  |  |
|  | RGS1 |  |  |  |
|  | BCL11A |  |  |  |
|  | COLEC12 |  |  |  |
|  | FLCN |  |  |  |
|  | GPI |  |  |  |
|  | LHFPL6 |  |  |  |
|  | NUCB2 |  |  |  |
|  | PDE3A |  |  |  |
|  | PFKL |  |  |  |
|  | PRICKLE1 |  |  |  |
|  | SIRT2 |  |  |  |
|  | SLC5A3 |  |  |  |
|  | ABL1 |  |  |  |
|  | CHD2 |  |  |  |
|  | F11R |  |  |  |
|  | IPO5 |  |  |  |
|  | RBM39 |  |  |  |
|  | SARS1 |  |  |  |
|  | STAT6 |  |  |  |
|  | STX3 |  |  |  |
|  | UMPS |  |  |  |
|  | ARL4D |  |  |  |
|  | CYP2A5 |  |  |  |
|  | ELOVL2 |  |  |  |
|  | HIVEP2 |  |  |  |
|  | HMBS |  |  |  |
|  | ITGAL |  |  |  |
|  | KNL1 |  |  |  |
|  | LAPTM5 |  |  |  |
|  | LTA |  |  |  |
|  | PLAC8 |  |  |  |
|  | RFTN1 |  |  |  |
|  | RPL7 |  |  |  |
|  | SLC34A2 |  |  |  |
|  | SLC5A1 |  |  |  |
|  | TPR |  |  |  |
|  | VMP1 |  |  |  |
|  | YBX1 |  |  |  |
|  | YWHAH |  |  |  |
|  | ADRA2A |  |  |  |
|  | CCL17 |  |  |  |
|  | DHX58 |  |  |  |
|  | FAM43A |  |  |  |
|  | FNDC3B |  |  |  |
|  | MYO10 |  |  |  |
|  | UCP3 |  |  |  |
|  | ACTC1 |  |  |  |
|  | EPOR |  |  |  |
|  | FBXO30 |  |  |  |
|  | GC |  |  |  |
|  | GPCPD1 |  |  |  |
|  | NUCKS1 |  |  |  |
|  | AQP7 |  |  |  |
|  | ASAH1 |  |  |  |
|  | GSDME |  |  |  |
|  | HHEX |  |  |  |
|  | PRKAR2B |  |  |  |
|  | PSPH |  |  |  |
|  | RCN1 |  |  |  |
|  | SLC25A1 |  |  |  |
|  | SLIT3 |  |  |  |
|  | WWOX |  |  |  |
|  | AK2 |  |  |  |
|  | DCT |  |  |  |
|  | DKK3 |  |  |  |
|  | EHD1 |  |  |  |
|  | GPD2 |  |  |  |
|  | HMGN2 |  |  |  |
|  | HOXA1 |  |  |  |
|  | IFI35 |  |  |  |
|  | IREB2 |  |  |  |
|  | KCTD12 |  |  |  |
|  | MXD4 |  |  |  |
|  | P2RX4 |  |  |  |
|  | PADI2 |  |  |  |
|  | RAMP1 |  |  |  |
|  | RUNX1T1 |  |  |  |
|  | SLC22A23 |  |  |  |
|  | STARD13 |  |  |  |
|  | TLN1 |  |  |  |
|  | ADH7 |  |  |  |
|  | CLMN |  |  |  |
|  | DACT1 |  |  |  |
|  | GCHFR |  |  |  |
|  | IL13RA2 |  |  |  |
|  | MXI1 |  |  |  |
|  | OAS2 |  |  |  |
|  | PALLD |  |  |  |
|  | PAPPA |  |  |  |
|  | PTPN3 |  |  |  |
|  | SMAD6 |  |  |  |
|  | TACC2 |  |  |  |
|  | AFF4 |  |  |  |
|  | CAD |  |  |  |
|  | FGF9 |  |  |  |
|  | LBR |  |  |  |
|  | WIPI1 |  |  |  |
|  | ATP6V1B2 |  |  |  |
|  | FRY |  |  |  |
|  | IMPA2 |  |  |  |
|  | IQGAP1 |  |  |  |
|  | ITIH4 |  |  |  |
|  | KCNJ2 |  |  |  |
|  | KIF4A |  |  |  |
|  | LRPPRC |  |  |  |
|  | NFE2L3 |  |  |  |
|  | PICALM |  |  |  |
|  | RAD54L |  |  |  |
|  | RASA1 |  |  |  |
|  | SLC22A18 |  |  |  |
|  | ARHGAP18 |  |  |  |
|  | AVPR1A |  |  |  |
|  | B4GALT5 |  |  |  |
|  | CD48 |  |  |  |
|  | DRAM1 |  |  |  |
|  | FAM20C |  |  |  |
|  | FZD1 |  |  |  |
|  | HDLBP |  |  |  |
|  | IL3 |  |  |  |
|  | IL4R |  |  |  |
|  | ITPR2 |  |  |  |
|  | LAMP3 |  |  |  |
|  | NAV1 |  |  |  |
|  | NCF4 |  |  |  |
|  | SERPINA6 |  |  |  |
|  | TET2 |  |  |  |
|  | TRIB2 |  |  |  |
|  | COL5A3 |  |  |  |
|  | CYP51A1 |  |  |  |
|  | LPXN |  |  |  |
|  | PLA2G7 |  |  |  |
|  | PTGIS |  |  |  |
|  | S1PR3 |  |  |  |
|  | SH3BP5 |  |  |  |
|  | YPEL5 |  |  |  |
|  | CBX4 |  |  |  |
|  | DIO3 |  |  |  |
|  | DNAJC10 |  |  |  |
|  | HIF1A |  |  |  |
|  | LRIG1 |  |  |  |
|  | NTN1 |  |  |  |
|  | OAS1 |  |  |  |
|  | PDPN |  |  |  |
|  | PLXDC2 |  |  |  |
|  | SLC46A1 |  |  |  |
|  | SLC9A1 |  |  |  |
|  | STRA6 |  |  |  |
|  | ACOX2 |  |  |  |
|  | AK3 |  |  |  |
|  | ALDH3B1 |  |  |  |
|  | CIDEA |  |  |  |
|  | CITED4 |  |  |  |
|  | FAM162A |  |  |  |
|  | FZD4 |  |  |  |
|  | HIBCH |  |  |  |
|  | IFI44L |  |  |  |
|  | PTPN13 |  |  |  |
|  | RHOBTB1 |  |  |  |
|  | RPL15 |  |  |  |
|  | RTP4 |  |  |  |
|  | SUCLG2 |  |  |  |
|  | TACC1 |  |  |  |
|  | TACR1 |  |  |  |
|  | TNFSF14 |  |  |  |
|  | AOC3 |  |  |  |
|  | CYP27B1 |  |  |  |
|  | DLAT |  |  |  |
|  | MAPK10 |  |  |  |
|  | PEA15 |  |  |  |
|  | PLSCR1 |  |  |  |
|  | PPP2R1A |  |  |  |
|  | RYR1 |  |  |  |
|  | SCARB2 |  |  |  |
|  | ARAP2 |  |  |  |
|  | ARHGEF3 |  |  |  |
|  | ATOX1 |  |  |  |
|  | CSTA |  |  |  |
|  | CXXC5 |  |  |  |
|  | CYP4A1 |  |  |  |
|  | DYRK2 |  |  |  |
|  | EPHB2 |  |  |  |
|  | FASL |  |  |  |
|  | GAB1 |  |  |  |
|  | GRINA |  |  |  |
|  | HTR3A |  |  |  |
|  | INA |  |  |  |
|  | LSP1 |  |  |  |
|  | MIR34A |  |  |  |
|  | MTF1 |  |  |  |
|  | NEXN |  |  |  |
|  | PAPSS1 |  |  |  |
|  | RPS13 |  |  |  |
|  | SEMA3B |  |  |  |
|  | SRGN |  |  |  |
|  | SRSF11 |  |  |  |
|  | ADRA1A |  |  |  |
|  | CYB5B |  |  |  |
|  | LNPEP |  |  |  |
|  | LXN |  |  |  |
|  | NUAK2 |  |  |  |
|  | NUDT7 |  |  |  |
|  | PSME4 |  |  |  |
|  | SCD2 |  |  |  |
|  | SLK |  |  |  |
|  | SNX10 |  |  |  |
|  | ZBTB10 |  |  |  |
|  | ABI3BP |  |  |  |
|  | BAG2 |  |  |  |
|  | EHBP1 |  |  |  |
|  | GNL3 |  |  |  |
|  | GPR155 |  |  |  |
|  | KCNB1 |  |  |  |
|  | PLD3 |  |  |  |
|  | PTGER3 |  |  |  |
|  | RPL5 |  |  |  |
|  | SCARA5 |  |  |  |
|  | SEMA7A |  |  |  |
|  | SYNJ2 |  |  |  |
|  | TNIK |  |  |  |
|  | TRA2A |  |  |  |
|  | ZMIZ1 |  |  |  |
|  | AMD1 |  |  |  |
|  | ARHGAP24 |  |  |  |
|  | CSRP3 |  |  |  |
|  | CX3CR1 |  |  |  |
|  | EMP2 |  |  |  |
|  | FUT8 |  |  |  |
|  | GMFB |  |  |  |
|  | LIN7A |  |  |  |
|  | MAD1L1 |  |  |  |
|  | MBL2 |  |  |  |
|  | MCTP1 |  |  |  |
|  | PDGFRL |  |  |  |
|  | PSD3 |  |  |  |
|  | SST |  |  |  |
|  | TBX21 |  |  |  |
|  | TNFSF4 |  |  |  |
|  | TRIM24 |  |  |  |
|  | ABCG8 |  |  |  |
|  | ACOT7 |  |  |  |
|  | ALDOB |  |  |  |
|  | ARRDC2 |  |  |  |
|  | EXT1 |  |  |  |
|  | KANK1 |  |  |  |
|  | METAP2 |  |  |  |
|  | SIRPA |  |  |  |
|  | ATP1B2 |  |  |  |
|  | CDV3 |  |  |  |
|  | COX7B |  |  |  |
|  | FERMT1 |  |  |  |
|  | PRKAG2 |  |  |  |
|  | PRRX1 |  |  |  |
|  | RPL23 |  |  |  |
|  | SMOC2 |  |  |  |
|  | TMEM158 |  |  |  |
|  | WNT11 |  |  |  |
|  | ACAD9 |  |  |  |
|  | AGPAT3 |  |  |  |
|  | CAVIN1 |  |  |  |
|  | CCPG1 |  |  |  |
|  | CORO2A |  |  |  |
|  | GPM6B |  |  |  |
|  | H4C3 |  |  |  |
|  | IFNGR2 |  |  |  |
|  | NBN |  |  |  |
|  | NDUFB8 |  |  |  |
|  | OASL |  |  |  |
|  | ORM2 |  |  |  |
|  | PDE1A |  |  |  |
|  | PPIC |  |  |  |
|  | PSMD11 |  |  |  |
|  | RCAN2 |  |  |  |
|  | RDH11 |  |  |  |
|  | RPL24 |  |  |  |
|  | SEPTIN9 |  |  |  |
|  | SNRPD1 |  |  |  |
|  | TKFC |  |  |  |
|  | ATOH8 |  |  |  |
|  | CD53 |  |  |  |
|  | CHPT1 |  |  |  |
|  | DHRS2 |  |  |  |
|  | GABRA2 |  |  |  |
|  | HEATR1 |  |  |  |
|  | IKZF1 |  |  |  |
|  | KLF7 |  |  |  |
|  | KLKB1 |  |  |  |
|  | MCCC2 |  |  |  |
|  | PHLDB2 |  |  |  |
|  | SERPINA3 |  |  |  |
|  | SGMS1 |  |  |  |
|  | SYNM |  |  |  |
|  | CDK7 |  |  |  |
|  | DIDO1 |  |  |  |
|  | HTT |  |  |  |
|  | KMO |  |  |  |
|  | MATN2 |  |  |  |
|  | NTRK1 |  |  |  |
|  | NUDT4 |  |  |  |
|  | OXTR |  |  |  |
|  | PPBP |  |  |  |
|  | RAP1A |  |  |  |
|  | RPL21 |  |  |  |
|  | RSL1D1 |  |  |  |
|  | SERINC2 |  |  |  |
|  | SH3BGRL3 |  |  |  |
|  | SSBP1 |  |  |  |
|  | TCF12 |  |  |  |
|  | ADAMTS9 |  |  |  |
|  | AIG1 |  |  |  |
|  | APEH |  |  |  |
|  | BLNK |  |  |  |
|  | CTSZ |  |  |  |
|  | ENTPD1 |  |  |  |
|  | MAP2K5 |  |  |  |
|  | MEIS1 |  |  |  |
|  | PDLIM2 |  |  |  |
|  | PREP |  |  |  |
|  | PSMD7 |  |  |  |
|  | TP73 |  |  |  |
|  | VAMP8 |  |  |  |
|  | APOM |  |  |  |
|  | BCOR |  |  |  |
|  | BLVRA |  |  |  |
|  | CALU |  |  |  |
|  | DEFB1 |  |  |  |
|  | EPHA3 |  |  |  |
|  | FAM210B |  |  |  |
|  | FUCA1 |  |  |  |
|  | NKX3-1 |  |  |  |
|  | PAM |  |  |  |
|  | PDZD2 |  |  |  |
|  | PLEK2 |  |  |  |
|  | RAD51B |  |  |  |
|  | SLC16A7 |  |  |  |
|  | SLCO3A1 |  |  |  |
|  | TXN2 |  |  |  |
|  | UBE2I |  |  |  |
|  | YPEL3 |  |  |  |
|  | BCKDHB |  |  |  |
|  | MYCBP2 |  |  |  |
|  | MYO1D |  |  |  |
|  | PRUNE2 |  |  |  |
|  | PTPRE |  |  |  |
|  | SPINT2 |  |  |  |
|  | SSBP2 |  |  |  |
|  | TAP2 |  |  |  |
|  | ADGRV1 |  |  |  |
|  | AMACR |  |  |  |
|  | CD22 |  |  |  |
|  | CHD7 |  |  |  |
|  | DAP |  |  |  |
|  | DGKA |  |  |  |
|  | EOMES |  |  |  |
|  | HNF1A |  |  |  |
|  | HS3ST1 |  |  |  |
|  | HS3ST3B1 |  |  |  |
|  | PCTP |  |  |  |
|  | PFN1 |  |  |  |
|  | PPP2R1B |  |  |  |
|  | RAB1A |  |  |  |
|  | RAB8B |  |  |  |
|  | RAPGEF2 |  |  |  |
|  | SEMA3A |  |  |  |
|  | SFTPB |  |  |  |
|  | TANK |  |  |  |
|  | TMBIM1 |  |  |  |
|  | WNT7B |  |  |  |
|  | CFL2 |  |  |  |
|  | COX6A2 |  |  |  |
|  | DPP7 |  |  |  |
|  | FLRT2 |  |  |  |
|  | GYS1 |  |  |  |
|  | IRF4 |  |  |  |
|  | PEMT |  |  |  |
|  | ROR1 |  |  |  |
|  | RPL22L1 |  |  |  |
|  | SERPINF2 |  |  |  |
|  | SLC22A4 |  |  |  |
|  | AKAP8L |  |  |  |
|  | BAIAP2L1 |  |  |  |
|  | CCL19 |  |  |  |
|  | COL13A1 |  |  |  |
|  | COQ8A |  |  |  |
|  | DNAJA4 |  |  |  |
|  | EML4 |  |  |  |
|  | EPPK1 |  |  |  |
|  | FAM111A |  |  |  |
|  | INF2 |  |  |  |
|  | PPA2 |  |  |  |
|  | RAB30 |  |  |  |
|  | SERPINB5 |  |  |  |
|  | CADPS2 |  |  |  |
|  | CARD10 |  |  |  |
|  | CLMP |  |  |  |
|  | DSG2 |  |  |  |
|  | ENDOG |  |  |  |
|  | EPB41L5 |  |  |  |
|  | EPSTI1 |  |  |  |
|  | FBP2 |  |  |  |
|  | FXN |  |  |  |
|  | GRK3 |  |  |  |
|  | SLC2A6 |  |  |  |
|  | SLC6A13 |  |  |  |
|  | SP7 |  |  |  |
|  | SYT12 |  |  |  |
|  | TICAM1 |  |  |  |
|  | ADAM12 |  |  |  |
|  | ATP5F1C |  |  |  |
|  | BPGM |  |  |  |
|  | CACNA1D |  |  |  |
|  | CDK14 |  |  |  |
|  | CTHRC1 |  |  |  |
|  | FNBP4 |  |  |  |
|  | GSPT1 |  |  |  |
|  | MAGED2 |  |  |  |
|  | MMUT |  |  |  |
|  | RASL11A |  |  |  |
|  | REV1 |  |  |  |
|  | S1PR2 |  |  |  |
|  | SRP72 |  |  |  |
|  | TPCN1 |  |  |  |
|  | TUBA8 |  |  |  |
|  | VEPH1 |  |  |  |
|  | ZMYM3 |  |  |  |
|  | ABTB2 |  |  |  |
|  | ACY1 |  |  |  |
|  | CD109 |  |  |  |
|  | IER5L |  |  |  |
|  | NTN4 |  |  |  |
|  | QKI |  |  |  |
|  | RPS27A |  |  |  |
|  | ST3GAL1 |  |  |  |
|  | TFE3 |  |  |  |
|  | UBE2B |  |  |  |
|  | ACAA1 |  |  |  |
|  | ADNP |  |  |  |
|  | AOC1 |  |  |  |
|  | BZW1 |  |  |  |
|  | COX6C |  |  |  |
|  | GALNT10 |  |  |  |
|  | GOSR2 |  |  |  |
|  | GRAMD2B |  |  |  |
|  | ILF3 |  |  |  |
|  | IRF5 |  |  |  |
|  | NACC2 |  |  |  |
|  | NUMB |  |  |  |
|  | PDK3 |  |  |  |
|  | RAI1 |  |  |  |
|  | RTN4RL1 |  |  |  |
|  | SLC43A2 |  |  |  |
|  | STAU2 |  |  |  |
|  | TMED2 |  |  |  |
|  | TTN |  |  |  |
|  | C8B |  |  |  |
|  | DTX4 |  |  |  |
|  | ETHE1 |  |  |  |
|  | EXOSC2 |  |  |  |
|  | FTO |  |  |  |
|  | HNRNPR |  |  |  |
|  | MACROH2A1 |  |  |  |
|  | MDN1 |  |  |  |
|  | NUP98 |  |  |  |
|  | PRRG4 |  |  |  |
|  | APOA5 |  |  |  |
|  | ARHGEF26 |  |  |  |
|  | CAMKK2 |  |  |  |
|  | CCN4 |  |  |  |
|  | DCC |  |  |  |
|  | FRMD4B |  |  |  |
|  | HR |  |  |  |
|  | JCHAIN |  |  |  |
|  | KLF3 |  |  |  |
|  | MMP11 |  |  |  |
|  | MMP19 |  |  |  |
|  | NDUFB6 |  |  |  |
|  | NFATC2 |  |  |  |
|  | PDZRN3 |  |  |  |
|  | PGAM2 |  |  |  |
|  | RIN2 |  |  |  |
|  | SALL1 |  |  |  |
|  | SNRK |  |  |  |
|  | SRPX |  |  |  |
|  | STAMBPL1 |  |  |  |
|  | SUCLG1 |  |  |  |
|  | TNFSF15 |  |  |  |
|  | TOR3A |  |  |  |
|  | TRPM4 |  |  |  |
|  | WASF2 |  |  |  |
|  | AJUBA |  |  |  |
|  | ATP6V0D1 |  |  |  |
|  | DDT |  |  |  |
|  | ENDOD1 |  |  |  |
|  | EVI2A |  |  |  |
|  | FGF10 |  |  |  |
|  | GCKR |  |  |  |
|  | GPRC5B |  |  |  |
|  | HUWE1 |  |  |  |
|  | PARPBP |  |  |  |
|  | PKP4 |  |  |  |
|  | PLA2G6 |  |  |  |
|  | PMS1 |  |  |  |
|  | POLR2A |  |  |  |
|  | PTBP3 |  |  |  |
|  | RPS21 |  |  |  |
|  | SEMA6A |  |  |  |
|  | SSB |  |  |  |
|  | TIMP4 |  |  |  |
|  | TMEM140 |  |  |  |
|  | ADRB3 |  |  |  |
|  | CYTIP |  |  |  |
|  | DEDD2 |  |  |  |
|  | GFOD1 |  |  |  |
|  | PLEKHA2 |  |  |  |
|  | PRAG1 |  |  |  |
|  | SLCO4C1 |  |  |  |
|  | ACAN |  |  |  |
|  | ACP3 |  |  |  |
|  | AP1S2 |  |  |  |
|  | CCR3 |  |  |  |
|  | CHD9 |  |  |  |
|  | CTPS2 |  |  |  |
|  | DLGAP4 |  |  |  |
|  | EP400 |  |  |  |
|  | FNBP1L |  |  |  |
|  | GFRA2 |  |  |  |
|  | GPR137B |  |  |  |
|  | GPR183 |  |  |  |
|  | GRHPR |  |  |  |
|  | KYAT3 |  |  |  |
|  | LRRN3 |  |  |  |
|  | MEX3B |  |  |  |
|  | PLA2G4C |  |  |  |
|  | PRKAB2 |  |  |  |
|  | PTGFR |  |  |  |
|  | RRAGC |  |  |  |
|  | SAMD9 |  |  |  |
|  | SCN8A |  |  |  |
|  | SEMA4D |  |  |  |
|  | SEPHS2 |  |  |  |
|  | SERINC3 |  |  |  |
|  | SLC51B |  |  |  |
|  | ALDH8A1 |  |  |  |
|  | APBB1IP |  |  |  |
|  | ARID1B |  |  |  |
|  | ARID2 |  |  |  |
|  | CALML4 |  |  |  |
|  | CLDN7 |  |  |  |
|  | FADS3 |  |  |  |
|  | GYG1 |  |  |  |
|  | HIVEP1 |  |  |  |
|  | HIVEP3 |  |  |  |
|  | IL17RB |  |  |  |
|  | IRAK3 |  |  |  |
|  | KCND2 |  |  |  |
|  | MICAL1 |  |  |  |
|  | PAG1 |  |  |  |
|  | SLC10A2 |  |  |  |
|  | SLC25A36 |  |  |  |
|  | SUB1 |  |  |  |
|  | TCIM |  |  |  |
|  | TMTC4 |  |  |  |
|  | UQCRC1 |  |  |  |
|  | CAPN5 |  |  |  |
|  | CBX1 |  |  |  |
|  | CYB561 |  |  |  |
|  | CYSTM1 |  |  |  |
|  | DECR2 |  |  |  |
|  | EFNA5 |  |  |  |
|  | IFI47 |  |  |  |
|  | MADD |  |  |  |
|  | NDUFA4 |  |  |  |
|  | OSR2 |  |  |  |
|  | PRR5L |  |  |  |
|  | THYN1 |  |  |  |
|  | TNRC6B |  |  |  |
|  | VIPR1 |  |  |  |
|  | WNT5B |  |  |  |
|  | AVPI1 |  |  |  |
|  | CTTNBP2NL |  |  |  |
|  | FABP6 |  |  |  |
|  | FBXW7 |  |  |  |
|  | GAS2 |  |  |  |
|  | MYCL |  |  |  |
|  | PLEKHA5 |  |  |  |
|  | RAB20 |  |  |  |
|  | RDH10 |  |  |  |
|  | SFXN3 |  |  |  |
|  | SLC23A2 |  |  |  |
|  | SLC31A2 |  |  |  |
|  | ABCA3 |  |  |  |
|  | ANK1 |  |  |  |
|  | CHSY1 |  |  |  |
|  | GFRA1 |  |  |  |
|  | HIP1R |  |  |  |
|  | HLA-A |  |  |  |
|  | JARID2 |  |  |  |
|  | KAZN |  |  |  |
|  | LOXL4 |  |  |  |
|  | OGFRL1 |  |  |  |
|  | PGPEP1 |  |  |  |
|  | PGRMC2 |  |  |  |
|  | PXMP4 |  |  |  |
|  | RIOK3 |  |  |  |
|  | S100A1 |  |  |  |
|  | TK2 |  |  |  |
|  | TMOD2 |  |  |  |
|  | TRIM14 |  |  |  |
|  | VAPB |  |  |  |
|  | ADGRG2 |  |  |  |
|  | CDC42EP2 |  |  |  |
|  | MAP3K4 |  |  |  |
|  | PPP3R1 |  |  |  |
|  | RASA3 |  |  |  |
|  | RPTOR |  |  |  |
|  | SEC63 |  |  |  |
|  | SIN3A |  |  |  |
|  | TMTC2 |  |  |  |
|  | TRPV2 |  |  |  |
|  | UBAP2L |  |  |  |
|  | UBB |  |  |  |
|  | EMILIN1 |  |  |  |
|  | EVA1A |  |  |  |
|  | FARSB |  |  |  |
|  | HTR7 |  |  |  |
|  | IGF2BP1 |  |  |  |
|  | MAP1A |  |  |  |
|  | NONO |  |  |  |
|  | PIK3C2B |  |  |  |
|  | SP100 |  |  |  |
|  | STXBP6 |  |  |  |
|  | UQCRQ |  |  |  |
|  | ARID5A |  |  |  |
|  | ATP5PB |  |  |  |
|  | CCS |  |  |  |
|  | CGN |  |  |  |
|  | MXRA8 |  |  |  |
|  | RNF125 |  |  |  |
|  | SCG5 |  |  |  |
|  | SERINC1 |  |  |  |
|  | ATP6V0B |  |  |  |
|  | CMTM3 |  |  |  |
|  | EDIL3 |  |  |  |
|  | FANCC |  |  |  |
|  | HSPB6 |  |  |  |
|  | LRRC8C |  |  |  |
|  | MYO6 |  |  |  |
|  | PKDCC |  |  |  |
|  | RBM33 |  |  |  |
|  | SERTAD2 |  |  |  |
|  | SNRPF |  |  |  |
|  | SORCS2 |  |  |  |
|  | TMCC3 |  |  |  |
|  | TMEM47 |  |  |  |
|  | TRMT61A |  |  |  |
|  | CD302 |  |  |  |
|  | DAGLB |  |  |  |
|  | GRM1 |  |  |  |
|  | LHFPL2 |  |  |  |
|  | MBD2 |  |  |  |
|  | PALM2AKAP2 |  |  |  |
|  | PIEZO1 |  |  |  |
|  | POLH |  |  |  |
|  | PRMT3 |  |  |  |
|  | REV3L |  |  |  |
|  | SAP18 |  |  |  |
|  | SH2D4A |  |  |  |
|  | SULT2B1 |  |  |  |
|  | ADRA1D |  |  |  |
|  | ALPK1 |  |  |  |
|  | ASCC3 |  |  |  |
|  | COX6A1 |  |  |  |
|  | CRYZ |  |  |  |
|  | CYB5R1 |  |  |  |
|  | FIBIN |  |  |  |
|  | FKTN |  |  |  |
|  | GCGR |  |  |  |
|  | LUC7L |  |  |  |
|  | MRPS27 |  |  |  |
|  | PARM1 |  |  |  |
|  | PSEN2 |  |  |  |
|  | PXN |  |  |  |
|  | SBK1 |  |  |  |
|  | TTC39C |  |  |  |
|  | APPL1 |  |  |  |
|  | COL4A6 |  |  |  |
|  | DYNC1I1 |  |  |  |
|  | ELK4 |  |  |  |
|  | GYS2 |  |  |  |
|  | HIF3A |  |  |  |
|  | KRT1 |  |  |  |
|  | LPCAT3 |  |  |  |
|  | PHLDB1 |  |  |  |
|  | RPS28 |  |  |  |
|  | SCAMP1 |  |  |  |
|  | SLC1A6 |  |  |  |
|  | SLC48A1 |  |  |  |
|  | TCEAL8 |  |  |  |
|  | TIMM8B |  |  |  |
|  | ATP5ME |  |  |  |
|  | CA1 |  |  |  |
|  | CES1D |  |  |  |
|  | CPZ |  |  |  |
|  | CSF2RA |  |  |  |
|  | DAO |  |  |  |
|  | FLVCR2 |  |  |  |
|  | KDM6A |  |  |  |
|  | MARVELD1 |  |  |  |
|  | NAGA |  |  |  |
|  | OMD |  |  |  |
|  | PIEZO2 |  |  |  |
|  | PPP1R1A |  |  |  |
|  | PRG4 |  |  |  |
|  | PRKD1 |  |  |  |
|  | PUS7 |  |  |  |
|  | RAB3D |  |  |  |
|  | SIDT2 |  |  |  |
|  | SLC27A5 |  |  |  |
|  | SLC39A11 |  |  |  |
|  | SYT1 |  |  |  |
|  | TDG |  |  |  |
|  | VAMP1 |  |  |  |
|  | CELSR2 |  |  |  |
|  | DYNLT3 |  |  |  |
|  | PTBP2 |  |  |  |
|  | SLC16A14 |  |  |  |
|  | TNFSF13B |  |  |  |
|  | TRAK2 |  |  |  |
|  | TRAM2 |  |  |  |
|  | ATP5MG |  |  |  |
|  | CDS2 |  |  |  |
|  | DPYS |  |  |  |
|  | EIF1AX |  |  |  |
|  | GOLM1 |  |  |  |
|  | HAUS4 |  |  |  |
|  | IMMT |  |  |  |
|  | MTOR |  |  |  |
|  | NDUFV1 |  |  |  |
|  | PHIP |  |  |  |
|  | PLIN5 |  |  |  |
|  | POLR1C |  |  |  |
|  | SLC25A30 |  |  |  |
|  | SYT7 |  |  |  |
|  | TANC2 |  |  |  |
|  | TRIOBP |  |  |  |
|  | VPS13A |  |  |  |
|  | AP1B1 |  |  |  |
|  | C1QTNF1 |  |  |  |
|  | CLEC2D |  |  |  |
|  | EPN2 |  |  |  |
|  | FBXO3 |  |  |  |
|  | FNDC1 |  |  |  |
|  | GBP3 |  |  |  |
|  | IFI16 |  |  |  |
|  | IL17RD |  |  |  |
|  | IL18R1 |  |  |  |
|  | KCNK2 |  |  |  |
|  | LAT |  |  |  |
|  | LIMK2 |  |  |  |
|  | MBTPS1 |  |  |  |
|  | MOGAT1 |  |  |  |
|  | NDUFA12 |  |  |  |
|  | PANX1 |  |  |  |
|  | PIM2 |  |  |  |
|  | PXDC1 |  |  |  |
|  | STARD5 |  |  |  |
|  | TRIM22 |  |  |  |
|  | TRPS1 |  |  |  |
|  | ADCY9 |  |  |  |
|  | CISD1 |  |  |  |
|  | HINT3 |  |  |  |
|  | ITM2A |  |  |  |
|  | NDUFAF4 |  |  |  |
|  | NRN1 |  |  |  |
|  | RBM15 |  |  |  |
|  | RPIA |  |  |  |
|  | SNRPN |  |  |  |
|  | ADORA3 |  |  |  |
|  | ALYREF |  |  |  |
|  | ANO3 |  |  |  |
|  | CAPNS1 |  |  |  |
|  | COX6B2 |  |  |  |
|  | DDHD1 |  |  |  |
|  | KBTBD11 |  |  |  |
|  | OTUD1 |  |  |  |
|  | SFRP4 |  |  |  |
|  | STK38L |  |  |  |
|  | TLR7 |  |  |  |
|  | TMEM86A |  |  |  |
|  | TNFA |  |  |  |
|  | CPSF6 |  |  |  |
|  | DPP9 |  |  |  |
|  | EXOC3 |  |  |  |
|  | MAP3K12 |  |  |  |
|  | MYEF2 |  |  |  |
|  | PTGR2 |  |  |  |
|  | RFK |  |  |  |
|  | RNF24 |  |  |  |
|  | SETD2 |  |  |  |
|  | STIM1 |  |  |  |
|  | TIMM9 |  |  |  |
|  | WDR4 |  |  |  |
|  | AQP8 |  |  |  |
|  | CCNL2 |  |  |  |
|  | DMKN |  |  |  |
|  | DTX3L |  |  |  |
|  | FCGBP |  |  |  |
|  | NAT10 |  |  |  |
|  | NEURL3 |  |  |  |
|  | NTM |  |  |  |
|  | PLA2G5 |  |  |  |
|  | PRMT2 |  |  |  |
|  | PTGES2 |  |  |  |
|  | TANC1 |  |  |  |
|  | ATG10 |  |  |  |
|  | CAMTA1 |  |  |  |
|  | DOCK5 |  |  |  |
|  | LDLRAD4 |  |  |  |
|  | PEX2 |  |  |  |
|  | PTPN12 |  |  |  |
|  | SETD5 |  |  |  |
|  | SLTM |  |  |  |
|  | STX12 |  |  |  |
|  | TCF7L1 |  |  |  |
|  | TENT5B |  |  |  |
|  | TM9SF3 |  |  |  |
|  | USP13 |  |  |  |
|  | ANGPTL3 |  |  |  |
|  | AP2A1 |  |  |  |
|  | CACNB4 |  |  |  |
|  | CD28 |  |  |  |
|  | CHST2 |  |  |  |
|  | CNKSR3 |  |  |  |
|  | IKZF2 |  |  |  |
|  | KCNN2 |  |  |  |
|  | PAN2 |  |  |  |
|  | POPDC2 |  |  |  |
|  | PSMA3 |  |  |  |
|  | RPL23A |  |  |  |
|  | TSPAN3 |  |  |  |
|  | CHD1 |  |  |  |
|  | COL8A2 |  |  |  |
|  | GALNT15 |  |  |  |
|  | HSD3B7 |  |  |  |
|  | IL2RB |  |  |  |
|  | KLHL29 |  |  |  |
|  | MAG |  |  |  |
|  | NTHL1 |  |  |  |
|  | OSER1 |  |  |  |
|  | RICTOR |  |  |  |
|  | TENM2 |  |  |  |
|  | U2SURP |  |  |  |
|  | WDFY3 |  |  |  |
|  | ABLIM2 |  |  |  |
|  | ATP6V1H |  |  |  |
|  | CIAPIN1 |  |  |  |
|  | CPPED1 |  |  |  |
|  | DGKH |  |  |  |
|  | EGFL6 |  |  |  |
|  | F8 |  |  |  |
|  | FCGR3A |  |  |  |
|  | FILIP1 |  |  |  |
|  | ITIH1 |  |  |  |
|  | IYD |  |  |  |
|  | MAPK11 |  |  |  |
|  | NDUFB3 |  |  |  |
|  | NEO1 |  |  |  |
|  | PPP1R16B |  |  |  |
|  | PREB |  |  |  |
|  | PRKG2 |  |  |  |
|  | PTPN18 |  |  |  |
|  | RASD2 |  |  |  |
|  | SLC16A9 |  |  |  |
|  | SRD5A2 |  |  |  |
|  | TEAD4 |  |  |  |
|  | CSPP1 |  |  |  |
|  | CXCR1 |  |  |  |
|  | ESR2 |  |  |  |
|  | GMDS |  |  |  |
|  | KCNJ11 |  |  |  |
|  | KHSRP |  |  |  |
|  | LARP6 |  |  |  |
|  | MEOX2 |  |  |  |
|  | NSG1 |  |  |  |
|  | SECISBP2L |  |  |  |
|  | TMEM50B |  |  |  |
|  | VPS13B |  |  |  |
|  | ATP6V0D2 |  |  |  |
|  | CETN3 |  |  |  |
|  | CILP |  |  |  |
|  | DCUN1D4 |  |  |  |
|  | DKK2 |  |  |  |
|  | FAM149A |  |  |  |
|  | FMO4 |  |  |  |
|  | FOXP4 |  |  |  |
|  | KITL |  |  |  |
|  | MFAP3L |  |  |  |
|  | MRPL20 |  |  |  |
|  | NECTIN4 |  |  |  |
|  | NOP16 |  |  |  |
|  | NR1I3 |  |  |  |
|  | NRARP |  |  |  |
|  | NUDT21 |  |  |  |
|  | PAK3 |  |  |  |
|  | PEX19 |  |  |  |
|  | S1PR5 |  |  |  |
|  | TLK1 |  |  |  |
|  | TMCC2 |  |  |  |
|  | AFDN |  |  |  |
|  | ARHGEF28 |  |  |  |
|  | CHIC2 |  |  |  |
|  | EBI3 |  |  |  |
|  | IL2 |  |  |  |
|  | NME3 |  |  |  |
|  | PTPN6 |  |  |  |
|  | RIT1 |  |  |  |
|  | SLC12A3 |  |  |  |
|  | SMYD3 |  |  |  |
|  | SPTSSA |  |  |  |
|  | TSPAN33 |  |  |  |
|  | ZMYM2 |  |  |  |
|  | ACVR2B |  |  |  |
|  | AP3S1 |  |  |  |
|  | CARD11 |  |  |  |
|  | COX7A1 |  |  |  |
|  | CRLS1 |  |  |  |
|  | CTCF |  |  |  |
|  | DGKG |  |  |  |
|  | FGF5 |  |  |  |
|  | FHOD1 |  |  |  |
|  | FOXJ1 |  |  |  |
|  | GOLIM4 |  |  |  |
|  | HAND2 |  |  |  |
|  | IDH3B |  |  |  |
|  | IDH3G |  |  |  |
|  | MRPL33 |  |  |  |
|  | MS4A1 |  |  |  |
|  | NOTCH4 |  |  |  |
|  | P3H2 |  |  |  |
|  | PLCXD2 |  |  |  |
|  | PPCS |  |  |  |
|  | REC8 |  |  |  |
|  | STON2 |  |  |  |
|  | TCF25 |  |  |  |
|  | THEM6 |  |  |  |
|  | TRAPPC6A |  |  |  |
|  | TRPV6 |  |  |  |
|  | UQCR11 |  |  |  |
|  | VGLL3 |  |  |  |
|  | VWA1 |  |  |  |
|  | BRD8 |  |  |  |
|  | GEMIN5 |  |  |  |
|  | HDAC8 |  |  |  |
|  | LYZ2 |  |  |  |
|  | PCDH18 |  |  |  |
|  | SCAF11 |  |  |  |
|  | SIRT4 |  |  |  |
|  | ZC3H13 |  |  |  |
|  | ZFYVE16 |  |  |  |
|  | AAK1 |  |  |  |
|  | ANXA10 |  |  |  |
|  | DAP3 |  |  |  |
|  | DNAJC6 |  |  |  |
|  | EEIG1 |  |  |  |
|  | ERP27 |  |  |  |
|  | IVL |  |  |  |
|  | PTPN22 |  |  |  |
|  | RGS20 |  |  |  |
|  | SGPP2 |  |  |  |
|  | SPNS2 |  |  |  |
|  | TRAFD1 |  |  |  |
|  | VWA5A |  |  |  |
|  | WTAP |  |  |  |
|  | ADGRD1 |  |  |  |
|  | FAR1 |  |  |  |
|  | GNL3L |  |  |  |
|  | HGS |  |  |  |
|  | KAT14 |  |  |  |
|  | LACC1 |  |  |  |
|  | MGARP |  |  |  |
|  | SERPINA3N |  |  |  |
|  | SNRPA1 |  |  |  |
|  | SNX5 |  |  |  |
|  | TMTC3 |  |  |  |
|  | TRIM35 |  |  |  |
|  | ARHGAP23 |  |  |  |
|  | CSNK1G1 |  |  |  |
|  | DLGAP1 |  |  |  |
|  | HIPK3 |  |  |  |
|  | MLYCD |  |  |  |
|  | MPZ |  |  |  |
|  | PGAM5 |  |  |  |
|  | TLR9 |  |  |  |
|  | ADPGK |  |  |  |
|  | CASP1 |  |  |  |
|  | FITM2 |  |  |  |
|  | FKBP1B |  |  |  |
|  | H4C9 |  |  |  |
|  | HERC4 |  |  |  |
|  | HPDL |  |  |  |
|  | HYCC2 |  |  |  |
|  | IL12RB2 |  |  |  |
|  | ITPRIPL2 |  |  |  |
|  | LCORL |  |  |  |
|  | MLLT10 |  |  |  |
|  | OSBPL8 |  |  |  |
|  | PHF11 |  |  |  |
|  | PMPCA |  |  |  |
|  | SAC3D1 |  |  |  |
|  | TTC17 |  |  |  |
|  | USP53 |  |  |  |
|  | UTP15 |  |  |  |
|  | COX15 |  |  |  |
|  | EDN3 |  |  |  |
|  | GDPD1 |  |  |  |
|  | HPN |  |  |  |
|  | MRPL50 |  |  |  |
|  | N4BP1 |  |  |  |
|  | NPPC |  |  |  |
|  | NTSR1 |  |  |  |
|  | PPM1G |  |  |  |
|  | PTGER1 |  |  |  |
|  | TEAD1 |  |  |  |
|  | CC2D2A |  |  |  |
|  | CD2BP2 |  |  |  |
|  | COMMD10 |  |  |  |
|  | CXCL6 |  |  |  |
|  | DUOX1 |  |  |  |
|  | EPC1 |  |  |  |
|  | GNA15 |  |  |  |
|  | IRF2BPL |  |  |  |
|  | NFS1 |  |  |  |
|  | RAB7B |  |  |  |
|  | RUFY3 |  |  |  |
|  | SEMA3G |  |  |  |
|  | SFXN4 |  |  |  |
|  | STOX2 |  |  |  |
|  | UQCR10 |  |  |  |
|  | USP34 |  |  |  |
|  | VSIR |  |  |  |
|  | DPP3 |  |  |  |
|  | FAM20A |  |  |  |
|  | MCCC1 |  |  |  |
|  | QPRT |  |  |  |
|  | SSTR3 |  |  |  |
|  | TMEM14A |  |  |  |
|  | BPNT2 |  |  |  |
|  | CDK16 |  |  |  |
|  | CLP1 |  |  |  |
|  | ERH |  |  |  |
|  | KIRREL3 |  |  |  |
|  | LSM5 |  |  |  |
|  | MGAT2 |  |  |  |
|  | MXRA7 |  |  |  |
|  | PCGF5 |  |  |  |
|  | PPP4R2 |  |  |  |
|  | SAT2 |  |  |  |
|  | SDAD1 |  |  |  |
|  | TSEN2 |  |  |  |
|  | WDR36 |  |  |  |
|  | WNT6 |  |  |  |
|  | CYP46A1 |  |  |  |
|  | FAM117B |  |  |  |
|  | FUT1 |  |  |  |
|  | MAN1C1 |  |  |  |
|  | NSD3 |  |  |  |
|  | SDHD |  |  |  |
|  | TET3 |  |  |  |
|  | BMAL2 |  |  |  |
|  | FGF23 |  |  |  |
|  | HIPK1 |  |  |  |
|  | HLA-DMB |  |  |  |
|  | MRPS22 |  |  |  |
|  | P2RX5 |  |  |  |
|  | RBM17 |  |  |  |
|  | RIC8A |  |  |  |
|  | SASH3 |  |  |  |
|  | SLC3A1 |  |  |  |
|  | SNN |  |  |  |
|  | STXBP2 |  |  |  |
|  | TIRAP |  |  |  |
|  | TRAF5 |  |  |  |
|  | UGT1A4 |  |  |  |
|  | VASH2 |  |  |  |
|  | BICD1 |  |  |  |
|  | HCN2 |  |  |  |
|  | HOOK3 |  |  |  |
|  | LIPH |  |  |  |
|  | MDM1 |  |  |  |
|  | MRPL13 |  |  |  |
|  | MRPL49 |  |  |  |
|  | NAP1L5 |  |  |  |
|  | PCDH10 |  |  |  |
|  | PCNX1 |  |  |  |
|  | RDH5 |  |  |  |
|  | SALL2 |  |  |  |
|  | SMARCA1 |  |  |  |
|  | TBL1X |  |  |  |
|  | ULK2 |  |  |  |
|  | USPL1 |  |  |  |
|  | ZBTB1 |  |  |  |
|  | EIF2B4 |  |  |  |
|  | FARS2 |  |  |  |
|  | HCN4 |  |  |  |
|  | IKBKE |  |  |  |
|  | RNF157 |  |  |  |
|  | SNX4 |  |  |  |
|  | ATP10A |  |  |  |
|  | BIRC5 |  |  |  |
|  | CABIN1 |  |  |  |
|  | CALN1 |  |  |  |
|  | CDC42BPB |  |  |  |
|  | CPVL |  |  |  |
|  | CUL4B |  |  |  |
|  | GABARAP |  |  |  |
|  | ISCA1 |  |  |  |
|  | ITPK1 |  |  |  |
|  | MIX23 |  |  |  |
|  | MRAP |  |  |  |
|  | MYCBP |  |  |  |
|  | NOD2 |  |  |  |
|  | PABPC1L |  |  |  |
|  | PDE8A |  |  |  |
|  | PHC2 |  |  |  |
|  | PHC3 |  |  |  |
|  | PRRX2 |  |  |  |
|  | RAD52 |  |  |  |
|  | REEP3 |  |  |  |
|  | RELL1 |  |  |  |
|  | SYTL5 |  |  |  |
|  | TWIST2 |  |  |  |
|  | ZC3H11A |  |  |  |
|  | ZRANB3 |  |  |  |
|  | BANK1 |  |  |  |
|  | SH2D3C |  |  |  |
|  | SUSD3 |  |  |  |
|  | AADAC |  |  |  |
|  | AAMP |  |  |  |
|  | ACAA1B |  |  |  |
|  | ACBD5 |  |  |  |
|  | CLN8 |  |  |  |
|  | CMSS1 |  |  |  |
|  | COX6B1 |  |  |  |
|  | HELZ |  |  |  |
|  | IFT70B |  |  |  |
|  | KAZALD1 |  |  |  |
|  | MRPL1 |  |  |  |
|  | MRPS7 |  |  |  |
|  | MTRR |  |  |  |
|  | ND4 |  |  |  |
|  | PI4K2B |  |  |  |
|  | PRR7 |  |  |  |
|  | PSMG1 |  |  |  |
|  | RCOR2 |  |  |  |
|  | ROS1 |  |  |  |
|  | SCFD2 |  |  |  |
|  | TMT1B |  |  |  |
|  | TPK1 |  |  |  |
|  | VAMP4 |  |  |  |
|  | BCL7A |  |  |  |
|  | NR3C1 |  |  |  |
|  | ABHD1 |  |  |  |
|  | ABHD15 |  |  |  |
|  | ARSA |  |  |  |
|  | CCDC136 |  |  |  |
|  | ELANE |  |  |  |
|  | GCSH |  |  |  |
|  | GPAT4 |  |  |  |
|  | GXYLT1 |  |  |  |
|  | IGDCC4 |  |  |  |
|  | KCNG1 |  |  |  |
|  | LIG3 |  |  |  |
|  | MOSPD1 |  |  |  |
|  | NDUFS2 |  |  |  |
|  | NHLRC2 |  |  |  |
|  | NPR1 |  |  |  |
|  | PGGT1B |  |  |  |
|  | RAPGEF6 |  |  |  |
|  | RIOK2 |  |  |  |
|  | RLF |  |  |  |
|  | SERPINA3K |  |  |  |
|  | SETD1B |  |  |  |
|  | SLAMF7 |  |  |  |
|  | TSPAN1 |  |  |  |
|  | WAPL |  |  |  |
|  | WBP1 |  |  |  |
|  | XPO6 |  |  |  |
|  | ZC3H12C |  |  |  |
|  | ZFC3H1 |  |  |  |
|  | ZNFX1 |  |  |  |
|  | HLX |  |  |  |
|  | HTR4 |  |  |  |
|  | MRPL18 |  |  |  |
|  | MYBPC3 |  |  |  |
|  | NDUFC2 |  |  |  |
|  | BDNF |  |  |  |
|  | CHRNE |  |  |  |
|  | CYP4A2 |  |  |  |
|  | GNG4 |  |  |  |
|  | HSF2 |  |  |  |
|  | MEGF10 |  |  |  |
|  | MEIG1 |  |  |  |
|  | MRPL14 |  |  |  |
|  | NDUFA6 |  |  |  |
|  | UST |  |  |  |
|  | ZBED5 |  |  |  |
|  | AAMDC |  |  |  |
|  | AXIN1 |  |  |  |
|  | BAG5 |  |  |  |
|  | CAR2 |  |  |  |
|  | CYP4F2 |  |  |  |
|  | FAM78A |  |  |  |
|  | GPD1L |  |  |  |
|  | H3C4 |  |  |  |
|  | ING5 |  |  |  |
|  | LDB2 |  |  |  |
|  | MYO9A |  |  |  |
|  | PRPF6 |  |  |  |
|  | RHOV |  |  |  |
|  | RO60 |  |  |  |
|  | SASS6 |  |  |  |
|  | SMG1 |  |  |  |
|  | SYNC |  |  |  |
|  | TLK2 |  |  |  |
|  | TRIM26 |  |  |  |
|  | TRIP10 |  |  |  |
|  | VEZF1 |  |  |  |
|  | GPR55 |  |  |  |
|  | GRPEL2 |  |  |  |
|  | NRAP |  |  |  |
|  | OASL2 |  |  |  |
|  | OSBPL5 |  |  |  |
|  | RB1 |  |  |  |
|  | RTTN |  |  |  |
|  | USP12 |  |  |  |
|  | ALG14 |  |  |  |
|  | ATE1 |  |  |  |
|  | CACNB3 |  |  |  |
|  | CNOT4 |  |  |  |
|  | DVL2 |  |  |  |
|  | H2AC20 |  |  |  |
|  | HM13 |  |  |  |
|  | HOOK1 |  |  |  |
|  | IFT74 |  |  |  |
|  | KLB |  |  |  |
|  | METTL9 |  |  |  |
|  | MRPS17 |  |  |  |
|  | MSH3 |  |  |  |
|  | NDUFS6 |  |  |  |
|  | NUBP1 |  |  |  |
|  | PARG |  |  |  |
|  | PRRC2B |  |  |  |
|  | RIOX2 |  |  |  |
|  | SMURF1 |  |  |  |
|  | THBS3 |  |  |  |
|  | TSPYL2 |  |  |  |
|  | NPC1L1 |  |  |  |
|  | PYROXD2 |  |  |  |
|  | AQP2 |  |  |  |
|  | CGRRF1 |  |  |  |
|  | CLCA2 |  |  |  |
|  | CUTC |  |  |  |
|  | CYRIA |  |  |  |
|  | DOK4 |  |  |  |
|  | DOK7 |  |  |  |
|  | ECM2 |  |  |  |
|  | EFS |  |  |  |
|  | GBP5 |  |  |  |
|  | HYLS1 |  |  |  |
|  | IPCEF1 |  |  |  |
|  | KANSL2 |  |  |  |
|  | MAN2B2 |  |  |  |
|  | MLF2 |  |  |  |
|  | MRPS28 |  |  |  |
|  | MST1R |  |  |  |
|  | NDUFB4 |  |  |  |
|  | PLEKHG1 |  |  |  |
|  | PTRH1 |  |  |  |
|  | RLIM |  |  |  |
|  | SLC22A12 |  |  |  |
|  | SLC35E1 |  |  |  |
|  | TBCEL |  |  |  |
|  | TRPC4 |  |  |  |
|  | TSFM |  |  |  |
|  | TUB |  |  |  |
|  | UTP18 |  |  |  |
|  | WHAMM |  |  |  |
|  | ZFYVE1 |  |  |  |
|  | ZNF652 |  |  |  |
|  | DZIP3 |  |  |  |
|  | HIF1AN |  |  |  |
|  | IGHM |  |  |  |
|  | RNF152 |  |  |  |
|  | SEPSECS |  |  |  |
|  | SERTAD3 |  |  |  |
|  | SYT4 |  |  |  |
|  | ABCA12 |  |  |  |
|  | AGPAT5 |  |  |  |
|  | ARHGAP19 |  |  |  |
|  | CACNA2D3 |  |  |  |
|  | FAM210A |  |  |  |
|  | IL12RB1 |  |  |  |
|  | LDHC |  |  |  |
|  | METRN |  |  |  |
|  | MSC |  |  |  |
|  | SAA4 |  |  |  |
|  | TFB2M |  |  |  |
|  | XIRP1 |  |  |  |
|  | ACTR1A |  |  |  |
|  | FAP |  |  |  |
|  | KIF7 |  |  |  |
|  | LDB1 |  |  |  |
|  | MRPS18C |  |  |  |
|  | NUFIP2 |  |  |  |
|  | RBM6 |  |  |  |
|  | SSTR1 |  |  |  |
|  | ST6GALNAC4 |  |  |  |
|  | TMX4 |  |  |  |
|  | TRIM32 |  |  |  |
|  | UBE2G2 |  |  |  |
|  | YTHDF3 |  |  |  |
|  | BTK |  |  |  |
|  | CNOT6L |  |  |  |
|  | GSTM1 |  |  |  |
|  | NDUFB7 |  |  |  |
|  | PBLD |  |  |  |
|  | UBN2 |  |  |  |
|  | VWCE |  |  |  |
|  | A4GALT |  |  |  |
|  | ACOT12 |  |  |  |
|  | COX11 |  |  |  |
|  | CUBN |  |  |  |
|  | FSD1L |  |  |  |
|  | GIPC2 |  |  |  |
|  | HELQ |  |  |  |
|  | HSPA14 |  |  |  |
|  | MIR17HG |  |  |  |
|  | TMEM184C |  |  |  |
|  | DHPS |  |  |  |
|  | EPC2 |  |  |  |
|  | MRPL41 |  |  |  |
|  | MTFR1 |  |  |  |
|  | NDUFB1 |  |  |  |
|  | PKD1 |  |  |  |
|  | TRPC1 |  |  |  |
|  | ASXL2 |  |  |  |
|  | GAPDHS |  |  |  |
|  | MFSD4A |  |  |  |
|  | MTERF3 |  |  |  |
|  | SLC25A34 |  |  |  |
|  | SLC35F5 |  |  |  |
|  | TMEFF1 |  |  |  |
|  | USH1C |  |  |  |
|  | ADGRL1 |  |  |  |
|  | AFMID |  |  |  |
|  | BNC1 |  |  |  |
|  | CAPRIN2 |  |  |  |
|  | CCR4 |  |  |  |
|  | CDYL |  |  |  |
|  | FNTB |  |  |  |
|  | GPR157 |  |  |  |
|  | ICOSLG |  |  |  |
|  | IFT20 |  |  |  |
|  | ING2 |  |  |  |
|  | MICALL2 |  |  |  |
|  | PIGN |  |  |  |
|  | PLPP6 |  |  |  |
|  | SCLT1 |  |  |  |
|  | TICAM2 |  |  |  |
|  | TRIQK |  |  |  |
|  | ZNF292 |  |  |  |
|  | CCNT1 |  |  |  |
|  | CDH16 |  |  |  |
|  | CEP85L |  |  |  |
|  | COX7A2L |  |  |  |
|  | OMA1 |  |  |  |
|  | SIRT1 |  |  |  |
|  | TMC4 |  |  |  |
|  | WDR72 |  |  |  |
|  | ATP8B2 |  |  |  |
|  | CALHM2 |  |  |  |
|  | CREBL2 |  |  |  |
|  | FNDC4 |  |  |  |
|  | LIN37 |  |  |  |
|  | LRRC1 |  |  |  |
|  | MAK |  |  |  |
|  | MED12 |  |  |  |
|  | NDUFB11 |  |  |  |
|  | NSMCE4A |  |  |  |
|  | ORC2 |  |  |  |
|  | PHKA2 |  |  |  |
|  | RPS6KA4 |  |  |  |
|  | SLC25A42 |  |  |  |
|  | SLITRK5 |  |  |  |
|  | SNRNP25 |  |  |  |
|  | SNX2 |  |  |  |
|  | SPATA18 |  |  |  |
|  | TRIM8 |  |  |  |
|  | ZXDC |  |  |  |
|  | ABI3 |  |  |  |
|  | CHL1 |  |  |  |
|  | IIGP1 |  |  |  |
|  | MAP1LC3B |  |  |  |
|  | ORAI3 |  |  |  |
|  | XYLT2 |  |  |  |
|  | ADRA2B |  |  |  |
|  | ARHGAP12 |  |  |  |
|  | CYP4F3 |  |  |  |
|  | DDI2 |  |  |  |
|  | EED |  |  |  |
|  | GRAP2 |  |  |  |
|  | NFYC |  |  |  |
|  | RRP7A |  |  |  |
|  | RSPH1 |  |  |  |
|  | SYNE3 |  |  |  |
|  | TLR8 |  |  |  |
|  | ATOSB |  |  |  |
|  | BAG4 |  |  |  |
|  | CANT1 |  |  |  |
|  | CCNJ |  |  |  |
|  | FEM1C |  |  |  |
|  | FZD9 |  |  |  |
|  | GPX1A |  |  |  |
|  | HAPLN3 |  |  |  |
|  | IL18BP |  |  |  |
|  | MTMR11 |  |  |  |
|  | PDSS2 |  |  |  |
|  | PLBD2 |  |  |  |
|  | SLC25A16 |  |  |  |
|  | STX6 |  |  |  |
|  | STX8 |  |  |  |
|  | TMEM38A |  |  |  |
|  | CLDN10 |  |  |  |
|  | GPATCH2L |  |  |  |
|  | MRPL24 |  |  |  |
|  | MUC13 |  |  |  |
|  | TMEM177 |  |  |  |
|  | AKAP10 |  |  |  |
|  | B3GNT7 |  |  |  |
|  | BEST1 |  |  |  |
|  | BROX |  |  |  |
|  | CLIC2 |  |  |  |
|  | CSTF1 |  |  |  |
|  | DCP1A |  |  |  |
|  | DENND4C |  |  |  |
|  | FAM120A |  |  |  |
|  | FBXW5 |  |  |  |
|  | GPR132 |  |  |  |
|  | HMBOX1 |  |  |  |
|  | IL3RA |  |  |  |
|  | ITFG1 |  |  |  |
|  | KNOP1 |  |  |  |
|  | MAPKAP1 |  |  |  |
|  | MCTP2 |  |  |  |
|  | MIR150 |  |  |  |
|  | PLA2G12B |  |  |  |
|  | PPP1R1C |  |  |  |
|  | RIMKLB |  |  |  |
|  | RNF150 |  |  |  |
|  | RUNDC3B |  |  |  |
|  | ST6GALNAC5 |  |  |  |
|  | VPS4B |  |  |  |
|  | ZCCHC14 |  |  |  |
|  | ANKMY2 |  |  |  |
|  | ATP8B4 |  |  |  |
|  | AZIN2 |  |  |  |
|  | KCNG3 |  |  |  |
|  | POLR2I |  |  |  |
|  | SCN9A |  |  |  |
|  | VARS2 |  |  |  |
|  | ACSM5 |  |  |  |
|  | EPOP |  |  |  |
|  | INHBC |  |  |  |
|  | MRPL15 |  |  |  |
|  | PKIG |  |  |  |
|  | SCUBE3 |  |  |  |
|  | TMEM87B |  |  |  |
|  | TRIP6 |  |  |  |
|  | ZBTB2 |  |  |  |
|  | CCDC28A |  |  |  |
|  | EXOC8 |  |  |  |
|  | TMEM218 |  |  |  |
|  | AGTRAP |  |  |  |
|  | CYP4A3 |  |  |  |
|  | FANCE |  |  |  |
|  | MOG |  |  |  |
|  | MRPL40 |  |  |  |
|  | ORAI1 |  |  |  |
|  | PAIP2B |  |  |  |
|  | POLR1E |  |  |  |
|  | SLC25A45 |  |  |  |
|  | UBE2D2 |  |  |  |
|  | FCRLA |  |  |  |
|  | MACC1 |  |  |  |
|  | MLXIP |  |  |  |
|  | RAD54L2 |  |  |  |
|  | DYM |  |  |  |
|  | MCRIP2 |  |  |  |
|  | MRPL37 |  |  |  |
|  | MRPL47 |  |  |  |
|  | MRPS16 |  |  |  |
|  | MRPS26 |  |  |  |
|  | NDUFC1 |  |  |  |
|  | NUDT3 |  |  |  |
|  | PLSCR2 |  |  |  |
|  | RABL3 |  |  |  |
|  | SPOCD1 |  |  |  |
|  | TAB3 |  |  |  |
|  | TRAPPC10 |  |  |  |
|  | EDARADD |  |  |  |
|  | MAK16 |  |  |  |
|  | MRPL9 |  |  |  |
|  | NDN |  |  |  |
|  | RPP25L |  |  |  |
|  | SYCE2 |  |  |  |
|  | DDX59 |  |  |  |
|  | DUSP13B |  |  |  |
|  | ELAPOR1 |  |  |  |
|  | IL36G |  |  |  |
|  | KCNN3 |  |  |  |
|  | KISS1R |  |  |  |
|  | MIR20A |  |  |  |
|  | MIR34C |  |  |  |
|  | RAB33A |  |  |  |
|  | SLC35F3 |  |  |  |
|  | TLL1 |  |  |  |
|  | AGRP |  |  |  |
|  | ANKRD29 |  |  |  |
|  | ASGR2 |  |  |  |
|  | FBXO22 |  |  |  |
|  | FBXO4 |  |  |  |
|  | GTF3C4 |  |  |  |
|  | IMP4 |  |  |  |
|  | KCTD17 |  |  |  |
|  | MTREX |  |  |  |
|  | NUDCD2 |  |  |  |
|  | PAXIP1 |  |  |  |
|  | PLAAT4 |  |  |  |
|  | PUM2 |  |  |  |
|  | REPS2 |  |  |  |
|  | ROBO4 |  |  |  |
|  | SLC28A1 |  |  |  |
|  | TRAPPC1 |  |  |  |
|  | TSTD1 |  |  |  |
|  | VPS41 |  |  |  |
|  | ZMYM6 |  |  |  |
|  | AGMO |  |  |  |
|  | COQ9 |  |  |  |
|  | RC3H1 |  |  |  |
|  | ACKR1 |  |  |  |
|  | CHST7 |  |  |  |
|  | DRAXIN |  |  |  |
|  | HNF4G |  |  |  |
|  | MINDY3 |  |  |  |
|  | PCX |  |  |  |
|  | PDZD8 |  |  |  |
|  | RFX3 |  |  |  |
|  | S100A2 |  |  |  |
|  | SCMH1 |  |  |  |
|  | SERPINB7 |  |  |  |
|  | SLAMF1 |  |  |  |
|  | SLX4 |  |  |  |
|  | TMEM59 |  |  |  |
|  | CLEC3B |  |  |  |
|  | GMPR2 |  |  |  |
|  | IFI204 |  |  |  |
|  | LETMD1 |  |  |  |
|  | MRPL22 |  |  |  |
|  | PMEL |  |  |  |
|  | RNF31 |  |  |  |
|  | RTCB |  |  |  |
|  | SPATA17 |  |  |  |
|  | MCPH1 |  |  |  |
|  | BTBD7 |  |  |  |
|  | CD300LF |  |  |  |
|  | CDHR1 |  |  |  |
|  | DNAAF2 |  |  |  |
|  | HGH1 |  |  |  |
|  | HLA-DRA |  |  |  |
|  | IL22RA1 |  |  |  |
|  | KLHL6 |  |  |  |
|  | LMBRD2 |  |  |  |
|  | LSMEM1 |  |  |  |
|  | RESF1 |  |  |  |
|  | SLC17A9 |  |  |  |
|  | SMIM7 |  |  |  |
|  | SNAPC4 |  |  |  |
|  | SS18L2 |  |  |  |
|  | TRUB1 |  |  |  |
|  | VAMP3 |  |  |  |
|  | ZNHIT6 |  |  |  |
|  | AGO3 |  |  |  |
|  | DNAAF1 |  |  |  |
|  | PBX4 |  |  |  |
|  | PNCK |  |  |  |
|  | SLC2A14 |  |  |  |
|  | SYCP1 |  |  |  |
|  | CLDN6 |  |  |  |
|  | DESI1 |  |  |  |
|  | EFCAB7 |  |  |  |
|  | GAREM1 |  |  |  |
|  | MIR27B |  |  |  |
|  | NEMP1 |  |  |  |
|  | PLEKHM3 |  |  |  |
|  | SLAIN1 |  |  |  |
|  | TBC1D20 |  |  |  |
|  | USP45 |  |  |  |
|  | AJAP1 |  |  |  |
|  | ALOX12B |  |  |  |
|  | ASB7 |  |  |  |
|  | CEP19 |  |  |  |
|  | CYP19A1 |  |  |  |
|  | TAF1A |  |  |  |
|  | TIGD2 |  |  |  |
|  | ZC3HAV1L |  |  |  |
|  | ZNF148 |  |  |  |
|  | BECN1 |  |  |  |
|  | LMAN2 |  |  |  |
|  | MXRA5 |  |  |  |
|  | NAP1L3 |  |  |  |
|  | NEK9 |  |  |  |
|  | PLGRKT |  |  |  |
|  | SCO1 |  |  |  |
|  | STX4 |  |  |  |
|  | WDR90 |  |  |  |
|  | ZBTB41 |  |  |  |
|  | ZFYVE19 |  |  |  |
|  | DSG3 |  |  |  |
|  | DUSP19 |  |  |  |
|  | ENTPD7 |  |  |  |
|  | EVI2B |  |  |  |
|  | H2AC11 |  |  |  |
|  | STAP1 |  |  |  |
|  | ARPC3 |  |  |  |
|  | C5AR2 |  |  |  |
|  | KLHDC4 |  |  |  |
|  | PHLPP2 |  |  |  |
|  | PRAM1 |  |  |  |
|  | SEC22A |  |  |  |
|  | SNAI3 |  |  |  |
|  | TRMT12 |  |  |  |
|  | UBE3D |  |  |  |
|  | ASPRV1 |  |  |  |
|  | COX16 |  |  |  |
|  | GOLGA8A |  |  |  |
|  | NOS2A |  |  |  |
|  | TBL2 |  |  |  |
|  | UBXN7 |  |  |  |
|  | VSTM4 |  |  |  |
|  | AMBRA1 |  |  |  |
|  | CLEC2B |  |  |  |
|  | FAM217B |  |  |  |
|  | MIR17 |  |  |  |
|  | NARS2 |  |  |  |
|  | PCED1B |  |  |  |
|  | PHF23 |  |  |  |
|  | PI4KB |  |  |  |
|  | TAF5L |  |  |  |
|  | TM2D3 |  |  |  |
|  | DNHD1 |  |  |  |
|  | IFT46 |  |  |  |
|  | L3MBTL1 |  |  |  |
|  | MB21D2 |  |  |  |
|  | MRPS21 |  |  |  |
|  | SMCR8 |  |  |  |
|  | SPNS1 |  |  |  |
|  | TMEM200A |  |  |  |
|  | TUBD1 |  |  |  |
|  | WDR59 |  |  |  |
|  | ZNRF3 |  |  |  |
|  | CDK2 |  |  |  |
|  | ANKS6 |  |  |  |
|  | CBX8 |  |  |  |
|  | CEP250 |  |  |  |
|  | DENND1C |  |  |  |
|  | DIRAS1 |  |  |  |
|  | EEFSEC |  |  |  |
|  | EXT2 |  |  |  |
|  | GYG2 |  |  |  |
|  | INPP5F |  |  |  |
|  | OSCAR |  |  |  |
|  | PTPMT1 |  |  |  |
|  | WWC3 |  |  |  |
|  | YIPF3 |  |  |  |
|  | AFF2 |  |  |  |
|  | BBS7 |  |  |  |
|  | EDA |  |  |  |
|  | FBXO8 |  |  |  |
|  | GTF2H2 |  |  |  |
|  | HINFP |  |  |  |
|  | LRRC4 |  |  |  |
|  | MRPS11 |  |  |  |
|  | PAPOLG |  |  |  |
|  | PUS7L |  |  |  |
|  | USP42 |  |  |  |
|  | OSBPL7 |  |  |  |
|  | TRPM8 |  |  |  |
|  | HARS2 |  |  |  |
|  | LCLAT1 |  |  |  |
|  | MEIS3 |  |  |  |
|  | PPAP2A |  |  |  |
|  | ARL10 |  |  |  |
|  | ARMC10 |  |  |  |
|  | C15ORF39 |  |  |  |
|  | CDKL3 |  |  |  |
|  | CDR2L |  |  |  |
|  | DCTN5 |  |  |  |
|  | EBF2 |  |  |  |
|  | INSM1 |  |  |  |
|  | ISG20L2 |  |  |  |
|  | KLHL18 |  |  |  |
|  | NDUFAF2 |  |  |  |
|  | NUFIP1 |  |  |  |
|  | PLPPR3 |  |  |  |
|  | SHLD2 |  |  |  |
|  | SNHG7 |  |  |  |
|  | TEX9 |  |  |  |
|  | VPS11 |  |  |  |
|  | WIPF2 |  |  |  |
|  | ZNF532 |  |  |  |
|  | CCDC146 |  |  |  |
|  | RRAGA |  |  |  |
|  | SARS2 |  |  |  |
|  | AIM2 |  |  |  |
|  | CENATAC |  |  |  |
|  | DQX1 |  |  |  |
|  | FBXO15 |  |  |  |
|  | FKBP15 |  |  |  |
|  | MED26 |  |  |  |
|  | MRPL48 |  |  |  |
|  | OBI1 |  |  |  |
|  | PHACTR3 |  |  |  |
|  | SPEF2 |  |  |  |
|  | WSCD2 |  |  |  |
|  | ZBTB14 |  |  |  |
|  | PLCXD1 |  |  |  |
|  | ANKRD13D |  |  |  |
|  | CFAP52 |  |  |  |
|  | COL21A1 |  |  |  |
|  | GPBAR1 |  |  |  |
|  | LHB |  |  |  |
|  | MATK |  |  |  |
|  | MEA1 |  |  |  |
|  | PEX11G |  |  |  |
|  | PIP5K1B |  |  |  |
|  | ANAPC10 |  |  |  |
|  | CEP43 |  |  |  |
|  | ENTPD8 |  |  |  |
|  | FBXO16 |  |  |  |
|  | JKAMP |  |  |  |
|  | MIR15A |  |  |  |
|  | MIR20B |  |  |  |
|  | NHSL2 |  |  |  |
|  | TUBB1 |  |  |  |
|  | UBE2G1 |  |  |  |
|  | ACBD7 |  |  |  |
|  | CCDC8 |  |  |  |
|  | F2RL3 |  |  |  |
|  | GBA2 |  |  |  |
|  | METTL22 |  |  |  |
|  | RBM41 |  |  |  |
|  | TIMM8A |  |  |  |
|  | CCDC137 |  |  |  |
|  | ERAP2 |  |  |  |
|  | FAAP20 |  |  |  |
|  | HOXD10 |  |  |  |
|  | ICAM4 |  |  |  |
|  | MYRFL |  |  |  |
|  | TBP |  |  |  |
|  | TMEM108 |  |  |  |
|  | UBE2D4 |  |  |  |
|  | CCDC12 |  |  |  |
|  | HECTD4 |  |  |  |
|  | KANSL1L |  |  |  |
|  | KIF6 |  |  |  |
|  | MARCHF4 |  |  |  |
|  | PODN |  |  |  |
|  | SPSB2 |  |  |  |
|  | TIMM22 |  |  |  |
|  | ZBTB8A |  |  |  |
|  | INTS1 |  |  |  |
|  | TFAP2E |  |  |  |
|  | AP3S2 |  |  |  |
|  | BBS1 |  |  |  |
|  | DNAI3 |  |  |  |
|  | EMSY |  |  |  |
|  | KIN |  |  |  |
|  | MIR99B |  |  |  |
|  | MRM2 |  |  |  |
|  | MYEOV |  |  |  |
|  | PRDM4 |  |  |  |
|  | SPP2 |  |  |  |
|  | STOML1 |  |  |  |
|  | WARS2 |  |  |  |
|  | ZNF267 |  |  |  |
|  | BCHE |  |  |  |
|  | CLDN8 |  |  |  |
|  | ERGIC2 |  |  |  |
|  | ZNF217 |  |  |  |
|  | IFI27L2A |  |  |  |
|  | KLF1 |  |  |  |
|  | CHSY3 |  |  |  |
|  | EPHA6 |  |  |  |
|  | IL36RN |  |  |  |
|  | LAPTM4A |  |  |  |
|  | MIR106B |  |  |  |
|  | SNAPC5 |  |  |  |
|  | XBP1 |  |  |  |
|  | CUL9 |  |  |  |
|  | EPHX1 |  |  |  |
|  | MFSD13A |  |  |  |
|  | MTHFSD |  |  |  |
|  | RFXAP |  |  |  |
|  | SDHAF1 |  |  |  |
|  | ZNF473 |  |  |  |
|  | ABCC1 |  |  |  |
|  | AOAH |  |  |  |
|  | CYSLTR2 |  |  |  |
|  | ADCK2 |  |  |  |
|  | COMMD5 |  |  |  |
|  | HOXA7 |  |  |  |
|  | MRPS23 |  |  |  |
|  | MRPS5 |  |  |  |
|  | MRPS9 |  |  |  |
|  | TMEM41A |  |  |  |
|  | VPS37D |  |  |  |
|  | CCDC115 |  |  |  |
|  | CCDC30 |  |  |  |
|  | DNAJC27 |  |  |  |
|  | FDX2 |  |  |  |
|  | FOXE1 |  |  |  |
|  | GLYAT |  |  |  |
|  | GZMK |  |  |  |
|  | TBC1D32 |  |  |  |
|  | TH |  |  |  |
|  | TNFSF10 |  |  |  |
|  | GPR107 |  |  |  |
|  | HCAR3 |  |  |  |
|  | UCN |  |  |  |
|  | FUT7 |  |  |  |
|  | PTGDR2 |  |  |  |
|  | ADIRF |  |  |  |
|  | ELMOD3 |  |  |  |
|  | FABP10A |  |  |  |
|  | GCOM1 |  |  |  |
|  | KLHL30 |  |  |  |
|  | NELFCD |  |  |  |
|  | PNMT |  |  |  |
|  | SLC6A18 |  |  |  |
|  | ABCB5 |  |  |  |
|  | ATP6V1G2 |  |  |  |
|  | C11ORF54 |  |  |  |
|  | FBXO46 |  |  |  |
|  | HRH4 |  |  |  |
|  | TAF11 |  |  |  |
|  | MBPA |  |  |  |
|  | CBFA2T2 |  |  |  |
|  | CLEC12A |  |  |  |
|  | MIR152 |  |  |  |
|  | MSH4 |  |  |  |
|  | TACO1 |  |  |  |
|  | BAXA |  |  |  |
|  | BTG4 |  |  |  |
|  | CREG2 |  |  |  |
|  | EIF4ENIF1 |  |  |  |
|  | GTF3C3 |  |  |  |
|  | MORC2 |  |  |  |
|  | TGS1 |  |  |  |
|  | ZSCAN21 |  |  |  |
|  | ORM3 |  |  |  |
|  | SLC24A2 |  |  |  |
|  | CYP2C9 |  |  |  |
|  | KCP |  |  |  |
|  | ARL14 |  |  |  |
|  | BBIP1 |  |  |  |
|  | BORCS5 |  |  |  |
|  | CACFD1 |  |  |  |
|  | PWWP2A |  |  |  |
|  | SNHG15 |  |  |  |
|  | TFRC |  |  |  |
|  | ZNF430 |  |  |  |
|  | ZNF624 |  |  |  |
|  | GEMIN8 |  |  |  |
|  | CDC20B |  |  |  |
|  | RPUSD2 |  |  |  |
|  | ABHD17C |  |  |  |
|  | APOBEC3G |  |  |  |
|  | DNAL4 |  |  |  |
|  | HENMT1 |  |  |  |
|  | IL31RA |  |  |  |
|  | MIR26B |  |  |  |
|  | MRPL10 |  |  |  |
|  | RASSF10 |  |  |  |
|  | RBFA |  |  |  |
|  | SP8 |  |  |  |
|  | GBA3 |  |  |  |
|  | KEAP1 |  |  |  |
|  | GGA1 |  |  |  |
|  | ANTKMT |  |  |  |
|  | ATXN1L |  |  |  |
|  | BCAS4 |  |  |  |
|  | EAPP |  |  |  |
|  | GFAP |  |  |  |
|  | MIRLET7I |  |  |  |
|  | PYHIN1 |  |  |  |
|  | TEX19 |  |  |  |
|  | ZNF436 |  |  |  |
|  | ZNF559 |  |  |  |
|  | ZNF721 |  |  |  |
|  | CALCR |  |  |  |
|  | CHRNA6 |  |  |  |
|  | SLC25A26 |  |  |  |
|  | FASTKD5 |  |  |  |
|  | MDP1 |  |  |  |
|  | CYP11A1 |  |  |  |
|  | H3C6 |  |  |  |
|  | NKPD1 |  |  |  |
|  | RAD9B |  |  |  |
|  | SPIC |  |  |  |
|  | TMEM217 |  |  |  |
|  | WFDC21P |  |  |  |
|  | ZDHHC1 |  |  |  |
|  | ZNF57 |  |  |  |
|  | MGAT4C |  |  |  |
|  | YPEL4 |  |  |  |
|  | SIRPB1 |  |  |  |
|  | TMEM68 |  |  |  |
|  | TTLL11 |  |  |  |
|  | AFG2B |  |  |  |
|  | CHP2 |  |  |  |
|  | CNEP1R1 |  |  |  |
|  | FAM199X |  |  |  |
|  | FAM98C |  |  |  |
|  | KIAA0319 |  |  |  |
|  | RIMBP3 |  |  |  |
|  | TDRD9 |  |  |  |
|  | CD300LB |  |  |  |
|  | HSD3B6 |  |  |  |
|  | CFAP96 |  |  |  |
|  | LYRM4 |  |  |  |
|  | NBPF10 |  |  |  |
|  | TMEM185A |  |  |  |
|  | ACSM2 |  |  |  |
|  | CTU2 |  |  |  |
|  | CD1D |  |  |  |
|  | CYP2D2 |  |  |  |
|  | ERICH3 |  |  |  |
|  | MOSMO |  |  |  |
|  | SMIM19 |  |  |  |
|  | HSPA8 |  |  |  |
|  | TAFAZZIN |  |  |  |
|  | UGT3A1 |  |  |  |
|  | VIPAS39 |  |  |  |
|  | LDHA |  |  |  |
|  | MRPL43 |  |  |  |
|  | MUC19 |  |  |  |
|  | TESPA1 |  |  |  |
|  | CCDC177 |  |  |  |
|  | NDUFAF6 |  |  |  |
|  | OARD1 |  |  |  |
|  | FASLG |  |  |  |
|  | PLAT |  |  |  |
|  | CATSPERD |  |  |  |
|  | H3-3A |  |  |  |
|  | LGALS12 |  |  |  |
|  | LRIG2 |  |  |  |
|  | MEI1 |  |  |  |
|  | TMEM200C |  |  |  |
|  | TRIM4 |  |  |  |
|  | TTI1 |  |  |  |
|  | PIK3R1 |  |  |  |
|  | COL3A1 |  |  |  |
|  | CYP4F5 |  |  |  |
|  | FTMT |  |  |  |
|  | IFI202B |  |  |  |
|  | NPPA |  |  |  |
|  | ODC1 |  |  |  |
|  | SMAD3 |  |  |  |
|  | ZNF195 |  |  |  |
|  | AGTR1B |  |  |  |
|  | UMOD |  |  |  |
|  | PRR18 |  |  |  |
|  | C9ORF85 |  |  |  |
|  | DEFB4A |  |  |  |
|  | FAAP24 |  |  |  |
|  | TNFRSF18 |  |  |  |
|  | ZNF124 |  |  |  |
|  | ZNF383 |  |  |  |
|  | GOT1 |  |  |  |
|  | CD86 |  |  |  |
|  | CTSD |  |  |  |
|  | JUN |  |  |  |
|  | PLAU |  |  |  |
|  | ZNF101 |  |  |  |
|  | CYP3A65 |  |  |  |
|  | PMAIP1 |  |  |  |
|  | CCR10 |  |  |  |
|  | KERA |  |  |  |
|  | C2CD4A |  |  |  |
|  | CD160 |  |  |  |
|  | KRT12 |  |  |  |
|  | GIPC3 |  |  |  |
|  | ACOX1 |  |  |  |
|  | MYD88 |  |  |  |
|  | AIFM1 |  |  |  |
|  | HMGB1 |  |  |  |
|  | AHR |  |  |  |
|  | JAK2 |  |  |  |
|  | SCARB1 |  |  |  |
|  | ABITRAM |  |  |  |
|  | C3ORF38 |  |  |  |
|  | CYP4F15 |  |  |  |
|  | ZNF232 |  |  |  |
|  | CD14 |  |  |  |
|  | GSTM2 |  |  |  |
|  | IL5 |  |  |  |
|  | CYBB |  |  |  |
|  | GSTA3 |  |  |  |
|  | TIMP2 |  |  |  |
|  | CCL4 |  |  |  |
|  | IRS1 |  |  |  |
|  | C9ORF152 |  |  |  |
|  | CCNB3 |  |  |  |
|  | LINC01588 |  |  |  |
|  | ZNF823 |  |  |  |
|  | BCL2L11 |  |  |  |
|  | RXRA |  |  |  |
|  | BCL2A |  |  |  |
|  | CASC15 |  |  |  |
|  | GPX4 |  |  |  |
|  | IL1RN |  |  |  |
|  | MZT2B |  |  |  |
|  | NMRAL2P |  |  |  |
|  | PCDHGA3 |  |  |  |
|  | LCN2 |  |  |  |
|  | PRKCA |  |  |  |
|  | TUSC1 |  |  |  |
|  | ZNF266 |  |  |  |
|  | ZNF493 |  |  |  |
|  | ZNF621 |  |  |  |
|  | ZNF85 |  |  |  |
|  | RPS6 |  |  |  |
|  | KCNK15 |  |  |  |
|  | ADRB2 |  |  |  |
|  | GSTT1B |  |  |  |
|  | C16ORF74 |  |  |  |
|  | GADD45B |  |  |  |
|  | SLFN12 |  |  |  |
|  | ZNF273 |  |  |  |
|  | GLUL |  |  |  |
|  | ACTB |  |  |  |
|  | C3 |  |  |  |
|  | NPPB |  |  |  |
|  | PYCARD |  |  |  |
|  | RRM2 |  |  |  |
|  | BBC3 |  |  |  |
|  | MIR181D |  |  |  |
|  | NOTCH2NLA |  |  |  |
|  | AKR1B1 |  |  |  |
|  | CHI3L2 |  |  |  |
|  | GADL1 |  |  |  |
|  | SLC16A6B |  |  |  |
|  | CLDN1 |  |  |  |
|  | TRAC |  |  |  |
|  | PGBD3 |  |  |  |
|  | MAFF |  |  |  |
|  | HBEGF |  |  |  |
|  | ABCB4 |  |  |  |
|  | CCL13 |  |  |  |
|  | KLRB1 |  |  |  |
|  | TSSK4 |  |  |  |
|  | COL2A1 |  |  |  |
|  | CALCA |  |  |  |
|  | KRT18 |  |  |  |
|  | LINC00174 |  |  |  |
|  | ZNF439 |  |  |  |
|  | ITGB1 |  |  |  |
|  | NCF1 |  |  |  |
|  | PPARD |  |  |  |
|  | GSTM3 |  |  |  |
|  | ZNF564 |  |  |  |
|  | CD1A |  |  |  |
|  | CFTR |  |  |  |
|  | HSD11B1 |  |  |  |
|  | THBS1 |  |  |  |
|  | CYP3A23-3A1 |  |  |  |
|  | NTRK2 |  |  |  |
|  | TGM2 |  |  |  |
|  | PDGFRB |  |  |  |
|  | RHOA |  |  |  |
|  | APOB |  |  |  |
|  | GLIPR1L2 |  |  |  |
|  | MAPK8 |  |  |  |
|  | SNAI1 |  |  |  |
|  | MSMO1 |  |  |  |
|  | AFP |  |  |  |
|  | NR4A2 |  |  |  |
|  | VDR |  |  |  |
|  | GBP |  |  |  |
|  | MIR106A |  |  |  |
|  | USP9Y |  |  |  |
|  | ST20-AS1 |  |  |  |
|  | ZNF398 |  |  |  |
|  | TAGLN |  |  |  |
|  | BMP2 |  |  |  |
|  | MAOB |  |  |  |
|  | NUPR1 |  |  |  |
|  | RAC1 |  |  |  |
|  | SLC7A5 |  |  |  |
|  | TIMP3 |  |  |  |
|  | CSF3 |  |  |  |
|  | CCL20 |  |  |  |
|  | HAVCR1 |  |  |  |
|  | SSMEM1 |  |  |  |
|  | CTSL |  |  |  |
|  | SESN2 |  |  |  |
|  | TGFA |  |  |  |
|  | PRPS1L1 |  |  |  |
|  | ANXA2 |  |  |  |
|  | DNM1L |  |  |  |
|  | CCNG1 |  |  |  |
|  | DUSP5 |  |  |  |
|  | PDGFB |  |  |  |
|  | RARA |  |  |  |
|  | COLCA1 |  |  |  |
|  | RGS2 |  |  |  |
|  | TNC |  |  |  |
|  | IGF2 |  |  |  |
|  | INHBA |  |  |  |
|  | MIF |  |  |  |
|  | SLC22A18AS |  |  |  |
|  | TPM1 |  |  |  |
|  | FDFT1 |  |  |  |
|  | PRDX2 |  |  |  |
|  | TGFBR2 |  |  |  |
|  | ITGB2 |  |  |  |
|  | PSAT1 |  |  |  |
|  | TNFRSF12A |  |  |  |
|  | MBP |  |  |  |
|  | REN |  |  |  |
|  | CLEC18B |  |  |  |
|  | KLF6 |  |  |  |
|  | PKD1L3 |  |  |  |
|  | SLC22A8 |  |  |  |
|  | MIR590 |  |  |  |
|  | ABCA11P |  |  |  |
|  | CASP6 |  |  |  |
|  | CHUK |  |  |  |
|  | MAP2K2 |  |  |  |
|  | PCK2 |  |  |  |
|  | PGK1 |  |  |  |
|  | ANXA5 |  |  |  |
|  | CRH |  |  |  |
|  | COX7A2A |  |  |  |
|  | SLX1A |  |  |  |
|  | ASH1L-AS1 |  |  |  |
|  | GPR31 |  |  |  |
|  | TK1 |  |  |  |
|  | PHLDA1 |  |  |  |
|  | CKB |  |  |  |
|  | CPT2 |  |  |  |
|  | ARIH2OS |  |  |  |
|  | IRS2 |  |  |  |
|  | KRBA2 |  |  |  |
|  | ZNF767P |  |  |  |
|  | BMP4 |  |  |  |
|  | PDGFRA |  |  |  |
|  | MOGAT3 |  |  |  |
|  | IRF7 |  |  |  |
|  | CYP26A1 |  |  |  |
|  | IFRD1 |  |  |  |
|  | EDNRA |  |  |  |
|  | GPNMB |  |  |  |
|  | ADAMTS1 |  |  |  |
|  | GUSBP14 |  |  |  |
|  | VEGFAB |  |  |  |
|  | IL33 |  |  |  |
|  | LGALS1 |  |  |  |
|  | MYLK |  |  |  |
|  | SELENBP1 |  |  |  |
|  | PRNP |  |  |  |
|  | CCL7 |  |  |  |
|  | GCH1 |  |  |  |
|  | SELENOP |  |  |  |
|  | TGFB3 |  |  |  |
|  | S100A4 |  |  |  |
|  | VLDLR |  |  |  |
|  | AQP1 |  |  |  |
|  | CYB5A |  |  |  |
|  | LINC01004 |  |  |  |
|  | OFCC1 |  |  |  |
|  | C6ORF52 |  |  |  |
|  | COL4A1 |  |  |  |
|  | SLC6A19A.1 |  |  |  |
|  | TBL1Y |  |  |  |
|  | GNAS |  |  |  |
|  | GSN |  |  |  |
|  | COL1A1 |  |  |  |
|  | FST |  |  |  |
|  | CBR1 |  |  |  |
|  | SOCS2 |  |  |  |
|  | LPIN1 |  |  |  |
|  | CS |  |  |  |
|  | FBXO32 |  |  |  |
|  | PCNA |  |  |  |
|  | PDE4B |  |  |  |
|  | STAT5B |  |  |  |
|  | CD83 |  |  |  |
|  | THBD |  |  |  |
|  | SELP |  |  |  |
|  | ADORA2A |  |  |  |
|  | NAPSB |  |  |  |
|  | ERRFI1 |  |  |  |
|  | GPAM |  |  |  |
|  | GSS |  |  |  |
|  | TNFAIP6 |  |  |  |
|  | STC2 |  |  |  |
|  | CST3 |  |  |  |
|  | TIPARP |  |  |  |
|  | XRCC1 |  |  |  |
|  | ORM1 |  |  |  |
|  | PFKP |  |  |  |
|  | SORD |  |  |  |
|  | VEGFC |  |  |  |
|  | ACO1 |  |  |  |
|  | GH1 |  |  |  |
|  | IDH1 |  |  |  |
|  | IL11A |  |  |  |
|  | JAK1 |  |  |  |
|  | IL24 |  |  |  |
|  | LILRA1 |  |  |  |
|  | SLC38A2 |  |  |  |
|  | MARCKS |  |  |  |
|  | ACOT1 |  |  |  |
|  | LRP1 |  |  |  |
|  | ATF2 |  |  |  |
|  | HOXA-AS2 |  |  |  |
|  | HSD17B4 |  |  |  |
|  | PLD1 |  |  |  |
|  | ACAA2 |  |  |  |
|  | ARG2 |  |  |  |
|  | NPIPB15 |  |  |  |
|  | NREP |  |  |  |
|  | E2F8 |  |  |  |
|  | SERPINB2 |  |  |  |
|  | HADH |  |  |  |
|  | PPP1R3C |  |  |  |
|  | SLC22A6 |  |  |  |
|  | ATP1B1 |  |  |  |
|  | MGP |  |  |  |
|  | NES |  |  |  |
|  | P4HB |  |  |  |
|  | SLC20A1 |  |  |  |
|  | BCL6 |  |  |  |
|  | AKAP12 |  |  |  |
|  | ATP5F1A |  |  |  |
|  | BRAF |  |  |  |
|  | IGFBP4 |  |  |  |
|  | AGER |  |  |  |
|  | GAS6 |  |  |  |
|  | HPGD |  |  |  |
|  | PFKFB3 |  |  |  |
|  | SLCO2B1 |  |  |  |
|  | CAPN1 |  |  |  |
|  | EIF4E |  |  |  |
|  | KLHL24B |  |  |  |
|  | SLC25A15B |  |  |  |
|  | USP41P |  |  |  |
|  | CCL11 |  |  |  |
|  | VCAN |  |  |  |
|  | CTSK |  |  |  |
|  | SKP2 |  |  |  |
|  | LAMC2 |  |  |  |
|  | SLC1A5 |  |  |  |
|  | EGLN3 |  |  |  |
|  | IL1A |  |  |  |
|  | NCF2 |  |  |  |
|  | COL18A1 |  |  |  |
|  | CD80 |  |  |  |
|  | EZH2 |  |  |  |
|  | UGT2B7 |  |  |  |
|  | ZFAND2A |  |  |  |
|  | PARP1 |  |  |  |
|  | GBP2 |  |  |  |
|  | HSD17B7 |  |  |  |
|  | PER1 |  |  |  |
|  | PLPP3 |  |  |  |
|  | RIPK1 |  |  |  |
|  | ARHGAP29B |  |  |  |
|  | AVP |  |  |  |
|  | MOV10B.1 |  |  |  |
|  | RUNX1 |  |  |  |
|  | CFD |  |  |  |
|  | ACAT1 |  |  |  |
|  | MUC5AC |  |  |  |
|  | ACP5 |  |  |  |
|  | HMGA1 |  |  |  |
|  | RND3 |  |  |  |
|  | VWF |  |  |  |
|  | ABCC5 |  |  |  |
|  | HADHA |  |  |  |
|  | SLC27A2 |  |  |  |
|  | MAT2A |  |  |  |
|  | PPP3CA |  |  |  |
|  | SLC30A1 |  |  |  |
|  | SIK1 |  |  |  |
|  | SULF2 |  |  |  |
|  | RORC |  |  |  |
|  | ACSS2 |  |  |  |
|  | PMP22 |  |  |  |
|  | GPD1 |  |  |  |
|  | IGF1 |  |  |  |
|  | MAP3K1 |  |  |  |
|  | SESN1 |  |  |  |
|  | SPHK1 |  |  |  |
|  | C1S |  |  |  |
|  | SOX4 |  |  |  |
|  | CIDEC |  |  |  |
|  | PHLDA3 |  |  |  |
|  | SPTAN1 |  |  |  |
|  | S1PR1 |  |  |  |
|  | PYGL |  |  |  |
|  | LY96 |  |  |  |
|  | PTPRF |  |  |  |
|  | ACO2 |  |  |  |
|  | DHRS3 |  |  |  |
|  | IFIT2 |  |  |  |
|  | PODXL |  |  |  |
|  | PGAM1 |  |  |  |
|  | PLCG1 |  |  |  |
|  | CD274 |  |  |  |
|  | LYN |  |  |  |
|  | MEF2C |  |  |  |
|  | PRKCZ |  |  |  |
|  | SLC19A1 |  |  |  |
|  | ACSL3 |  |  |  |
|  | SORL1 |  |  |  |
|  | BCL2A1 |  |  |  |
|  | COL12A1 |  |  |  |
|  | LIFR |  |  |  |
|  | TBX3 |  |  |  |
|  | ARRDC3 |  |  |  |
|  | SDHA |  |  |  |
|  | SLC25A20 |  |  |  |
|  | HTATIP2 |  |  |  |
|  | ZBTB16 |  |  |  |
|  | COL6A1 |  |  |  |
|  | CYP4B1 |  |  |  |
|  | EIF2A |  |  |  |
|  | PLTP |  |  |  |
|  | PTP4A1 |  |  |  |
|  | TNFAIP2 |  |  |  |
|  | WNT4 |  |  |  |
|  | ENO3 |  |  |  |
|  | GNAI2 |  |  |  |
|  | NEDD4L |  |  |  |
|  | UGT2B1 |  |  |  |
|  | USP2 |  |  |  |
|  | F2RL1 |  |  |  |
|  | ICAL1 |  |  |  |
|  | MYBL1 |  |  |  |
|  | PC |  |  |  |
|  | OLR1 |  |  |  |
|  | RND1 |  |  |  |
|  | CENPA |  |  |  |
|  | NSDHL |  |  |  |
|  | LAMP1 |  |  |  |
|  | MXD1 |  |  |  |
|  | FAAH |  |  |  |
|  | ROCK2 |  |  |  |
|  | SERPING1 |  |  |  |
|  | SOCS1 |  |  |  |
|  | ID4 |  |  |  |
|  | TJP2 |  |  |  |
|  | LCAT |  |  |  |
|  | PMEPA1 |  |  |  |
|  | P4HA1 |  |  |  |
|  | GATA6 |  |  |  |
|  | MLKL |  |  |  |
|  | COL4A2 |  |  |  |
|  | DCXR |  |  |  |
|  | ADORA2B |  |  |  |
|  | AGTR1A |  |  |  |
|  | GUCY1A1 |  |  |  |
|  | KRT17 |  |  |  |
|  | ATP2B1 |  |  |  |
|  | DMD |  |  |  |
|  | ECE1 |  |  |  |
|  | LINC00968 |  |  |  |
|  | REL |  |  |  |
|  | BDH1 |  |  |  |
|  | PTCH1 |  |  |  |
|  | FGF7 |  |  |  |
|  | IL13RA1 |  |  |  |
|  | ECI1 |  |  |  |
|  | CXCR2 |  |  |  |
|  | CASP7 |  |  |  |
|  | PPARGC1B |  |  |  |
|  | ADRA1B |  |  |  |
|  | APOC3 |  |  |  |
|  | IFI27 |  |  |  |
|  | RETSAT |  |  |  |
|  | CAMK2D |  |  |  |
|  | CHI3L1 |  |  |  |
|  | PPARGC1A |  |  |  |
|  | ADGRG1 |  |  |  |
|  | HIPK2 |  |  |  |
|  | ACADL |  |  |  |
|  | IL1RAP |  |  |  |
|  | SELL |  |  |  |
|  | SORT1 |  |  |  |
|  | SFRP2 |  |  |  |
|  | AGRN |  |  |  |
|  | ANKRD1 |  |  |  |
|  | NOTCH3 |  |  |  |
|  | PDLIM1 |  |  |  |
|  | PRKG1 |  |  |  |
|  | RPS27L |  |  |  |
|  | TNS1 |  |  |  |
|  | CDKN1B |  |  |  |
|  | CDK1 |  |  |  |
|  | MKI67 |  |  |  |
|  | HSPA1L |  |  |  |
|  | NEFM |  |  |  |
|  | NRP2 |  |  |  |
|  | GPT2 |  |  |  |
|  | CAV2 |  |  |  |
|  | HNRNPD |  |  |  |
|  | NPIPB7 |  |  |  |
|  | PEX11A |  |  |  |
|  | PON2 |  |  |  |
|  | PTGER2 |  |  |  |
|  | ABCC6 |  |  |  |
|  | CPS1 |  |  |  |
|  | LASP1 |  |  |  |
|  | CAST |  |  |  |
|  | DYNC1H1 |  |  |  |
|  | PTMA |  |  |  |
|  | BCAR3 |  |  |  |
|  | DLD |  |  |  |
|  | EIF2AK2 |  |  |  |
|  | SLC27A1 |  |  |  |
|  | DEPTOR |  |  |  |
|  | ETV5 |  |  |  |
|  | ISYNA1 |  |  |  |
|  | AGO2 |  |  |  |
|  | DLG1 |  |  |  |
|  | SPP1 |  |  |  |
|  | ATF3 |  |  |  |
|  | CAVIN2 |  |  |  |
|  | ST3GAL5 |  |  |  |
|  | FAT1 |  |  |  |
|  | MYOF |  |  |  |
|  | CRAT |  |  |  |
|  | EIF4A2 |  |  |  |
|  | HOMER1 |  |  |  |
|  | INS |  |  |  |
|  | CXCL11 |  |  |  |
|  | IQGAP2 |  |  |  |
|  | TRADD |  |  |  |
|  | ITGA3 |  |  |  |
|  | LBH |  |  |  |
|  | RXRB |  |  |  |
|  | VEGFD |  |  |  |
|  | PAH |  |  |  |
|  | MDH2 |  |  |  |
|  | EPB41L3 |  |  |  |
|  | PPARG |  |  |  |
|  | PPM1D |  |  |  |
|  | FDXR |  |  |  |
|  | ABHD2 |  |  |  |
|  | SLC2A5 |  |  |  |
|  | ADIPOR2 |  |  |  |
|  | PIK3CD |  |  |  |
|  | ACADS |  |  |  |
|  | FZD2 |  |  |  |
|  | OSMR |  |  |  |
|  | SH3PXD2A-AS1 |  |  |  |
|  | CDCA7 |  |  |  |
|  | HILPDA |  |  |  |
|  | KCNN4 |  |  |  |
|  | PVR |  |  |  |
|  | APOC1 |  |  |  |
|  | CDK5R1 |  |  |  |
|  | CKAP4 |  |  |  |
|  | ESR1 |  |  |  |
|  | MARCKSL1 |  |  |  |
|  | NID1 |  |  |  |
|  | SLC4A7 |  |  |  |
|  | HMOX2 |  |  |  |
|  | IFNGR1 |  |  |  |
|  | TMSB4X |  |  |  |
|  | ADH4 |  |  |  |
|  | CCNL1 |  |  |  |
|  | IRF9 |  |  |  |
|  | CXCL2 |  |  |  |
|  | PDXK |  |  |  |
|  | PKP2 |  |  |  |
|  | STOM |  |  |  |
|  | SIGLEC17P |  |  |  |
|  | AKT3 |  |  |  |
|  | MSR1 |  |  |  |
|  | ALDH9A1 |  |  |  |
|  | ANKRD12 |  |  |  |
|  | KL |  |  |  |
|  | PLIN1 |  |  |  |
|  | E2F3 |  |  |  |
|  | TENT5C |  |  |  |
|  | ZMYND8 |  |  |  |
|  | GUCY1B1 |  |  |  |
|  | NDUFS4 |  |  |  |
|  | GABBR2 |  |  |  |
|  | NPC2 |  |  |  |
|  | ABCG2 |  |  |  |
|  | GBP1 |  |  |  |
|  | GLS2 |  |  |  |
|  | RASSF4 |  |  |  |
|  | TLR1 |  |  |  |
|  | IVD |  |  |  |
|  | MAPK9 |  |  |  |
|  | RRS1 |  |  |  |
|  | SOD2 |  |  |  |
|  | SULF1 |  |  |  |
|  | CTNND1 |  |  |  |
|  | DLK1 |  |  |  |
|  | HSD17B6 |  |  |  |
|  | SAMHD1 |  |  |  |
|  | SET |  |  |  |
|  | ABHD4 |  |  |  |
|  | AMIGO2 |  |  |  |
|  | TP53INP1 |  |  |  |
|  | PDHA1 |  |  |  |
|  | MAP3K6 |  |  |  |
|  | STK17B |  |  |  |
|  | ETFB |  |  |  |
|  | KISS1 |  |  |  |
|  | SLCO1B2 |  |  |  |
|  | ITGB8 |  |  |  |
|  | AACS |  |  |  |
|  | EGF |  |  |  |
|  | EPB41L1 |  |  |  |
|  | PIK3CG |  |  |  |
|  | PDE4A |  |  |  |
|  | CHST15 |  |  |  |
|  | JADE1 |  |  |  |
|  | HTR2B |  |  |  |
|  | DHRS11 |  |  |  |
|  | RALA |  |  |  |
|  | RNF144B |  |  |  |
|  | SPRY1 |  |  |  |
|  | TRPV4 |  |  |  |
|  | CYP17A1 |  |  |  |
|  | FDX1 |  |  |  |
|  | ITGA7 |  |  |  |
|  | ADIPOQ |  |  |  |
|  | BDKRB1 |  |  |  |
|  | CDKN2D |  |  |  |
|  | LAMA4 |  |  |  |
|  | ATP5F1E |  |  |  |
|  | PDE3B |  |  |  |
|  | SLC51A |  |  |  |
|  | TBXA2R |  |  |  |
|  | ASL |  |  |  |
|  | MMD |  |  |  |
|  | NFKBIE |  |  |  |
|  | BBOX1 |  |  |  |
|  | HSDL2 |  |  |  |
|  | KCNJ8 |  |  |  |
|  | NAV3 |  |  |  |
|  | PANK1 |  |  |  |
|  | PIK3CB |  |  |  |
|  | PPP1R14B |  |  |  |
|  | MRPS18B |  |  |  |
|  | MYO5A |  |  |  |
|  | CLEC7A |  |  |  |
|  | ECI2 |  |  |  |
|  | IGF2BP3 |  |  |  |
|  | PIK3C2A |  |  |  |
|  | RNF19B |  |  |  |
|  | SARDH |  |  |  |
|  | TAX1BP3 |  |  |  |
|  | TBC1D4 |  |  |  |
|  | TOB1 |  |  |  |
|  | HCAR2 |  |  |  |
|  | PTH1R |  |  |  |
|  | RHBDF2 |  |  |  |
|  | DNASE1L3 |  |  |  |
|  | ARL4C |  |  |  |
|  | CASP8 |  |  |  |
|  | PCYOX1 |  |  |  |
|  | TRPM7 |  |  |  |
|  | ABHD5 |  |  |  |
|  | MAP3K14 |  |  |  |
|  | PTPRO |  |  |  |
|  | STEAP3 |  |  |  |
|  | BMI1 |  |  |  |
|  | RAPH1 |  |  |  |
|  | RPS3A |  |  |  |
|  | RRAS |  |  |  |
|  | GSK3A |  |  |  |
|  | ABCD2 |  |  |  |
|  | ABL2 |  |  |  |
|  | ACADSB |  |  |  |
|  | FXYD5 |  |  |  |
|  | MRPL12 |  |  |  |
|  | PECR |  |  |  |
|  | SLC46A3 |  |  |  |
|  | KIF14 |  |  |  |
|  | KLF12 |  |  |  |
|  | CH25H |  |  |  |
|  | OGN |  |  |  |
|  | IL10RA |  |  |  |
|  | TNFRSF11A |  |  |  |
|  | DEPDC1 |  |  |  |
|  | PLAAT3 |  |  |  |
|  | TLR6 |  |  |  |
|  | ARHGAP5 |  |  |  |
|  | ATOSA |  |  |  |
|  | CNDP2 |  |  |  |
|  | CSF2RB |  |  |  |
|  | ELOVL3 |  |  |  |
|  | MPZL1 |  |  |  |
|  | MUC2 |  |  |  |
|  | SEMA5A |  |  |  |
|  | UGT1A1 |  |  |  |
|  | CGB3 |  |  |  |
|  | COX5A |  |  |  |
|  | SSTR2 |  |  |  |
|  | ADCY5 |  |  |  |
|  | GFPT2 |  |  |  |
|  | GRPEL1 |  |  |  |
|  | PPP1CA |  |  |  |
|  | SCPEP1 |  |  |  |
|  | SLC7A11 |  |  |  |
|  | HIBADH |  |  |  |
|  | FGD4 |  |  |  |
|  | FH |  |  |  |
|  | HLA-B |  |  |  |
|  | MRPS6 |  |  |  |
|  | SGMS2 |  |  |  |
|  | ERCC4 |  |  |  |
|  | LINC01970 |  |  |  |
|  | ETFDH |  |  |  |
|  | SERPINB1 |  |  |  |
|  | YPEL2 |  |  |  |
|  | ALDH1A1 |  |  |  |
|  | GDF15 |  |  |  |
|  | TBK1 |  |  |  |
|  | ZC3HAV1 |  |  |  |
|  | AMOTL2 |  |  |  |
|  | AUH |  |  |  |
|  | CHCHD10 |  |  |  |
|  | MDM2 |  |  |  |
|  | P2RY1 |  |  |  |
|  | SPARCL1 |  |  |  |
|  | CHRDL1 |  |  |  |
|  | COX7C |  |  |  |
|  | NDUFA5 |  |  |  |
|  | NDUFV2 |  |  |  |
|  | ADCY1 |  |  |  |
|  | PRDM16 |  |  |  |
|  | NDUFAB1 |  |  |  |
|  | DHODH |  |  |  |
|  | WWC1 |  |  |  |
|  | DFFB |  |  |  |
|  | FGR |  |  |  |
|  | LPGAT1 |  |  |  |
|  | NUAK1 |  |  |  |
|  | PIAS1 |  |  |  |
|  | APOE |  |  |  |
|  | HPGDS |  |  |  |
|  | PPM1K |  |  |  |
|  | SLC22A2 |  |  |  |
|  | SPATA13 |  |  |  |
|  | G6PD |  |  |  |
|  | PAX8 |  |  |  |
|  | ADCY3 |  |  |  |
|  | CD70 |  |  |  |
|  | N4BP2L1 |  |  |  |
|  | XDH |  |  |  |
|  | SUCLA2 |  |  |  |
|  | RPS6KB1 |  |  |  |
|  | TAOK1 |  |  |  |
|  | USP15 |  |  |  |
|  | HEXA |  |  |  |
|  | SETBP1 |  |  |  |
|  | TMTC1 |  |  |  |
|  | FABP4 |  |  |  |
|  | HSPA1A |  |  |  |
|  | NR1H4 |  |  |  |
|  | HSPB1 |  |  |  |
|  | SAMD9L |  |  |  |
|  | MAST4 |  |  |  |
|  | PAMR1 |  |  |  |
|  | ZCCHC24 |  |  |  |
|  | CXCR3 |  |  |  |
|  | RNF213 |  |  |  |
|  | ELK3 |  |  |  |
|  | PXYLP1 |  |  |  |
|  | SLC7A6 |  |  |  |
|  | SMIM3 |  |  |  |
|  | TMED10 |  |  |  |
|  | ADGRL2 |  |  |  |
|  | ATP11A |  |  |  |
|  | NME4 |  |  |  |
|  | PACSIN2 |  |  |  |
|  | PTPN14 |  |  |  |
|  | SPTLC3 |  |  |  |
|  | APCDD1 |  |  |  |
|  | ND6 |  |  |  |
|  | NDUFA1 |  |  |  |
|  | NDUFS5 |  |  |  |
|  | RALGDS |  |  |  |
|  | RPL30 |  |  |  |
|  | TNFSF12 |  |  |  |
|  | FSHB |  |  |  |
|  | OSTM1 |  |  |  |
|  | SDHC |  |  |  |
|  | CDC42EP4 |  |  |  |
|  | IL10RB |  |  |  |
|  | STING1 |  |  |  |
|  | TIFA |  |  |  |
|  | UQCRB |  |  |  |
|  | VGLL4 |  |  |  |
|  | CYP51A1-AS1 |  |  |  |
|  | LERFS |  |  |  |
|  | LINC01686 |  |  |  |
|  | B3GNT5 |  |  |  |
|  | CCNC |  |  |  |
|  | CTDSPL |  |  |  |
|  | CYP7A1 |  |  |  |
|  | GALNT16 |  |  |  |
|  | KIF21B |  |  |  |
|  | MIPEP |  |  |  |
|  | OLFML2A |  |  |  |
|  | ABCA1 |  |  |  |
|  | MMP1 |  |  |  |
|  | SELPLG |  |  |  |
|  | NDUFS8 |  |  |  |
|  | SLC27A3 |  |  |  |
|  | TMEM171 |  |  |  |
|  | EIF2AK3 |  |  |  |
|  | TIMM17A |  |  |  |
|  | ADIPOR1 |  |  |  |
|  | EHF |  |  |  |
|  | NDUFB2 |  |  |  |
|  | UQCRFS1 |  |  |  |
|  | BMP2K |  |  |  |
|  | EPS15 |  |  |  |
|  | GMPPB |  |  |  |
|  | HIC1 |  |  |  |
|  | HMGCS1 |  |  |  |
|  | NDUFA3 |  |  |  |
|  | NDUFS3 |  |  |  |
|  | H2BC5 |  |  |  |
|  | PPP1R15B |  |  |  |
|  | HJV |  |  |  |
|  | NME7 |  |  |  |
|  | PILRA |  |  |  |
|  | RFFL |  |  |  |
|  | RNF145 |  |  |  |
|  | TTYH2 |  |  |  |
|  | ADRA2C |  |  |  |
|  | COX7A2 |  |  |  |
|  | ABHD11 |  |  |  |
|  | SLC38A5 |  |  |  |
|  | MARCO |  |  |  |
|  | PRICKLE2 |  |  |  |
|  | GSTA2 |  |  |  |
|  | XIAP |  |  |  |
|  | BNC2 |  |  |  |
|  | CXCL15 |  |  |  |
|  | GEMIN4 |  |  |  |
|  | LRP10 |  |  |  |
|  | MOGS |  |  |  |
|  | NDUFA7 |  |  |  |
|  | RMI2 |  |  |  |
|  | FGD3 |  |  |  |
|  | ST3GAL4 |  |  |  |
|  | TFB1M |  |  |  |
|  | TP53INP2 |  |  |  |
|  | LRIG3 |  |  |  |
|  | CEBPA |  |  |  |
|  | CD44 |  |  |  |
|  | KCNJ12 |  |  |  |
|  | ADTRP |  |  |  |
|  | ATP5MC3 |  |  |  |
|  | AGPAT4 |  |  |  |
|  | CYSLTR1 |  |  |  |
|  | H1-10 |  |  |  |
|  | ACHE |  |  |  |
|  | MRPL17 |  |  |  |
|  | NDUFB10 |  |  |  |
|  | NDUFB5 |  |  |  |
|  | SLC12A1 |  |  |  |
|  | AGPS |  |  |  |
|  | CCDC88C |  |  |  |
|  | LCOR |  |  |  |
|  | MAN1A2 |  |  |  |
|  | TJP1 |  |  |  |
|  | COL1A2 |  |  |  |
|  | PCK1 |  |  |  |
|  | PNOC |  |  |  |
|  | SULT1A1 |  |  |  |
|  | NEURL1B |  |  |  |
|  | SOCS5 |  |  |  |
|  | SURF1 |  |  |  |
|  | TNFRSF1A |  |  |  |
|  | CNR1 |  |  |  |
|  | GABPB1 |  |  |  |
|  | LEAP2 |  |  |  |
|  | PGP |  |  |  |
|  | CORO2B |  |  |  |
|  | CPED1 |  |  |  |
|  | SOX7 |  |  |  |
|  | TRIM13 |  |  |  |
|  | ANKRD44 |  |  |  |
|  | JUNB |  |  |  |
|  | LEPR |  |  |  |
|  | MCOLN1 |  |  |  |
|  | PHF1 |  |  |  |
|  | CLU |  |  |  |
|  | MAP2K1 |  |  |  |
|  | NDUFA13 |  |  |  |
|  | GBP6 |  |  |  |
|  | L3MBTL3 |  |  |  |
|  | ABCA8 |  |  |  |
|  | ALPP |  |  |  |
|  | OCLN |  |  |  |
|  | RPS23 |  |  |  |
|  | CIDEB |  |  |  |
|  | SUMF1 |  |  |  |
|  | DENND5A |  |  |  |
|  | TXNIP |  |  |  |
|  | USP3 |  |  |  |
|  | IGF1R |  |  |  |
|  | CRP |  |  |  |
|  | BAK1 |  |  |  |
|  | DMAC2L |  |  |  |
|  | PTGIR |  |  |  |
|  | BCL2L1 |  |  |  |
|  | EIF4EBP1 |  |  |  |
|  | ALPI |  |  |  |
|  | UCP2 |  |  |  |
|  | CLCN7 |  |  |  |
|  | IGSF10 |  |  |  |
|  | NDUFA8 |  |  |  |
|  | HPS1 |  |  |  |
|  | LDLRAP1 |  |  |  |
|  | PLSCR4 |  |  |  |
|  | RASAL1 |  |  |  |
|  | SCRIB |  |  |  |
|  | MMP2 |  |  |  |
|  | ITGA11 |  |  |  |
|  | MRPL4 |  |  |  |
|  | PALD1 |  |  |  |
|  | SDSL |  |  |  |
|  | NCOA6 |  |  |  |
|  | TAC1 |  |  |  |
|  | CCDC85A |  |  |  |
|  | IL13 |  |  |  |
|  | CAV1 |  |  |  |
|  | ITGAE |  |  |  |
|  | MYL2 |  |  |  |
|  | DDIT4 |  |  |  |
|  | MRPL45 |  |  |  |
|  | TRPM6 |  |  |  |
|  | SH3TC1 |  |  |  |
|  | CP |  |  |  |
|  | PHLDB3 |  |  |  |
|  | FOSL1 |  |  |  |
|  | MAPK4 |  |  |  |
|  | INSIG1 |  |  |  |
|  | NDUFB9 |  |  |  |
|  | IGFBP3 |  |  |  |
|  | TIAM2 |  |  |  |
|  | IL18RAP |  |  |  |
|  | JAZF1 |  |  |  |
|  | KLRG1 |  |  |  |
|  | CERS5 |  |  |  |
|  | FAM241A |  |  |  |
|  | SGK1 |  |  |  |
|  | ISCU |  |  |  |
|  | NDRG1 |  |  |  |
|  | CXCR4 |  |  |  |
|  | TLR2 |  |  |  |
|  | ZBTB18 |  |  |  |
|  | BTG2 |  |  |  |
|  | MRPS14 |  |  |  |
|  | NOS1 |  |  |  |
|  | ARG1 |  |  |  |
|  | EID3 |  |  |  |
|  | ITPKC |  |  |  |
|  | MPO |  |  |  |
|  | MRPS12 |  |  |  |
|  | HLA-DQB1 |  |  |  |
|  | MRPL46 |  |  |  |
|  | NDUFA11 |  |  |  |
|  | FOXO1 |  |  |  |
|  | PKD1L2 |  |  |  |
|  | GPR18 |  |  |  |
|  | IL17A |  |  |  |
|  | PDK4 |  |  |  |
|  | CDK6 |  |  |  |
|  | MRPL51 |  |  |  |
|  | CCND2 |  |  |  |
|  | PLEKHM1 |  |  |  |
|  | SOCS3 |  |  |  |
|  | EPG5 |  |  |  |
|  | ACAD8 |  |  |  |
|  | ALOX5 |  |  |  |
|  | CARS2 |  |  |  |
|  | TTR |  |  |  |
|  | APAF1 |  |  |  |
|  | MRPL16 |  |  |  |
|  | MRPS18A |  |  |  |
|  | APOL6 |  |  |  |
|  | CD300A |  |  |  |
|  | SACM1L |  |  |  |
|  | ALDH2 |  |  |  |
|  | AREG |  |  |  |
|  | BNIP3 |  |  |  |
|  | EGFR |  |  |  |
|  | KATNAL1 |  |  |  |
|  | SLC3A2 |  |  |  |
|  | DMC1 |  |  |  |
|  | G6PC1 |  |  |  |
|  | MMP13 |  |  |  |
|  | PKM |  |  |  |
|  | TRPV1 |  |  |  |
|  | IER3 |  |  |  |
|  | F2 |  |  |  |
|  | DCUN1D3 |  |  |  |
|  | ID2 |  |  |  |
|  | DUSP6 |  |  |  |
|  | HGF |  |  |  |
|  | ACP6 |  |  |  |
|  | MRPL52 |  |  |  |
|  | SLC22A9 |  |  |  |
|  | TMPRSS6 |  |  |  |
|  | ZNF644 |  |  |  |
|  | ID1 |  |  |  |
|  | ANXA1 |  |  |  |
|  | MRPL34 |  |  |  |
|  | PLIN2 |  |  |  |
|  | HSP90B1 |  |  |  |
|  | TF |  |  |  |
|  | CTH |  |  |  |
|  | GRSF1 |  |  |  |
|  | MRPL30 |  |  |  |
|  | MRPL35 |  |  |  |
|  | NHS |  |  |  |
|  | RDH14 |  |  |  |
|  | GPX3 |  |  |  |
|  | IL12A |  |  |  |
|  | ACSL1 |  |  |  |
|  | FKBP5 |  |  |  |
|  | CACNG4 |  |  |  |
|  | HSPA9 |  |  |  |
|  | FABP1 |  |  |  |
|  | TXN |  |  |  |
|  | HK2 |  |  |  |
|  | LY6K |  |  |  |
|  | RIOX1 |  |  |  |
|  | PEX12 |  |  |  |
|  | ADM |  |  |  |
|  | NHLRC3 |  |  |  |
|  | PTK2 |  |  |  |
|  | S100A9 |  |  |  |
|  | CD40 |  |  |  |
|  | CAPS2 |  |  |  |
|  | NOL7 |  |  |  |
|  | SELE |  |  |  |
|  | CFLAR |  |  |  |
|  | IL1R1 |  |  |  |
|  | S100A8 |  |  |  |
|  | ACTA2 |  |  |  |
|  | CACNA2D4 |  |  |  |
|  | KLF4 |  |  |  |
|  | CYP4A8 |  |  |  |
|  | ADO |  |  |  |
|  | ILDR1 |  |  |  |
|  | HES1 |  |  |  |
|  | LIF |  |  |  |
|  | MRPL53 |  |  |  |
|  | SCRG1 |  |  |  |
|  | FCF1 |  |  |  |
|  | CDH1 |  |  |  |
|  | DCSTAMP |  |  |  |
|  | CYP2C70 |  |  |  |
|  | FAS |  |  |  |
|  | SLC10A1 |  |  |  |
|  | BHLHE40 |  |  |  |
|  | ACLY |  |  |  |
|  | TNFRSF11B |  |  |  |
|  | SNAI2 |  |  |  |
|  | LMNA |  |  |  |
|  | CDKN1A |  |  |  |
|  | TFF1 |  |  |  |
|  | MRPS15 |  |  |  |
|  | SENP8 |  |  |  |
|  | KBTBD7 |  |  |  |
|  | PRKAA1 |  |  |  |
|  | ZNF589 |  |  |  |
|  | SLC2A3 |  |  |  |
|  | ATP2A2 |  |  |  |
|  | B3GALNT2 |  |  |  |
|  | KNG1 |  |  |  |
|  | ACE2 |  |  |  |
|  | TFAM |  |  |  |
|  | CCDC121 |  |  |  |
|  | SF3B6 |  |  |  |
|  | EHHADH |  |  |  |
|  | MT2 |  |  |  |
|  | SPARC |  |  |  |
|  | APP |  |  |  |
|  | CREBBP |  |  |  |
|  | MIR98 |  |  |  |
|  | GTF2E1 |  |  |  |
|  | BCL2 |  |  |  |
|  | DNAJB9 |  |  |  |
|  | MGLL |  |  |  |
|  | SLC22A1 |  |  |  |
|  | CSF1 |  |  |  |
|  | CHAC1 |  |  |  |
|  | RHOB |  |  |  |
|  | THRSP |  |  |  |
|  | ZFP36 |  |  |  |
|  | CALHM6 |  |  |  |
|  | CYCS |  |  |  |
|  | VDAC1 |  |  |  |
|  | HMGCS2 |  |  |  |
|  | CYP27A1 |  |  |  |
|  | ACSL4 |  |  |  |
|  | SAT1 |  |  |  |
|  | EGR1 |  |  |  |
|  | ACADM |  |  |  |
|  | ALAS1 |  |  |  |
|  | F2R |  |  |  |
|  | EPO |  |  |  |
|  | TUBA1A |  |  |  |
|  | CCNG2 |  |  |  |
|  | ELOVL6 |  |  |  |
|  | IRF1 |  |  |  |
|  | TLR4 |  |  |  |
|  | VIM |  |  |  |
|  | PLK2 |  |  |  |
|  | ISG15 |  |  |  |
|  | G0S2 |  |  |  |
|  | DPYD |  |  |  |
|  | INS1 |  |  |  |
|  | M1AP |  |  |  |
|  | VCAM1 |  |  |  |
|  | ATP1A1 |  |  |  |
|  | FGFR1 |  |  |  |
|  | CXCL9 |  |  |  |
|  | ENPP2 |  |  |  |
|  | PRKCE |  |  |  |
|  | EDN1 |  |  |  |
|  | CYP7B1 |  |  |  |
|  | MYH7 |  |  |  |
|  | ANXA3 |  |  |  |
|  | FMO1 |  |  |  |
|  | HNF4A |  |  |  |
|  | NT5E |  |  |  |
|  | CALHM5 |  |  |  |
|  | PRP4K |  |  |  |
|  | FN1 |  |  |  |
|  | HP |  |  |  |
|  | CFL1 |  |  |  |
|  | S100A6 |  |  |  |
|  | BTG1 |  |  |  |
|  | TIMP1 |  |  |  |
|  | CXCL1 |  |  |  |
|  | CYP24A1 |  |  |  |
|  | GADD45A |  |  |  |
|  | SERPINE2 |  |  |  |
|  | PIR |  |  |  |
|  | PRDX3 |  |  |  |
|  | HSPA2 |  |  |  |
|  | TUBB2A |  |  |  |
|  | CTSS |  |  |  |
|  | NEDD9 |  |  |  |
|  | IL11 |  |  |  |
|  | FOSL2 |  |  |  |
|  | ALDH3A2 |  |  |  |
|  | ITGA5 |  |  |  |
|  | LIPC |  |  |  |
|  | IL1RL1 |  |  |  |
|  | IL6ST |  |  |  |
|  | LEP |  |  |  |
|  | UCP1 |  |  |  |
|  | CDC42 |  |  |  |
|  | TPM3 |  |  |  |
|  | ADA |  |  |  |
|  | PLK3 |  |  |  |
|  | STC1 |  |  |  |
|  | HMGCR |  |  |  |
|  | PECAM1 |  |  |  |
|  | SLCO1A1 |  |  |  |
|  | ACACB |  |  |  |
|  | MMP9 |  |  |  |
|  | HEXB |  |  |  |
|  | FBP1 |  |  |  |
|  | LITAF |  |  |  |
|  | NFATC1 |  |  |  |
|  | INHBE |  |  |  |
|  | FADS1 |  |  |  |
|  | LIPG |  |  |  |
|  | RAN |  |  |  |
|  | NFKBIB |  |  |  |
|  | COX4I1 |  |  |  |
|  | IL1R2 |  |  |  |
|  | SLC22A7 |  |  |  |
|  | TRPA1 |  |  |  |
|  | AK4 |  |  |  |
|  | RBP1 |  |  |  |
|  | GUSB |  |  |  |
|  | LBP |  |  |  |
|  | RGS4 |  |  |  |
|  | ITGAV |  |  |  |
|  | KLF2 |  |  |  |
|  | MMP10 |  |  |  |
|  | CCNA2 |  |  |  |
|  | ERO1A |  |  |  |
|  | STAR |  |  |  |
|  | BDKRB2 |  |  |  |
|  | CCR7 |  |  |  |
|  | F10 |  |  |  |
|  | PHLDA2 |  |  |  |
|  | MAPK14 |  |  |  |
|  | ABCD3 |  |  |  |
|  | NFAT5 |  |  |  |
|  | ETV7 |  |  |  |
|  | SDF2L1 |  |  |  |
|  | CEBPB |  |  |  |
|  | GPAT3 |  |  |  |
|  | OGDH |  |  |  |
|  | KCNMA1 |  |  |  |
|  | DDIT3 |  |  |  |
|  | MAP3K8 |  |  |  |
|  | GEM |  |  |  |
|  | CCL3 |  |  |  |
|  | GLO1 |  |  |  |
|  | NDUFS1 |  |  |  |
|  | AGTR1 |  |  |  |
|  | PTEN |  |  |  |
|  | STAT1 |  |  |  |
|  | CCN2 |  |  |  |
|  | ACADVL |  |  |  |
|  | NLRP3 |  |  |  |
|  | ATP2B4 |  |  |  |
|  | SAA1 |  |  |  |
|  | RXRG |  |  |  |
|  | NIBAN1 |  |  |  |
|  | LPL |  |  |  |
|  | PROCR |  |  |  |
|  | AKR1D1 |  |  |  |
|  | POMC |  |  |  |
|  | SORBS1 |  |  |  |
|  | PTGS1 |  |  |  |
|  | DUSP1 |  |  |  |
|  | LDLR |  |  |  |
|  | PTAFR |  |  |  |
|  | PRKY |  |  |  |
|  | OGA |  |  |  |
|  | ACSM3 |  |  |  |
|  | BAD |  |  |  |
|  | SRC |  |  |  |
|  | CSF2 |  |  |  |
|  | GPT |  |  |  |
|  | SLC38A1 |  |  |  |
|  | DHRS9 |  |  |  |
|  | UQCRC2 |  |  |  |
|  | CACNA2D1 |  |  |  |
|  | ANKRD37 |  |  |  |
|  | STAT3 |  |  |  |
|  | CCDC80 |  |  |  |
|  | ALPL |  |  |  |
|  | CASP9 |  |  |  |
|  | CENPU |  |  |  |
|  | ETFA |  |  |  |
|  | SEMA6D |  |  |  |
|  | PPP1CB |  |  |  |
|  | SLCO1A2 |  |  |  |
|  | VGF |  |  |  |
|  | GAPDH |  |  |  |
|  | METRNL |  |  |  |
|  | NINJ1 |  |  |  |
|  | CYP1B1 |  |  |  |
|  | DDR1 |  |  |  |
|  | CXCL12 |  |  |  |
|  | SEC61A1 |  |  |  |
|  | ERN1 |  |  |  |
|  | TRIB3 |  |  |  |
|  | ATP5PD |  |  |  |
|  | HSPD1 |  |  |  |
|  | PLA1A |  |  |  |
|  | PRXL2A |  |  |  |
|  | TNFRSF10B |  |  |  |
|  | ASNS |  |  |  |
|  | AGPAT2 |  |  |  |
|  | ATP5F1D |  |  |  |
|  | NFE2L2 |  |  |  |
|  | OSBPL3 |  |  |  |
|  | PPARA |  |  |  |
|  | HSPA5 |  |  |  |
|  | SRXN1 |  |  |  |
|  | SCD |  |  |  |
|  | NR4A1 |  |  |  |
|  | RETREG1 |  |  |  |
|  | HSD17B12 |  |  |  |
|  | SLC2A4 |  |  |  |
|  | OTC |  |  |  |
|  | FRMD4A |  |  |  |
|  | IKBKB |  |  |  |
|  | POU2F1 |  |  |  |
|  | GCLC |  |  |  |
|  | FJX1 |  |  |  |
|  | GSTP1 |  |  |  |
|  | CCL2 |  |  |  |
|  | IL4 |  |  |  |
|  | ANGPTL4 |  |  |  |
|  | ATP5MF |  |  |  |
|  | ATP5PF |  |  |  |
|  | ITGAM |  |  |  |
|  | PPP1R15A |  |  |  |
|  | NOX4 |  |  |  |
|  | CDK9 |  |  |  |
|  | ME1 |  |  |  |
|  | UQCRH |  |  |  |
|  | GSTT1 |  |  |  |
|  | MRPL19 |  |  |  |
|  | TTLL7 |  |  |  |
|  | BAX |  |  |  |
|  | GSK3B |  |  |  |
|  | CCND1 |  |  |  |
|  | NOS3 |  |  |  |
|  | CLSTN2 |  |  |  |
|  | SERPINE1 |  |  |  |
|  | CCNB1 |  |  |  |
|  | GPX1 |  |  |  |
|  | ABCC2 |  |  |  |
|  | CRYAB |  |  |  |
|  | FASN |  |  |  |
|  | ABCB9 |  |  |  |
|  | CYP3A4 |  |  |  |
|  | CYP1A2 |  |  |  |
|  | AKR1B10 |  |  |  |
|  | EPHX2 |  |  |  |
|  | SQLE |  |  |  |
|  | CEBPD |  |  |  |
|  | SOX13 |  |  |  |
|  | FOS |  |  |  |
|  | VEGFA |  |  |  |
|  | ALDOA |  |  |  |
|  | CAT |  |  |  |
|  | PRKCB |  |  |  |
|  | DENND1B |  |  |  |
|  | DIO2 |  |  |  |
|  | CREB1 |  |  |  |
|  | GCLM |  |  |  |
|  | ZNF395 |  |  |  |
|  | NFKBIA |  |  |  |
|  | IGFBP5 |  |  |  |
|  | SOD1 |  |  |  |
|  | MRPS34 |  |  |  |
|  | RELA |  |  |  |
|  | PDK1 |  |  |  |
|  | FAR2 |  |  |  |
|  | ABCG5 |  |  |  |
|  | IL18 |  |  |  |
|  | CXCL5 |  |  |  |
|  | CDKN1C |  |  |  |
|  | DGAT2 |  |  |  |
|  | JAG1 |  |  |  |
|  | IGFBP2 |  |  |  |
|  | PKLR |  |  |  |
|  | CCL5 |  |  |  |
|  | ACAT2 |  |  |  |
|  | AKT1 |  |  |  |
|  | ALB |  |  |  |
|  | CBS |  |  |  |
|  | MTTP |  |  |  |
|  | CITED2 |  |  |  |
|  | CYP8B1 |  |  |  |
|  | CFB |  |  |  |
|  | CXCL10 |  |  |  |
|  | CD36 |  |  |  |
|  | MYC |  |  |  |
|  | SLC2A1 |  |  |  |
|  | ABCB1 |  |  |  |
|  | CPT1A |  |  |  |
|  | CYP2E1 |  |  |  |
|  | IL12B |  |  |  |
|  | GSR |  |  |  |
|  | TRAF6 |  |  |  |
|  | ETS2 |  |  |  |
|  | DEPP1 |  |  |  |
|  | GCG |  |  |  |
|  | INSIG2 |  |  |  |
|  | NFKB1 |  |  |  |
|  | ATP8B1 |  |  |  |
|  | MIR16 |  |  |  |
|  | CROT |  |  |  |
|  | SQSTM1 |  |  |  |
|  | CMBL |  |  |  |
|  | TGFB1 |  |  |  |
|  | CTNNB1 |  |  |  |
|  | NGF |  |  |  |
|  | AGT |  |  |  |
|  | NQO1 |  |  |  |
|  | PTGS2 |  |  |  |
|  | MMP3 |  |  |  |
|  | MMP8 |  |  |  |
|  | TP53 |  |  |  |
|  | ASPA |  |  |  |
|  | SREBF1 |  |  |  |
|  | IL10 |  |  |  |
|  | CYP1A1 |  |  |  |
|  | SLC2A2 |  |  |  |
|  | MAPK1 |  |  |  |
|  | CXCL8 |  |  |  |
|  | ABCC3 |  |  |  |
|  | ACACA |  |  |  |
|  | PLA2G2A |  |  |  |
|  | IFNG |  |  |  |
|  | IL6 |  |  |  |
|  | HMOX1 |  |  |  |
|  | TNF |  |  |  |
|  | CASP3 |  |  |  |
|  | MAPK3 |  |  |  |
|  | ABCB11 |  |  |  |
|  | IL1B |  |  |  |
|  | NOS2 |  |  |  |
|  | TNFSF11 |  |  |  |
|  | ICAM1 |  |  |  |
|  | C1S |  |  |  |
|  | CTSC |  |  |  |
|  | CXCL12 |  |  |  |
|  | FGF2 |  |  |  |
|  | TNFSF11 |  |  |  |
