# Supplementary material for: Immunomodulatory Mechanism of Baiyaojian Decoction on Periodontitis: Network Pharmacology, Single‐Cell RNA Sequencing and Molecular Docking
Source: J Cell Mol Med. 2026 Jan 28;30(3):e71034. doi: 10.1111/jcmm.71034 (PMC12851902; doi:10.1111/jcmm.71034)
Supplement: Supplementary file 5 — Table S4: Potential therapeutic targets of Baiyaojian decoction in the treatment of periodontitis. [file JCMM-30-e71034-s005.docx]

**Supplementary Table 4 Potential therapeutic targets of Baiyaojian decoction in the treatment of periodontitis**

RXRA,CYP19A1,MAPK14,CNR1,AR,MAPK1,ADORA3,NCSTN,ESR1,ESR2,PRKCD,C5AR1,CHRM3,CCR1,CYP1B1,ADORA1,ABCG2,HSD17B1,ABCC1,SHBG,MMP13,PTGS1,MMP12,SRC,CHRNA7,TERT,SERPINE1,PLG,ACHE,GUSB,KDR,AKR1B1,CDK1,CCNB1,CDK2,NTRK1,DNMT1,FUT4,STAT1,MMP2,IGF1R,MMP9,ODC1,PARP1,TNKS,MET,ABCB1,PPARG,PLA2G2A,CCNA2,HSD17B2,BCL2,CYP1A2,GABRA5,NOS2,ADORA2B,JAK2,HDAC1,PTGER1,CCNE2,MTOR,PIK3CD,PIK3CB,PIK3CG,PIK3CA,PDE5A,PRKDC,GYS1,TGFBR1,PTK2B,MAPK8,CCR2,PTGER4,PTGER2,PTGER3,PTGS2,CNR2,MAP2K1,SRD5A1,ELANE,CSF1R,GSK3B,CTSS,CTSL,FLT1,CCND3,CCND1,CCND2,CXCR2,CFD,PPIA,BDKRB1,EGFR,ADORA2A,HTR1A,CTSK,TRPA1,IKBKB,MMP3,MMP1,ITGAV,ITGB3,TRPV1,NTRK2,MAPK10,MAPK9,FAP,P2RX7,PTK2,ALOX5,ERBB2,PDGFRA,TTR,PDGFRB,ALPL,MIF,GRK6,TLR4,CYP2C9,CYP3A4,F3,NFE2L2,STAT3,CSNK2A1,TEK,FGR,NFKB1,CFTR,GSR,HSP90AA1,AKT1,CD38,AHR,ASAH1,ADAMTS5,IDO1,CHEK2,PDE4C,MMP8,DRD1,PLAU,ICAM1,SELE,CTSB,ALOX15,KIT,F2,MPO,PIK3R1,PYGL,CXCR1,AXL,MCL1,MME,IGFBP3,ACE,ECE1,FABP4,EGLN1,AGTR1,ERN1,CASP3,FBP1,CASP7,CASP8,CASP1,HIF1A,CCR4,CYP1A1,KDM6B,FTO,RELA,TLR9,NGFR,TOP2A,CTNNB1,HSP90B1,LDHA,BCL2L1,IL1B,PRKCA,JUN,PTGES,HMOX1,LIPA,MAPK3,ITGAL,TYMS,MAPT,MYLK,ESRRA,METAP2,ATM,PLA2G7,PDE2A,SLC6A4,MDM2,HDAC6,OPRM1,KDM1A,PREP,CTSC,HTR2C,TGM2,MALT1,NAMPT,HTR2A
